# Piercing of Consciousness as a Threshold-Crossing Operation

**DOI:** 10.1016/j.cub.2017.06.047

**Published:** 2017-08-07

**Authors:** Yul H.R. Kang, Frederike H. Petzschner, Daniel M. Wolpert, Michael N. Shadlen

**Affiliations:** 1 Department of Neuroscience, Zuckerman Mind Brain Behavior Institute, Columbia University, New York, NY 10032, USA; 2 Translational Neuromodeling Unit (TNU), Institute for Biomedical Engineering, University of Zurich and ETH Zurich, 8032 Zurich, Switzerland; 3 Computational and Biological Learning Laboratory, Department of Engineering, Cambridge University, Cambridge CB2 1PZ, UK; 4 Kavli Institute, Columbia University, New York, NY 10032, USA; 5 Howard Hughes Medical Institute, Columbia University, New York, NY 10032, USA

**Keywords:** consciousness, decision making, mental chronometry, motion perception, reaction time

## Abstract

Many decisions arise through an accumulation of evidence to a terminating threshold. The process, termed bounded evidence accumulation (or drift diffusion), provides a unified account of decision speed and accuracy, and it is supported by neurophysiology in human and animal models. In many situations, a decision maker may not communicate a decision immediately and yet feel that at some point she had made up her mind. We hypothesized that this occurs when an accumulation of evidence reaches a termination threshold, registered, subjectively, as an “aha” moment. We asked human participants to make perceptual decisions about the net direction of dynamic random dot motion. The difficulty and viewing duration were controlled by the experimenter. After indicating their choice, participants adjusted the setting of a clock to the moment they felt they had reached a decision. The subjective decision times (*t*_*SD*_s) were faster on trials with stronger (easier) motion, and they were well fit by a bounded drift-diffusion model. The fits to the *t*_*SD*_s alone furnished parameters that fully predicted the choices (accuracy) of four of the five participants. The quality of the prediction provides compelling evidence that these subjective reports correspond to the terminating process of a decision rather than a post hoc inference or arbitrary report. Thus, conscious awareness of having reached a decision appears to arise when the brain’s representation of accumulated evidence reaches a threshold or bound. We propose that such a mechanism might play a more widespread role in the “piercing of consciousness” by non-conscious thought processes.

## Highlights

• Perceptual decisions can arise through an accumulation of evidence to a threshold
• After a stimulus, participants set a clock to the moment they had reached a decision
• An evidence accumulation model fit to these times allowed predictions of accuracy
• The sense of having decided is mediated by a threshold on accumulated evidence

## Teaser

Decision makers often feel there is a moment of having reached a decision during deliberation. Kang and Petzschner et al. asked subjects to report this time by setting a clock. The subjective decision times predicted choice accuracy, suggesting that awareness of completion is mediated by a threshold crossing on accumulated evidence.

## Introduction

We are not consciously aware of all of the information delivered from the senses to the brain, nor are we aware of the operations that underlie the thoughts that do pierce consciousness. Indeed, the transition from non-conscious processing to conscious awareness is one of the great mysteries of psychology and neuroscience. In a series of classic studies, Libet and colleagues used “mental chronometry” to identify the time that human volunteers felt they made a conscious decision to initiate a movement [1, 2, 3]. Libet suggested that this was the moment that subjects “willed” their movement. He and others to follow were fascinated by the observation that neural events related to the movement could be detected hundreds of milliseconds before the subjects were aware [4], leading to philosophical speculation about volition and free will. However, it is unsurprising that neural events would precede conscious awareness. Indeed, it has been suggested that the moment of awareness might reflect the completion of a decision process [5, 6]—in this case, a commitment to a proposition to move.

Studies of decision making in animals and humans indicate that many decisions arise from an accumulation of evidence to a criterion. The process, termed bounded evidence accumulation or bounded drift diffusion, explains the speed and accuracy of many types of decisions, including recognition memory, food preference, and perceptual category [7, 8, 9]. The mechanism is especially well suited to explain perceptual decisions that are informed by a sequence of independent, noisy samples of evidence. For example, when humans and monkeys are asked to decide the net direction of motion (e.g., left versus right) of a dynamic random dot display, their choices and reaction times (RTs) are explained by a model in which evidence is accumulated until it reaches one of two bounds, thereby determining which decision is made and marking the end of deliberation. The mechanism is supported by neural recordings in human, nonhuman primates, and rodents, which demonstrate neural correlates of evidence accumulation and termination thresholds [8, 10, 11, 12, 13, 14, 15].

Termination thresholds might also apply to decisions that are not communicated immediately, as they are in reaction time studies, but instead occur without any overt sign of completion. Even without time pressure, a decision maker might terminate a decision covertly before all of the evidence has been received and thus ignore potentially useful information. Without an accompanying behavior, such termination has been deduced indirectly by analyzing decisions and showing that they are not affected by the late arrival of evidence [16]. However, this conclusion is not widely accepted [17]. We hypothesized that a putative termination threshold might be registered, subjectively, as an “aha” moment, similar to the moment that Libet’s participants reported about their will to move. We therefore set out to test whether mental chronometry marks decision termination. Up to now, it has been thought that objective validation of a subjective decision time is a logical impossibility, given the absence of an objective manifestation with which to compare it [18]. However, bounded evidence accumulation models furnish a test of a stringent prediction: if subjective times correspond to decision termination, then they ought to predict decision accuracy. Here, we test this prediction and show that they do.

## Results

### Experiment 1: Controlled Viewing Duration with Subjective Decision Times

**Figure 1:**
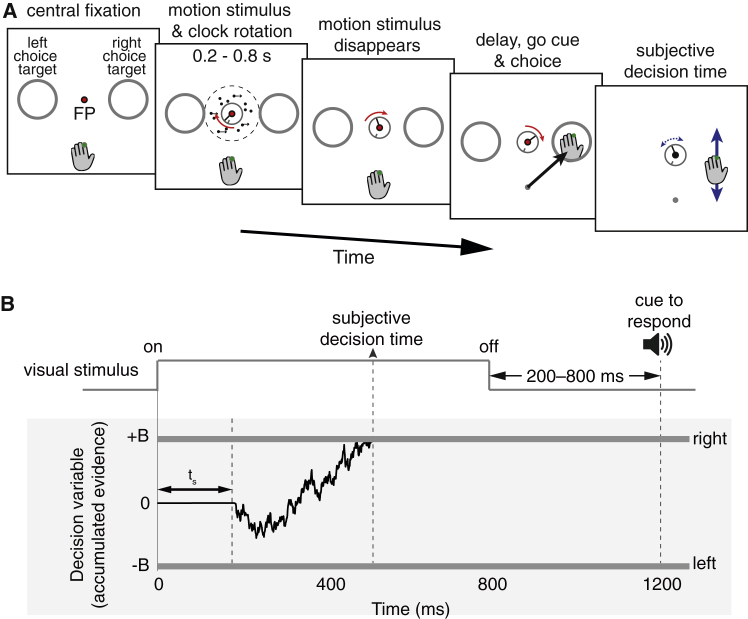
Subjective Report of Decision Termination in a Perceptual Task (A) Controlled-duration task. On each trial, participants fixated a central fixation point (FP). A random dot motion stimulus then appeared at the same time as a central clock started rotating. Participants were asked to judge the direction of motion (left versus right) and also the position of the clock hand at the time they made their decision. After a computer-controlled time (0.2–0.8 s), the motion stimulus was extinguished, and after a delay (0.2–0.8 s), a tone sounded and participants indicated the perceived motion direction by moving the cursor to one of two choice targets. They then reported their subjective decision time by moving a stylus to position the clock hand at the remembered clock location at the time of their decision about the motion direction (see STAR Methods, Methods S1 and S2, and Figure S1). (B) Information flow diagram showing visual stimulus and hypothesized events leading to a decision. The visual stimulus gives rise to a decision variable (black trace) that is the accumulation of noisy evidence. The decision is complete when a “right” or “left” bound is crossed (that is, when ±*B* of evidence has accumulated). The example illustrates a trial that gives rise to a rightward choice with decision time around 500 ms, although the stimulus lasts 800 ms. Data from neural recordings [16, 19] suggest that the delay from motion onset to the beginning of the accumulation (*t*_*s*_) is around 200 ms. In general, the reported subjective decision time (*t*_*SD*_) might differ from the actual moment of decision termination by additional delays attributed to perceptual and cognitive operations associated with storage and recall of the clock position.

Five participants performed a direction discrimination task in which they were asked to decide the net direction of dynamic random dots, viewed on a computer display (Figure 1A). The difficulty of the decision was controlled by the probability, *C*, that each dot will reappear Δ*t* later, either at displacement, Δ*x*, along an axis of motion, or randomly replaced by a new dot (see STAR Methods). We refer to *C* as the motion coherence (or motion strength) and use its sign to indicate a direction. Both the direction and strength of motion were randomized from trial to trial, and viewing duration was controlled by the experimenter. Besides the random dot motion, the display consisted of a central fixation point, two “choice targets,” and a “clock”. After the motion display ended and an additional delay period, participants indicated their decision about the direction of motion by using a hand-held stylus to move a cursor to the left or right choice target. They were then asked to restore the clock “handle” to the position it had attained at the moment they felt they had decided the direction, what we term “subjective decision time”. The participants received extensive training on the use of the clock (see STAR Methods and Methods S1 and S2), and we ensured that they could use the clock accurately to report the time of an auditory cue presented at a random time during motion viewing (Figure S1).

**Figure 2:**
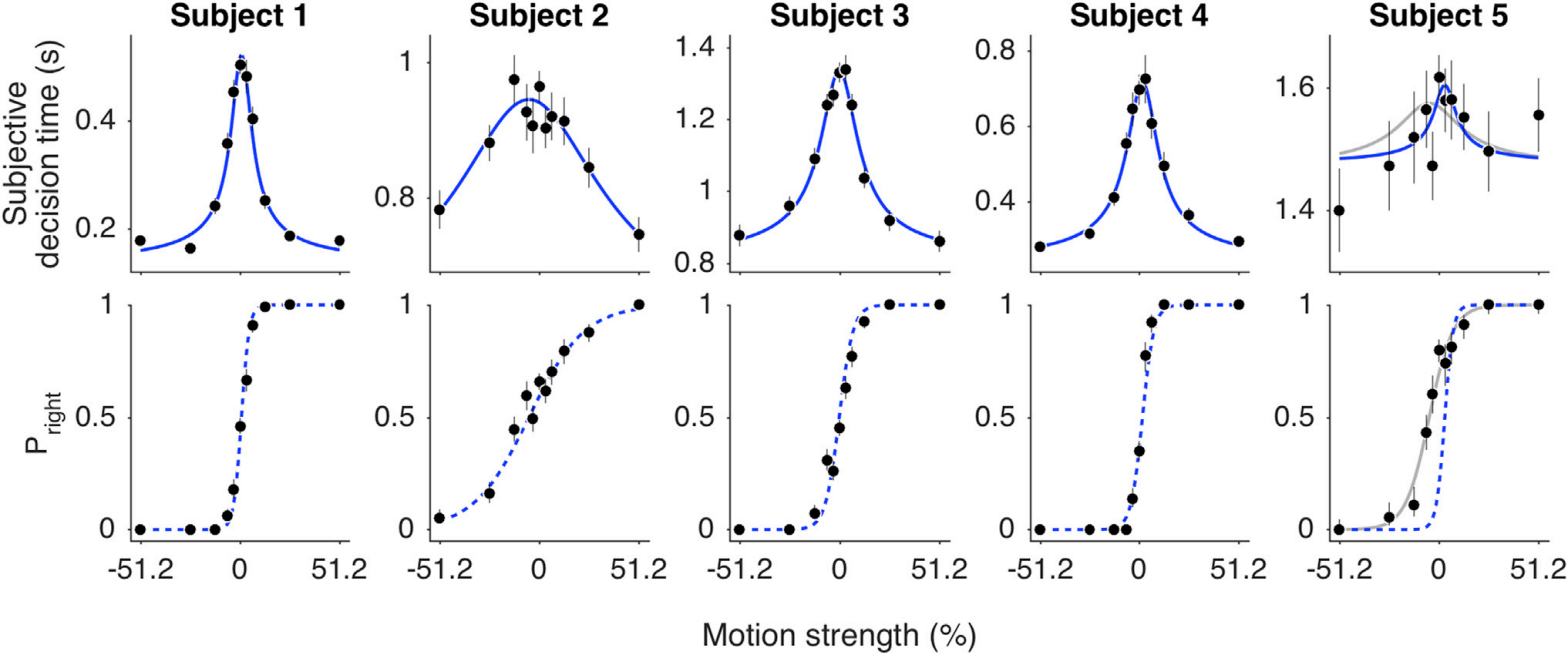
Subjective Decision Times Reflect Termination of a Decision Process Data are from five participants tested on a controlled-duration task for the trials in which the motion display lasted 800 ms. Subjective decision times (top) and proportion of rightward choices (bottom) are plotted as a function of motion strength (negative and positive values indicate leftward and rightward direction, respectively). Blue solid lines are drift-diffusion fits to the *t*_*SD*_ data, and blue dashed lines are predictions using the parameters of the *t*_*SD*_ fits (parameters in Table 1). For subject 5, the gray lines are the joint fits to the *t*_*SD*_ and choice. Points are means ± SEM.

Subjective decision times (*t*_*SD*_s) varied as a function of motion strength. The data in the top row of Figure 2 were obtained using a motion stimulus duration of 800 ms. The *t*_*SD*_s were shortest when the motion was strong and longest when the motion was weak. This pattern was statistically reliable for four of five subjects as well as at a group level (p < 10^−6^; GLM; see STAR Methods). The pattern is qualitatively similar to mean response times observed in free-response paradigms, in which viewers are allowed to indicate their decision with an action whenever ready (e.g., [20]). The solid blue curves in these panels are fits of a parsimonious drift-diffusion model to the mean *t*_*SD*_s (see Figure 1B and STAR Methods), treating them as if they are reaction times. The idea is that a decision completes when the accumulation of noisy samples of evidence reaches an upper or lower bound. The shape of the curve is determined by two parameters: (1) a term, *κ*, that determines the evidence drawn at each time step, δ*t*, from a Gaussian distribution with mean *κC*δ*t* and variance δ*t* and (2) the bound height, ±*B*. Translations along the abscissa and ordinate are captured by a coherence bias term (*C*_*0*_) (see [21]) and a non-decision time (*t*_*ND*_), respectively (parameters in Table 1). Based on the *t*_*ND*_, the actual time of decision termination occurred within the stimulus duration for subjects 1–4. By eye, the fits capture the data reasonably well for all subjects except subject 5. Thus, for four of the subjects, *t*_*SD*_s appear to conform to the same regularities as explicit reaction times. To evaluate this assertion, the same diffusion model should account for the choices the subjects made about direction.

**Table 1:** Parameters of the Drift-Diffusion Model Fit to the *t*_*SD*_ Data in the Controlled-Duration Task

	*B*	*κ*	*C*_*0*_	*t*_*ND*_
Subject 1	0.62 ± 0.01	40.4 ± 3.3	0.007 ± 0.004	0.131 ± 0.006
Subject 2	0.62 ± 0.13	5.7 ± 3.9	−0.053 ± 0.030	0.562 ± 0.166
Subject 3	0.74 ± 0.02	19.2 ± 2.6	−0.001 ± 0.006	0.790 ± 0.027
Subject 4	0.70 ± 0.02	24.3 ± 3.4	0.018 ± 0.007	0.227 ± 0.015
Subject 5	0.33 ± 0.10	24.6 ± 7.7	−0.050 ± 0.008	1.463 ± 0.050

Parameters are shown ±SE.

The graphs in the lower row of Figure 2 show the influence of motion strength and direction on the subjects’ choices. Decisions were perfectly accurate at the strongest motion strengths (leftmost and rightmost points) and near chance at the weakest motion strengths (middle of the graph). Note that the dashed curves are not fits to the data. They are predictions of the choice proportions from the diffusion model using the parameters derived from the fits to the *t*_*SD*_s. If the *t*_*SD*_s reflect the termination of a bounded diffusion process, then the choice proportions are a logistic function of 2*Bκ*(*C − C*_*0*_), where *C* is signed motion strength. These predictions are remarkably good for subjects 1–4 (p = 0.002, 0.005, 0.045, and 0.01, respectively; comparison with log likelihood of the observed choices given shuffled *t*_*SD*_s; see STAR Methods). For the fifth subject, not surprisingly, we could not use *t*_*SD*_s to predict the choices (p = 0.71). Instead, we show the combined fit of the choice and *t*_*SD*_s from this subject’s data (gray curves). The fit is driven primarily by the choice frequencies (lower panel). A group level analysis using the data from subjects 1–4 reveals that choices were significantly better described by the predictions from the fit to each subject’s own *t*_*SD*_s than by a random combination of the parameters from the other subjects (none of the 531,441 combinations were better than the original; see STAR Methods). From these fits and predictions, we conclude that, for four of the subjects, the *t*_*SD*_ reports correspond to the termination of evidence accumulation and commitment to a perceptual decision.

**Figure 3:**
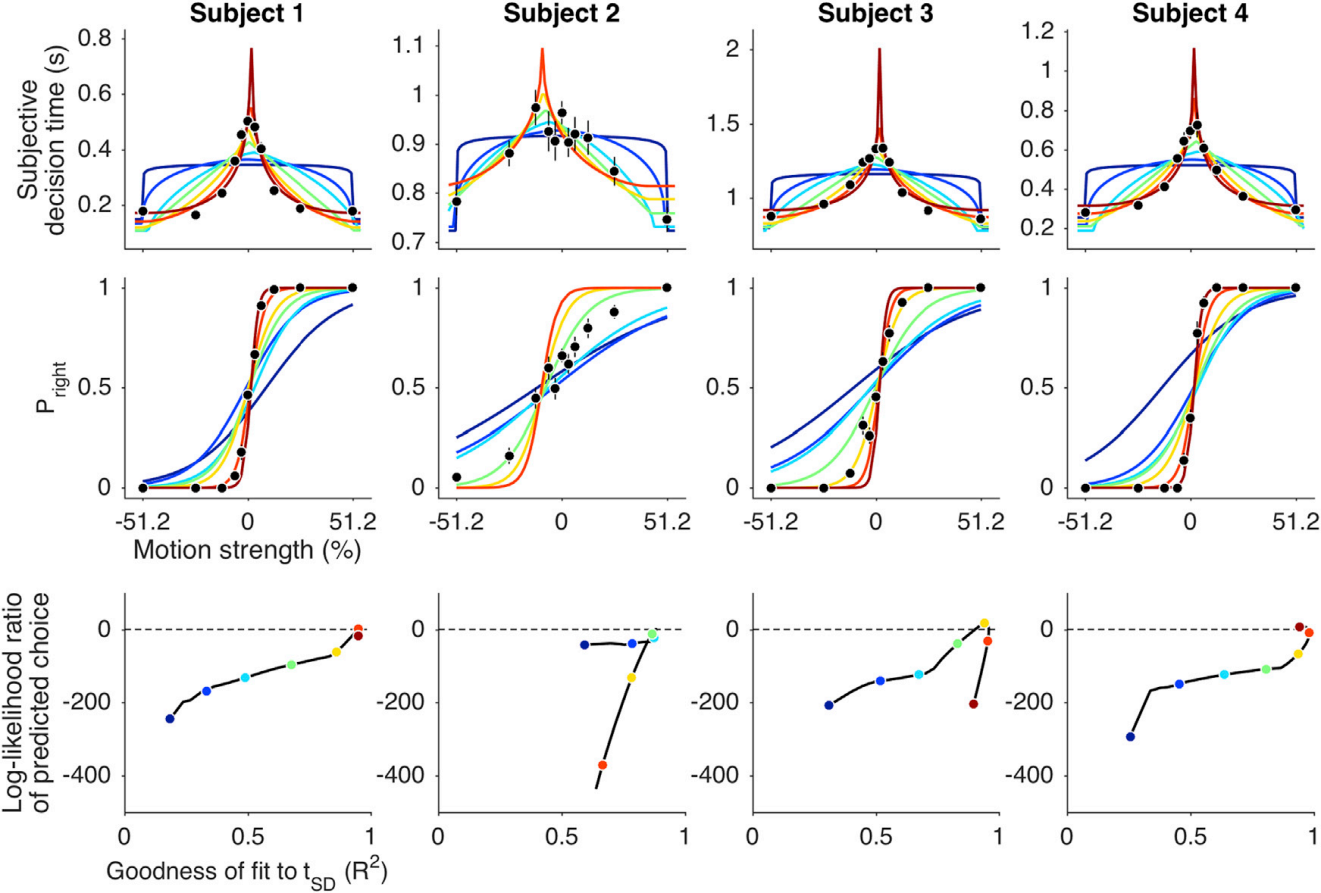
Many Possible Clock Reports Could Reflect Motion Strength but Would Furnish Inferior Predictions of Choice Behavior The *t*_*SD*_s and choice proportions are shown by the black points in the upper two rows (same data as in Figure 2; subjects 1–4). Top row: examples of possible functions of difficulty generated from reflected cumulative beta distributions, scaled and shifted to best fit the observed *t*_*SD*_s. These functions were used to produce surrogate clock settings at the 11 values of motion strength. Middle row: examples of predicted choice functions using the surrogate clock times from the functions in the top row (corresponding colors). The surrogate clock settings (*t*_*surr*_) were fit by Equation 2 to derive parameters that specify the choice function (Equation 1). Bottom row: comparison of the quality of the predictions from surrogate clock times to the prediction obtained from the observed *t*_*SD*_s. Log-likelihood ratio of the choice predictions is shown as a function of the degree to which the surrogates approximate the real data (*R*^*2*^) for a range of possible functions (black trace). The examples in the upper rows are shown by colored points. Log-likelihood ratio less than zero indicates inferior predictions from the surrogate times compared to the observed *t*_*SD*_s. Only functions that do not distort the observed *t*_*SD*_s (*R*^*2*^≈1) predict the choice functions as well as the data. See also Figure S2.

Our main conclusion rests on the capacity to predict the choice functions. We wished to evaluate the assertion that the quality of these predictions suggests that *t*_*SD*_s were in fact indicative of actual terminations of a drift-diffusion process. Clearly, random reports of decision time would not yield sensible predictions, nor does the pattern of *t*_*SD*_s displayed by subject 5. However, one might reasonably ask whether any systematic use of the clock would yield predictions of the choice functions like those in Figure 2. We pursued two approaches to this challenge, shown in Figures 3 and S2.

We considered a broad family of functions that describe an increase in *t*_*SD*_s with difficulty. Figure 3 (top) shows representative functions, which range from a squashed semicircle to a very peaky spike. We used these functions to distort the observed *t*_*SD*_ means from subjects 1–4 and asked whether they could produce reasonable predictions of the choice functions. For each parameterization, we scaled and shifted the function to best fit the observed *t*_*SD*_s. As shown in the top row of Figure 3, some of these functions lie close to the actual data (e.g., yellow peaked, *R*^2^ = 0.88; averaged across subjects 1–4), whereas others do not (e.g., blue semicircle, *R*^2^ = 0.53). We then sampled these curves at the 11 motion strengths to obtain surrogate *t*_*SD*_s and applied the same procedure used on the actual *t*_*SD*_ to predict the choices (Figure 3, middle row). That is, we fit the surrogate *t*_*SD*_s with the parsimonious bounded drift-diffusion model, extracted the three parameters (*B*, *κ*, and *C*_*0*_), and generated the logistic choice predictions. The quality of the prediction is captured by the log likelihood of the 11 observations given the prediction from the surrogate *t*_*SD*_s, which we compare to the log likelihood given the predictions based on the real *t*_*SD*_s (log likelihood ratio; logLR). As shown in the bottom row, the predictions from surrogate *t*_*SD*_s are generally extremely poor (logLR ≪ 0). They only rival the predictions from the actual data when the function approximates the real data (e.g., *R*^2^≈1). The exercise reveals that even modest distortions of the data (i.e., reduced *R*^2^) produce markedly inferior predictions of the choice functions (Figure 3, bottom row). We reached the same conclusion using a second strategy to produce surrogate *t*_*SD*_s, in which we permuted the intervals between the original *t*_*SD*_s, preserving their rank order (Figure S2).

These analyses highlight the precision in our capacity to predict the choice functions in Figure 2 based solely on the clock settings (mean *t*_*SD*_s). Many systematic uses of the clock that might have been used to communicate difficulty would not predict the choices as well as those established by our hypothesis—the clock times mark the termination of decisions arising from the accumulation of noisy evidence until it reaches a left or right terminating bound. Clearly, it is not the case that we could have predicted the subjects’ choices as well as we did using any arbitrary but systematic clock settings. Put another way, had the clock settings represented some post hoc assessment of difficulty, they would have had to conform coincidentally to the functional form of decision terminations that just so happened to predict the choice proportions. These considerations bear on the main alternatives to our hypothesis, considered below.

**Figure 4:**
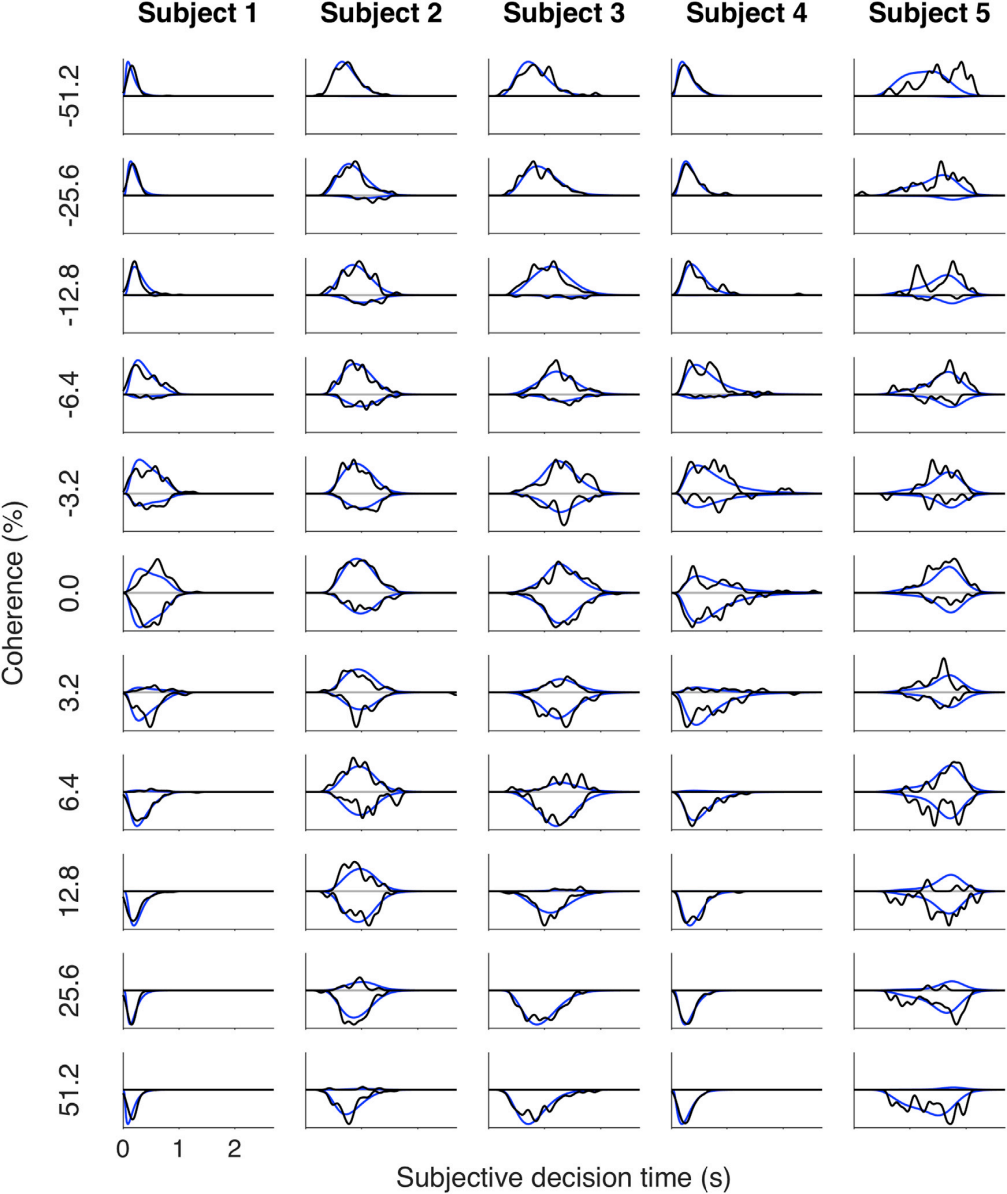
Distribution of *t*_*SD*_s Each row represents a coherence level, each column a subject. Ordinate is proportion of responses with the *t*_*SD*_s from the controlled-duration experiment, signed by the direction of response (positive, right; negative, left). The scale of each row is normalized to fit the row’s height for visualization. Black lines are the data, and blue lines are the drift diffusion fit with collapsing bounds (parameters in Table S3). The data are smoothed in time with a Gaussian kernel (σ = 0.05 s) for visualization. Goodness of fit, quantified by the Jensen-Shannon divergence, is displayed in Figure S4. The more elaborate model also accounts for the reaction time distributions (and slow errors) in the free-response task (data not shown). See also Figure S3.

Another feature of the data supports the interpretation that the *t*_*SD*_s mark the termination of a decision process. In actual reaction time studies, in which subjects respond as soon as ready with an answer (unlike our experiment 1), it has been shown that the full distribution of response latencies across trials is explained by a more elaborate model of bounded evidence accumulation—in particular, one in which the flat bounds are replaced by time-dependent, collapsing bounds [22, 23]. We used such a model to fit the *t*_*SD*_ reports from the controlled-duration task (Figure S3). As shown in Figure 4, the conformance to data is impressive for subjects 1–4 but less so for subject 5. To quantify the goodness of fit, we calculated the Jensen-Shannon divergence (JSD) between the fitted and observed distributions for each of the five subjects (Figure S4). Random shuffling of the fitted and observed distributions across motion strengths supports rejection of the null hypothesis that the quality of the fit would arise by chance, knowing only choice proportions and the mean *t*_*SD*_ values (p < 0.02, subjects 1–4; p > 0.9, subject 5). We used a bootstrap procedure to obtain confidence intervals on the JSD (error bars; Figure S4), which, not surprisingly, identifies subject 5 as an outlier. The ability to explain the distribution of *t*_*SD*_s makes it all the more unlikely that the clock settings from subjects 1–4 represent anything other than termination of an accumulation of noisy evidence.

**Figure 5:**
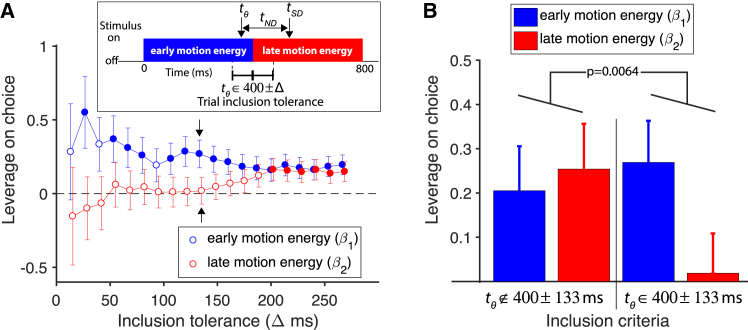
Effect of Trial-to-Trial Variation in the Noisy Motion Information on Choice The analysis compares the leverage of information before and after a putative threshold crossing that terminates integration. Leverage is based on logistic regression of motion energy (right minus left; see STAR Methods), controlling for motion strength. (A) Leverage of early- and late-motion energy (blue and red, respectively). The estimates are shown as a function of the inclusion window. Pairs of points include only trials with estimated termination times (*t*_θ_) near 400 ms. (Inset shows how the putative termination time *t*_θ_ is acquired on each trial. It is the *t*_*SD*_ from the clock setting minus the non-decision time estimated from the fits in Figure 2, top row). Filled symbols designate non-zero leverage (p < 0.05). Larger tolerances permit inclusion of more trials but blur the distinction between pre- and post-*t*_θ_. Error bars indicate SE. (B) Difference in the leverage of motion energy before and after 400 ms requires *t*_θ_ to be near 400 ms. The bars on the right side show the leverage values using tolerance of ±133 ms (same value and SE as the points in A marked by arrows). Bars on the left show the average leverages obtained by sampling the complementary trials with *t*_θ_ outside this tolerance window (5,000 bootstraps of sets of 873 trials; error bars are average of the SE from the bootstraps). The distribution of differences (β_1_–β_2_) rarely exceeds the observed difference (p < 0.007). Combined data from subjects 1–4 using motions strengths 0%, ±3.2%, and ±6.4% coherence.

Finally, we analyzed the motion information in the RDM itself to test whether the subjective decision times demarcate completion of the decisions. According to our hypothesis, motion information in the display should support the choice only up to the time that the accumulated noisy evidence reaches a bound. On each trial, we estimated this time (*t*_θ_) from the clock report minus the non-decision time obtained from the fits in Figure 2 using the three weakest motion strengths and asked whether the information before or after *t*_θ_ was the more informative about the subsequent choice. To place these motion energy comparisons on equal footing, we always used the first and last half (400 ms each) of the display (Figure 5A, inset). Importantly, we restricted the analysis to trials in which *t*_θ_ was close to this midpoint. To increase the power of the analysis, we examined a range of tolerances on *t*_θ_ by requiring it to be within ±Δ ms of the midpoint, thereby varying the number of trials that met the criterion. Figure 5A compares the leverage of the integrated motion energy before and after the midpoint on choice, controlling for motion strength (logistic regression; see STAR Methods). The leftmost red and blue points contain data from the 82 trials in which *t*_θ_ was within 400 ± 13.3 ms from motion onset (i.e., video frames 30 and 31). By widening the acceptance window to 400 ± 26.6 ms (170 trials), we achieve greater power and infer the greatest leverage of the motion information before the midpoint. The graph shows that widening the tolerance (so that eventually all trials are included) leads to a gradual dissipation of the leverage of the motion energy before the midpoint and an increase in the leverage of information after 400 ms. At larger tolerance, the before/after designation no longer matters.

Because we always used the first and second halves of the display to perform these calculations, a possible concern is that the division at 400 ms merely reflects the fact that early stimulus information is more influential than late and says little about whether the *t*_θ_ are informative. To evaluate this, we performed a bootstrap analysis (n = 5,000), in which we compared the leverage of the pre- and post-400-ms motion energy on trials with a *t*_θ_ within ±133 ms window of the midpoint with those with *t*_θ_ outside this window (Figure 5B). We chose this window to balance power against dissipation of the effect (see STAR Methods). We evaluated the null hypothesis that the difference in leverage for the pre- and post-400-ms motion energy was the same for these two sets of trials. This showed that the pre-400-ms motion energy had significantly more leverage than the post-400-ms motion energy on choice only when *t*_θ_ was within the ±133 ms window (p < 0.007; one-tailed). This held for many windows from 26 to 160 ms (six p < 0.05 and five p < 0.1).

### Experiment 2: Free Response with Subjective Decision Times

According to our hypothesis, *t*_*SD*_s mark the termination of the same type of process that gives rise to reaction times, when subjects control their viewing time. We therefore collected data on a free-response version of the task from the same subjects after they completed the first experiment. All aspects of the task, including the clock, were the same as the version above, except that, instead of waiting for the random dots to disappear after a preset duration, the participant reported each decision as soon as she was ready by moving the stylus to one of the choice targets and subsequently set the clock to report a subjective decision time. The *t*_*SD*_s in this experiment are not particularly illuminating, because they might be coupled to reaction times even if they did not indicate decision termination. However, they do serve as a sanity check, and in this regard, it is reassuring that the *t*_*SD*_s were indeed correlated with RTs (Pearson's *r* = 0.80–0.96; p < 10^−10^), consistent with previous studies [24, 25, 26]. For subjects 1–4, most *t*_*SD*_s preceded the RTs (range 52%–98%). For subject 5, only 15% of the *t*_*SD*_s preceded the RTs, consistent with the observation that this participant deployed the clock settings differently than the others. Presumably, the decision to report and the decision to move are not the same process, just as the decision to report with a saccade or a reach movement is subserved by different circuits [27, 28]. Therefore, in principle, the decision to report could occur after the decision to move the stylus, using information from the random dot display that did not arrive in time to affect the hand movement [29, 30, 31]. Indeed, in almost all of the trials in which the RT preceded *t*_*SD*_s, the difference was less than 400 ms (99.5% of all trials and 97% of trials in which the RT preceded *t*_*SD*_s, across subjects 1–4).

**Figure 6:**
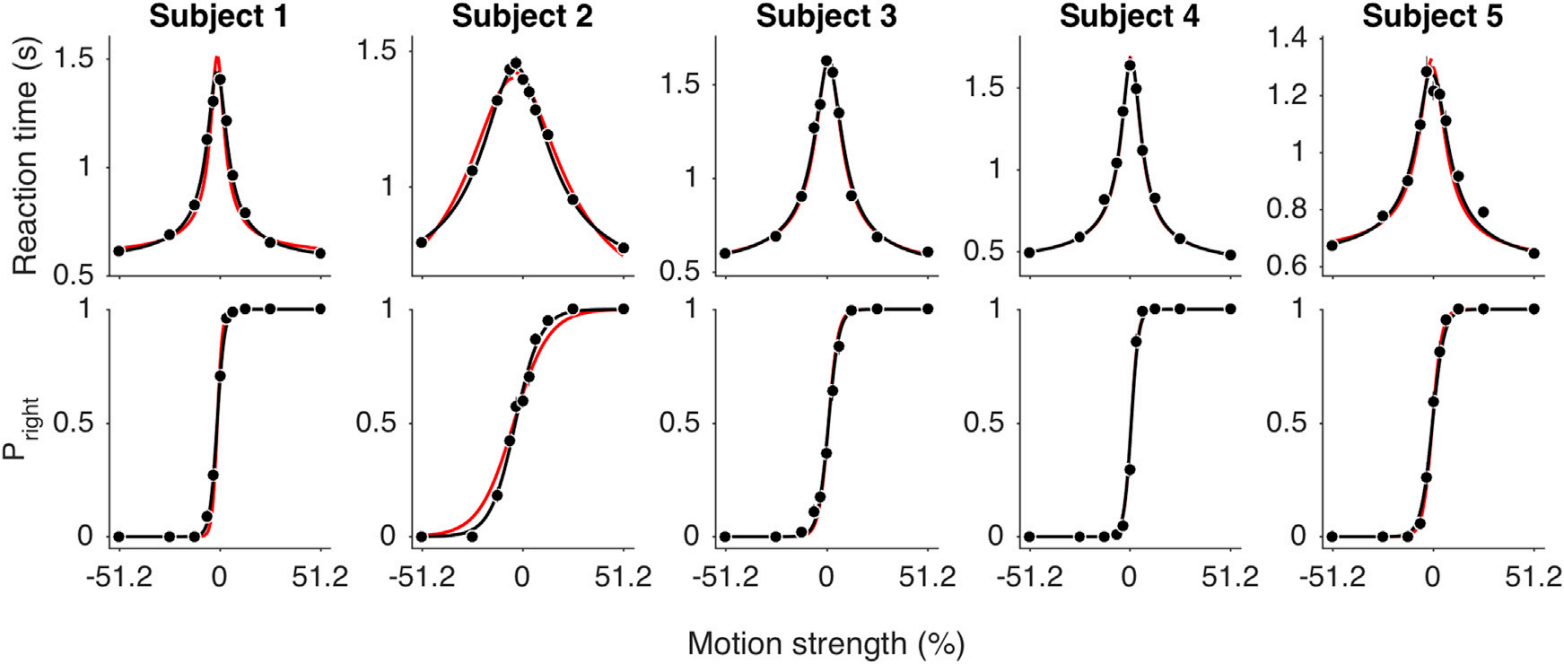
Reaction Times and Choices in the Free-Response Task Reaction times (top) and proportion of rightward choices (bottom) are plotted as a function of motion strength for the five subjects (mean ± SEM). Solid lines are drift-diffusion fits to RT and choice data (black; parameters in Table S1). Red curves show fits with *κ* fixed to the parameters from the *t*_*SD*_ fits for each subject in the controlled-duration task (parameters in Table S2). See also Figure S5.

The choice and reaction time data from all five subjects were well described by bounded drift diffusion (Figure 6). The black curves are fits of the parsimonious model, used above, to the choice-reaction time data from the free-response task. They are joint fits to both sets of observations—choice and RT—rather than predictions (parameters in Table S1). The fitted bound height was higher in the free-response task, but this is not surprising because subjects could avail themselves of up to 2.7 s of evidence in this task, compared to a maximum of 800 ms in the controlled-duration task. In contrast, we reasoned that the parameter, *κ*, should be similar in the two experiments, because it represents the conversion of motion strength to the signal-to-noise ratio of momentary evidence, which tends to be stable when humans and monkeys alter their speed-accuracy tradeoff or their bias [10, 21]. As shown in Figure S5, the scaling parameters, *κ*, estimated from the two tasks were similar for the subjects (Pearson’s *r* = 0.97, p = 0.007 for subjects 1–5; *r* = 0.97, p = 0.032 with subject 5 removed). Indeed, the red curves in Figure 6 for subjects 1–4 are fits that constrain *κ* to the value derived from the *t*_*SD*_s in the controlled-duration task (Table S2; for subject 5, we used *κ* from the joint fit to choice-*t*_*SD*_ data). The similarity to the black curves implies that deducing the signal-to-noise parameter solely from subjective reports of the time of decision completion in the controlled-duration task predicted a key parameter of the mechanism that would give rise to decisions in a later experiment.

These observations (Figures 6 and S5) lend further support to our hypothesis that a common mechanism supports decision times reported explicitly or via mental chronometry. They also underscore the counterexample of subject 5, because the consistency of *κ* demonstrates that he used a similar mechanism of evidence accumulation to make decisions on the controlled-duration and free-response tasks, yet the *t*_*SD*_s obtained in the controlled-duration task were uninformative.

### Alternative Hypotheses

We considered several alternative explanations of the subjective reports, which would imply that they are not signatures of decision termination. One possibility is that the *t*_*SD*_s do not represent a termination at all because the subjects used all of the information in the motion display to form their decisions. This alternative cannot explain the capacity to use the *t*_*SD*_s to predict the choice frequencies (Figure 2), and it is directly refuted by the analyses of motion energy in Figure 5, which shows that subjects ignore late-arriving information when the inferred termination times are near the midpoint of the trial.

Another class of alternatives would allow for early termination of evidence accumulation but posit that such events are not reflected in the *t*_*SD*_s. For example, the reports might be assigned, inferentially or postdictively, to a moment between the start and end of the dot motion [32, 33, 34]. The subject could believe in the experience of completing the decision, but it would have no correspondence to the actual time of commitment [35]. This is an intriguing idea, but it fails to account for the dependency of *t*_*SD*_s on motion strength. Any reasonable alternative must account for this regularity. For example, the subjects might have used the clock as a rating scale for difficulty, setting the clock nearer the starting position when the stimulus appeared more coherent (or felt easier). There are three reasons to reject this alternative: (1) monotonic transformations of difficulty are generally incapable of achieving choice predictions unless they happen to be nearly identical to *t*_*SD*_s (Figures 3 and S2); (2) the idea provides no explanation for the distribution of *t*_*SD*_s across trials (Figure 4), and (3) it fails to explain the difference in leverage of the early and late motion information on choice—specifically, the sensitivity of this analysis to the use of the corresponding trial’s *t*_*SD*_ (Figure 5). These considerations rule out most “clock as rating scale” alternatives, but there is one that remains.

It has been shown that elapsed decision time bears on confidence that a decision is correct [36], and for some observers, elapsed time is more important than motion strength. It might therefore be argued that, despite the instruction to indicate the time of a decision, subjects used the clock as a rating scale, placing the hand closer to its initial position if they were more confident. However, this possibility is incompatible with observations on the subset of trials in which motion was displayed for only 200 ms. Not surprisingly, subjects were less sensitive to the motion on these trials compared to trials with motion displayed for 800 ms (p < 0.002 for all except subject 2, whose trend was of the same sign: p = 0.32; see Figure S6), yet the *t*_*SD*_s were shorter (Δduration range: −401 ms to −64 ms; p < 10^−6^). If the clock settings were a report of confidence, they should have exhibited the opposite trend. We conclude that four of the five subjects reported *t*_*SD*_s that were linked to the time of decision termination, as they were instructed.

## Discussion

Our findings exploit a well-studied task that has been used to expose the neural mechanisms of a perceptual decision. Although the decision is about a perceptual quality or category, the task is less a model of perception, which is typically fast [37, 38], and more like the kind of deliberative decisions we make over more prolonged intervals based on a sequence of samples of evidence [39, 40]. Its main advantages are the quantitative agreement with the mathematical depiction of bounded evidence accumulation and the correspondence with neural recordings, mainly from rhesus monkeys (e.g., [8, 41]). Thus, it furnishes an empirical test of the hypothesis that the feeling of “having decided” corresponds to a threshold crossing that marks the moment that the accumulated evidence reaches a terminating bound.

In a free-response experiment, there is an overt manifestation of decision termination, namely the reaction time, whereas, in our controlled-duration experiment, there is only the memory of the time of the feeling of having decided—that is, the clock settings (*t*_*SD*_s). Without another indication of when the decision terminated, it might seem impossible to associate *t*_*SD*_s with termination of the decision [18]. However, we reasoned that we could overcome this limitation by using the *t*_*SD*_s to account for the one other experimental observation: the choices. We fit the *t*_*SD*_s to a bounded evidence accumulation model to derive three parameters (*κ*, *B*, and *C*_*0*_) that would fully specify the choice frequencies as a function of motion strength. The success of these predictions was remarkable in four of the five subjects. Thus, we conclude that the time that these subjects reported that they had decided does indeed mark the time of decision termination. It is not the actual time but offset by a constant, analogous to the motor preparatory component of the reaction times in a free-response experiment (see below).

Had the subjects indicated their choices at the time they made them, we would suspect that their clock settings merely indicated the time of the action—that is, a post hoc report of another process, such as movement of the stylus. In that case, we could not argue that they mark a subjective awareness of decision termination. This is a reasonable interpretation of the *t*_*SD*_s in the free-response task, but not in the controlled-duration task, in which subjects only indicated their response after a variable delay period. There was no event in the experiment or the subject’s behavior that could be assigned a time on the clock except for the one that the subjects were asked to note—the feeling that they had made up their mind.

One might argue that the subjects were performing a mock free-response task in their mind and reporting *t*_*SD*_s at the time of their planned action. This is unlikely because four of the subjects had never experienced a reaction time experiment and we did not introduce the free-response task until after data collection was completed on the controlled viewing duration task. There is, of course, a sense in which this proposal is consistent with our interpretation. We have argued forcefully that the subjective decision times mark a mental event that is mediated by a threshold on the evidence accumulation—that is, the same type of process that underlies decision termination in a free-response (reaction time) experiment. Finally, we cannot rule out the possibility that subjects changed their mind (e.g., see [29, 42]), but that is likely to have involved only a small fraction of trials.

There are several important implications of our result. First, it confirms that the brain exercises a stopping criterion on a stream of evidence, even when the environment (or experimenter) controls the duration of the stream of evidence [16, 17]. Put another way, even when there is no overt measure of reaction time, decision makers terminate their decisions in a manner similar to the way they would if they were responding when ready. It suggests that accuracy in simple two-alternative, forced-choice experiments is not determined solely by considerations of signal to noise, as commonly assumed, but the speed-accuracy policy adopted by the subject. Without a measure of RT, the speed of a decision is not known, but it is conceivable that variation in accuracy among individuals and during learning reflects differences in the speed-accuracy trade-off, even when there is no outward manifestation of decision time. The present study shows that this time may be consciously registered as an “aha” moment, available to the decision maker if asked (or knew she would be asked).

A second implication concerns mental chronometry itself—that is, the validity of the clock report. It has been argued that such reports may be too unreliable or too biased to mark actual mental events [26, 43, 44]. Further, without an objective measure of the mental event, it seemed impossible to validate that the thing being timed had actually occurred at that time or at some lawful latency. The present findings support the validity of the clock reports. No doubt they are imprecise, but the conformance to actual RT distributions suggests that some of the imprecision is a reflection of the variability of mental events themselves. We provide an example in which they are tied to a mental event that has no external manifestation and thus seems to be untestable, just as in the Libet experiments. However, we showed that the event had predictive power and that it corresponds to the type of termination events that lead to reaction times in other experiments, including the free-response experiment with our subjects. We cannot argue that mental chronometry is valid in other settings, such as the Libet experiments. However, to the extent that an urge to move (as in Libet) is effectively a decision to move, we are inclined to think so.

Like actual RTs, the time of the report—be it a movement or a mental note of the position of the clock—differs from the time of the decision. This discrepancy, termed the non-decision time (*t*_*ND*_) in RT experiments, is typically 300–400 ms for motion discrimination, depending on the response modality. Electrophysiology in the monkey suggests that ∼200 ms of this *t*_*ND*_ is in the time it takes information in the video display to impact the representation of the accumulated evidence [19]. The rest of the *t*_*ND*_ is accounted for by a latency between the termination of the accumulation and initiation of the motor response. For mental chronometry, the *t*_*ND*_ might comprise systematic biases in the perception of the clock position or the recall of the position or both, as argued by skeptics of Libet’s use and interpretations (e.g., [43, 45]). This does not negate the validity of *t*_*ND*_, however, as it allows us to infer the decision time (*t*_θ_) from the subjective report to predict not only the choices but an approximate point in time that divides the stimulus into information used and ignored on the trial (Figure 5).

The intriguing insight furnished by the present study is that the moment of subjective awareness of having decided reflects the termination of a decision process. Here, there is substantial evidence that decision termination is mediated by application of a threshold to the neural representation of accumulating evidence [8, 46, 47], and this also holds in experiments that do not allow for free responses, as in our controlled-duration experiment [13, 16, 48]. This operation may be more widespread, as many mental processes achieve a state of completion, which is effectively a decision point. Of course, not all involve a choice among discrete propositions, like the motion discrimination task; nor are all as simple as a decision to commence a movement, as in Libet’s studies. Some might involve a transition or branching from one step of reasoning to another in more complex problems involving strategy (e.g., foraging and medical diagnosis). The common feature is a satisfaction of some termination criterion before proceeding. Such termination events do not result invariably in conscious awareness, but the subjective decision times assessed here (and by Libet) do necessitate conscious awareness, by definition. Indeed, the main distinction between non-conscious and conscious decisions might be simply the possibility of reporting in some way, even if only provisionally [5]. Thus, we suggest that the neurophysiological process responsible for completing the decision to report is also responsible for piercing conscious awareness. This assertion may not satisfy philosophers who postulate a distinction between what is reportable and what is in conscious awareness (e.g., access and phenomenal consciousness [49]). These philosophers might think it is possible for consciousness awareness to lag behind the decision to report. There may be some room in the non-decision time to countenance this argument, but it is narrow.

The capacity to report is the criterion we use informally to query whether an agent is consciously aware of something, but it has a deeper significance. We speculate that the decision to report—even if only provisionally—is the common element connecting those mental processes that pierce conscious awareness. Consider that non-conscious knowledge of objects (e.g., position, shape, and desirability) corresponds to affordances [50], a term that refers to ways of interacting with the object (e.g., position for looking, shape for grasping, and desirability for eating, mating, or fleeing). These provisional affordances confer properties that are as much about our possible actions as they are about the object. The possibility of reporting a feature of an object to another agent—or to oneself in the future (e.g., using episodic memory)—changes the balance away from my possible actions and toward the object, which inhabits not just the personal space of my own actions but also the mental space of another’s mind [51]. For example, the location of the object transcends my personal frame of reference, and the object itself seems to possess qualities that are independent of my actions—an essence, as it were [52]. These philosophical speculations concern the content of conscious experience (e.g., what it is that we might report), whereas the *t*_*SD*_s in our experiment merely mark the time that the subject decided to possibly render it. Of course, many decisions arise without triggering a decision to report, and these remain unconscious to us.

Importantly, we do not claim that our subjects were not conscious of the deliberation process itself. They might have been consciously aware of some or all of the random dot motion leading to the decision. There are two ways to account for this within our framework. First, the visual system might analyze other features of the random dot display and reach provisional decisions to report (e.g., a cluster of dots resembling a geometric shape). Second, during deliberation, one might reach a decision by applying a lower threshold but then change one’s mind [29, 30] or reaffirm by applying a more conservative threshold. Each of these mini-decisions might pierce consciousness. Second, when a process pierces consciousness, it carries with it content and associations that are there to be reported as well. Some of this content could be in visual working memory (e.g., appearance of some pattern in the dots) or working memory of the experience of deliberating [53], what is sometimes referred to as metacognition [54]. These explanations are not mutually exclusive. In the first, there are many piercings. In the second, there could be only one, with content from the past. Our experimental findings do not address the second idea, as we only asked participants to report left and right and the moment that they “felt they had decided in their mind” (see STAR Methods). Rather, they suggest that the piercing of conscious awareness might be mediated by a process resembling the termination of simpler decisions with overt manifestations of completion (e.g., reaction time). This raises the intriguing possibility that consciousness itself may be closer to a neuroscientific explanation than is commonly thought, as knowledge of the neurobiology of decision making is rapidly advancing.

## STAR★Methods

### Key Resources Table

**Table:** 

REAGENT or RESOURCE	SOURCE	IDENTIFIER
**Deposited Data**		

Raw and analyzed data	This paper	https://github.com/yulkang/SubjDecTime.git

**Software and Algorithms**		

Classical Statistics	MATLAB	https://www.mathworks.com/products/matlab.html
Evidence Accumulation Models	This paper	https://github.com/yulkang/SubjDecTime.git
Sensitivity Analysis of choice predictions	This paper	https://github.com/yulkang/SubjDecTime.git
Motion Energy Analysis	This paper	https://github.com/yulkang/SubjDecTime.git

### Contact for Reagent and Resource Sharing

Further information and requests for resources should be directed to and will be fulfilled by the Lead Contact, Michael Shadlen (shadlen@columbia.edu).

### Experimental Model and Subject Details

Five human participants (3 male and 2 female aged 25–38) provided written informed consent and took part in the study. All participants were naive about the hypotheses of the experiment. No gender specific analyses were performed, owing to sample size. All participants had normal or corrected-to-normal vision. The study was approved by the local ethics committee (Institutional Review Board of Columbia University Medical Center).

### Method Details

#### Apparatus

Participants sat in a semi-dark booth in front of a monitor (Vision Master 1451; 1400 × 1050 resolution, 75 Hz refresh rate). A headrest and chinrest ensured a viewing distance of 55 cm. Hand movements were recorded using a hand-held stylus on a tablet surface (Wacom Intuos4 XL, Kazo, Japan; 200 Hz, resolution 0.005 mm). The position of the stylus was mapped onto the stimulus screen and indicated by a small green cursor.

#### Tasks

Participants discriminated the net direction (left or right) of stochastic random dot motion [11, 55]. The dots were displayed for one frame (13.3 ms), and four frames later a subset of these dots were displaced in the direction of motion while the rest of the dots were displaced randomly. Thus dots in frame five might contain displaced dots from frame 1; same for frames 6 and 2, and so forth. The dot density was 16.7 dots deg^-2^ s^-1^ and displacements were consistent with a motion speed of 1.25° s^-1^ (2.64 pixels per 53.3 ms). The difficulty of the task was manipulated through the coherence of the stimulus, defined as the probability that each dot would be displaced as opposed to randomly replaced (online MATLAB code for the motion stimulus [56]:). Motion direction to the left or right (indicated by the sign of *C*) occurred with equal probability. The motion strengths (|*C*|) were sampled uniformly from 6 different coherence levels (0, 3.2, 6.4, 12.8, 25.6, and 51.2%). On the 0% coherence trials, the direction deemed correct was assigned randomly.

The dots were restricted to an annulus defined by invisible concentric circles with diameters 1° and 5° at the center of the screen. A timing device, termed a clock (1° diameter), after Libet [1, 2, 3], was centered at the fixation point and surrounded by the random dots. Based on extensive piloting, we settled on this geometry because it facilitated simultaneous processing of the random dot motion while tracking the clock (i.e., minimized interference). The clock had a hand and a small tick mark that indicated the position of the clock hand at the time of the motion onset. The initial position of the clock hand was random (uniform distribution on circle). The clock hand period was 2.7 s (2.3 rad/s), which is 1.7 times the longest motion stimulus plus longest delay.

Participants initiated a trial by moving the stylus on the tablet to place the cursor at the ‘home’ position, indicated by a gray circle (0.3° diameter) at the bottom of the screen, and by fixating a central red circle (0.1° diameter). Two choice targets (4° diameter circles) then appeared 5° to the left and right of the fixation point (Figure 1 and Methods S1 and S2) followed by a short delay (0.5 s). The motion stimulus and the clock then appeared simultaneously. In the controlled-duration experiment, the RDM was displayed for a duration drawn from {0.2, 0.4, 0.6, 0.8 s} with corresponding probabilities of {0.125, 0.0625. 0.0625, 0.75}, followed by a random delay (same distribution as the dot motion but sampled independently) during which the clock continued. We oversampled the longest duration of RDM and the delay period because we expected they would permit the largest range of *t*_*SD*_. We also sampled the short duration to encourage subjects to utilize the stimulus stream from the onset. The intermediate durations (0.4 and 0.6 s) were included to discourage subjects from using two different strategies for short and long duration trials.

A beep then cued participants to indicate their decision about direction by moving the stylus/cursor to one of the choice targets, which brightened to signal acceptance as the subject maintained central fixation. Participants then reported their subjective decision time (*t*_*SD*_). They were instructed to indicate the “position of the clock hand at the moment you decided—in your mind—whether the motion is to the left or to the right” and to “move the pen until the clock hand marks the position it was in when you made the motion decision” (see Methods S1 and S2). To do this they moved the cursor downward from the choice target to adjust the clock hand until it matched the remembered clock position at the subjective decision time and pressed the center button, among three vertically arranged buttons, with the other hand (Figure 1). Instead of reporting their subjective decision time, participants could also indicate via a button press that they did not remember the position of the clock hand at the time of their decision or they did not make a decision about the motion direction. Based on pilot data, we expected that subjects would utilize the option more often when the stimulus duration was short, but they rarely reported not making a decision or not remembering the position of the clock hand. Subjects were then informed by one of two different sounds whether the motion direction decision was correct or not, leading to the gain or loss of a point respectively, and a visual display of the score, which concluded the trial.

Participants were required to maintain central fixation throughout the trial (window ± 3°; although the absolute position varied between trials, the eye positions were within 1.0° of the average position of each trial in 95% of the trials except for subject 5 in the controlled-duration experiment, whose eye positions were within 1.7°). Eye position was monitored at 1 kHz using an Eyelink 1000 (SR Research Ltd., Mississauga, Ontario, Canada) to ensure fixation during stimulus viewing.

After data collection was completed on the controlled-duration task, participants moved to the free-response task. This task was identical to the controlled-duration task, except that the subject could terminate the trial at any time during motion viewing, by moving the cursor to the choice target. Participants were instructed to report their decision “as soon as you are ready with an answer.” The RDM stimulus was extinguished once the cursor crossed the boundary of the home position. This event also marked the RT measured from the onset of RDM. As in the controlled-duration task, participants then indicated their subjective decision time (*t*_*SD*_) by reporting the position of the clock hand at the time they felt they had made the decision about the motion direction. To do this they moved the cursor downward from the choice target to adjust the clock hand until it matched the remembered clock position at the subjective decision time (Figure 1). Instead of reporting their subjective decision time, participants could also indicate via button presses that they did not remember the position of the clock hand at the time of their decision. There was no option to indicate that they did not make a decision about the motion direction, since the stimulus was on until they made a decision. Subjects received the same auditory and visual feedback on the accuracy of their choice as in the controlled-duration task.

Each participant performed 870–2030 trials of the controlled-duration task and 1000-2100 trials of the free-response task. Participants completed all sessions with the controlled-duration task before they were instructed and tested on the free-response task. When explaining the free-response task to the subjects, all but one (subject 4) stated that they had not performed this kind of task before.

#### Training

All subjects received extensive training prior to the experiment over a number of days. The training was carefully scripted. For each training session and task, participants viewed instructions in a PowerPoint presentation accompanied with video demonstrations of the task (see Methods S1 and S2). They then had to correctly answer a set of task-related questions before proceeding to the experiment. They were also allowed to review the instruction as needed. This proceeded as follows:

(1) Controlled-duration random dot motion task without a clock or reporting of subjective decision time. This proceeded in several steps with sets of progressively lower coherences and shorter durations of the motion stimulus: a) 76.8% only, and 800ms only; b) 76.8% only, and the same duration distribution as in the controlled-duration experiment from here on; c) 51.2% only; d) 12.8, 25.6, and 51.2%; e) 3.2, 6.4, 12.8, 25.6, and 51.2%; f) 0, 3.2, 6.4, 12.8, 25.6, and 51.2%, as in the controlled-duration experiment. Participants were required to achieve accuracy > 90% correct on the strongest motion strength and successfully follow the instructions in 80% of the trials (90% for step f) before proceeding to the next step.
(2) Clock training and validation. Participants performed controlled-duration trials during which a beep (3 kHz, 20 ms) occurred at a random time (uniform 0–2.6 s, in steps of 25 ms). Participants were required to report the clock hand position at the time they heard the beep. They received auditory feedback of success if the estimate was within ± 200ms of the true time. The training proceeded in several stages: a) Indicating the beep timing without the motion stimulus; b) Indicating beep timing while ignoring the motion stimulus; c) Indicating beep timing while ignoring the motion stimulus and the ‘go’ beep that has a different pitch (1 kHz, 50ms). Sessions were repeated until subjects reached 80% accuracy in beep timing report and successfully followed the instructions in 80% of the trials.

Subjects completed data collection on the controlled-duration experiment before they began training on the free-response version of the task.

(3) Free-response (reaction time) random dot motion task with reporting of subjective decision time. After subjects completed the controlled-duration experiment, they received instruction for the free-response task and went on to perform the task.

In total subjects performed between 12 and 23 sessions of training and experiment, spanning an average of 55 days.

#### Computational Modeling

We fit the data using two drift-diffusion models. We used both a simple (“parsimonious”) model with flat bounds as well as a more elaborate model in which the bounds collapse over time. The simple model is adequate to fit the mean decision times (hence RTs and *t*_*SD*_) for correct trials only—that is, trials in which the decision favors the direction supported by the sign of the coherence, taking into account any bias (as explained below). The fits supply four free parameters, three of which can be used to predict the choice functions (Figure 2). The more elaborate model explains *t*_*SD*_ on error trials and accounts for the *t*_*SD*_ distributions, but it must be fit to the *t*_*SD*_ conditioned on correct/incorrect trials. Thus it fits—as opposed to predicts—the choice function. We exploited the prediction of the parsimonious model as a stringent test of the hypothesis that the measured *t*_*SD*_ were indicative of the termination of a bounded evidence accumulation process.

#### Evidence Accumulation Model with Flat Bounds

We used a variant of the drift-diffusion model [7, 20] to fit the *t*_*SD*_ from the controlled-duration experiment and used the parameters of these fits to predict the choice frequencies. The model posits that evidence accumulates from zero until it reaches an upper or lower bound, ±*B*), which determines the initial choice and decision time. The increments of momentary evidence are idealized as Gaussian distributed random variables with unit variance per second and mean *κ(C*-*C*_*0*_*)*, where *C* is signed motion strength (specified as the proportion of dots moving in the net motion direction: positive for rightward, negative for leftward); *κ*, *B* and *C*_*0*_ are free parameters. The expectation of the momentary evidence is also termed the drift rate. Intuitively, *B* is the square root of the mean decision time when the drift rate equals zero, and *κB* controls the sensitivity (i.e., accuracy as a function of *C*); *C*_*0*_ is a coherence offset, which explains the bias (if any) for one of the choices. The model predicts the probability of terminating at ± *B*, hence the proportion of rightward choices as function of signed coherence,

(Equation 1) $$P_{right}\left(C\right)={\left[1+\exp\left(-2\kappa \left(C-C_{0}\right)B\right)\right]}^{-1},$$

and the mean decision time, which differs from the reported *t*_*SD*_ by an additional fixed latency, termed the non-decision time (*t*_*ND*_),

(Equation 2) $$t_{SD}\left(C\right)=\frac{B}{\kappa \left(C-C_{0}\right)}\tanh\left(\kappa \left(C-C_{0}\right)B\right)+t_{ND}.$$

The parsimonious model is only capable of explaining the mean decision times when the choice is in the same direction as the drift rate. Absent bias, these would be rightward choices for positive coherences, leftward choices for negative coherences, and all choices for 0% coherence. In general these are the directions of the more numerous choices at each coherence, including 0. In practice, we identified the trials for analysis of the *t*_*SD*_ by finding the point of subjective equality from a simple logistic fit to choice and selecting rightward choice trials when *P*_*righ*t_ > 0.5 and leftward choice trials for *P*_*righ*t_ < 0.5. We did not use the logistic to estimate the parameter, *C*_*0*_.

To fit Equation 2 to the mean *t*_*SD*_, we maximized the log likelihood, assuming Gaussian noise with standard deviation given by the standard errors of the means in the data (error bars, Figure 2, top). We optimized using MATLAB’s *fmincon* using analytic gradients. We derived analytic Hessians to obtain standard errors on parameters (Table 1).

We then asked whether the fitted parameters (*κ*, *B* & *C*_*0*_) could predict the choice proportions for each of the five subjects (Equation 1; blue dashed curves, Figure 2, lower). To examine the significance of the prediction, we generated 400 datasets with *t*_*SD*_ shuffled across coherences, fitted parameters to each dataset and computed the log likelihood of the prediction of the choice. We computed the p value as the proportion of log likelihoods of the datasets (shuffled and original, total 401 datasets; see [57, 58] for rationale) that are not lower than that of the original dataset.

We also used the simple drift-diffusion model to fit the RT data in the free-response task. Here we allowed different *t*_*ND*_ for left and right decisions to account for potentially different motor latencies. We fit the RT and choice functions jointly by maximizing the contribution to the likelihood from the mean RTs (Gaussian error) and the choices (binomial error, see [20]). We used the same approach to render the gray curves in Figure 2 (subject 5).

#### Model with Collapsing Bounds

For the model with collapsing bounds, instead of stationary bounds, we implemented two time-dependent absorbing boundaries ± *A(t)*. The bounds collapse toward zero with dynamics parameterized using the regularized incomplete beta function (I):

(Equation 3) $$A\left(t\right)=A_{0}\left(1-I_{t^{\prime }}\left(\beta_{1},\beta_{2}\right)\right),$$

where *t*′ is normalized time (i.e., *t*/2.7) so that the collapse is complete by the maximum time allowed from the random dot stimulus onset until leaving the home position (2.7 s). We used the incomplete beta distribution for simplicity and flexibility; a variety of alternatives suffice as well [59, 60]. Rather than representing the collapsing bound by the beta distribution parameters directly we represented the shape of the collapsing bound by two constructed parameters: *B*_*log*_ = log_10_(*β*_1_*β*_2_) which correlates with the slope of the collapse and *t*_*β*_ = *β*_1_/(*β*_1_+*β*_2_) which is around the time of the steepest descent in the normalized time (1 corresponds to 2.7 s).

As we wished to evaluate the likelihood of individual *t*_*SD*_ we modeled the non-decision time with a gamma distribution (parameterized by mean μ and standard deviation σ) which ensures that non-decision time was always positive. We optimized the parameters of the model (*κ, C*_*0*_, *A*_*0*_, *B*_*log*_, *t*_*β*_, μ, σ) to maximize the likelihood of the observed *t*_*SD*_ and the choices using MATLAB’s fmincon function. We fit three sets of measurements for each subject: the *t*_*SD*_ from the controlled-duration, *t*_*SD*_ from the free-response, and also the RTs from the free-response (in which we allowed separate *t*_*ND*_ distributions for left and right decisions to account for potentially different motor latencies).

To estimate the standard error we sampled from the posterior distribution of the parameters using Metropolis sampling. We initialized 12 chains in the neighborhood of the mode found from the gradient descent procedure and sampled 5000 times after burn-in of 5000 samples. We used the multivariate normal distribution as the proposal distribution, and adapted its covariance every 100 trials using up to 1000 previous samples’ covariance during burn-in [61, 62]. Every parameter converged, as determined by the ratio of within- and between-chain variance $\left(\hat{R}<1.1\right)$ as previously described [63]. The standard error was taken as the standard deviation of the Monte Carlo samples.

To examine whether *t*_*SD*_ differed for correct and incorrect decisions, we used simple linear regression using only motion coherences with at least one error (Figure S3):

(Equation 4) $$t_{SD}=k_{1}+k_{2}\left|C-C_{0}\right|+k_{3}I,$$

where *I* is an indicator variable (0 for correct and 1 for incorrect). We tested the null hypothesis that *k*_*3*_ = 0.

We measured the similarity of the fitted and the observed *t*_*SD*_ distributions (Figure 4) using the Jensen-Shannon divergence (JSD). The JSD is roughly the expectation of the log likelihood of observing one density function at the values of another. It is a symmetrized version of the Kullback-Leibler divergence. The comparisons were made after matching the proportion of errors of the fitted distribution to that of the observed distributions (results were nearly identical when we did not match the proportions). To obtain confidence intervals (error bars, Figure S4) we performed a bootstrap analysis. We resampled the data with replacement, fitted the model to the resampled data and obtained a JSD (200 repetitions). We also evaluated the null hypothesis that the observed goodness of fit (JSD) would be explained solely by the variation in mean *t*_*SD*_ as a function of motion strength. For each subject, we produced shuffled predicted distributions by permuting fitted distributions associated with each motion strength and choice type (left and right). To isolate the divergence arising from the shape of the distribution, we matched the summed probability within each combination of coherence and choice to the observed proportion in the data and shifted the predicted distribution in time to match the mean *t*_*SD*_. We report a one-tailed test, p = (*N*_*0*_ + 1) / (*N* + 1), where *N* = 200, and *N*_*0*_ is the number of JSDs produced by this method that are less than or equal to the original JSD [57, 58].

### Quantification and Statistical Analysis

#### Data Processing and Analysis

For each trial, we recorded the choice (left or right) and the subjective decision time (calculated from the clock position indicated by the participant). In the free-response experiment, we also recorded the reaction time—from motion stimulus onset to when the cursor left the home position.

Trials were discarded if the subject left the home location before the ‘go’ beep (controlled-duration task, 0.2%–1.4% of trials) or entered the target location after the clock made one full revolution (2.7 s from the motion stimulus onset, 0%–0.4% of trials in either task in every subject except for subject 5 in the controlled-duration task, 2.3%), resulting in one lost point.

We also excluded trials in which subjects reported that they did not make a decision (5% of trials in subject 4, no trials in all other subjects) or did not remember the position of the clock hand at the time they made their decision (< 0.1% of trials in all subjects and paradigms except 1.4% and 0.3% of trials in subject 4 in the free-response and controlled-duration tasks, respectively). The first 200 trials of each task were designated as practice trials and were not included in the data analysis.

To characterize the main effect of motion strength (|*C*|) on subjective decision time, we used a generalized linear mixed model (GLM), similar to repeated-measures, 2-way ANCOVA. We tested the main effect of |*C*| on *t*_*SD*_, treating subjects (n = 5) as random effects. Visual inspection of residual plots did not reveal obvious deviations from homoscedasticity or normality. We report the p value from the saturated model using likelihood ratio test. The unsaturated model (no interaction between subject and |*C*|) was inferior to the saturated model (BIC) but also showed a significant main effect of |*C*| in the combined dataset. Statistical analyses were performed in MATLAB (MathWorks). The outcome of the statistical analyses is reported in the Results section.

#### Sensitivity analysis of choice predictions

Our central claim that *t*_*SD*_ reflects termination of an accumulation of noisy evidence to a bound relies on the capacity to fit these times with a parsimonious bounded drift-diffusion model and to use the fit to establish predictions for the choice proportions. These predictions were surprisingly good for four of the five subjects. We wished to assess how sensitive our ability to predict choice is to variations in the parameters of the drift diffusion model and in the values of *t*_*SD*_. We used four complementary strategies to achieve this.

First, we generated 400 datasets with *t*_*SD*_ shuffled across coherences, fitted parameters to each dataset and computed the log likelihood of the prediction of the choice. We computed the p value as the proportion of log likelihoods of the datasets (shuffled and original, total 401 datasets) that are not lower than that of the original dataset. We report these p values in Results for each of the five subjects.

Second, for the four subjects for whom we could predict choice based on their subjective decision times, we compared the quality of these predictions using parameters obtained from the parsimonious model fits to the data from the other three subjects. Naturally, any combination of the three parameters instantiates a drift diffusion model and therefore describes a bell-shaped function of *t*_*SD*_ versus motion strength. We performed a group level analysis in which we examined all possible combinations of the three parameters (*κ*, *B*, *C*_*0*_) from three of the subjects (3^3^ = 27 combinations) and used these to predict a fourth subject’s choice. We combined the log likelihoods of choice across the four subjects in all possible ways (27^4^ = 531,441 combinations). These bootstrap samples were compared to the log-likelihood prediction from each subject’s own parameter fits, summed across the subjects. This allowed us to assess the probability that we could predict the subject’s choices better with parameters fit to *t*_*SD*_ from the other subjects’ parameters than from parameters fit to the subject’s own *t*_*SD*_. We also performed this analysis on subjects 1-4 including the parameters from the fit to subject 5 (the gray curves in Figure 2). Only 3 of 10^5^ (of the possible 10^9^) combinations we tried were better than the combined likelihoods from subjects 1-4.

Third, we assessed a range of transformations of the mean *t*_*SD*_ values consistent with a monotonic increase in *t*_*SD*_ as a function of motion strength. The analysis has two purposes: (i) to determine whether any systematic setting of the clock as a function of motion strength is capable of predicting choice, and (ii) to assess the amount of perturbation required to achieve the choice predictions that would be reliably worse than those based on the data. To do this we fit reflected cumulative beta distributions to *t*_*SD*_ for each subject. We minimized the sum of squared errors between the mean *t*_*SD*_ for each signed coherence,

(Equation 5) $$t_{SD}=k_{1}+k_{2}*\text{cbeta}\left(\left|C-k_{3}\right|/0.512,\alpha ,2-\alpha \right),$$

where cbeta(*x,a,b*) is the integral from 0 to *x* of the beta distribution with parameters *a* and *b*. We fit *k*_*1*_ and *k*_*2*_ which allow us to offset and scale the function and *k*_*3*_ which allows the maximum to be centered at nonzero coherence (analogous to *C*_*0*_ in Equation 2). We varied the parameter of the distributions, α, from 0.01 to 1.95 in 31 steps. Each value of α yields a shape, which we scaled and centered to best match the original data by fitting Equation 5 (least-squares). The *R*^*2*^ value from this fit characterizes the degree of distortion imposed by Equation 5. (Figure 3, top row) shows a selection of fits for seven α parameters equally spaced in the range. For each fit, we generated surrogate clock times (*t*_*surr*_) from the fitted function and then fit these using the parsimonious bounded drift-diffusion model (Equation 2). We compared the likelihood of observing the choices based on *t*_*surr*_ and *t*_*SD*_ which we display as a log likelihood ratio in Figure 3 (bottom). This analysis was performed for subjects 1-4, whose *t*_*SD*_ predicted choice.

Fourth, we examined our ability to predict choice when the *t*_*SD*_ are jittered while preserving their order of *t*_*SD*_ across coherence from the original data (Figure S2). To do this, for each subject we ranked the mean *t*_*SD*_ and calculated the difference between consecutive *t*_*SD*_s. We randomly permuted the order of these differences and regenerated surrogate data (*t*_*surr*_) by accumulating these difference from the shortest *t*_*SD*_. This method ensures that the shortest and longest *t*_*surr*_ are identical to the shortest and longest *t*_*SD*_ as is the rank order with respect to signed motion strength. We repeated this procedure 1000 times for each subject. We fit each set of *t*_*surr*_ with the parsimonious diffusion model (Equation 2) and generated predicted choice functions. We calculated the log-likelihood ratio between these predictions and the fit to the actual data. We also calculated for each jitter, the average absolute difference in times of *t*_*surr*_ compared to the original data: $\Delta \equiv \text{mean}\left(\left|t_{SD,i}-t_{surr,i}\right|\right)$, where *i* represents signed coherence. We tested the null hypothesis that jittering the data would not affect our ability to predict choices. To do this we examined the correlation between log-likelihood ratio and the Δ. As a measure of sensitivity we used linear regression to determine the value of Δ at which the average log-likelihood ratio fell to −10.

Note that the distortions introduced using the latter two strategies (Figures 3 and S2) can lead to the same fitted function as the measured *t*_*SD*,_ but unlike unbiased jitter in the *t*_*SD*_ observations, that is not the expectation. They are thus designed to sample order preserving distortions more efficiently. Similarly a constant offset to the observed decision times would only affect *t*_*ND*_ which does not affect the choice predictions.

#### Analysis of motion energy

We performed a reverse correlation analysis aimed at characterizing the stochastic motion information in each trial that might have affected the direction choice on that trial. We used the three weakest motion strengths for this analysis (|*C*| ≤ 0.064) using the 800 ms duration trials from the controlled duration task (combining data from subjects 1-4; 2766 trials). The sequence of random dots shown on each trial was represented as a 3-dimensional binary array (x, y, t). We applied a 3-dimensional FFT to the first and second half of the movie (frames 1-30 and 31-60) and integrated the amplitude (complex modulus) in appropriate octants (passband 2-8 Hz, 0.5-6 cyc/deg) that represent rightward and leftward motions, which were compared (subtraction) to yield a difference in motion energy in favor of rightward, *E*_*R-L*_. We verified that this quantity depends linearly on signed motion strength, *C*.

According to our hypothesis, the subject may not use all the information in the display to reach a decision. Information from this display should bear on the choice before the accumulation reaches a bound. We estimated the time of this threshold crossing (*t*_θ_) by subtracting the non-decision time, obtained from the fits in Figure 2, from *t*_*SD*_ (i.e., the clock setting) on that trial: *t*_θ_ *= t*_*SD*_-*t*_*ND*_. For the analyses depicted in Figure 5, we always divided the trial into two equal halves, which we refer to as “pre” and “post.” Strictly speaking, these designations refer to *t*_θ_ = 400 ms, but we included a range of *t*_θ_ = 400 ± Δ, in steps of 13.3 ms (i.e., video frames). We adopted this approach because it simplifies the integration in the frequency domain, which invites complications when the comparison is between stimulus epochs of different lengths. Importantly, the only difference in the calculation of the points in Figure 5A are which trials are included. For each value of Δ, we applied a logistic regression (GLM) to obtain estimates of the leverage of motion energy before and after 400 ms on choice:

(Equation 6) $$P_{right}={\left[1+\text{exp}\left\{-\left(\beta_{0}+\beta_{1}E_{R-L}^{pre}+\beta_{2}E_{R-L}^{post}+\beta_{3}C\right)\right\}\right]}^{-1},$$

where *P*_*righ*t_ is the probability of a rightward choice, the β_i_ are fitted coefficients, and *C* is signed motion strength. The points in Figure 5 are the coefficients β_1_ and β_2_ with standard errors.

The bootstrap analysis (Figure 5B) was performed using Δ = 133 ms, a value chosen to be large enough to permit inclusion of many trials but small enough to observe a clear difference in leverage (873 trials; arrows, Figure 5A). We evaluate the probability of observing a difference, β_1_−β_2_, from random sets of 873 trials, sampled with replacement from trials with |*t*_θ_−400| > 133 ms.

We chose to to compute motion energy in the frequency domain [64, 65, 66] instead of the convolution based approach using motion filters defined as functions of space and time. This is partly because the method captures a broad range of possible directional noise, which is not restricted to the passband occupied by the signal dots and, more importantly, because it allowed us to test cutoff times designated by windowing the stimulus with the precision of video frame rate rather than inferring a time point from the filtered motion energy based on a rise and decay of the filter (e.g., [16]). A potential drawback is the need to focus on a restricted range of *t*_θ_, which limits our ability to draw conclusions about the precision of the *t*_θ_ estimates and therefore *t*_*SD*_ and *t*_*ND*_.

### Data and Software Availability

Data and code are available at https://github.com/yulkang/SubjDecTime.git.

## Author Contributions

Conceptualization, M.N.S., F.H.P., and Y.H.R.K.; Methodology, Y.H.R.K., F.H.P., D.M.W., and M.N.S.; Software, Y.H.R.K., F.H.P., D.M.W., and M.N.S.; Formal Analysis, Y.H.R.K. F.H.P., D.M.W., and M.N.S.; Writing – Original Draft, Y.H.R.K. and F.H.P.; Writing – Review & Editing, D.M.W. and M.N.S.; Funding Acquisition, D.M.W. and M.N.S.; Investigation, Y.H.R.K. and F.H.P.; Visualization, Y.H.R.K., F.H.P., D.M.W., and M.N.S.; Resources and Supervision, M.N.S.

## Supplemental Information

Document S1. Figures S1–S6 and Tables S1–S3

Methods S1. Controlled Duration Example Trial

Methods S2. tSD Instructions

Document S2. Article plus Supplemental Information
