# Supplementary material for: Piercing of Consciousness as a Threshold-Crossing Operation
Source: Curr Biol. 2017 Aug 7;27(15):2285–2295.e6. doi: 10.1016/j.cub.2017.06.047 (PMC5558038; doi:10.1016/j.cub.2017.06.047)
Supplement: Document S1. Figures S1–S6 and Tables S1–S3 [file mmc1.pdf]

Current Biology, Volume *27*

Supplemental Information

Piercing of Consciousness as a Threshold-Crossing Operation

Yul H.R. Kang, Frederike H. Petzschner, Daniel M. Wolpert, and Michael N. Shadlen

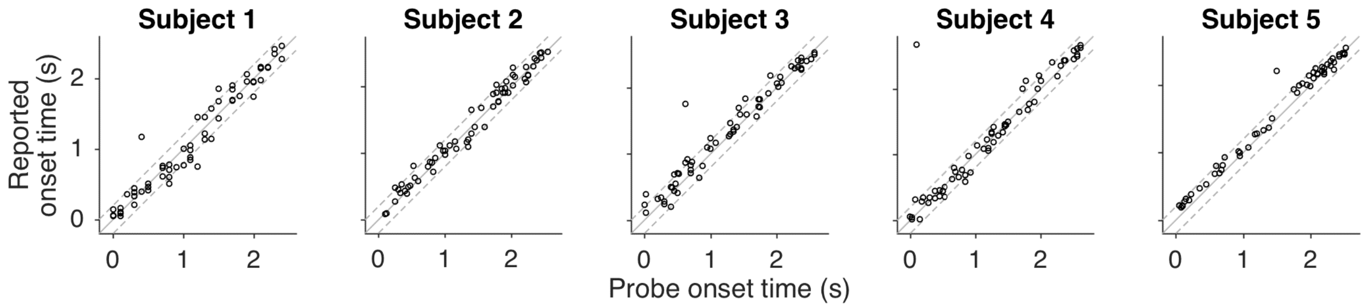

Figure S1. Accuracy of the clock timing report of a tone. Related to Figure 1.
Previous work suggests that timing reports using a clock can be unreliable [S1]. To test the validity of the clock reports, we used a separate training task where subjects used the clock to indicate the timing of a brief tone, presented at a random time during motion viewing. The solid gray line is the identity line and the dashed lines indicate ±200 ms from the actual beep time. Subjects received auditory feedback that indicated whether their report was within this range of accuracy. The time of the beep accounted for 83 to 98% of the variance in the reported beep times ($p < 10^{-20}$ in all subjects). The regression slope was close to 1 (range: 0.93–0.99) and offset close to zero (0.00–0.16). The responses closely followed the actual beep onset (standard deviation of the timing error: 0.11–0.34 s).

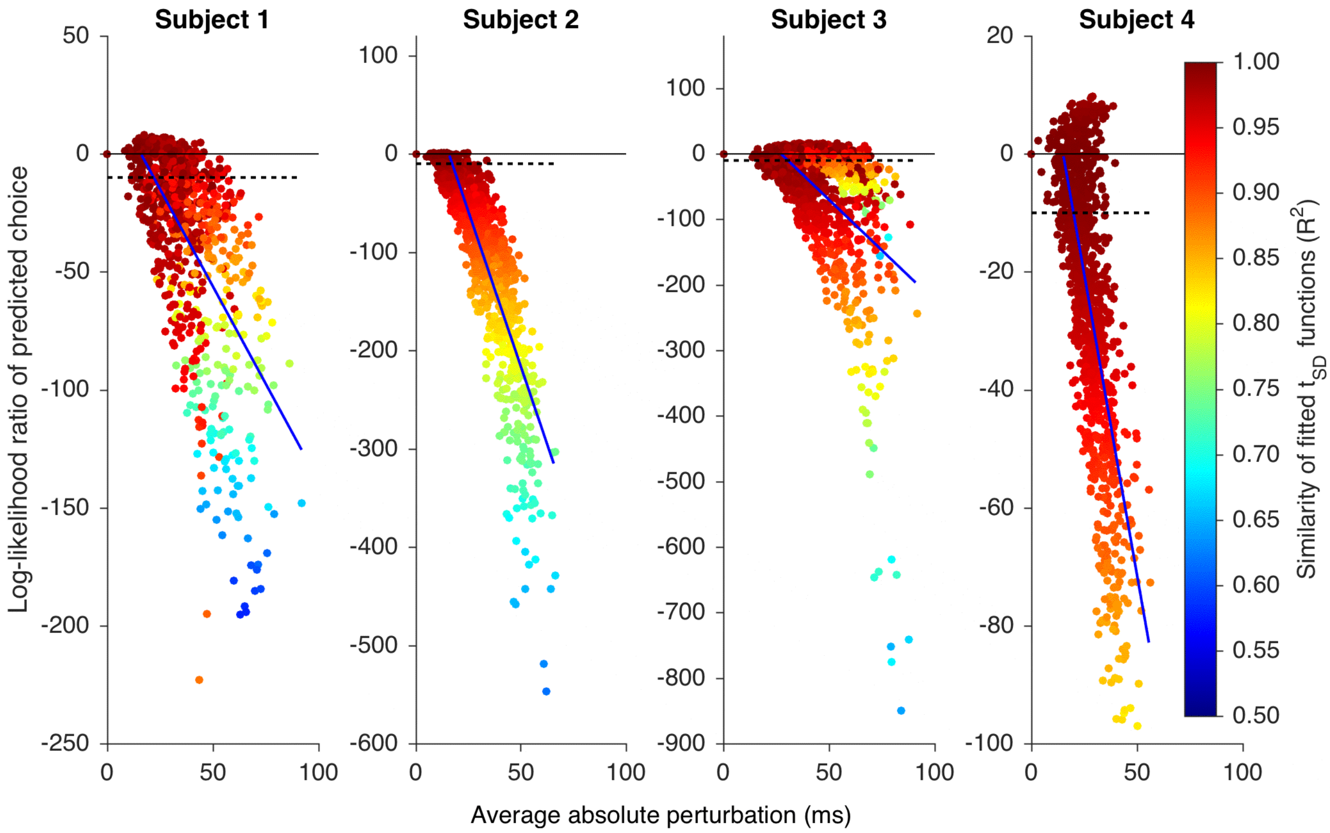

Figure S2. Order preserving perturbations to subjective decision times impair choice prediction. Related to Figure 3.

Log likelihood ratio (logLR) of the choice predictions established from surrogate times (t_{surr}) relative to choice predictions from the observed t_{SD}. The logLR is plotted as a function of the average absolute difference between t_{surr} and t_{SD}. Jittered data (t_{surr}) were generated by permuting the intervals between mean t_{SD} while preserving the minimum, maximum and order of the original t_{SD} (see STAR Methods). Each permutation can be characterized by the mean absolute deviation of the surrogate t_{SD} (abscissa) and the correlation between the fits of Equation 2 to perturbed and unperturbed data (color of the marker). The regression (blue lines) furnishes a test of the null hypothesis that only the order of the t_{SD} matters for predicting the choices ($p < 10^{-60}$ for subjects 1–4). The regression provides an estimate of the degree of perturbation to the t_{SD} required to produce reliably poorer predictions of the choice data. For example, perturbations of t_{SD} by 19 to 31 ms (across subjects) yield predicted choice functions that are 10 units of log likelihood ratio worse, on average, than the predictions from the observed t_{SD} (i.e., odds ratio < 0.0001; horizontal black dashed lines). The correlation between fits to t_{SD} and t_{surr} (color) furnishes the useful insight that larger perturbations of the t_{SD} yield choice predictions that are as good (or better) than the original data only when they fail to yield a different fit (i.e., high R^2) by bounded drift-diffusion (Equation 2).

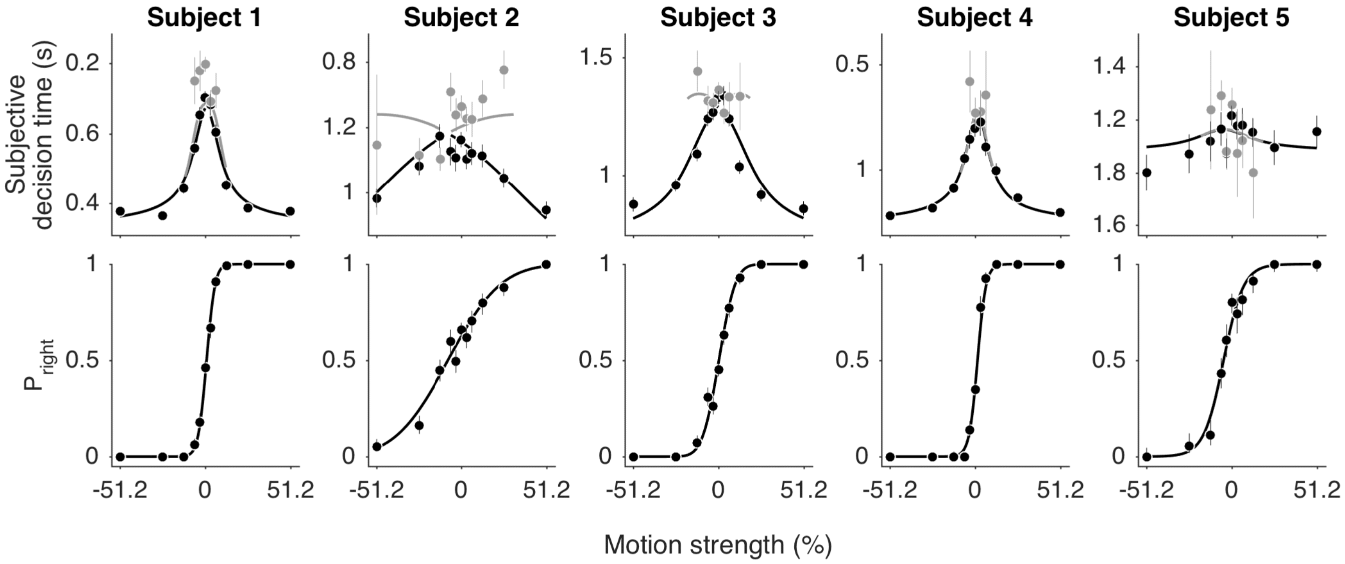

Figure S3. Fits of the elaborated drift-diffusion model with time-dependent collapsing bounds to subjective decision times on the controlled-duration experiment. Related to Figure 4. Black points are identical to those in Figure 2 of the main text. Grey symbols show the t_{SD} associated with errors (mean ± SEM). Solid lines are model fits. Subjective decision times for errors are only plotted for motion strengths that have at least 3 trials. Note that t_{SD} on error trials are longer on average (subjects 1–4: 59–121 ms, $p < 0.03$; subject 5: 11 ms, $p = 0.8$; see STAR Methods). This feature is also explained by collapsing termination bounds [S2,3], although the pattern is only weakly captured by the fits (gray curves).

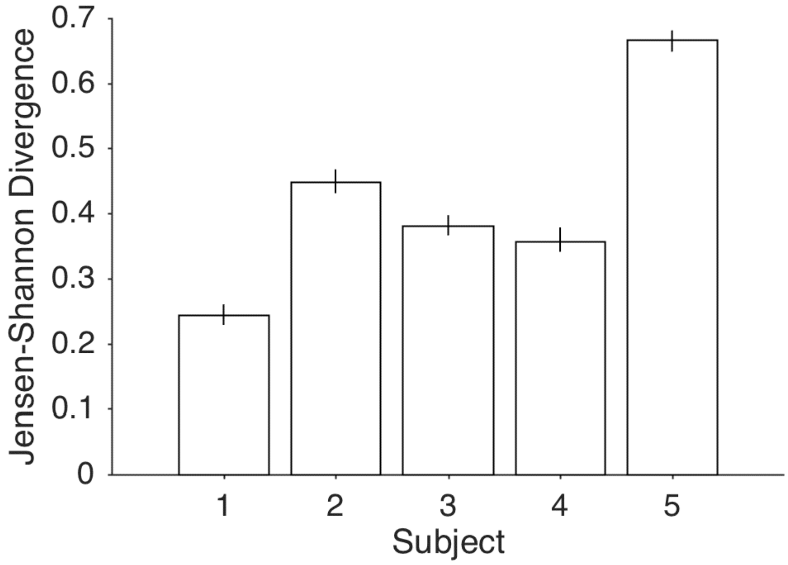

Figure S4. Goodness of fit to observed t_{SD} distributions in the controlled-duration experiment using a drift-diffusion model with time-dependent, collapsing bounds. Related to Figure 4. The bars show the median of the bootstrapped Jensen-Shannon divergence for each of the five subjects (subjects 1–5). Error bars show the 95% CI (see STAR Methods).

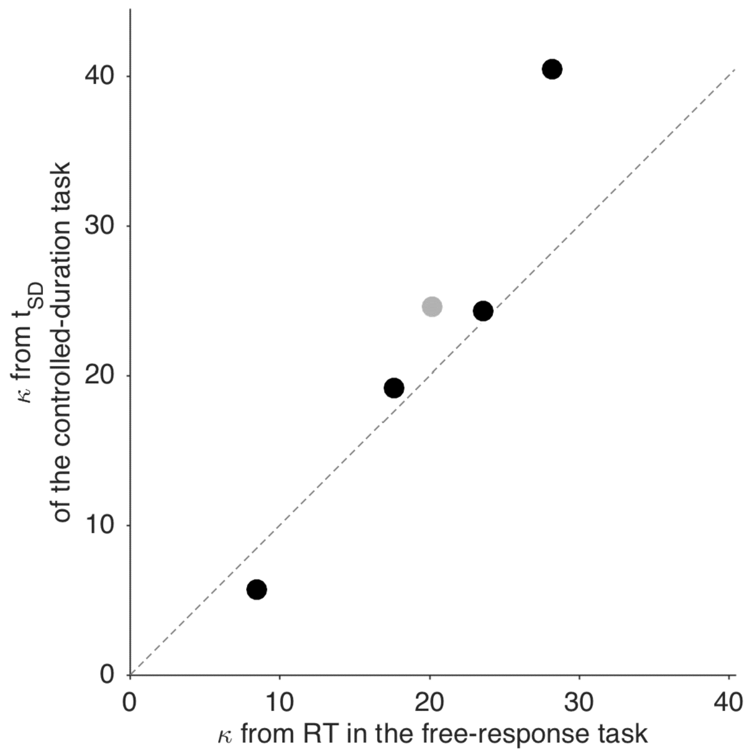

Figure S5. Sensitivity to motion is similar whether derived from subjective decision times (*t_{SD}*) or reaction times. Related to Figure 6.
Scatter plot compares signal-to-noise scaling parameter (κ) derived from the choice-RT data of the free-response and t_{SD} of the controlled-duration task. The κ for the ordinate for subjects 1–4 comes from the fit to the t_{SD} ignoring choices; for subject 5 it comes from the joint fit to the t_{SD} and choices (gray dot). The dotted line is the identity line. In addition to statistics described in the main text, we performed a bootstrap test, using the sum of the squared difference between fitted κ values:

$$D = \sum_{s=1}^{5} \left(\kappa_{t_{SD}}^{s} - \kappa_{RT}^{s} \right)^2$$

where s is the index for each subject. To assess the distribution of *D* under the null hypothesis, we computed the value of *D* for all $n!$ possible permutations across subjects and computed the proportion of *D* that are not larger than the original *D*, to obtain p-values [S4,5]. The p-value is 0.017 with all five subjects, and 0.042 with subject 5 excluded. The analysis supports the hypothesis that the κ parameters from the t_{SD} (controlled-duration task) and the RT (free-response task) are significantly closer to each other than by chance.

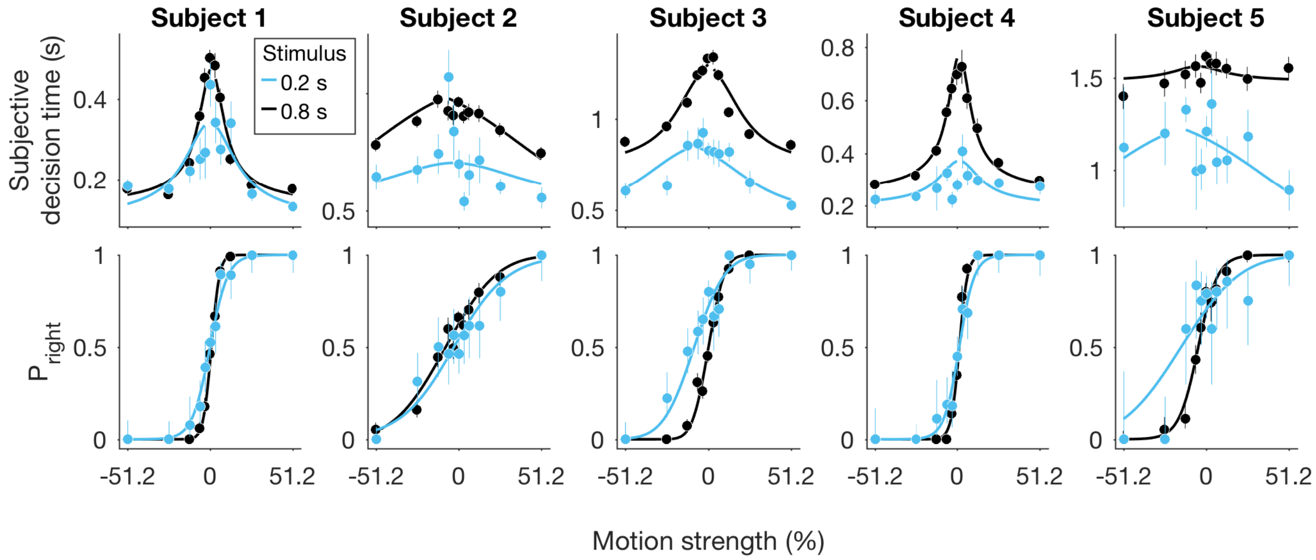

Figure S6. t_{SD} and performance as a function of motion viewing duration. Related to Figure 4.

We compared performance on trials using 200 ms versus 800 ms viewing durations. Not surprisingly, sensitivity to random dot motion was better on the longer duration trials. This was supported by logistic regression:

$$P_{right} = [1 + \exp(-(k_1 + k_2 C \ + k_3 I + k_4 I \cdot C)]^{-1}$$

where *I* is an indicator variable (0 for 800 ms and 1 for 200 ms display). This improvement was statistically reliable for all subjects except subject 2 who showed the same trend (H_0: $k_4=0$; $p < 0.002$; for subject 2, $p = 0.32$; t-test). Unsurprisingly, the t_{SD} were shorter on the trials with 200 ms versus 800 ms viewing durations (ANOVA with categorical factors of absolute coherence, random dot duration and their interaction; $p < 10^{-6}$ for all subjects). We also confirmed these conclusions were robust to analyzing only a subset of trials matched for the sum of motion and delay durations (i.e., 200 ms motion plus 800 ms delay and vice versa).

	B	κ	C_0	$t_{ND,left}$	$t_{ND,right}$
Subject 1	0.95 ± 0.01	28.2 ± 1.1	-0.014 ± 0.001	0.543 ± 0.007	0.538 ± 0.008
Subject 2	0.93 ± 0.01	8.5 ± 0.4	-0.037 ± 0.004	0.575 ± 0.020	0.581 ± 0.019
Subject 3	1.07 ± 0.01	17.6 ± 0.6	0.010 ± 0.002	0.478 ± 0.010	0.464 ± 0.010
Subject 4	1.13 ± 0.01	23.6 ± 0.9	0.009 ± 0.001	0.405 ± 0.005	0.385 ± 0.008
Subject 5	0.84 ± 0.01	20.2 ± 1.2	-0.003 ± 0.003	0.597 ± 0.011	0.572 ± 0.009

Table S1. Parameters of the drift-diffusion model fit jointly to the RT and choice data in the free-response task. Related to Figure 6.
Parameters are shown ±SE.

	B	κ	C_0	$t_{ND,left}$	$t_{ND,right}$
Subject 1	0.97 ± 0.01	40.4	-0.013 ± 0.001	0.579 ± 0.006	0.579 ± 0.007
Subject 2	0.99 ± 0.01	5.7	-0.045 ± 0.005	0.414 ± 0.013	0.436 ± 0.013
Subject 3	1.07 ± 0.01	19.2	0.010 ± 0.002	0.492 ± 0.008	0.480 ± 0.008
Subject 4	1.13 ± 0.01	24.3	0.008 ± 0.001	0.408 ± 0.004	0.389 ± 0.006
Subject 5	0.84 ± 0.01	24.6	-0.003 ± 0.003	0.621 ± 0.008	0.590 ± 0.007

Table S2. Parameters of the drift-diffusion model fit jointly to the RT and choice data in the free-response task with κ fixed from the fits to the t_{SD} of the controlled-duration task. Related to Figure 6.
Parameters are shown ±SE. Grey cells indicate parameters that are fixed (from Table 1).

	B_0	B_{log}	t_β	κ	C_0	μ	σ
Subject 1	0.58 ± 0.02	2.53 ± 0.43	0.26 ± 0.02	33.9 ± 1.9	0.008 ± 0.002	0.130 ± 0.007	0.103 ± 0.006
Subject 2	1.68 ± 0.23	1.31 ± 0.19	0.26 ± 0.02	4.1 ± 0.4	-0.079 ± 0.011	0.245 ± 0.071	0.163 ± 0.018
Subject 3	1.05 ± 0.05	4.17 ± 0.92	0.27 ± 0.00	14.2 ± 0.9	0.002 ± 0.003	0.667 ± 0.027	0.268 ± 0.009
Subject 4	0.67 ± 0.02	3.02 ± 1.06	0.80 ± 0.08	27.5 ± 2.0	0.014 ± 0.003	0.236 ± 0.012	0.151 ± 0.009
Subject 5	0.21 ± 0.08	3.01 ± 1.06	0.49 ± 0.24	33.3 ± 10.2	-0.050 ± 0.008	1.497 ± 0.039	0.408 ± 0.017

Table S3. Parameters of the drift-diffusion model with collapsing bounds fit jointly to the t_{SD} and choice data of the controlled-duration task. Related to Figure 4.
Parameters are shown ±SE.

Supplemental References

1. Miller, J., Vieweg, P., Kruize, N., and McLea, B. (2010). Subjective reports of stimulus, response, and decision times in speeded tasks: how accurate are decision time reports? Consciousness and Cognition *19*, 1013–1036.

2. Churchland, A.K., Kiani, R., and Shadlen, M.N. (2008). Decision-making with multiple alternatives. Nat. Neurosci. *11*, 693–702.

3. Drugowitsch, J., Moreno-Bote, R., Churchland, A.K., Shadlen, M.N., and Pouget, A. (2012). The cost of accumulating evidence in perceptual decision making. J. Neurosci. *32*, 3612–3628.

4. North, B.V., Curtis, D., and Sham, P.C. (2002). A note on the calculation of empirical P values from Monte Carlo procedures. Am. J. Hum. Genet. *71*, 439–441.

5. North, B.V., Curtis, D., and Sham, P.C. (2003). A note on the calculation of empirical P values from Monte Carlo procedures. Am. J. Hum. Genet. *72*, 498–499.
