# Supplementary figures and images for: Piercing of Consciousness as a Threshold-Crossing Operation
Source: Curr Biol. 2017 Aug 7;27(15):2285–2295.e6. doi: 10.1016/j.cub.2017.06.047 (PMC5558038; doi:10.1016/j.cub.2017.06.047)

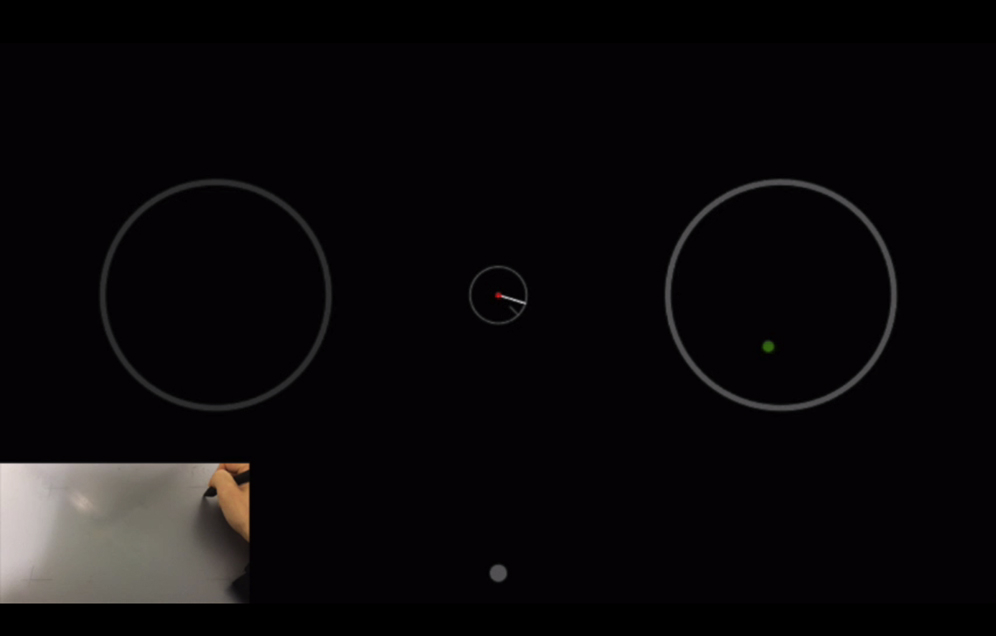

Supplement: Methods S1. Controlled Duration Example Trial [file mmc2.jpg]
