# Supplementary material for: Piercing of Consciousness as a Threshold-Crossing Operation
Source: Curr Biol. 2017 Aug 7;27(15):2285–2295.e6. doi: 10.1016/j.cub.2017.06.047 (PMC5558038; doi:10.1016/j.cub.2017.06.047)

## Slide 1
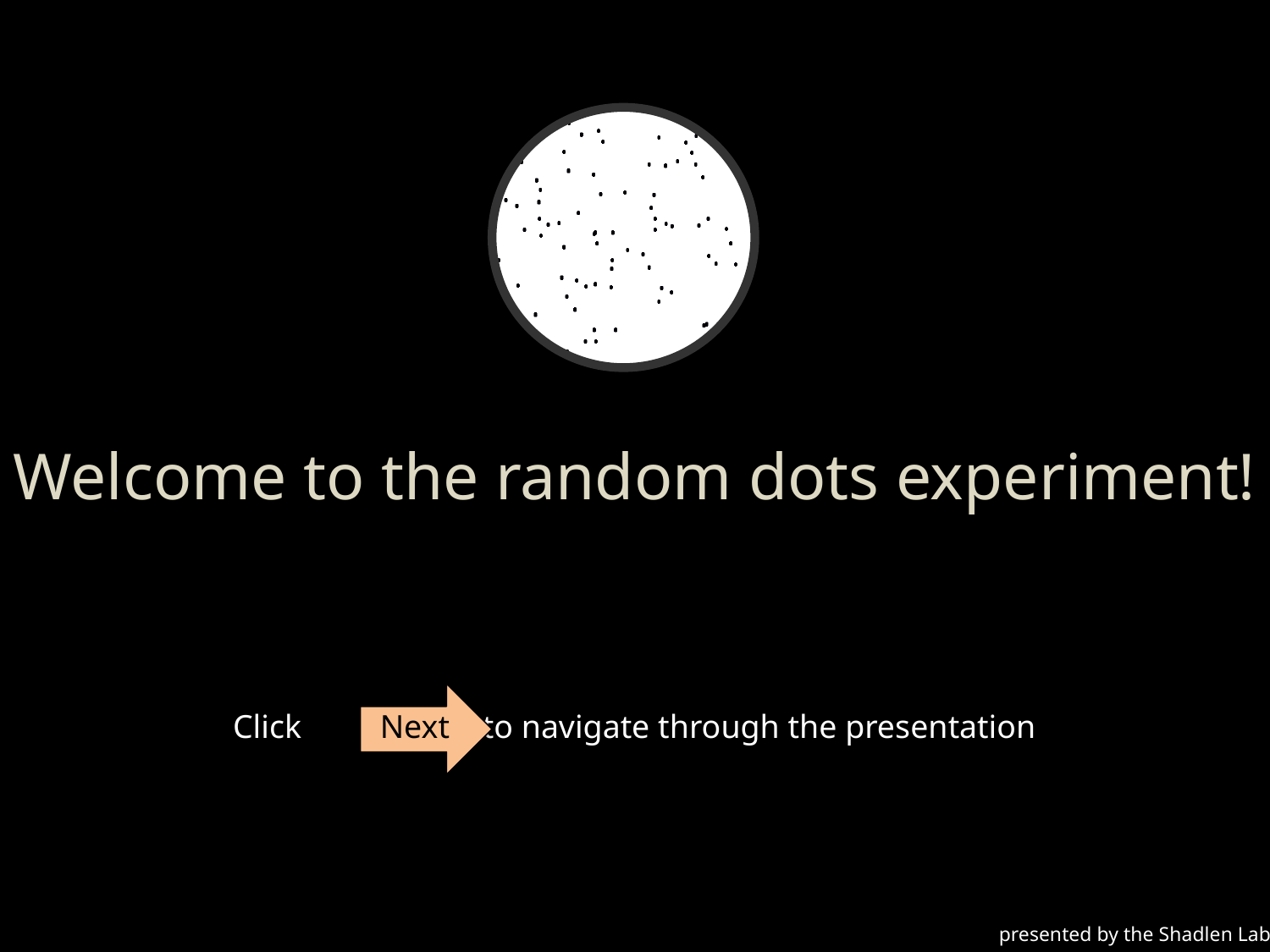

# Welcome to the random dots experiment!
Next
Click to navigate through the presentation
presented by the Shadlen Lab

## Slide 2
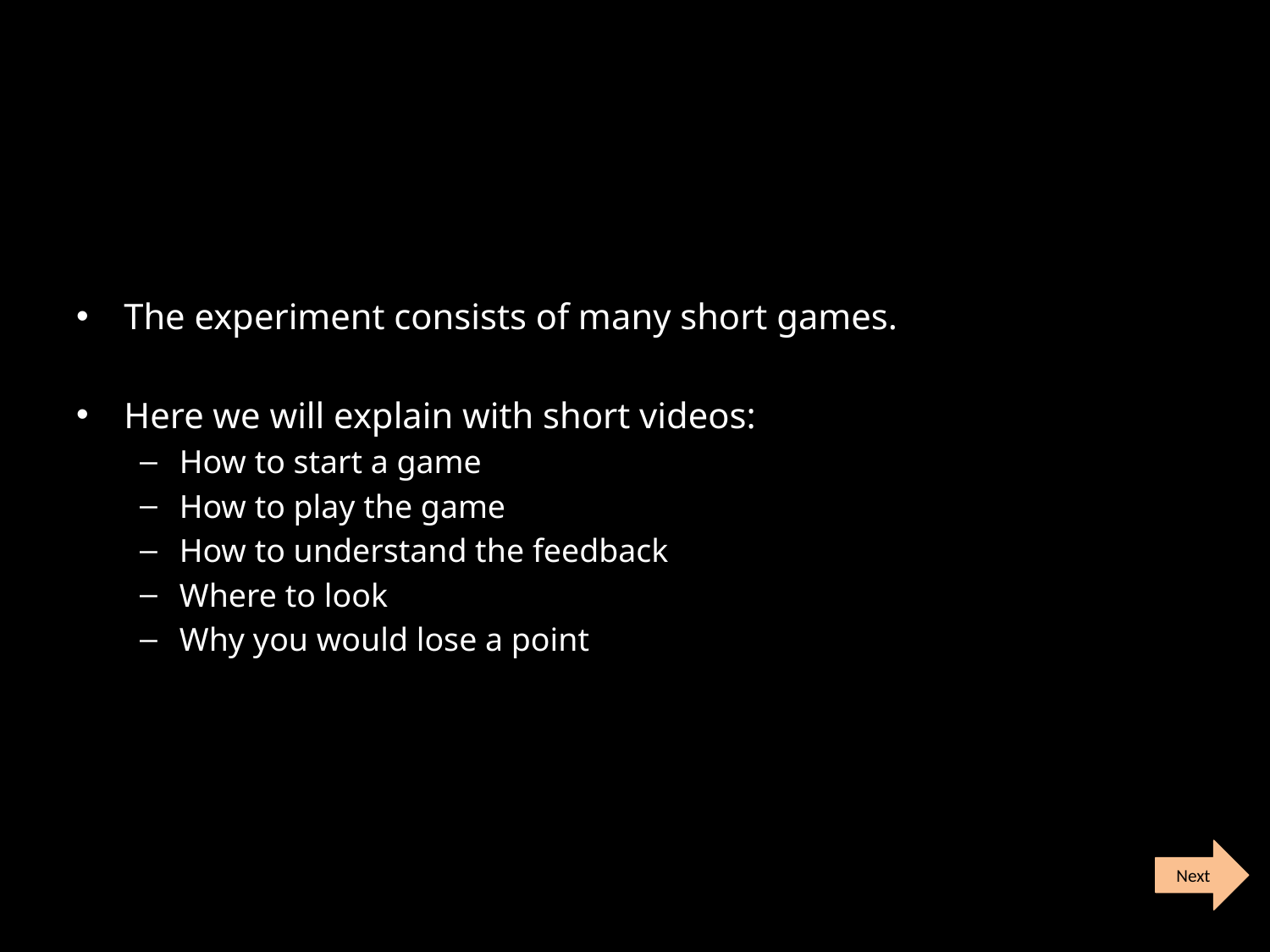

The experiment consists of many short games.
Here we will explain with short videos:
How to start a game
How to play the game
How to understand the feedback
Where to look
Why you would lose a point
Next

## Slide 3
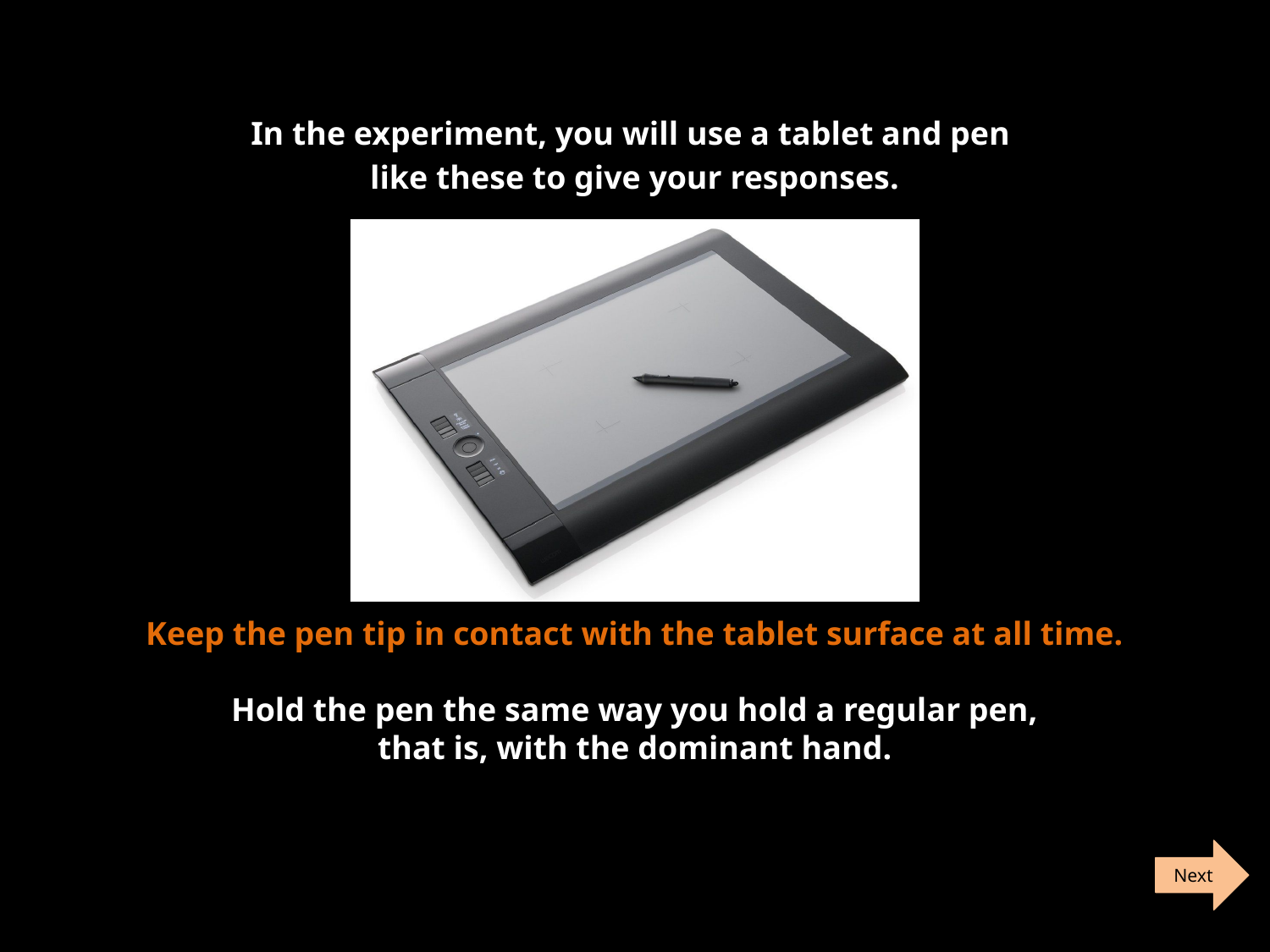

In the experiment, you will use a tablet and pen
like these to give your responses.
Keep the pen tip in contact with the tablet surface at all time.
Hold the pen the same way you hold a regular pen,
that is, with the dominant hand.
Next

## Slide 4
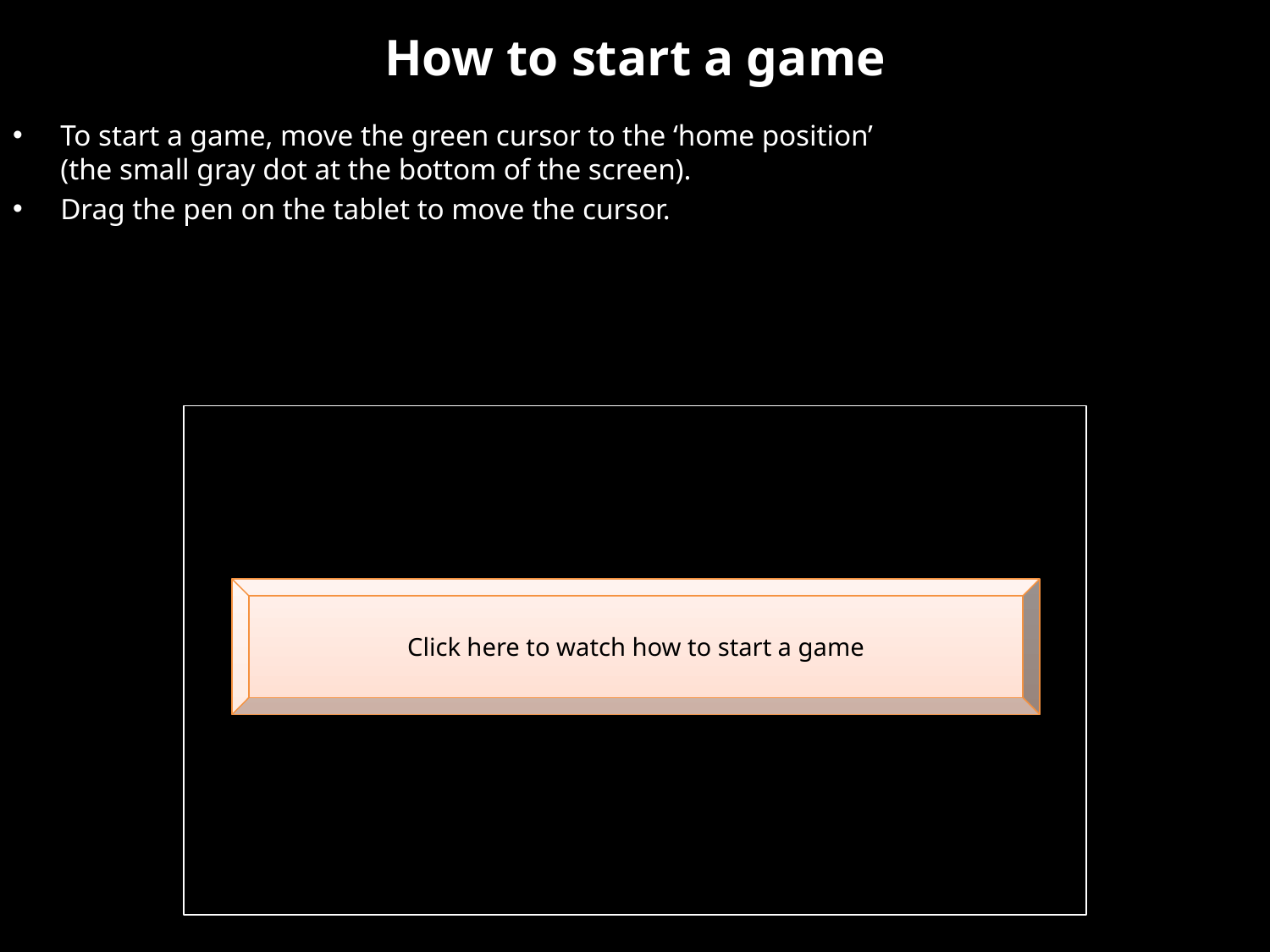

# How to start a game
To start a game, move the green cursor to the ‘home position’
(the small gray dot at the bottom of the screen).
Drag the pen on the tablet to move the cursor.
Click here to watch how to start a game

## Slide 5
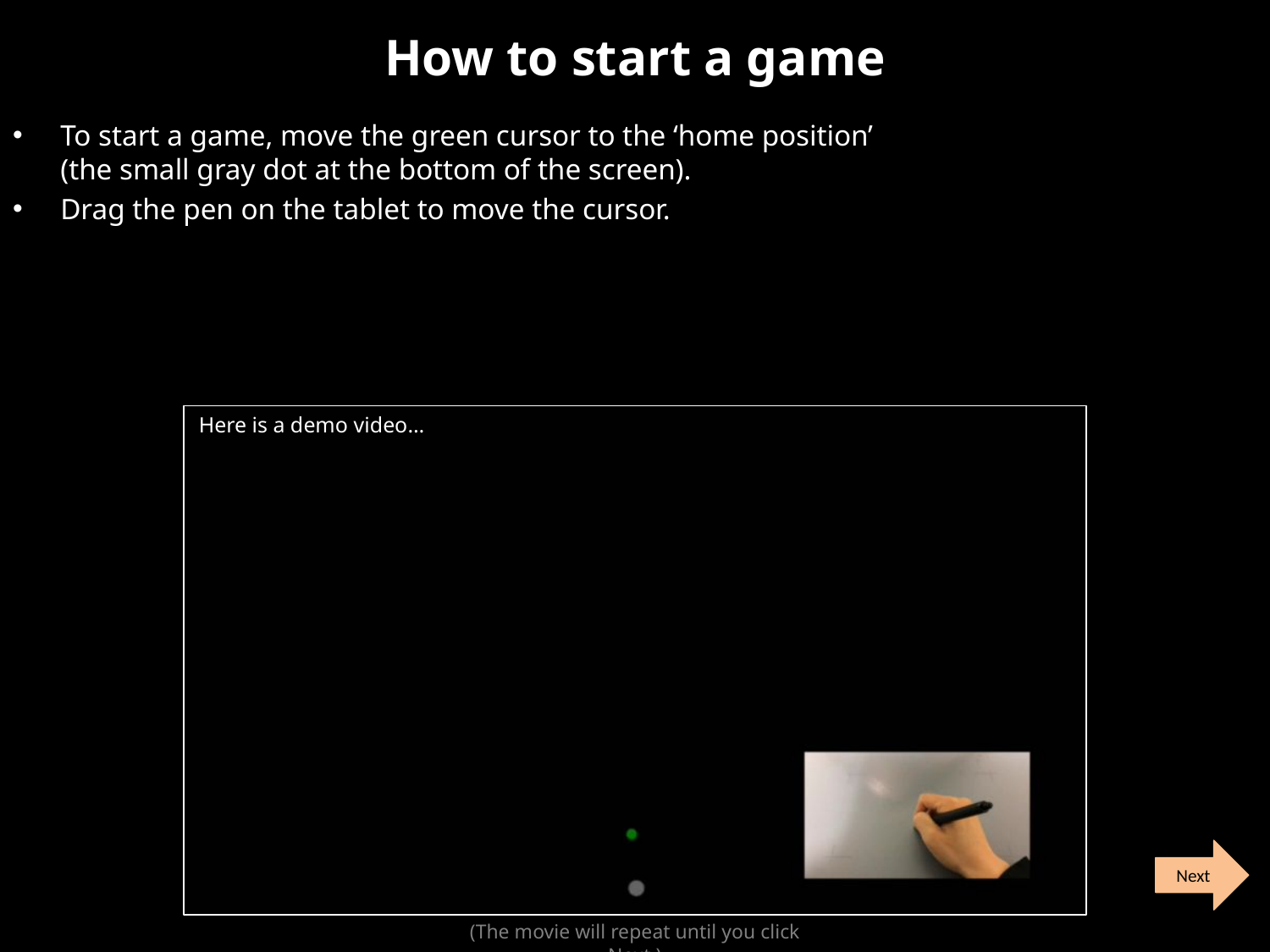

# How to start a game
To start a game, move the green cursor to the ‘home position’
(the small gray dot at the bottom of the screen).
Drag the pen on the tablet to move the cursor.
Here is a demo video…

## Slide 6
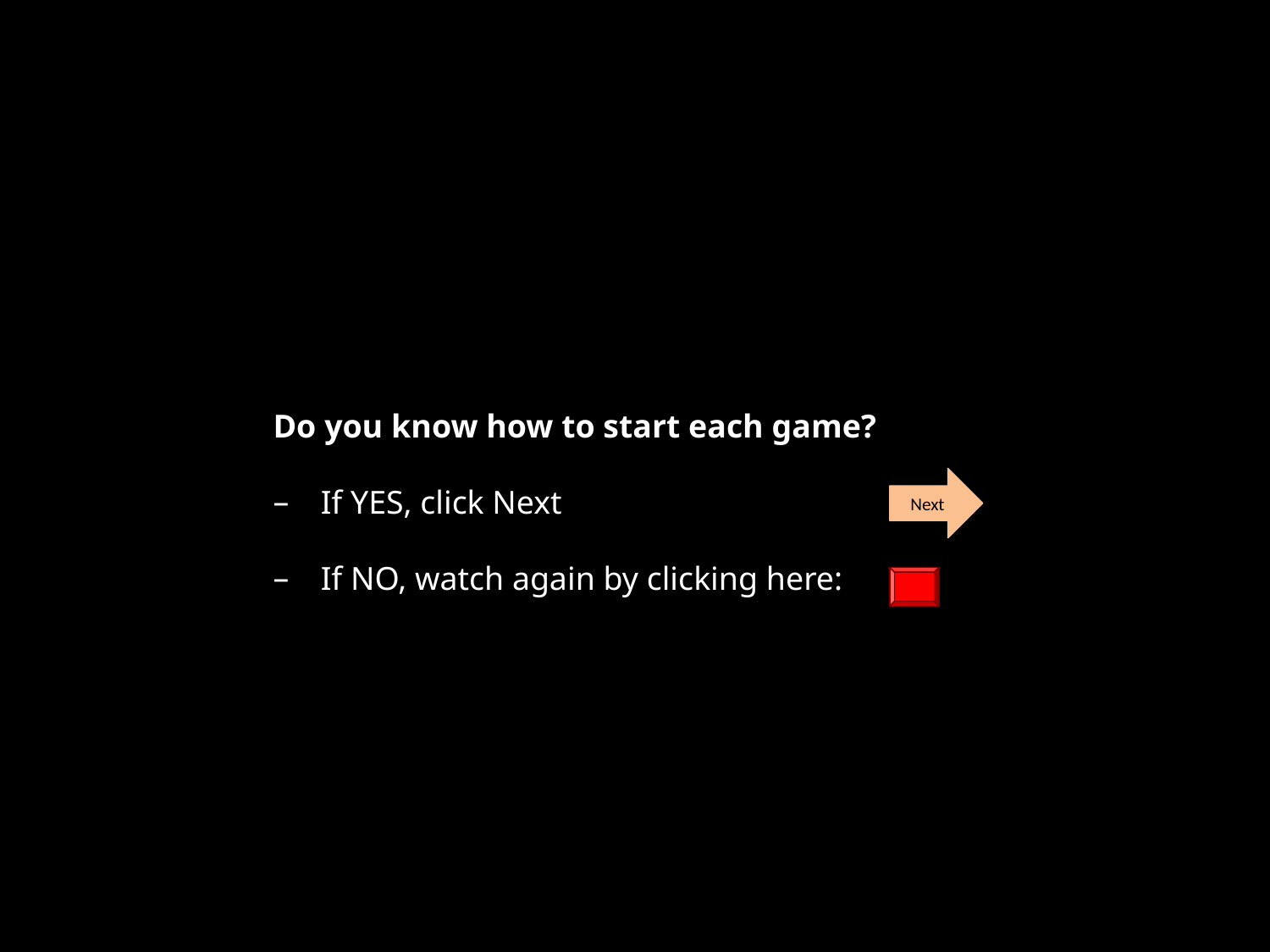

# Do you know how to start each game?
Next
If YES, click Next
If NO, watch again by clicking here:

## Slide 7
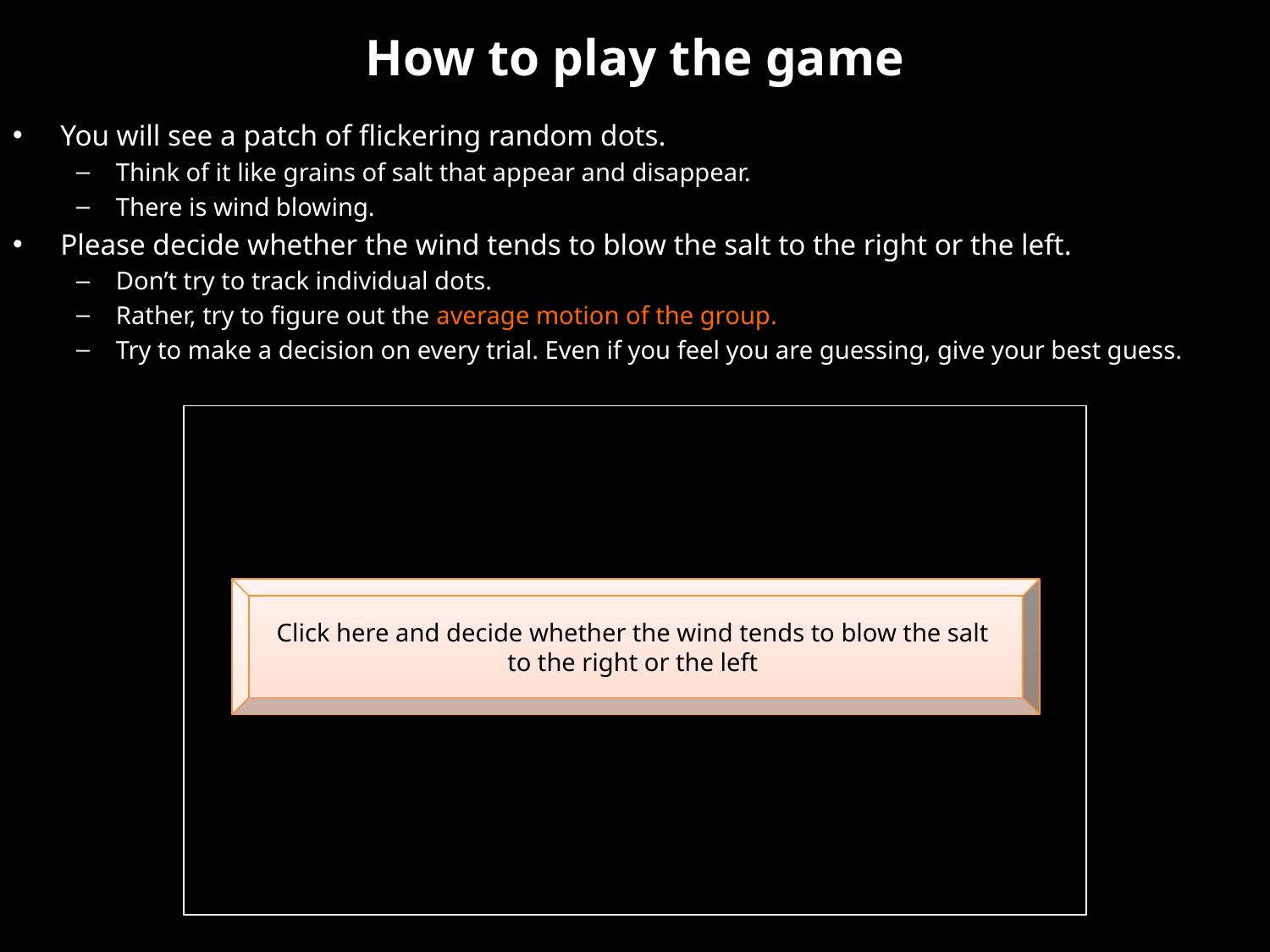

# How to play the game
You will see a patch of flickering random dots.
Think of it like grains of salt that appear and disappear.
There is wind blowing.
Please decide whether the wind tends to blow the salt to the right or the left.
Don’t try to track individual dots.
Rather, try to figure out the average motion of the group.
Try to make a decision on every trial. Even if you feel you are guessing, give your best guess.
Click here and decide whether the wind tends to blow the salt
to the right or the left

## Slide 8
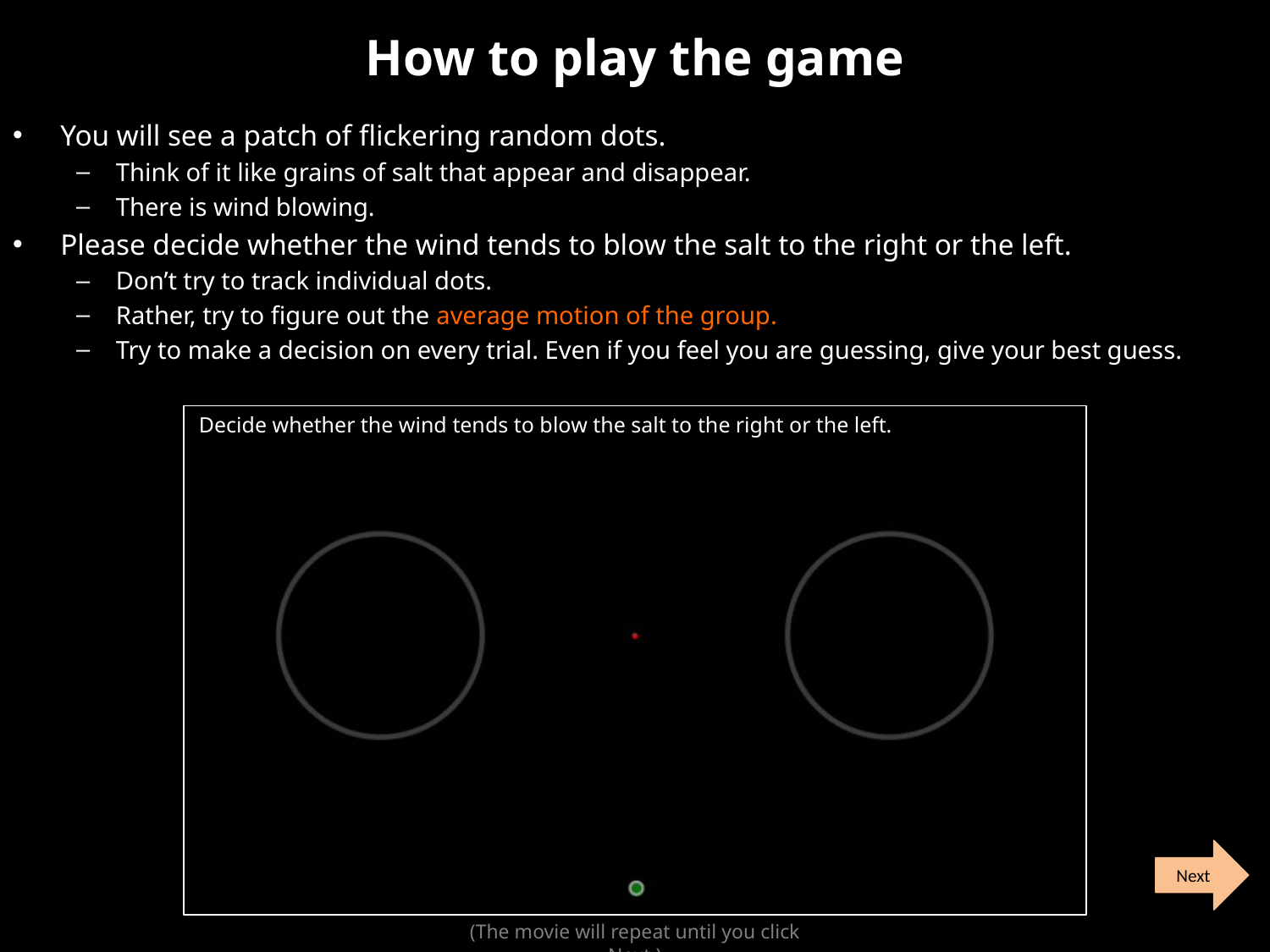

# How to play the game
You will see a patch of flickering random dots.
Think of it like grains of salt that appear and disappear.
There is wind blowing.
Please decide whether the wind tends to blow the salt to the right or the left.
Don’t try to track individual dots.
Rather, try to figure out the average motion of the group.
Try to make a decision on every trial. Even if you feel you are guessing, give your best guess.
Decide whether the wind tends to blow the salt to the right or the left.

## Slide 9
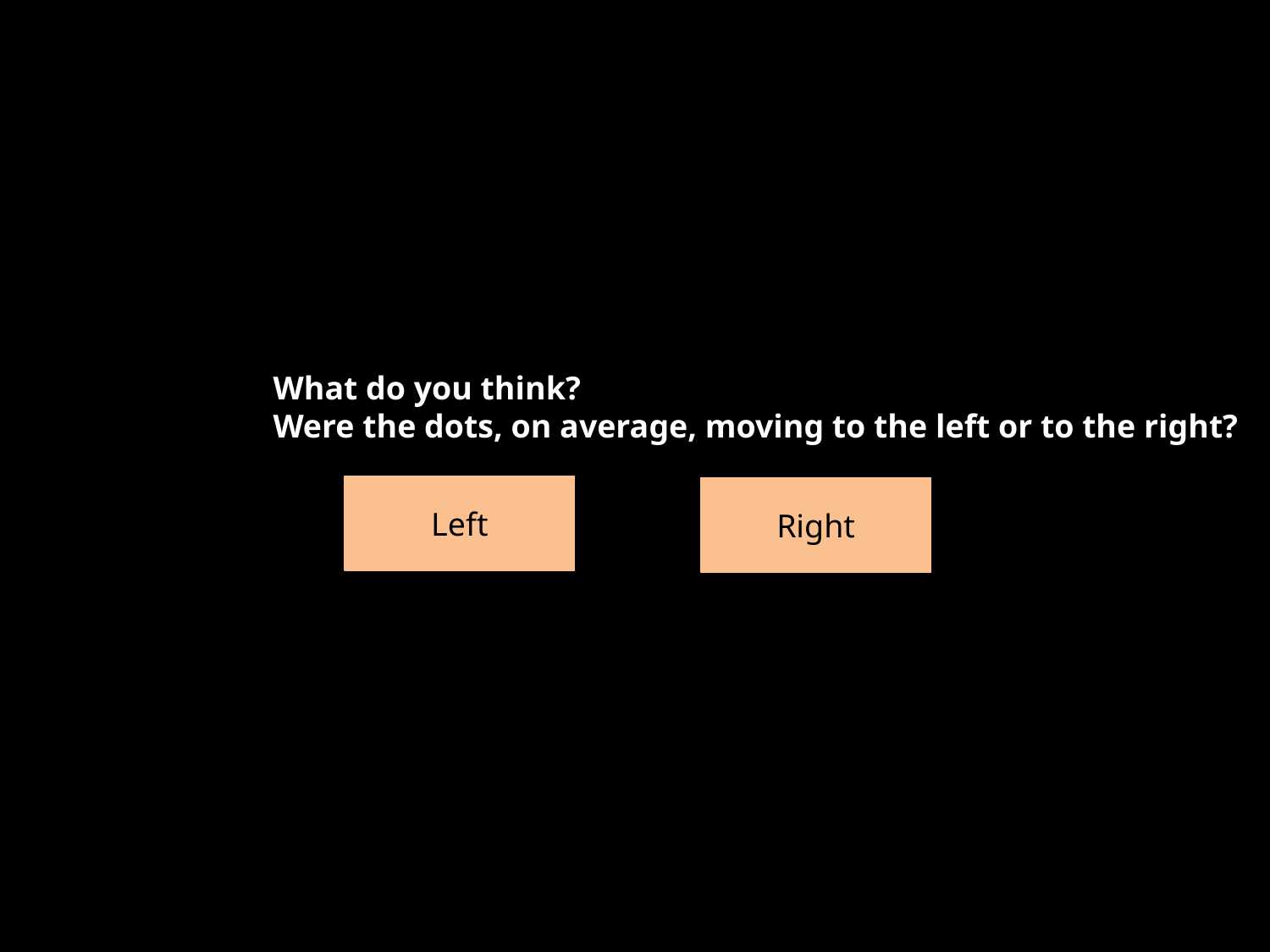

# What do you think? Were the dots, on average, moving to the left or to the right?
Left
Right

## Slide 10
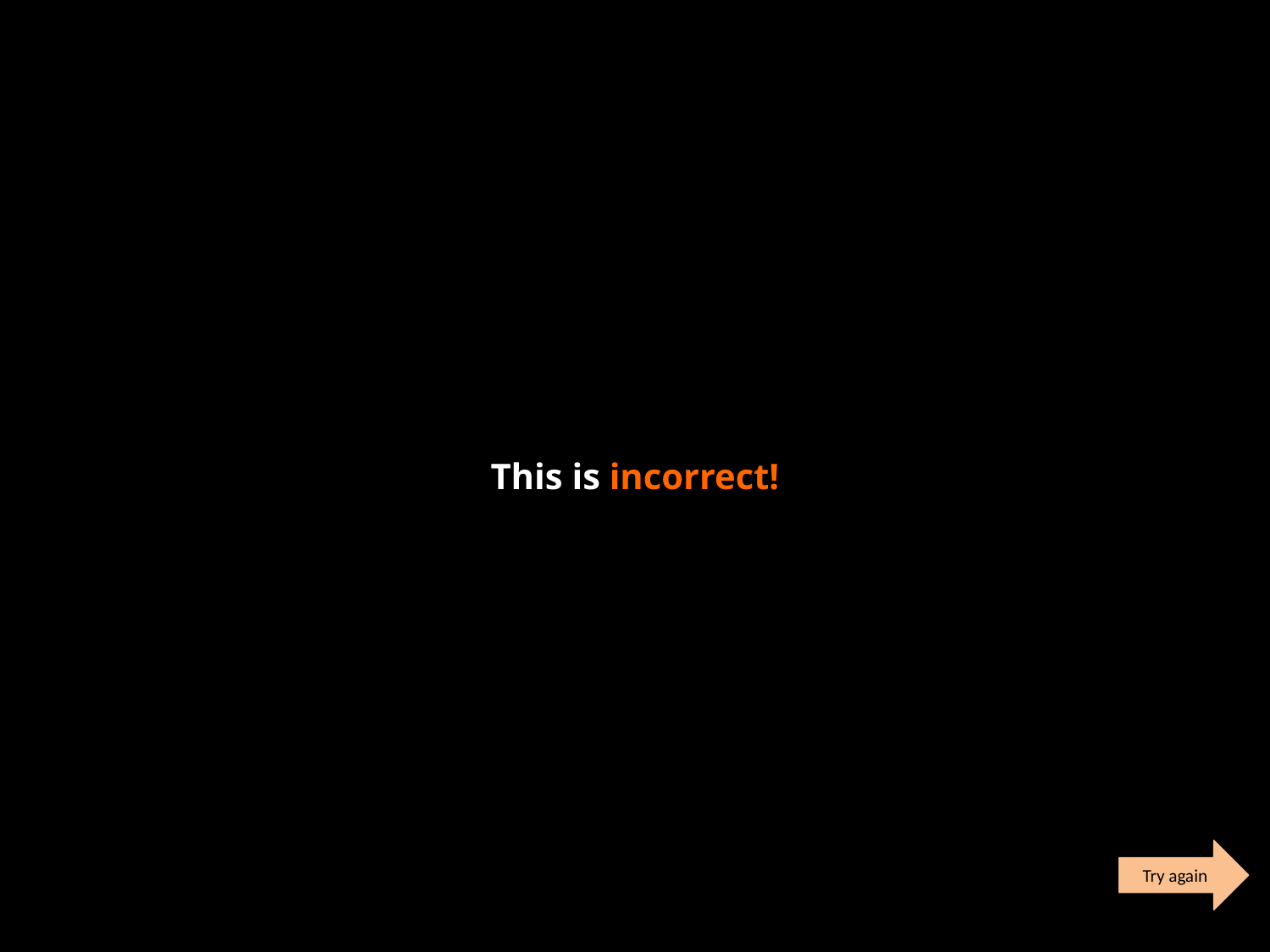

This is incorrect!
Try again

## Slide 11
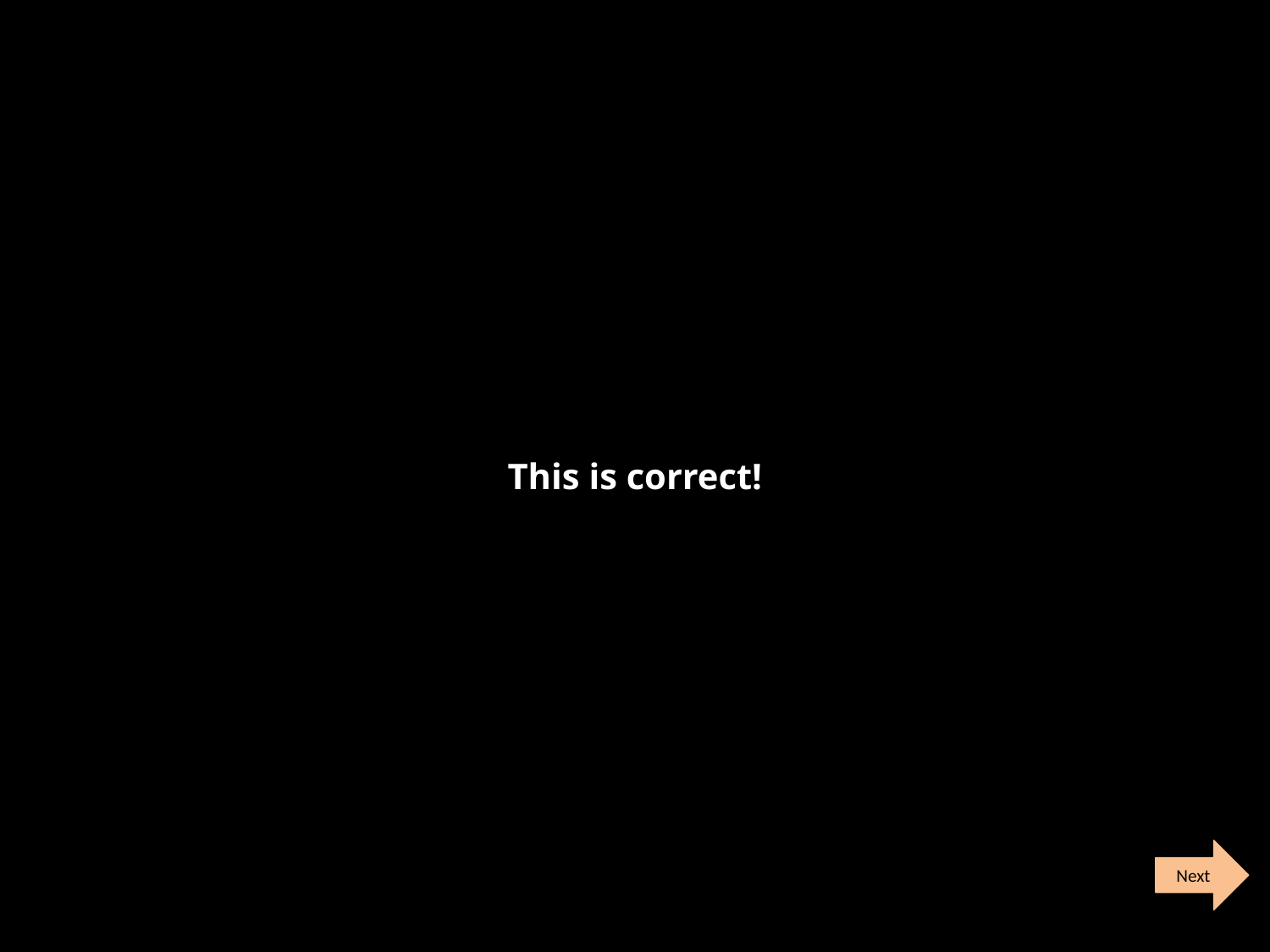

This is correct!
Next

## Slide 12
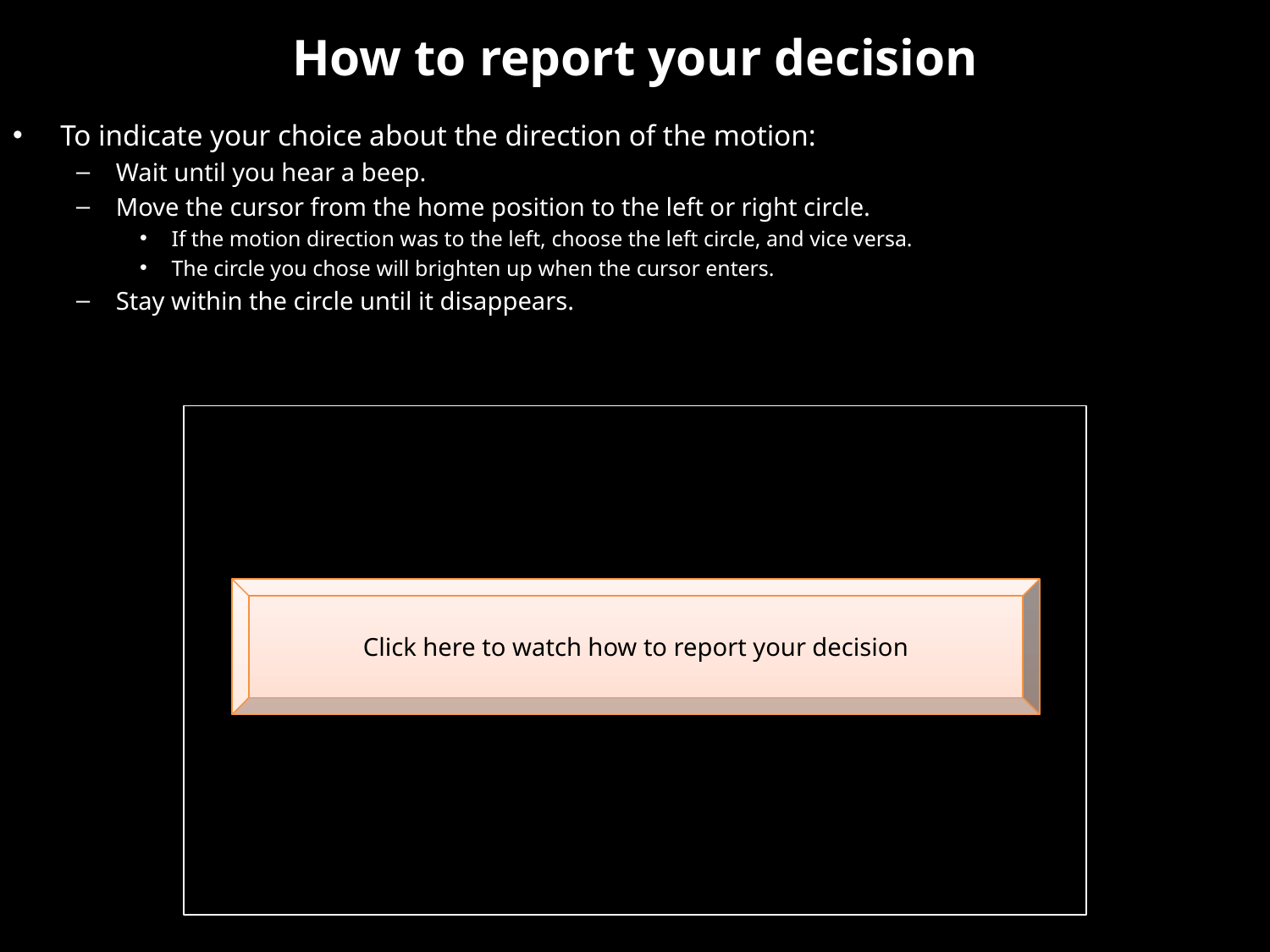

# How to report your decision
To indicate your choice about the direction of the motion:
Wait until you hear a beep.
Move the cursor from the home position to the left or right circle.
If the motion direction was to the left, choose the left circle, and vice versa.
The circle you chose will brighten up when the cursor enters.
Stay within the circle until it disappears.
Click here to watch how to report your decision

## Slide 13
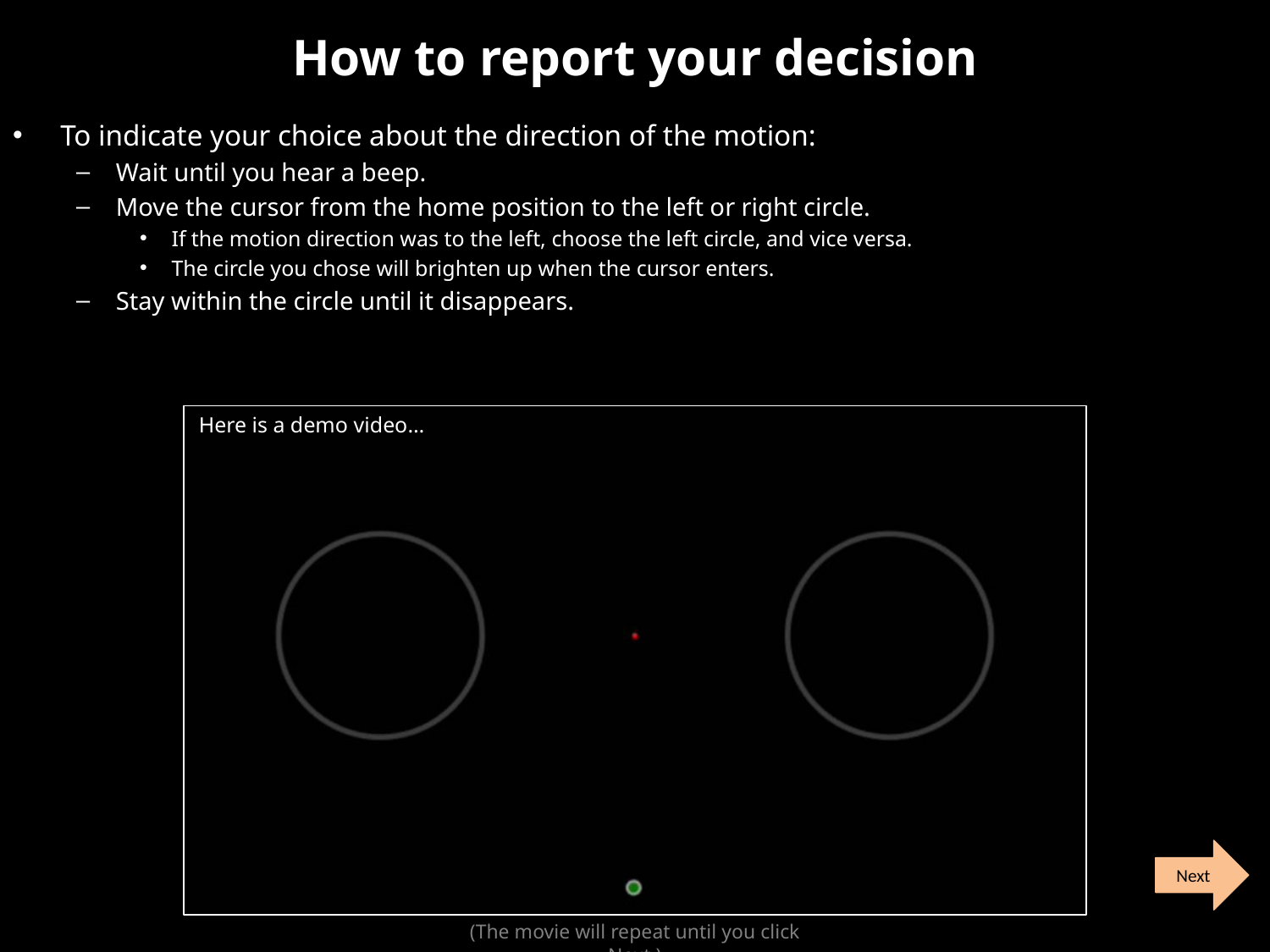

# How to report your decision
To indicate your choice about the direction of the motion:
Wait until you hear a beep.
Move the cursor from the home position to the left or right circle.
If the motion direction was to the left, choose the left circle, and vice versa.
The circle you chose will brighten up when the cursor enters.
Stay within the circle until it disappears.
Here is a demo video…

## Slide 14
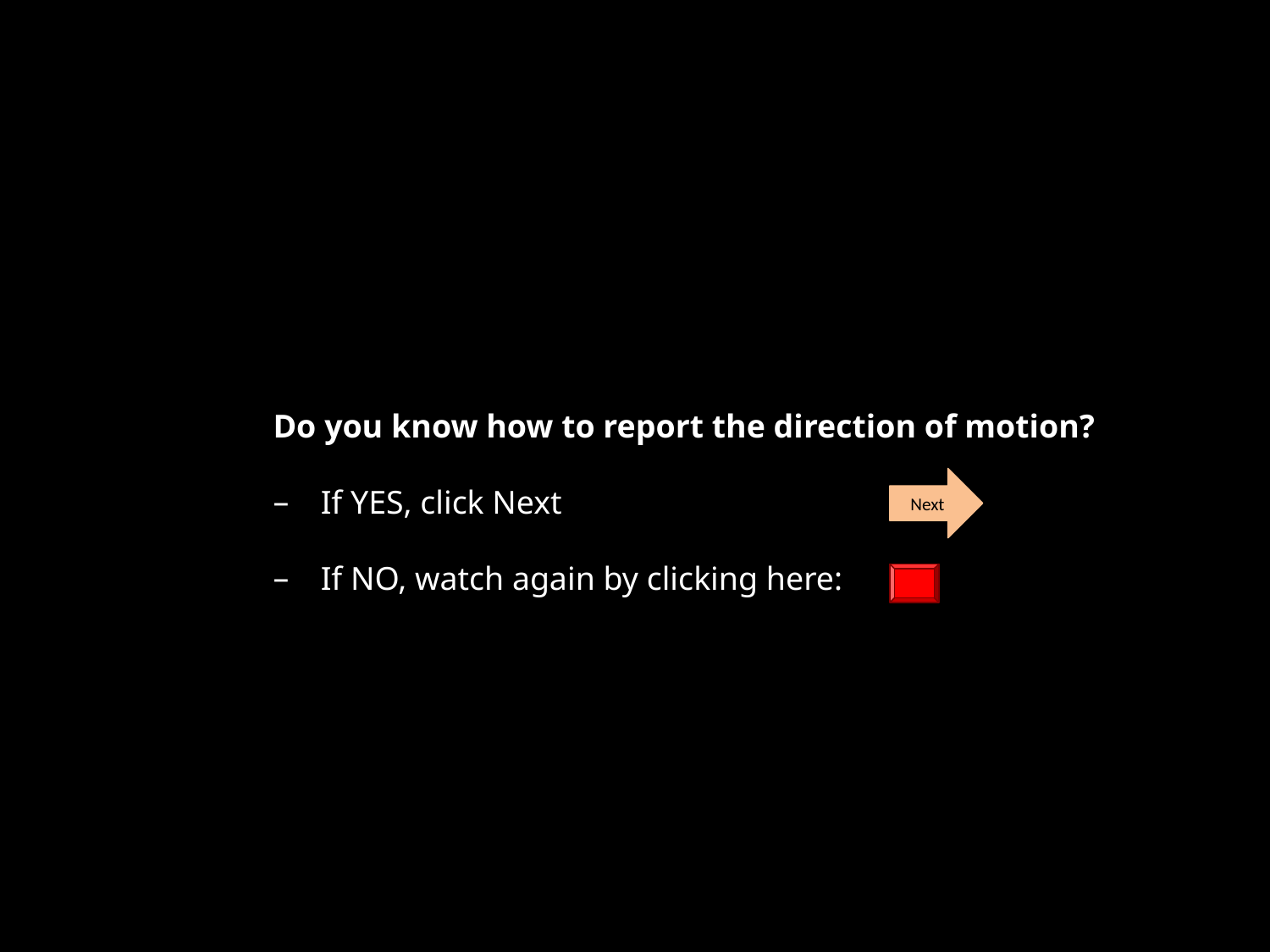

# Do you know how to report the direction of motion?
Next
If YES, click Next
If NO, watch again by clicking here:

## Slide 15
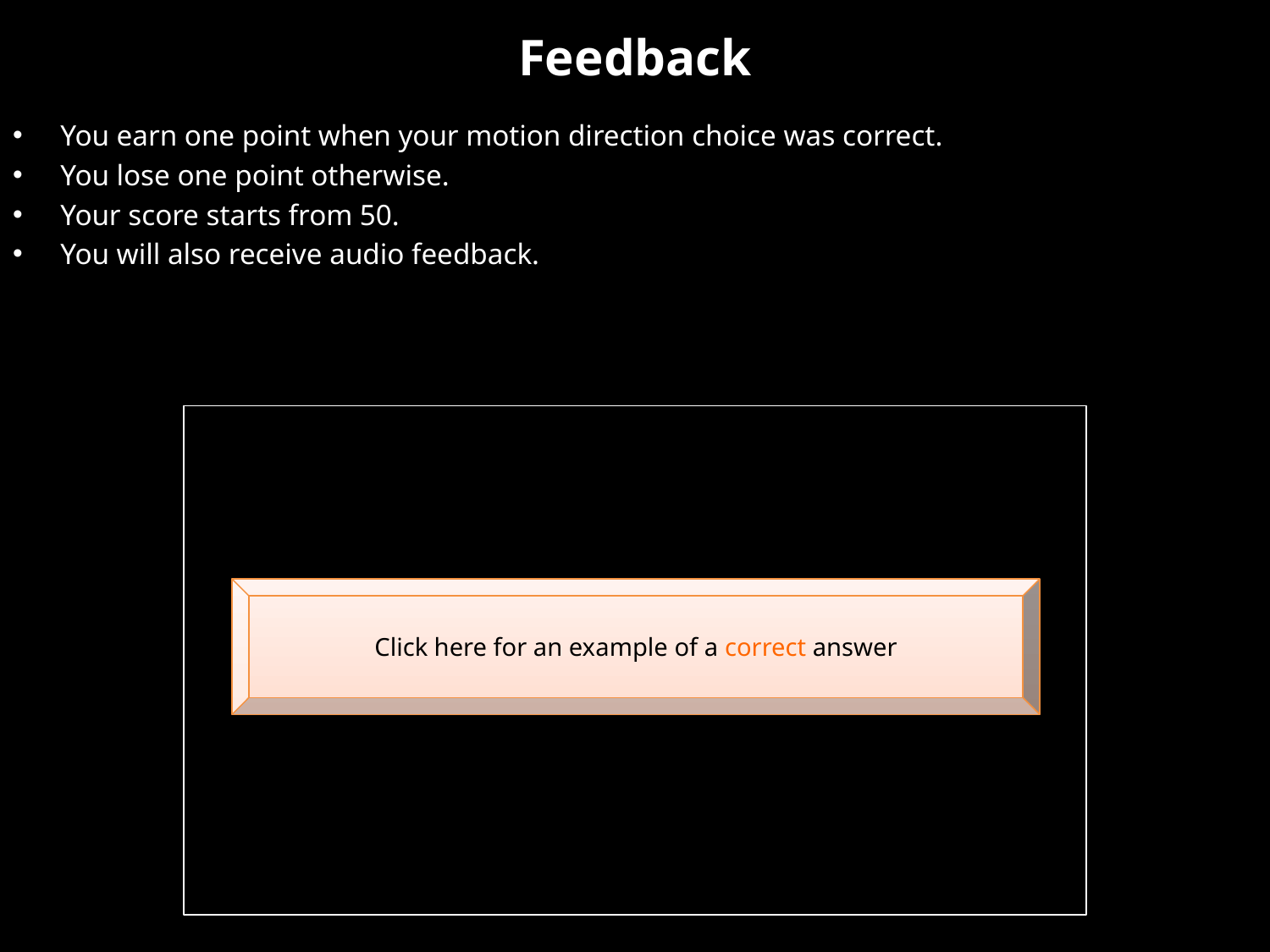

# Feedback
You earn one point when your motion direction choice was correct.
You lose one point otherwise.
Your score starts from 50.
You will also receive audio feedback.
Click here for an example of a correct answer

## Slide 16
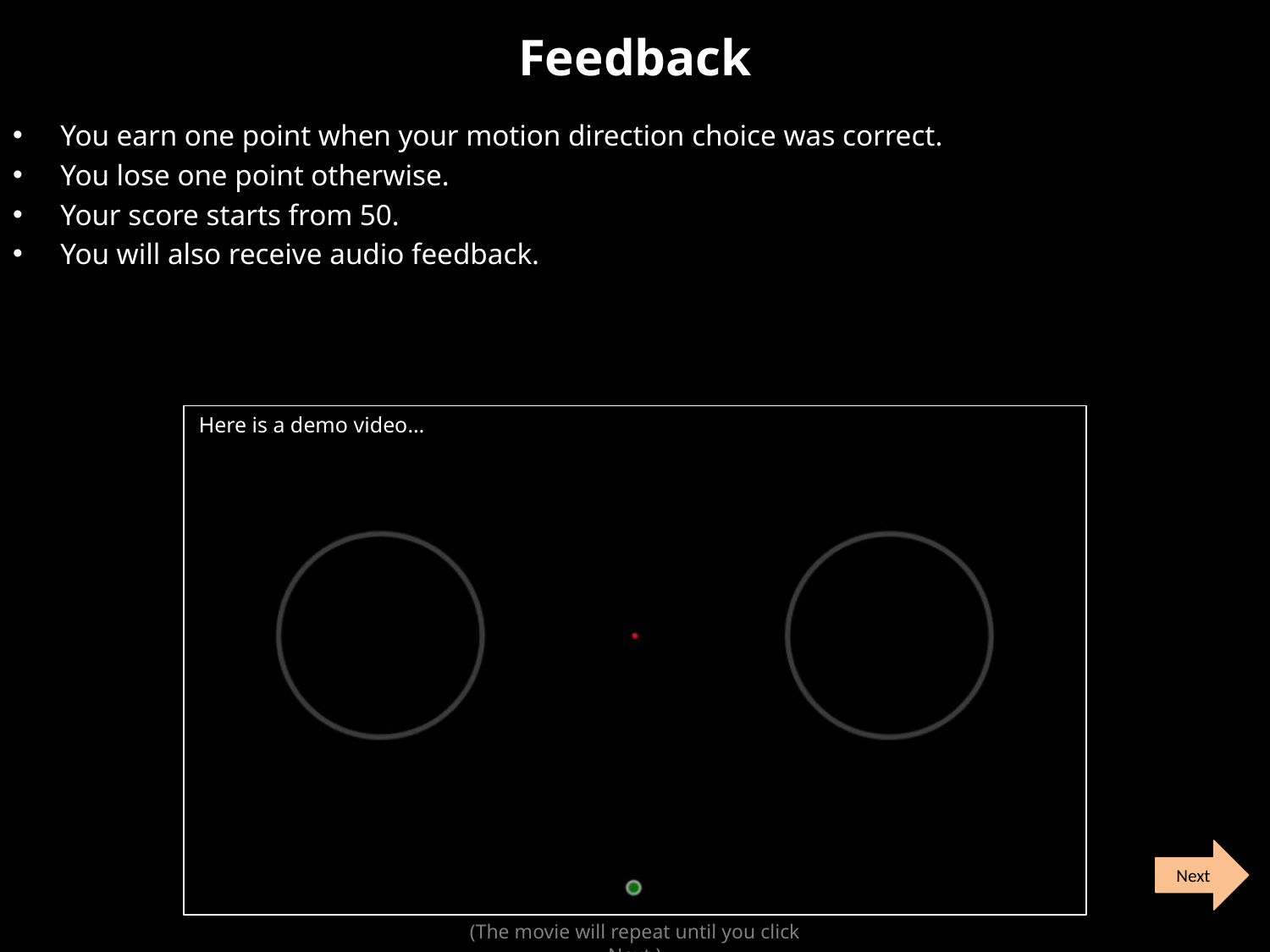

# Feedback
You earn one point when your motion direction choice was correct.
You lose one point otherwise.
Your score starts from 50.
You will also receive audio feedback.
Here is a demo video…

## Slide 17
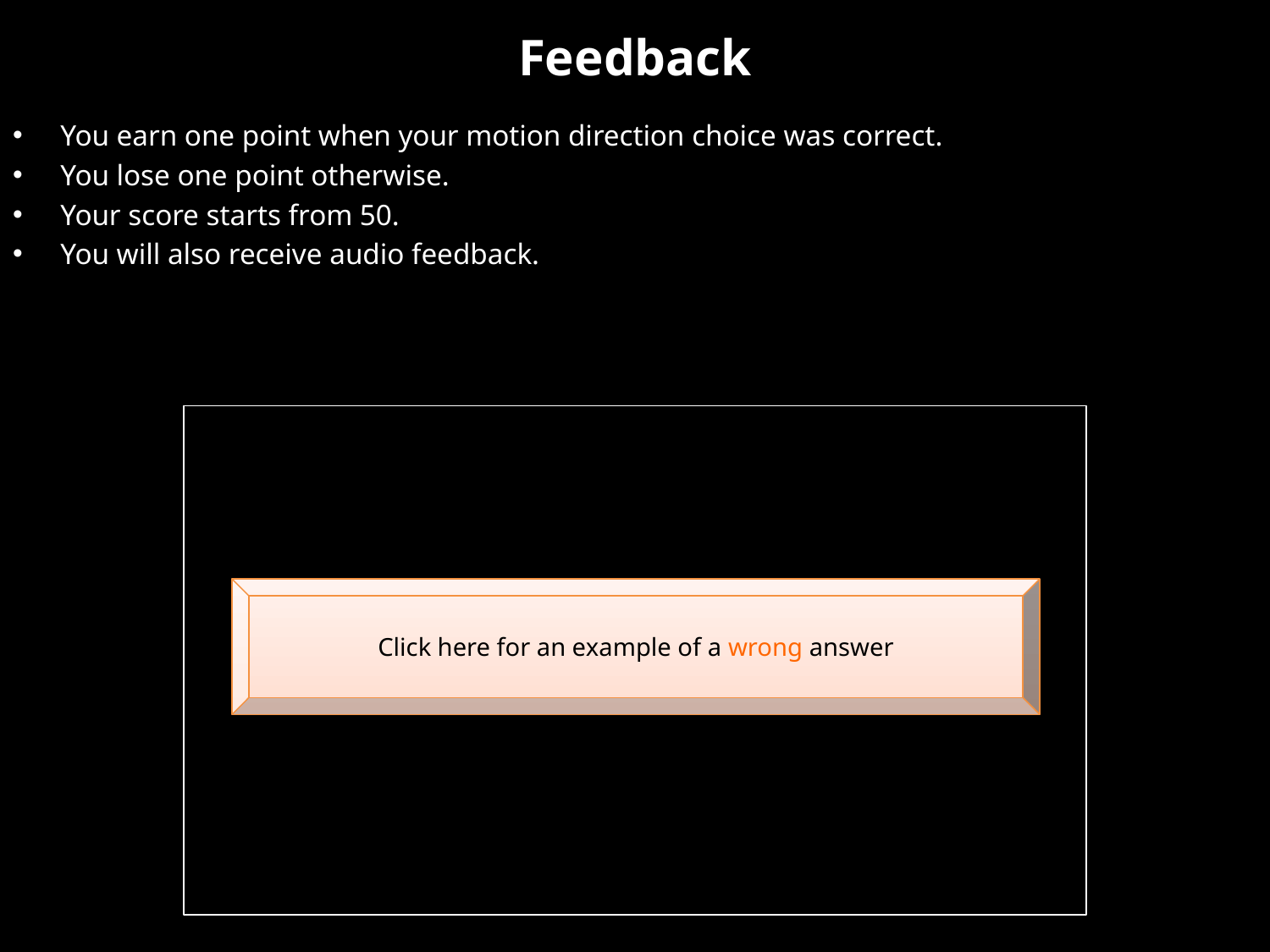

# Feedback
You earn one point when your motion direction choice was correct.
You lose one point otherwise.
Your score starts from 50.
You will also receive audio feedback.
Click here for an example of a wrong answer

## Slide 18
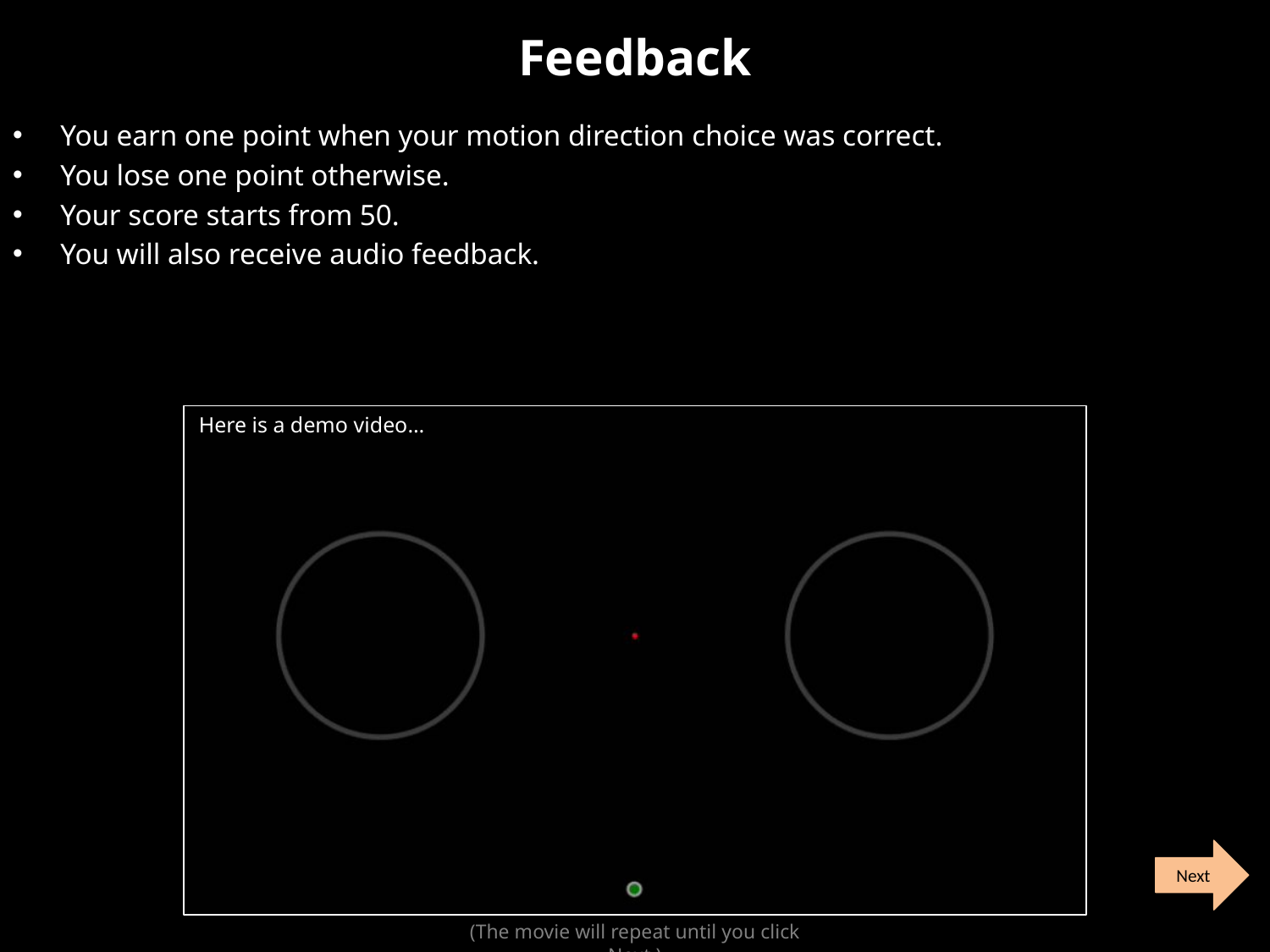

# Feedback
You earn one point when your motion direction choice was correct.
You lose one point otherwise.
Your score starts from 50.
You will also receive audio feedback.
Here is a demo video…

## Slide 19
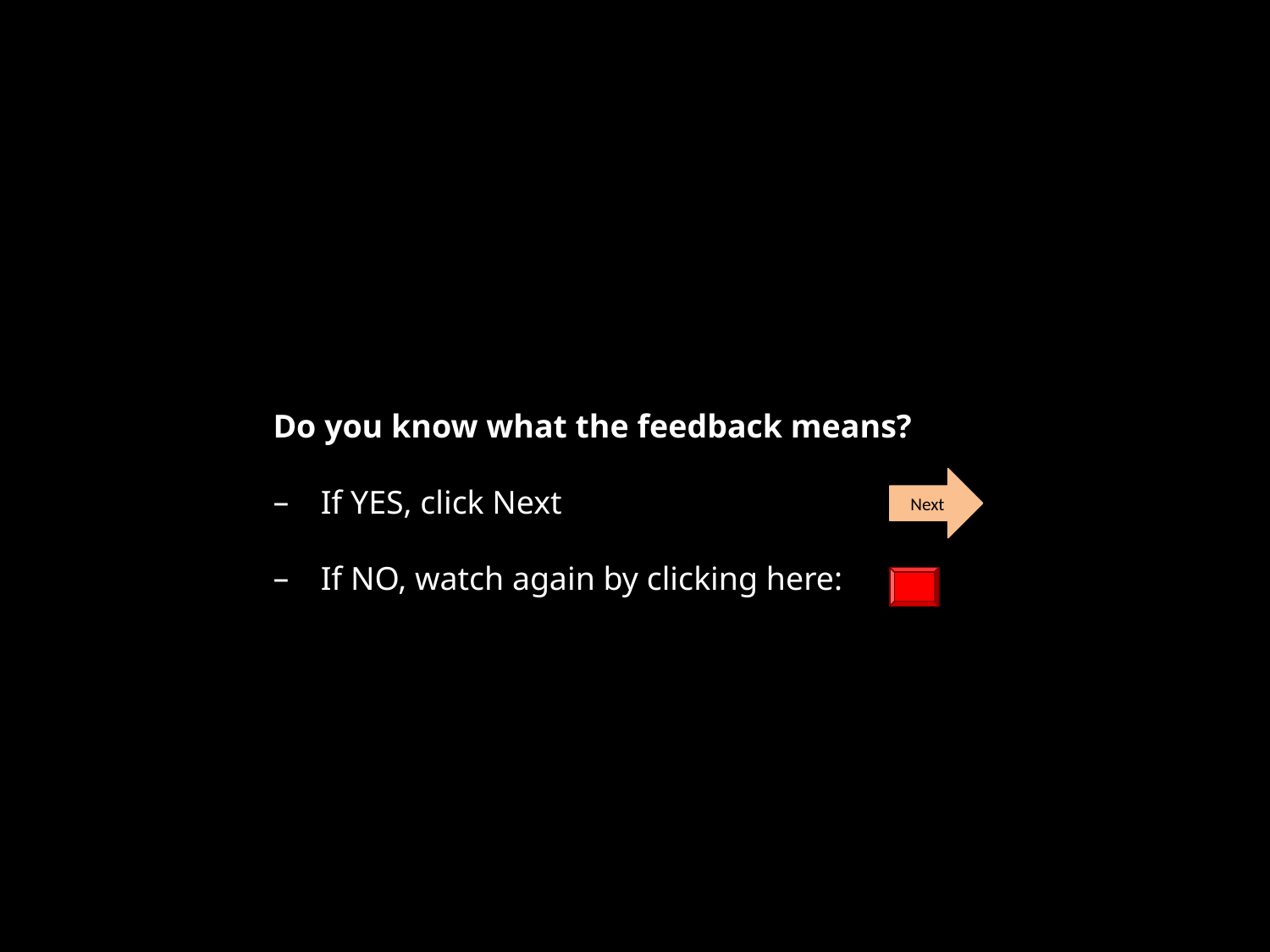

# Do you know what the feedback means?
Next
If YES, click Next
If NO, watch again by clicking here:

## Slide 20
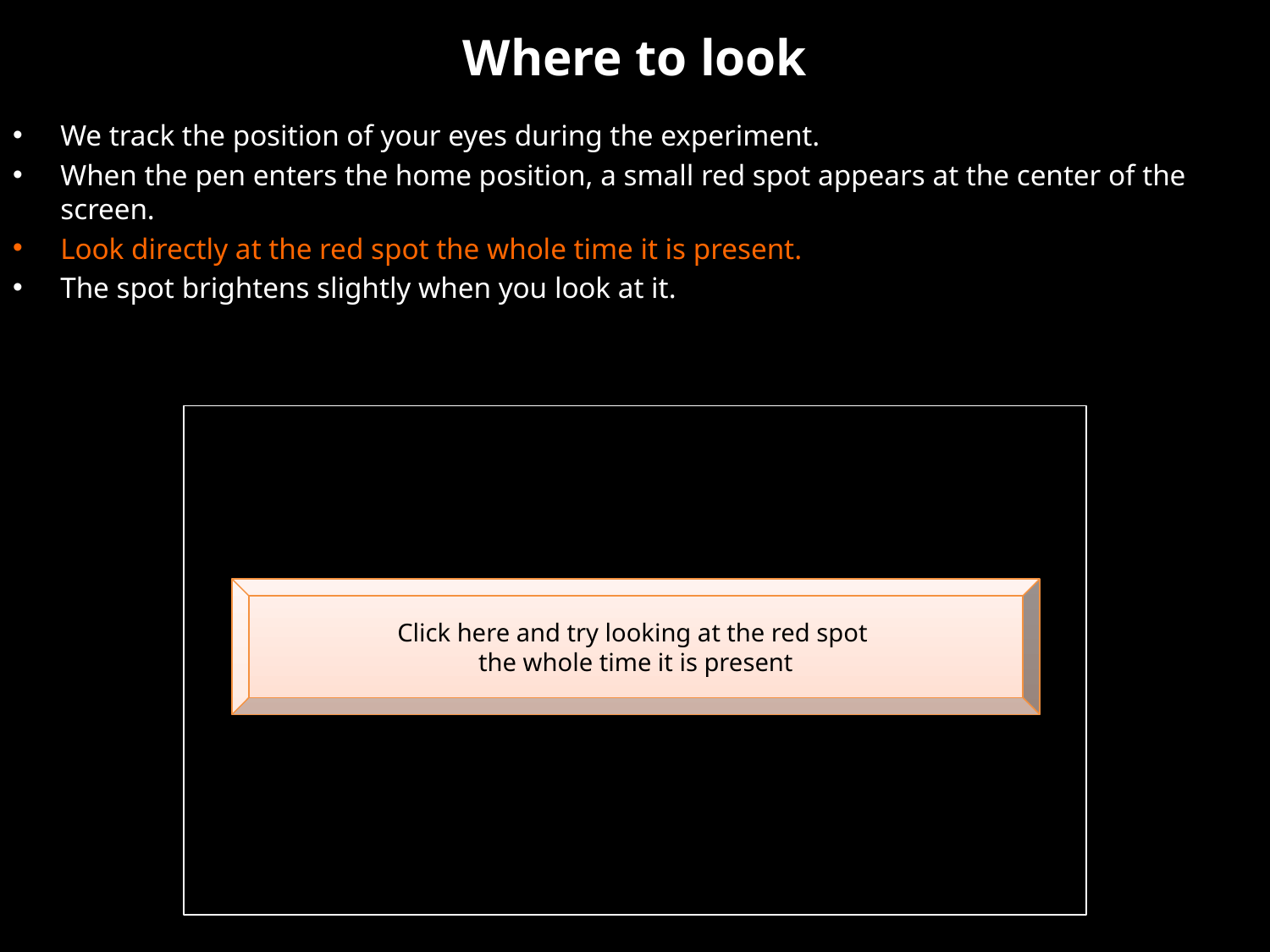

# Where to look
We track the position of your eyes during the experiment.
When the pen enters the home position, a small red spot appears at the center of the screen.
Look directly at the red spot the whole time it is present.
The spot brightens slightly when you look at it.
Click here and try looking at the red spot
the whole time it is present

## Slide 21
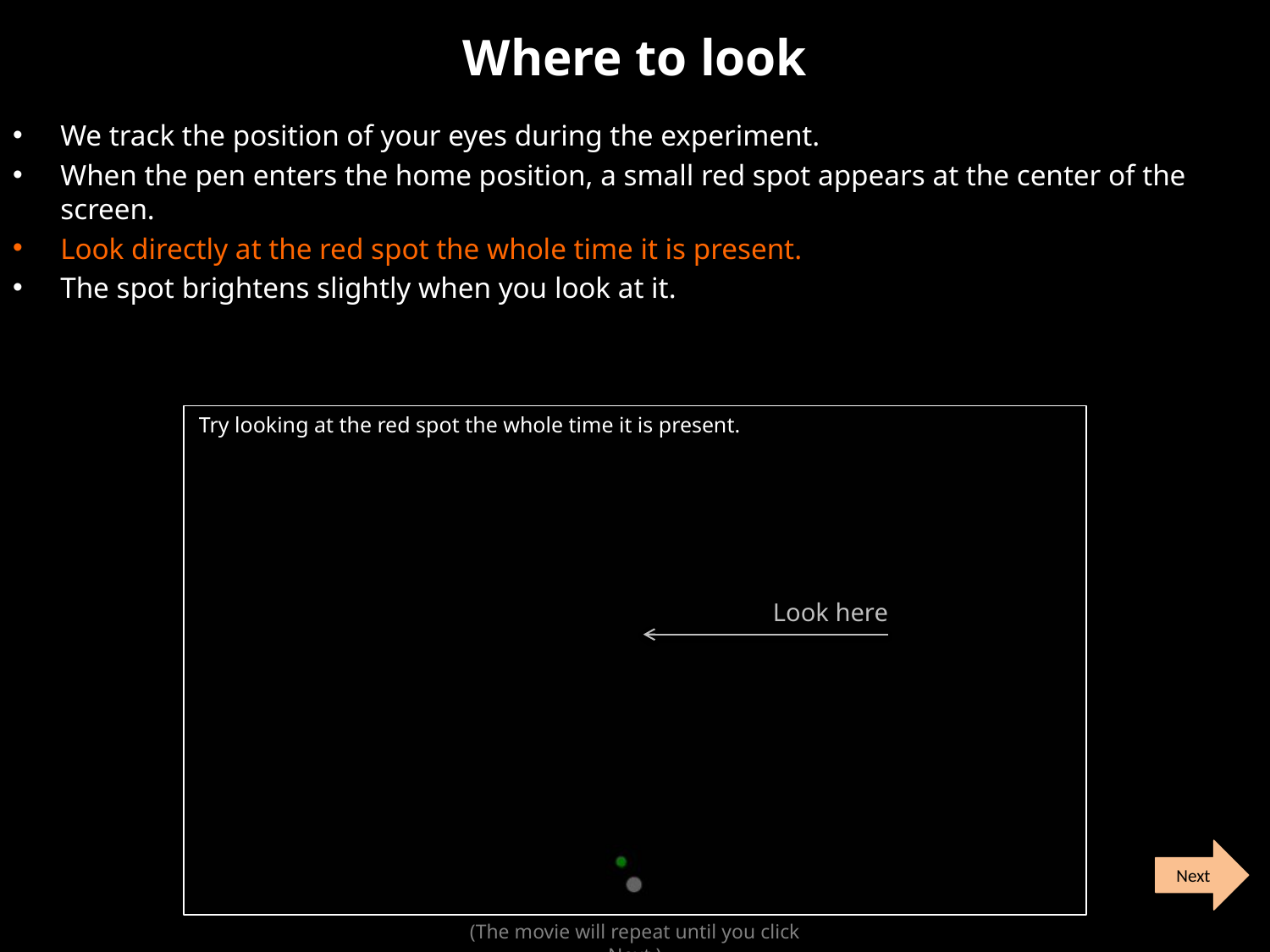

# Where to look
We track the position of your eyes during the experiment.
When the pen enters the home position, a small red spot appears at the center of the screen.
Look directly at the red spot the whole time it is present.
The spot brightens slightly when you look at it.
Try looking at the red spot the whole time it is present.
Look here

## Slide 22
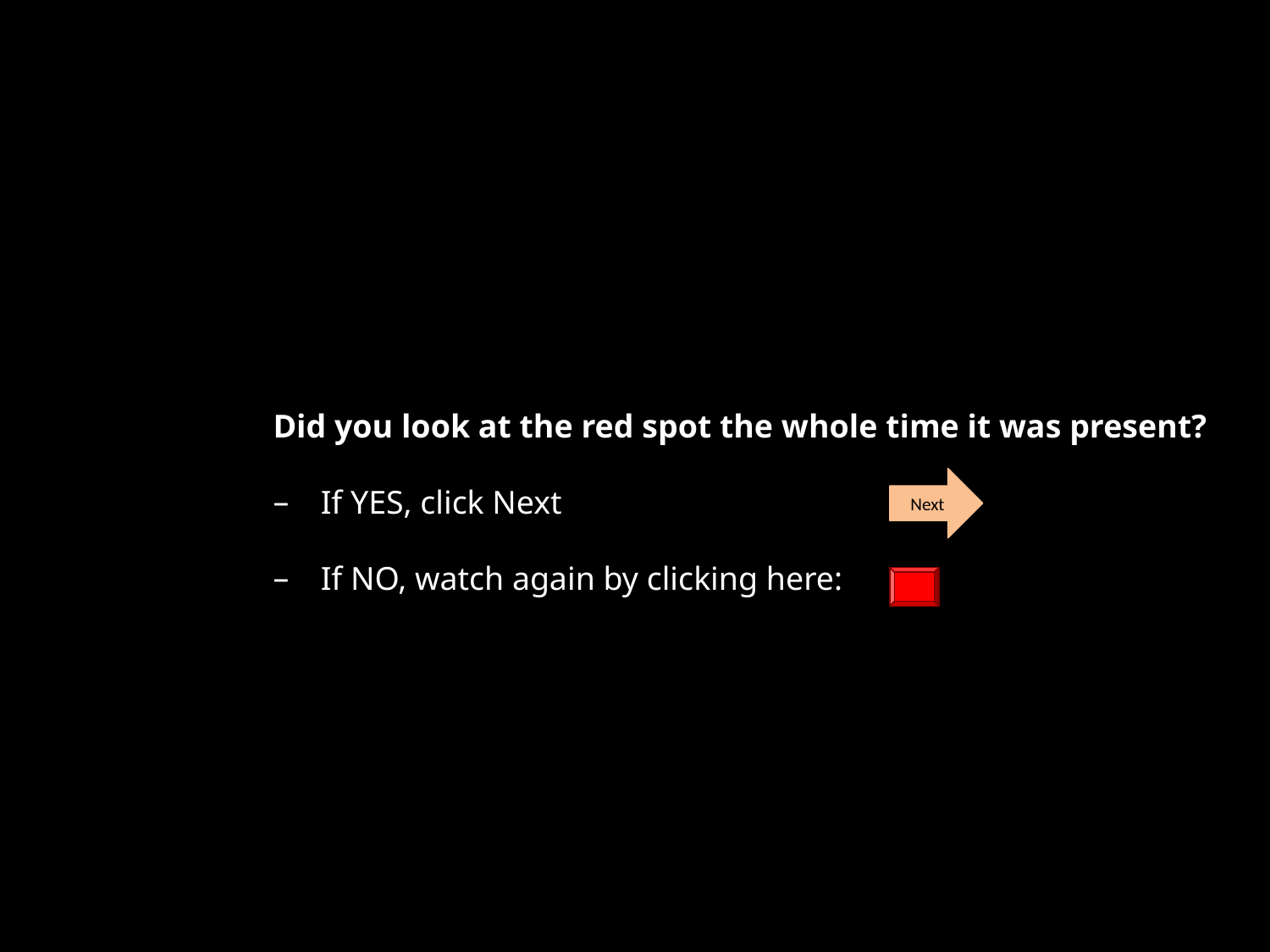

# Did you look at the red spot the whole time it was present?
Next
If YES, click Next
If NO, watch again by clicking here:

## Slide 23
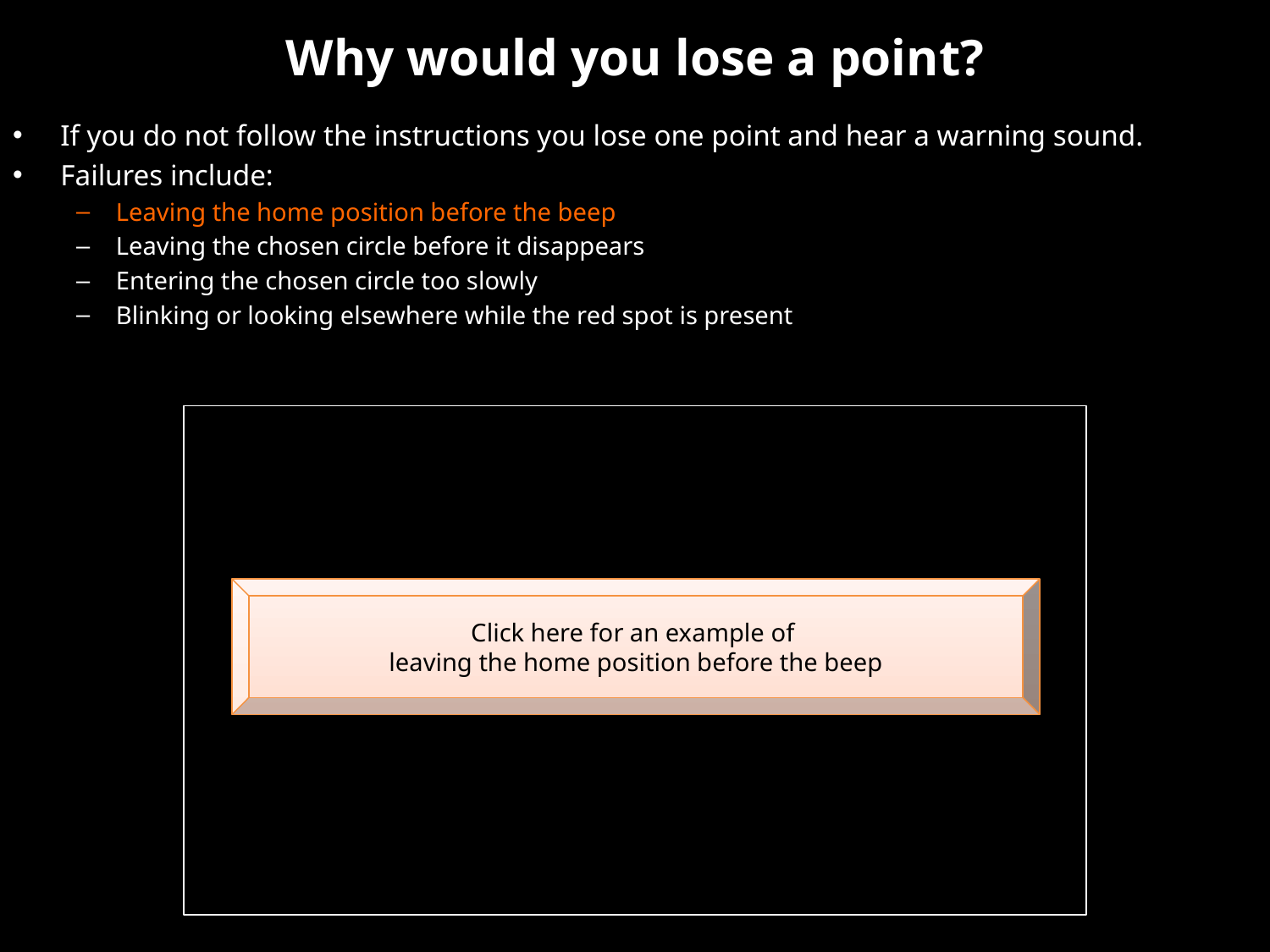

# Why would you lose a point?
If you do not follow the instructions you lose one point and hear a warning sound.
Failures include:
Leaving the home position before the beep
Leaving the chosen circle before it disappears
Entering the chosen circle too slowly
Blinking or looking elsewhere while the red spot is present
Click here for an example of
leaving the home position before the beep

## Slide 24
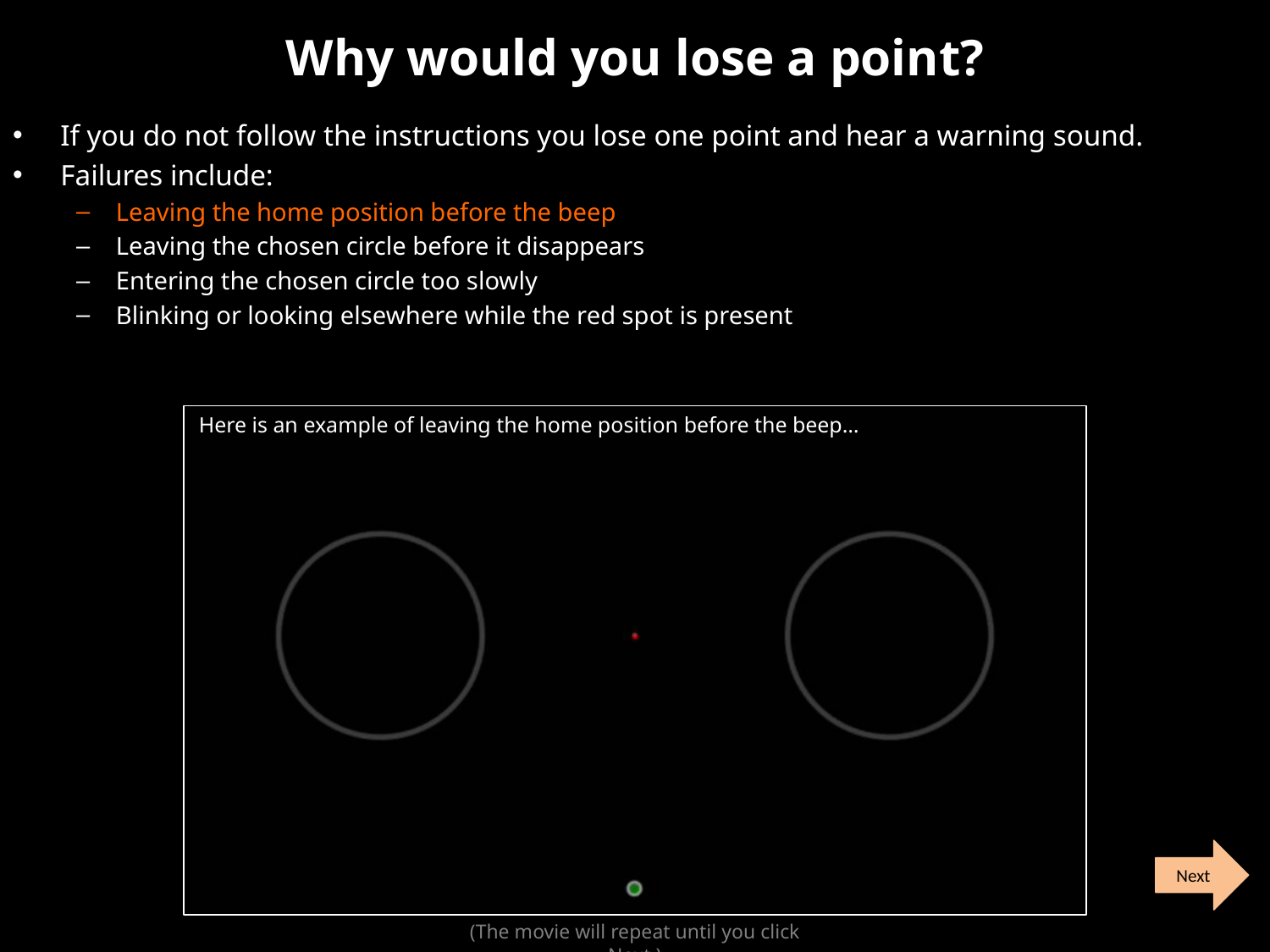

# Why would you lose a point?
If you do not follow the instructions you lose one point and hear a warning sound.
Failures include:
Leaving the home position before the beep
Leaving the chosen circle before it disappears
Entering the chosen circle too slowly
Blinking or looking elsewhere while the red spot is present
Here is an example of leaving the home position before the beep…

## Slide 25
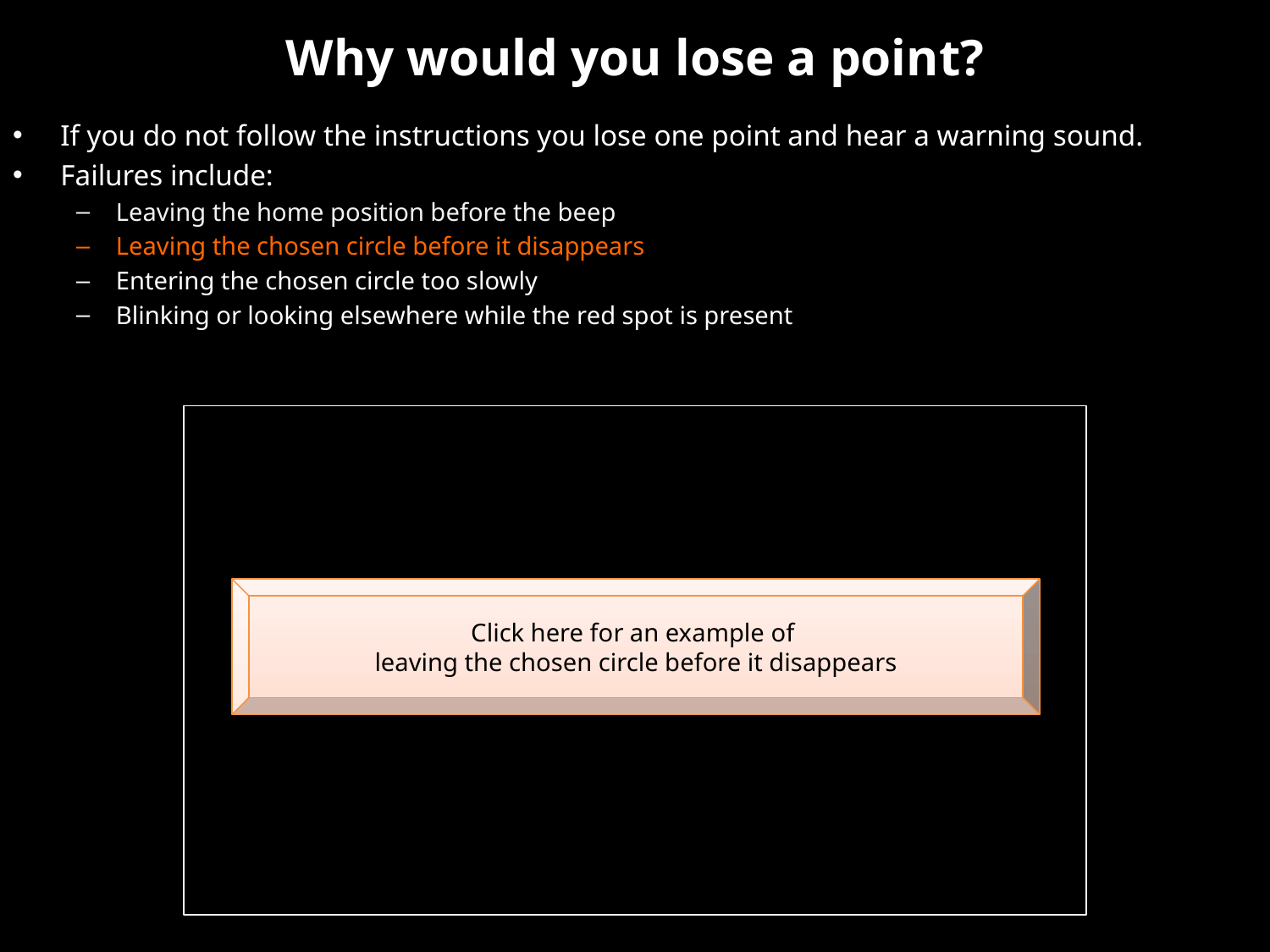

# Why would you lose a point?
If you do not follow the instructions you lose one point and hear a warning sound.
Failures include:
Leaving the home position before the beep
Leaving the chosen circle before it disappears
Entering the chosen circle too slowly
Blinking or looking elsewhere while the red spot is present
Click here for an example of
leaving the chosen circle before it disappears

## Slide 26
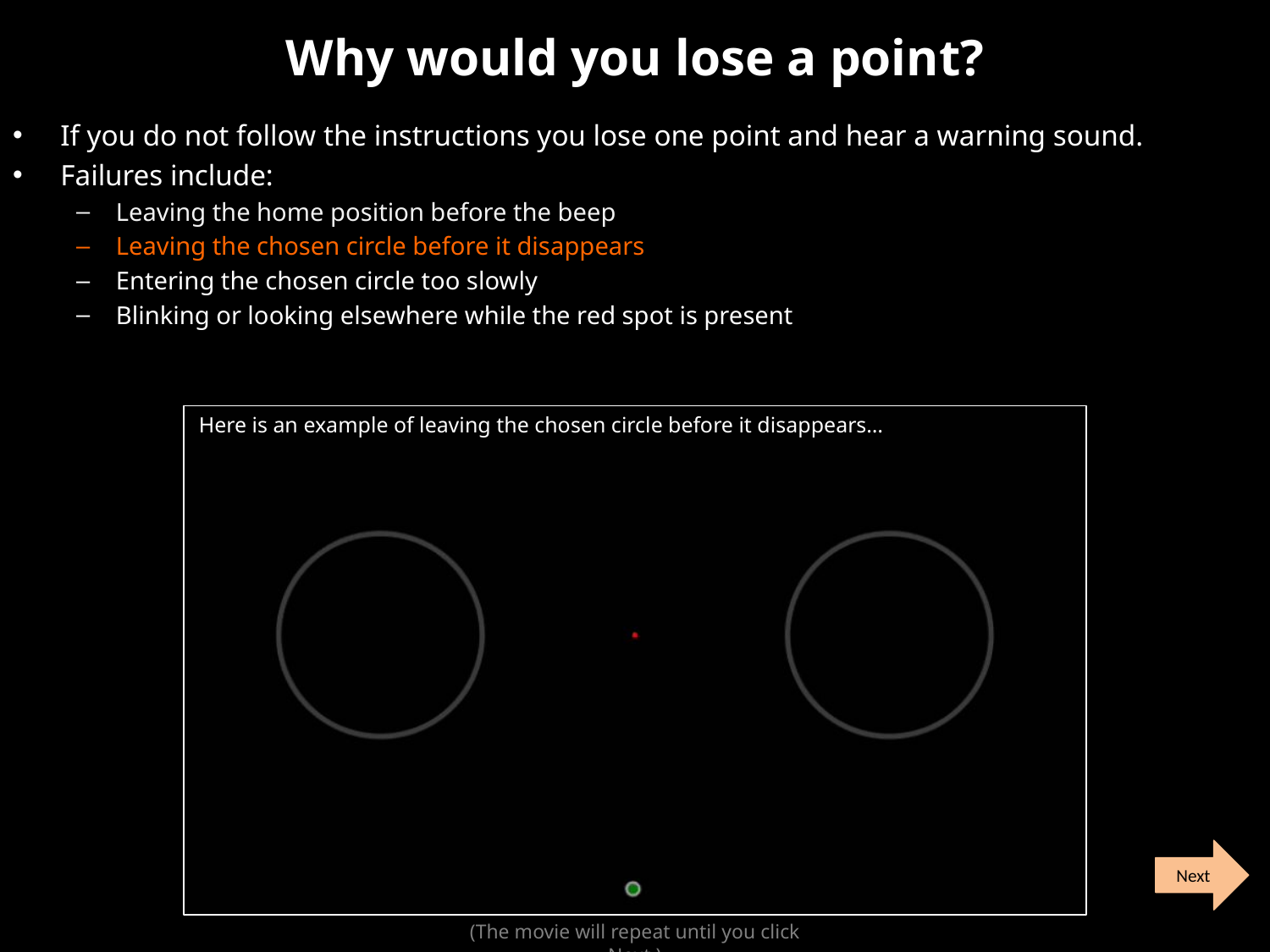

# Why would you lose a point?
If you do not follow the instructions you lose one point and hear a warning sound.
Failures include:
Leaving the home position before the beep
Leaving the chosen circle before it disappears
Entering the chosen circle too slowly
Blinking or looking elsewhere while the red spot is present
Here is an example of leaving the chosen circle before it disappears…

## Slide 27
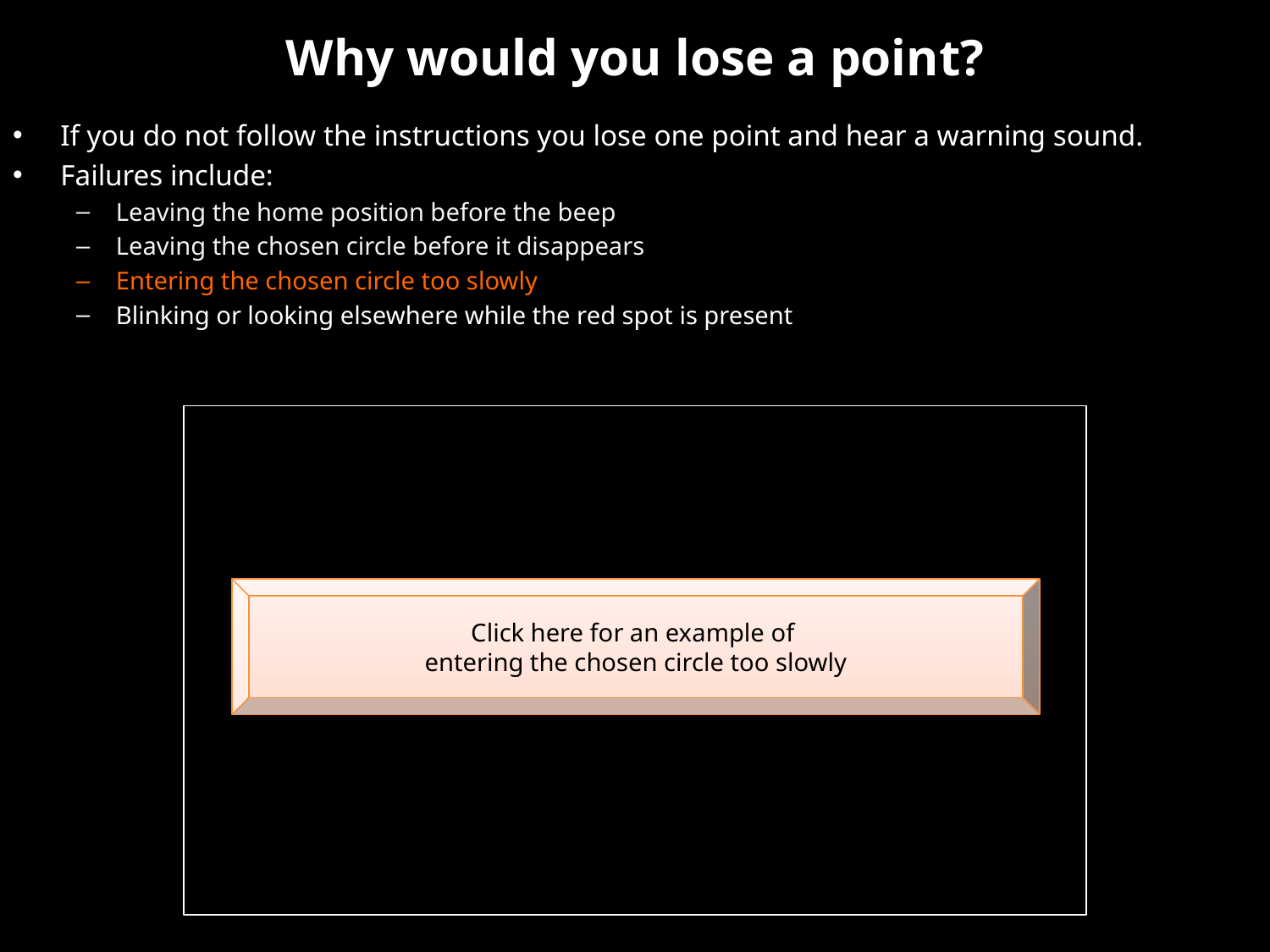

# Why would you lose a point?
If you do not follow the instructions you lose one point and hear a warning sound.
Failures include:
Leaving the home position before the beep
Leaving the chosen circle before it disappears
Entering the chosen circle too slowly
Blinking or looking elsewhere while the red spot is present
Click here for an example of
entering the chosen circle too slowly

## Slide 28
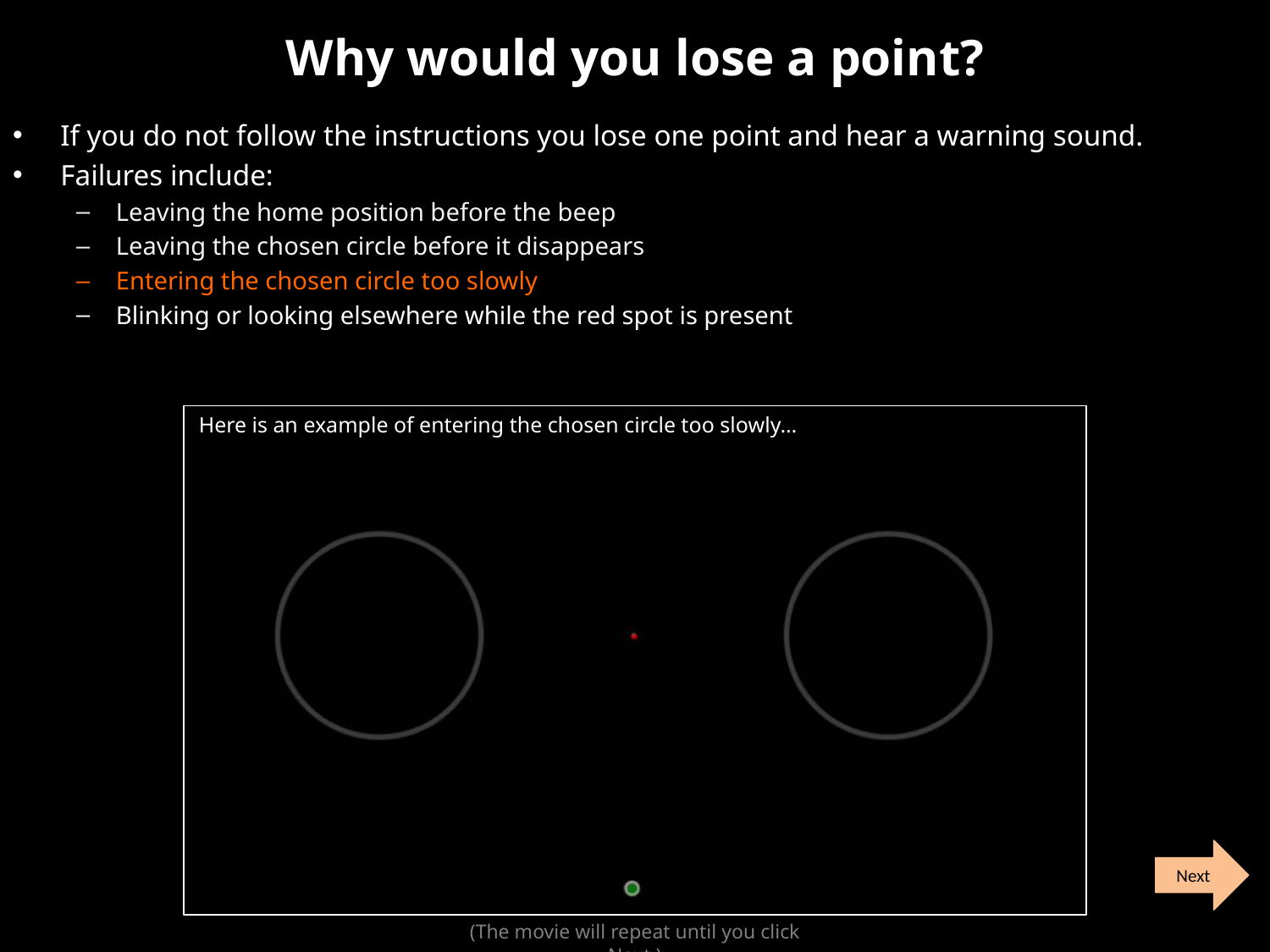

# Why would you lose a point?
If you do not follow the instructions you lose one point and hear a warning sound.
Failures include:
Leaving the home position before the beep
Leaving the chosen circle before it disappears
Entering the chosen circle too slowly
Blinking or looking elsewhere while the red spot is present
Here is an example of entering the chosen circle too slowly…

## Slide 29
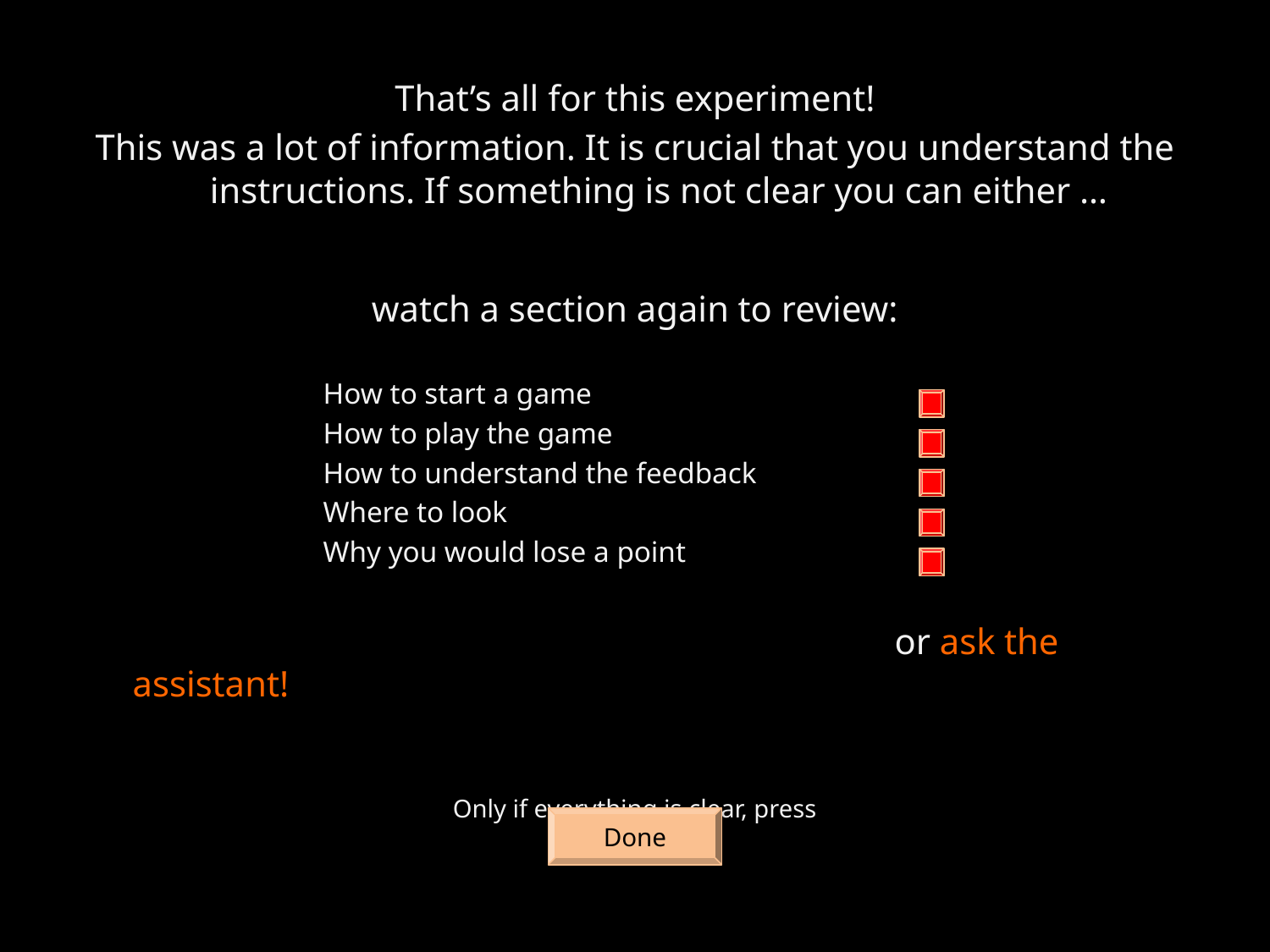

That’s all for this experiment!
This was a lot of information. It is crucial that you understand the instructions. If something is not clear you can either …
watch a section again to review:
How to start a game
How to play the game
How to understand the feedback
Where to look
Why you would lose a point
						or ask the assistant!
Only if everything is clear, press
Done

## Slide 30
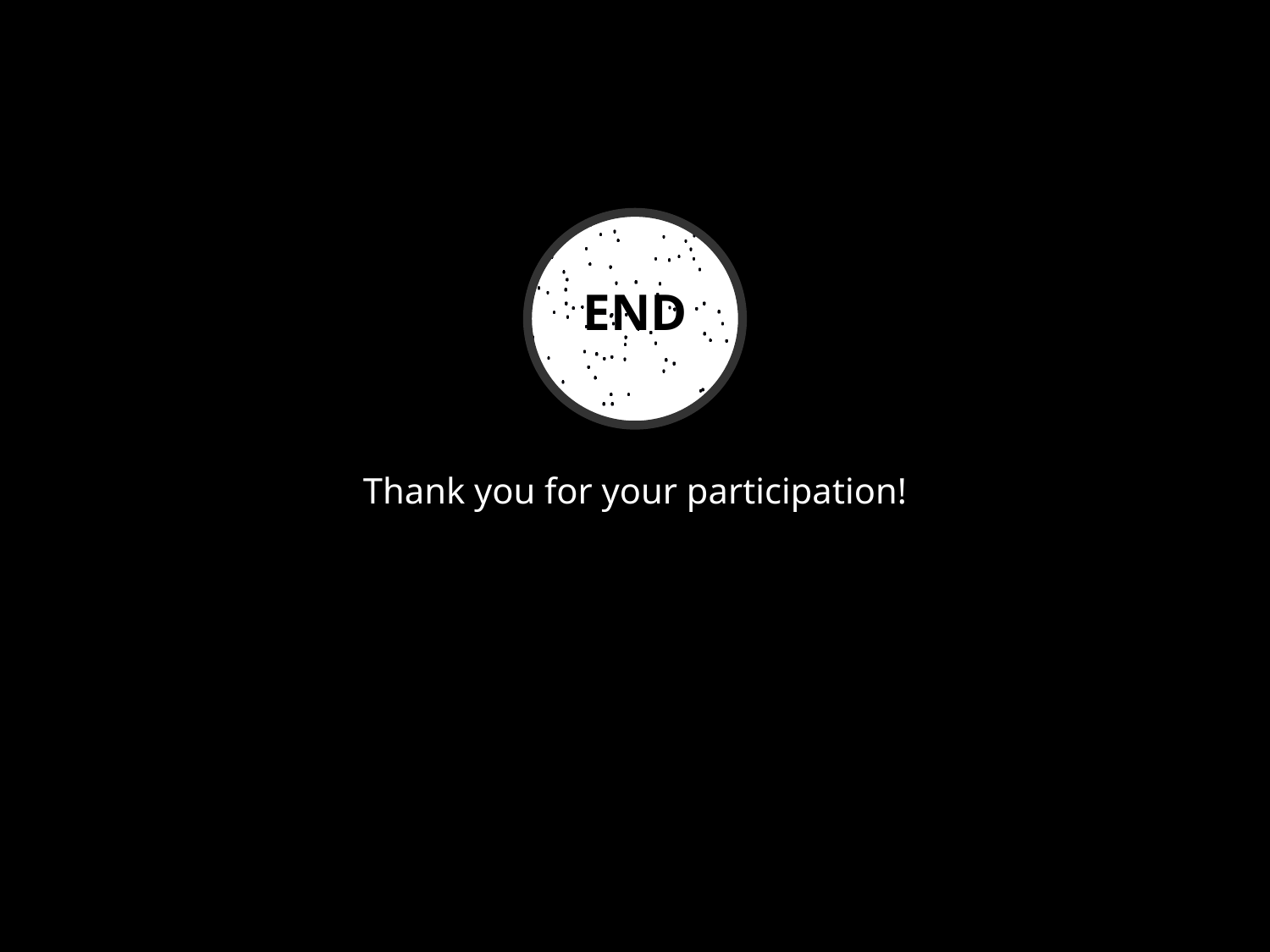

# END
Thank you for your participation!

## Slide 31
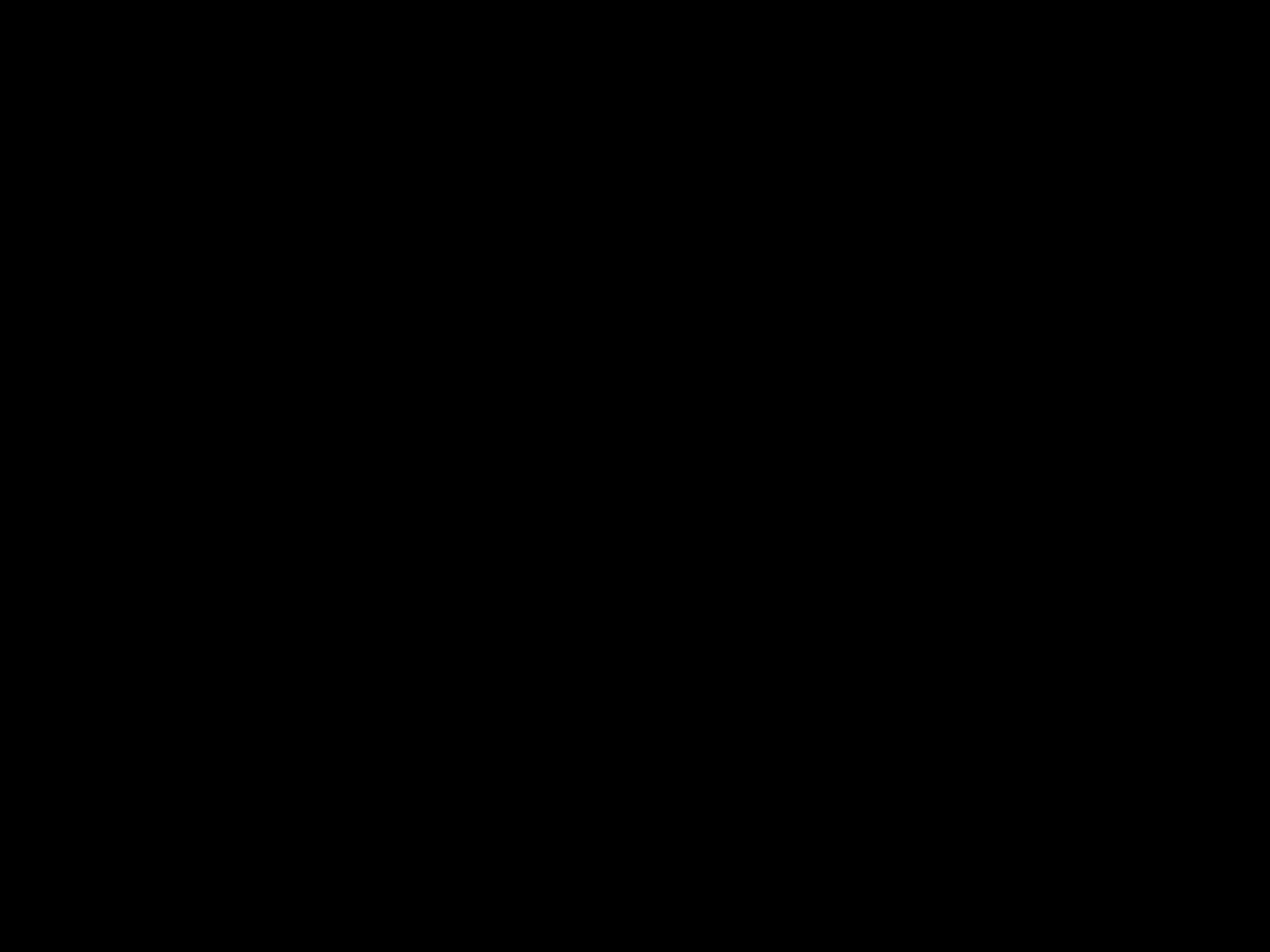

#

## Slide 32
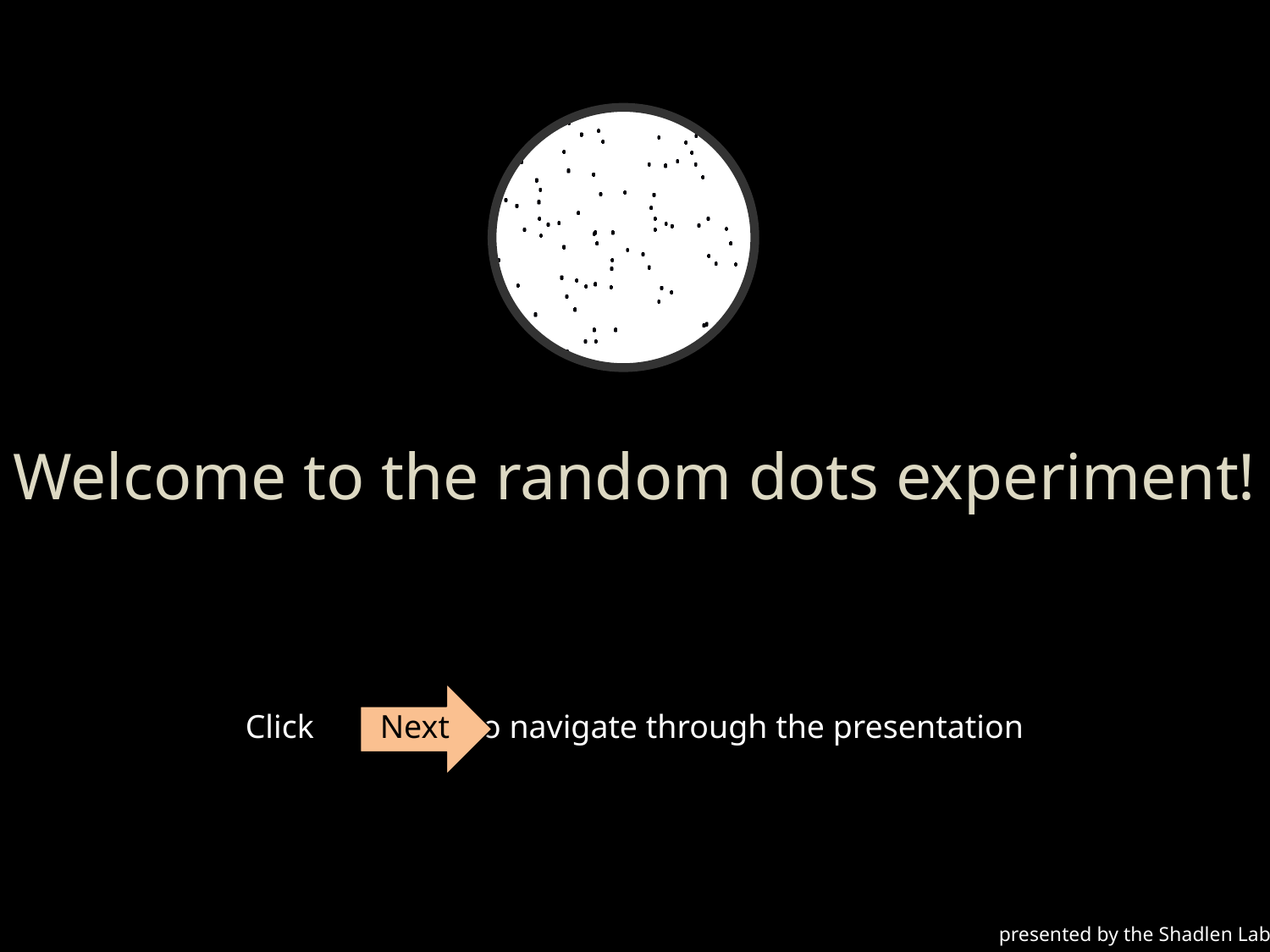

# Welcome to the random dots experiment!
Next
Click to navigate through the presentation
presented by the Shadlen Lab

## Slide 33
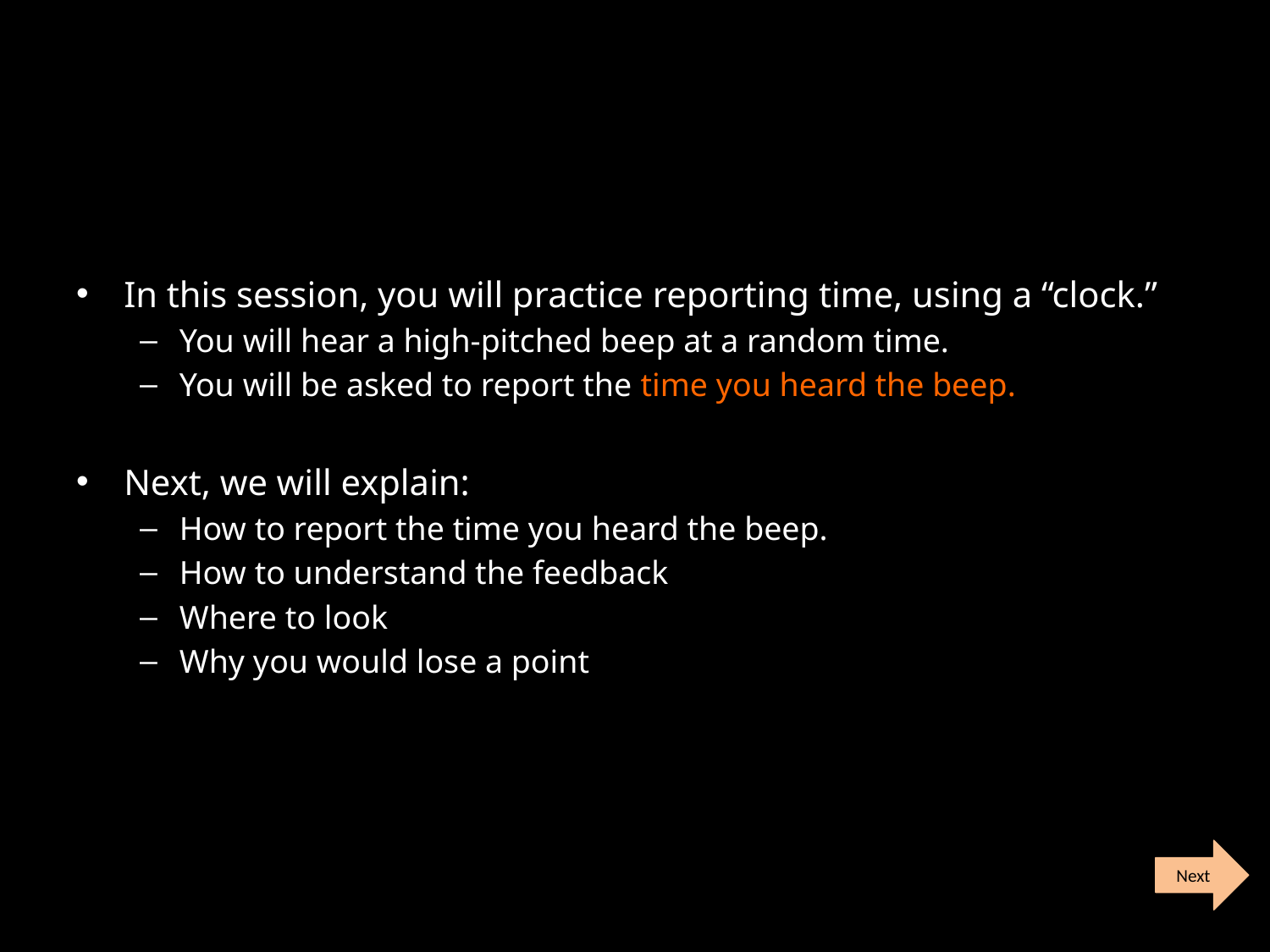

In this session, you will practice reporting time, using a “clock.”
You will hear a high-pitched beep at a random time.
You will be asked to report the time you heard the beep.
Next, we will explain:
How to report the time you heard the beep.
How to understand the feedback
Where to look
Why you would lose a point
Next

## Slide 34
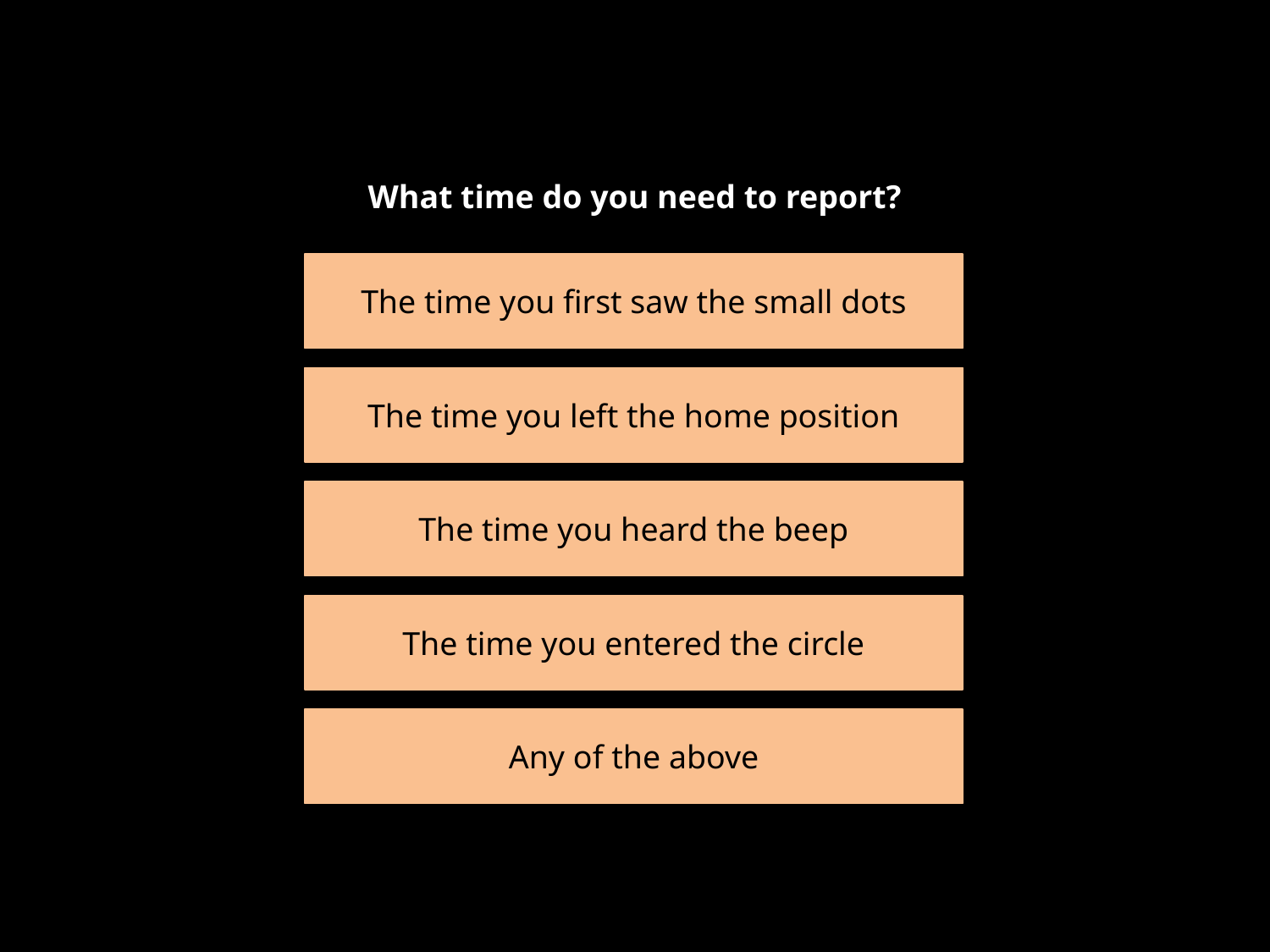

What time do you need to report?
The time you first saw the small dots
The time you left the home position
The time you heard the beep
The time you entered the circle
Any of the above

## Slide 35
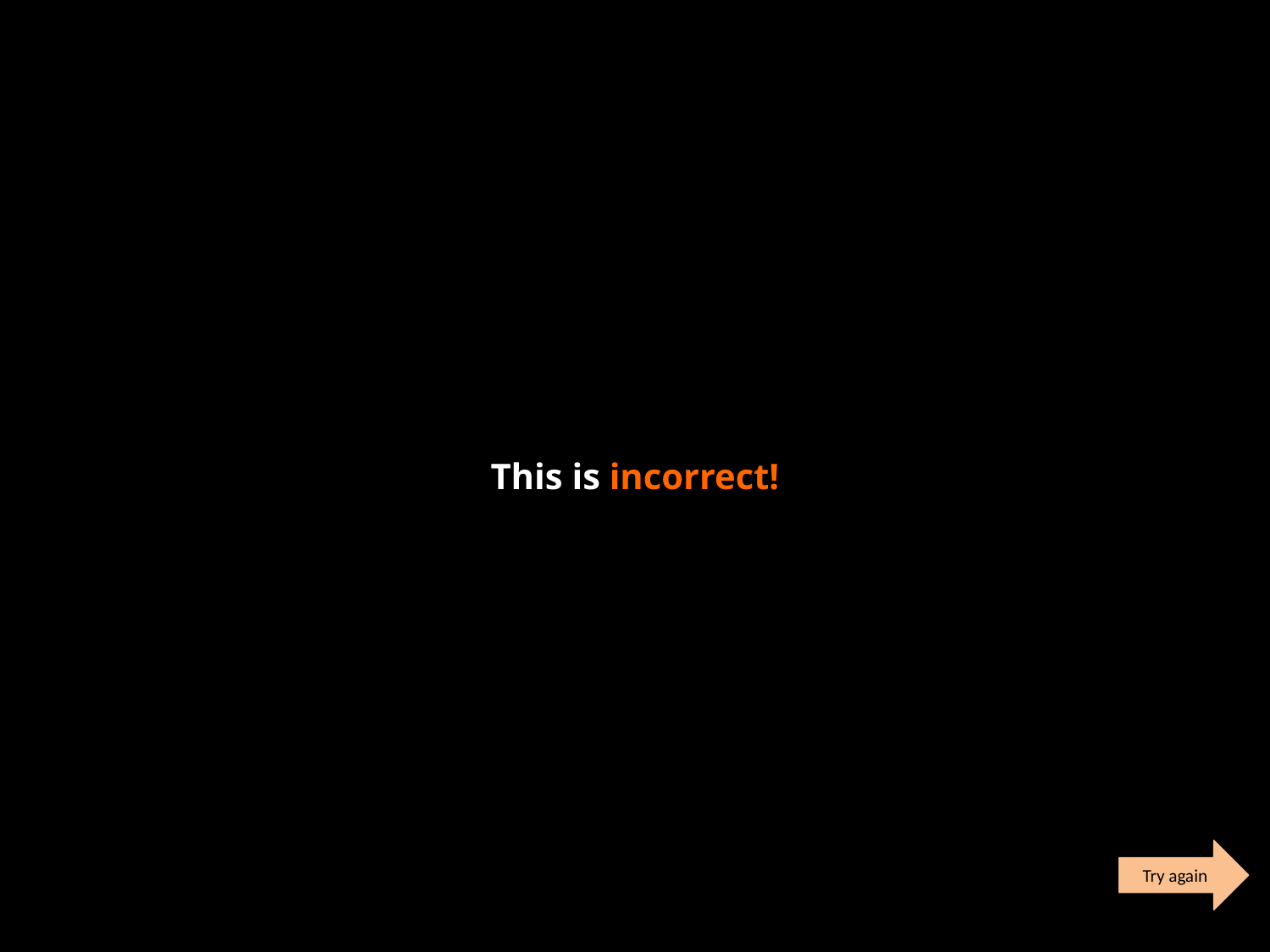

This is incorrect!
Try again

## Slide 36
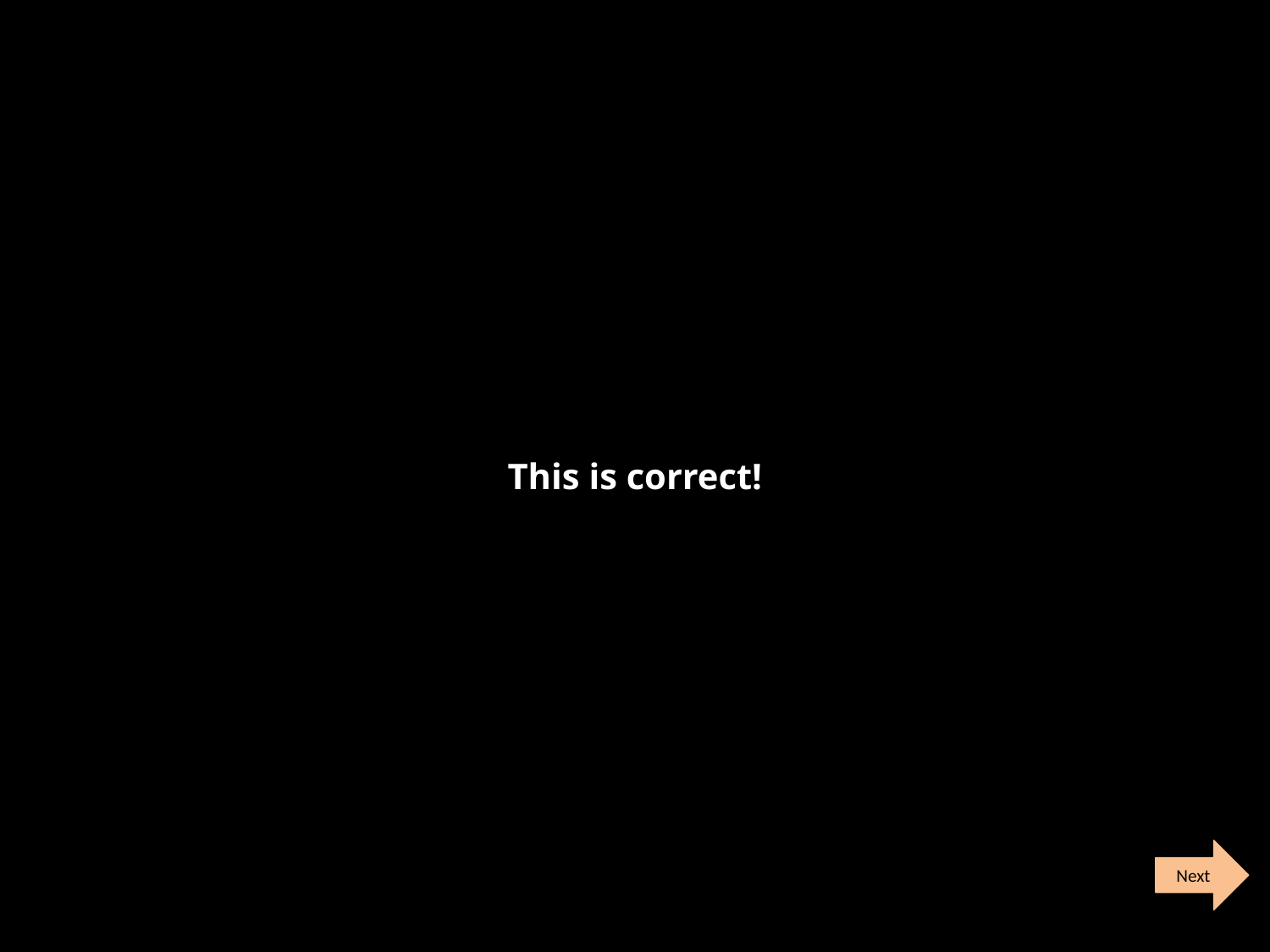

This is correct!
Next

## Slide 37
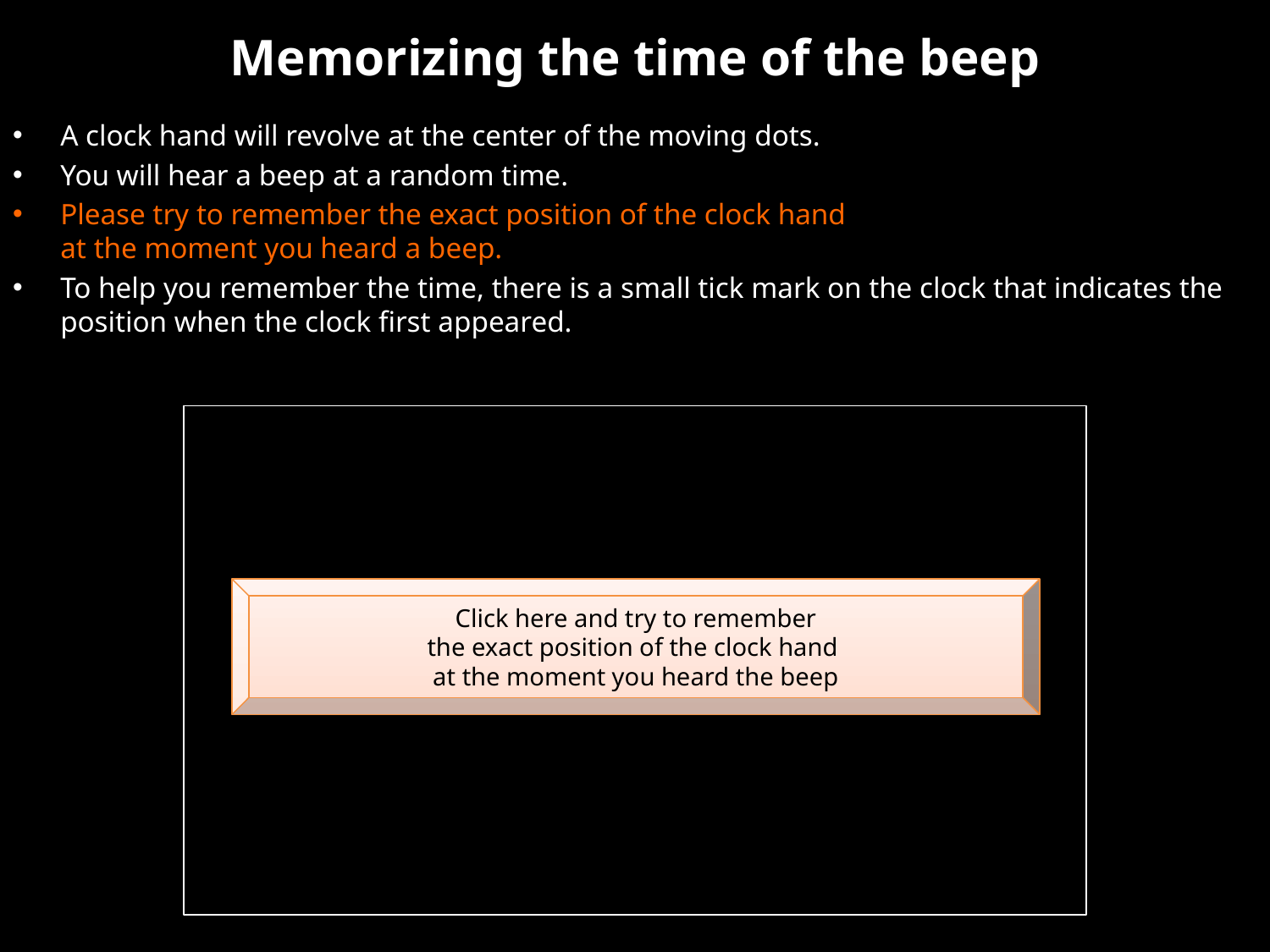

# Memorizing the time of the beep
A clock hand will revolve at the center of the moving dots.
You will hear a beep at a random time.
Please try to remember the exact position of the clock hand
at the moment you heard a beep.
To help you remember the time, there is a small tick mark on the clock that indicates the position when the clock first appeared.
Click here and try to remember
the exact position of the clock hand
at the moment you heard the beep

## Slide 38
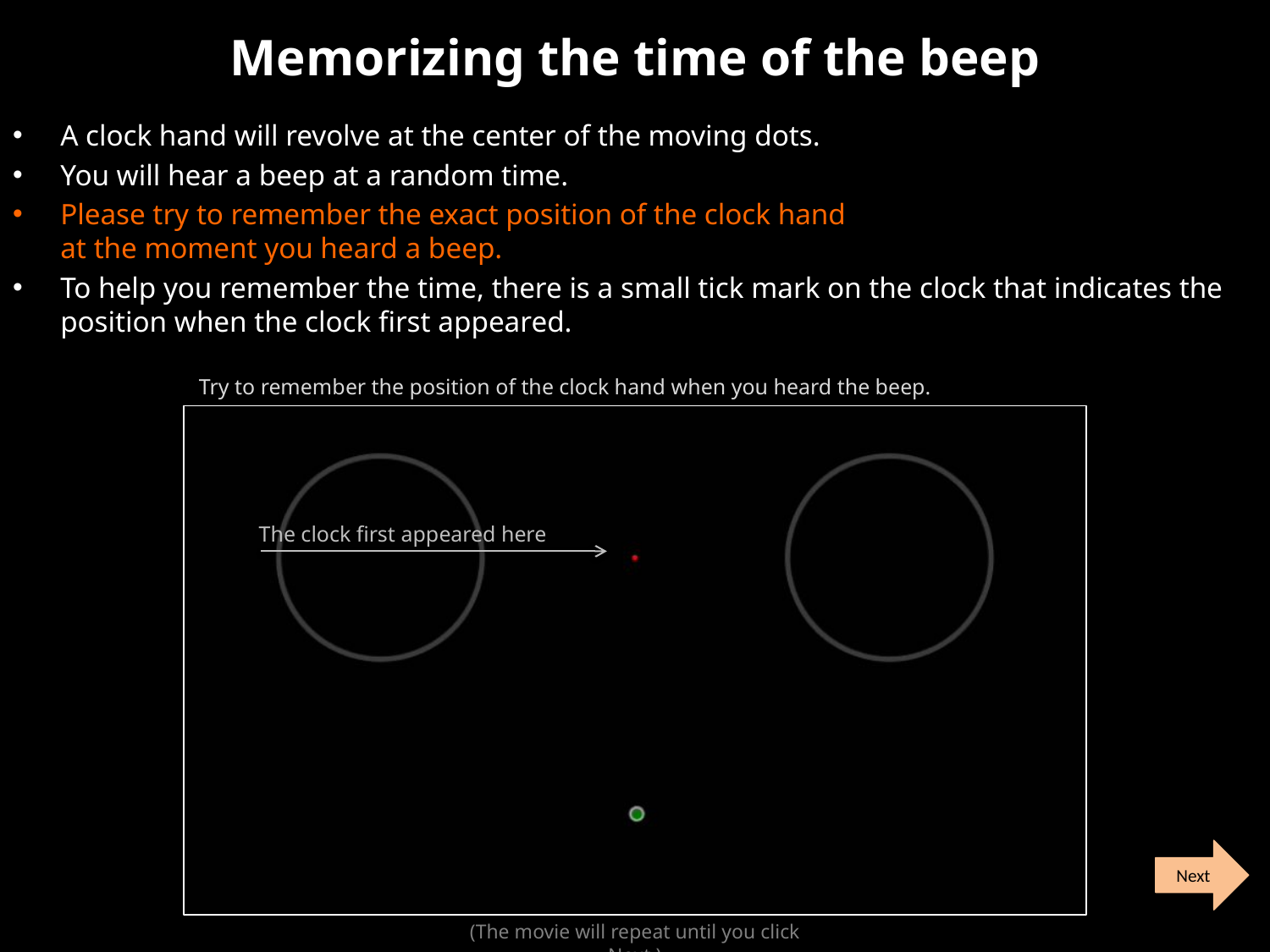

# Memorizing the time of the beep
A clock hand will revolve at the center of the moving dots.
You will hear a beep at a random time.
Please try to remember the exact position of the clock hand
at the moment you heard a beep.
To help you remember the time, there is a small tick mark on the clock that indicates the position when the clock first appeared.
Try to remember the position of the clock hand when you heard the beep.
The clock first appeared here

## Slide 39
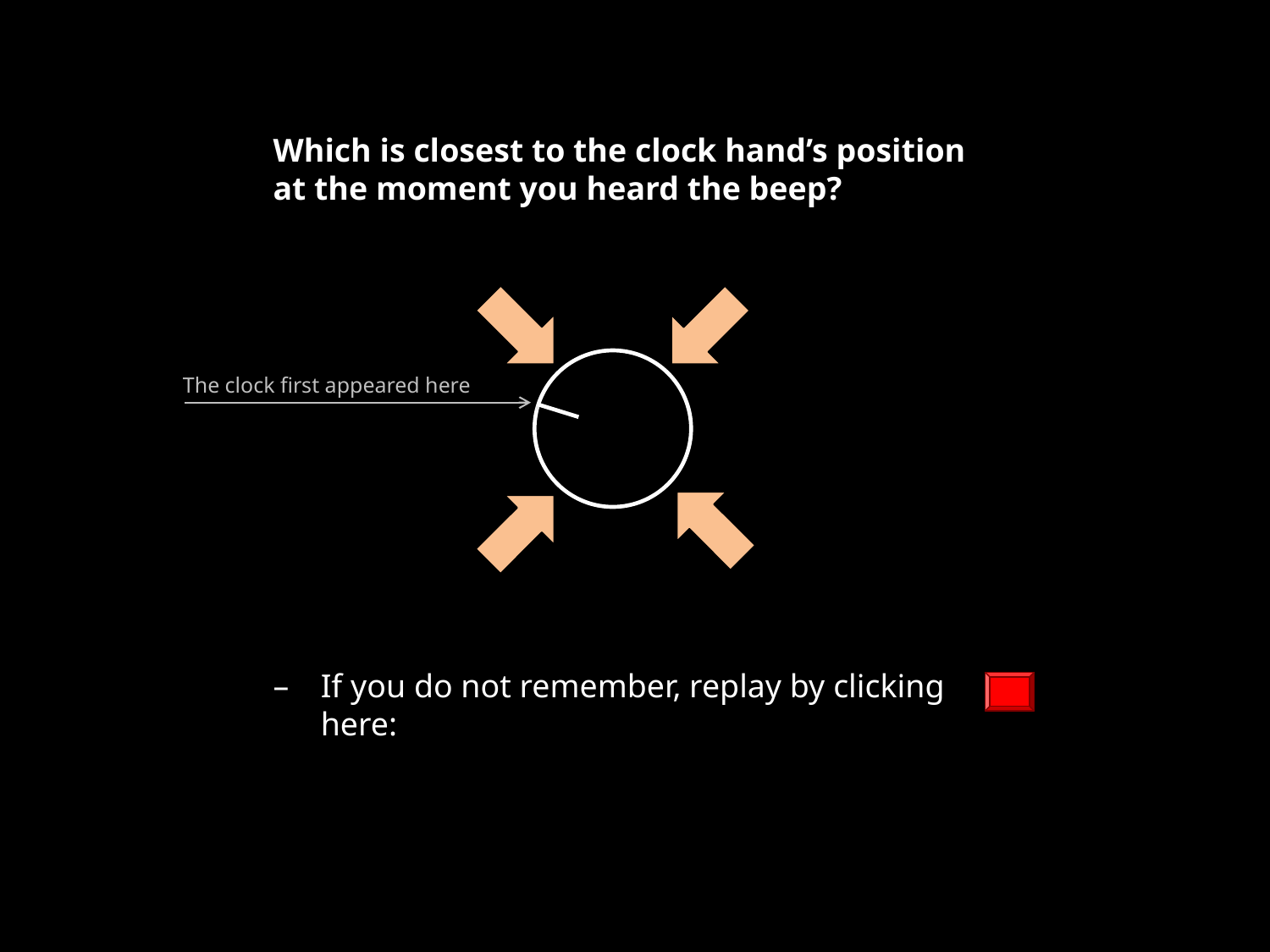

# Which is closest to the clock hand’s position at the moment you heard the beep?
The clock first appeared here
If you do not remember, replay by clicking here:

## Slide 40
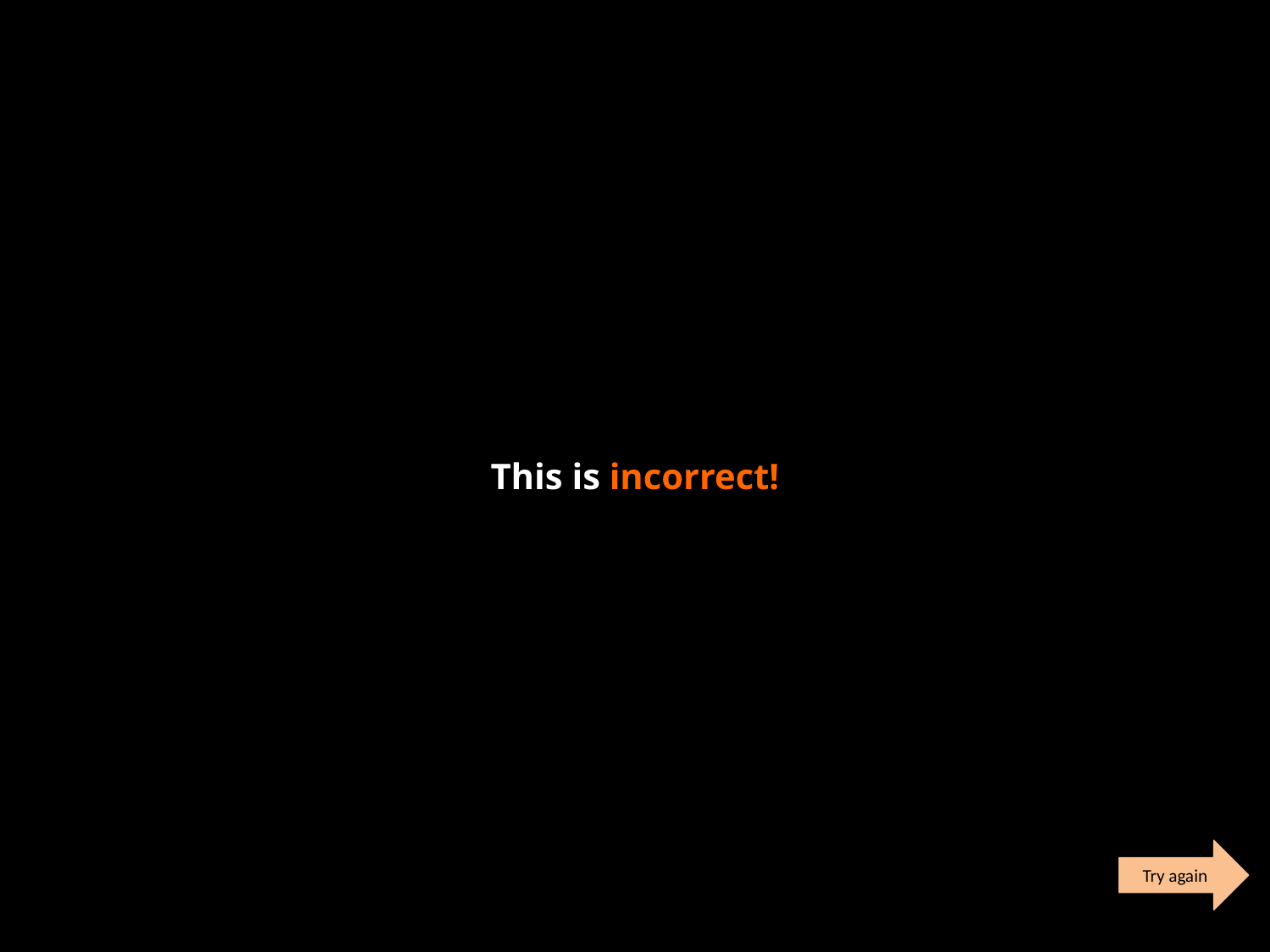

This is incorrect!
Try again

## Slide 41
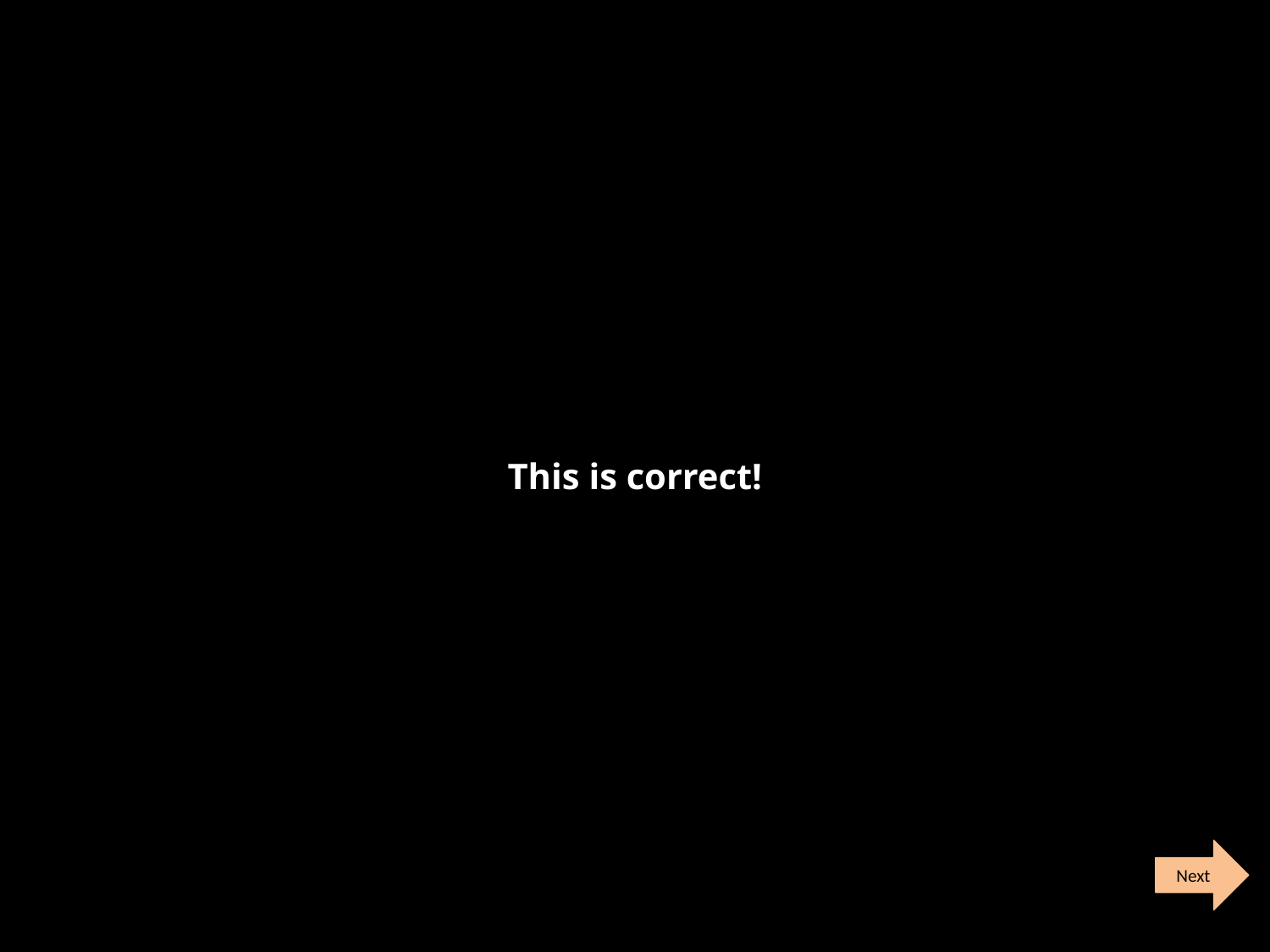

This is correct!
Next

## Slide 42
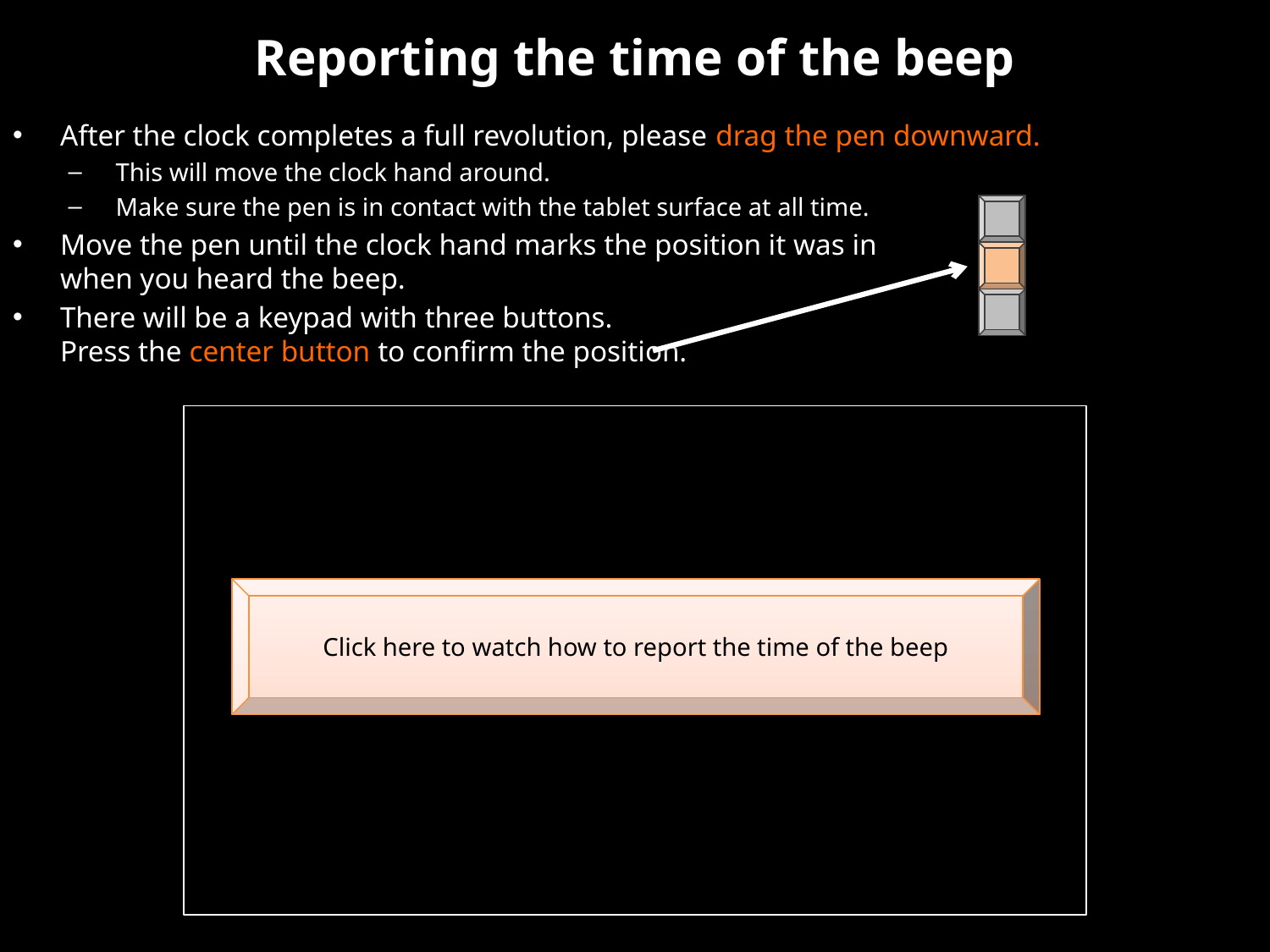

# Reporting the time of the beep
After the clock completes a full revolution, please drag the pen downward.
This will move the clock hand around.
Make sure the pen is in contact with the tablet surface at all time.
Move the pen until the clock hand marks the position it was in
when you heard the beep.
There will be a keypad with three buttons.
Press the center button to confirm the position.
Click here to watch how to report the time of the beep

## Slide 43
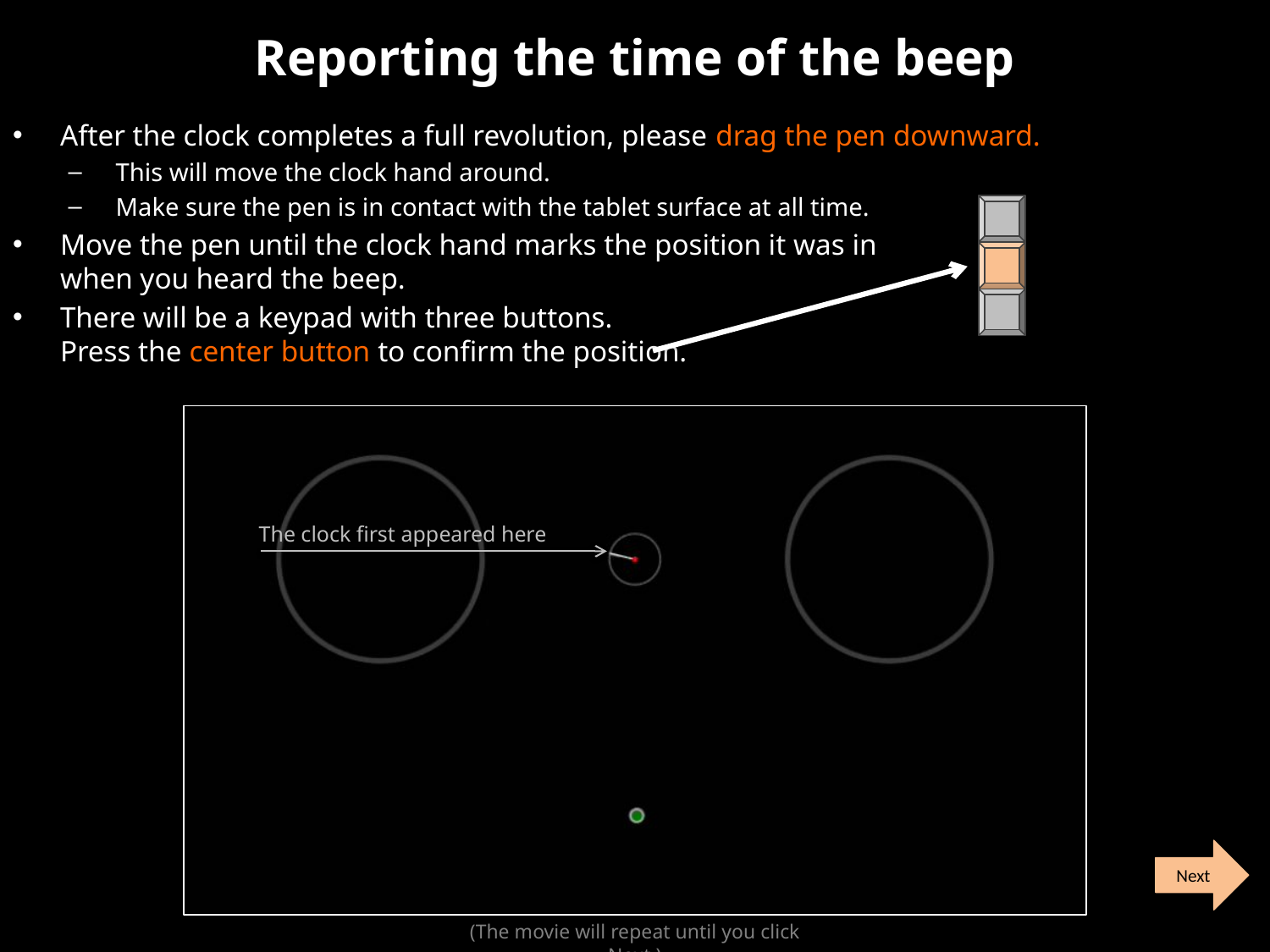

# Reporting the time of the beep
After the clock completes a full revolution, please drag the pen downward.
This will move the clock hand around.
Make sure the pen is in contact with the tablet surface at all time.
Move the pen until the clock hand marks the position it was in
when you heard the beep.
There will be a keypad with three buttons.
Press the center button to confirm the position.
The clock first appeared here

## Slide 44
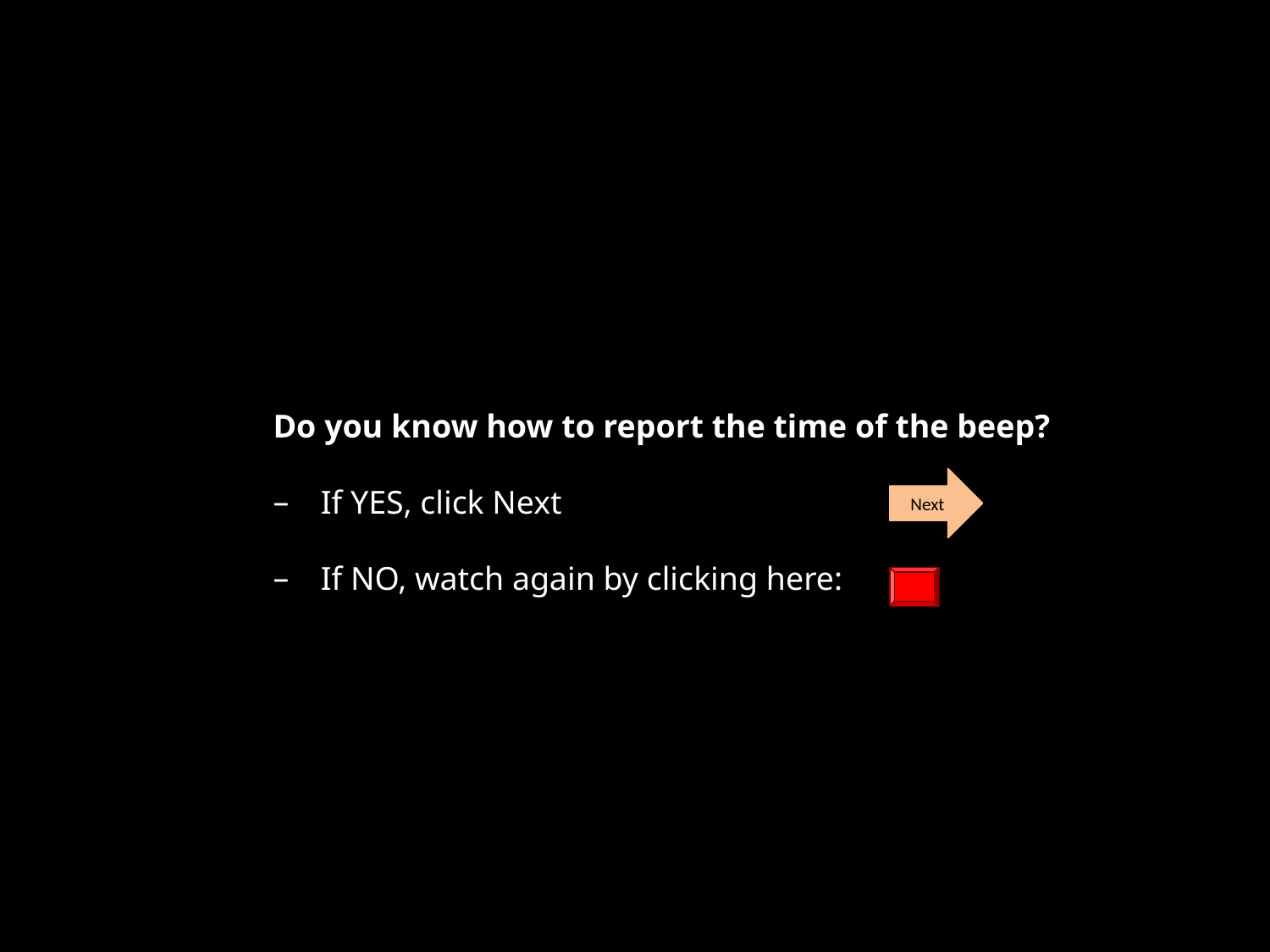

# Do you know how to report the time of the beep?
Next
If YES, click Next
If NO, watch again by clicking here:

## Slide 45
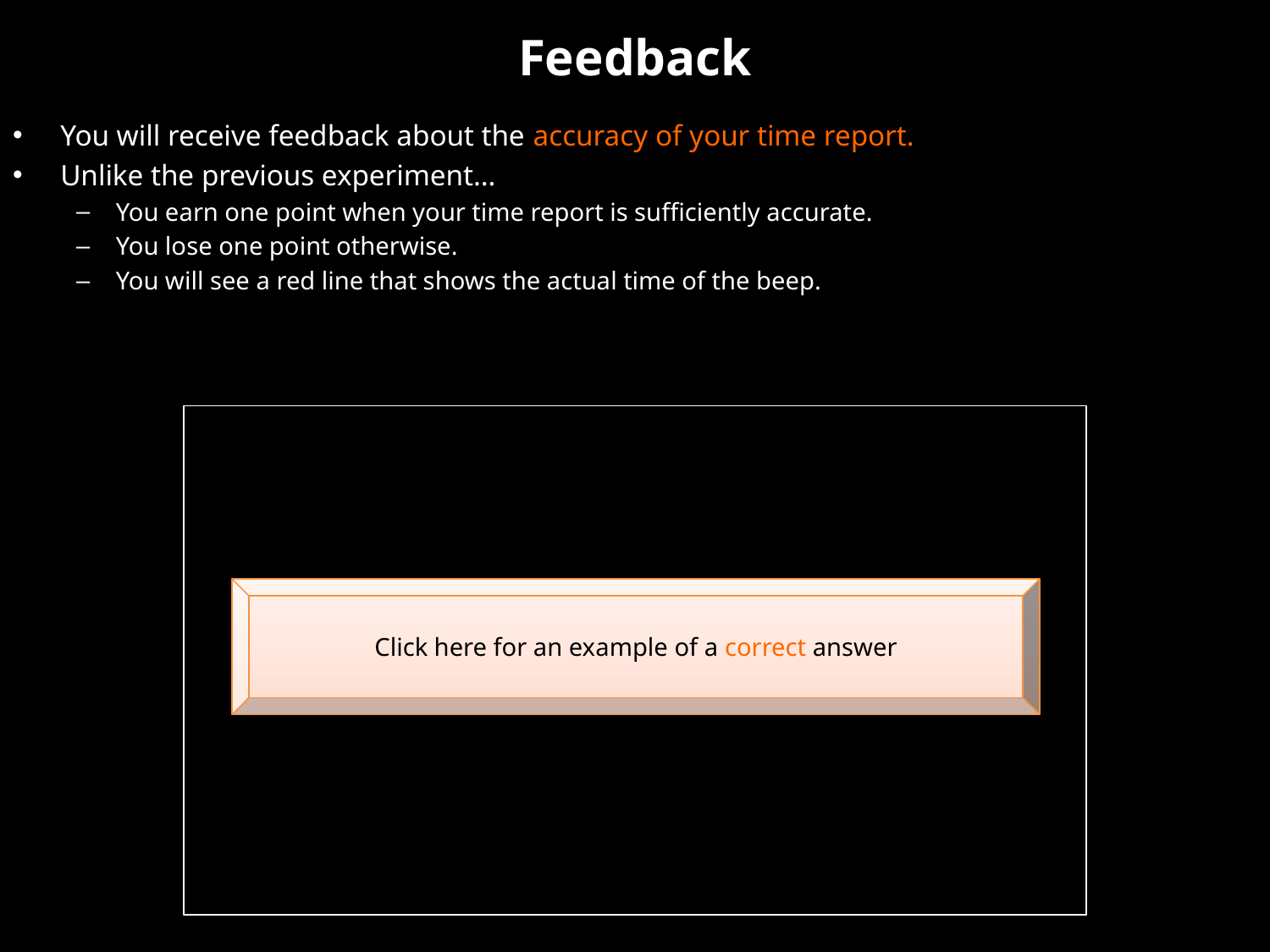

# Feedback
You will receive feedback about the accuracy of your time report.
Unlike the previous experiment…
You earn one point when your time report is sufficiently accurate.
You lose one point otherwise.
You will see a red line that shows the actual time of the beep.
Click here for an example of a correct answer

## Slide 46
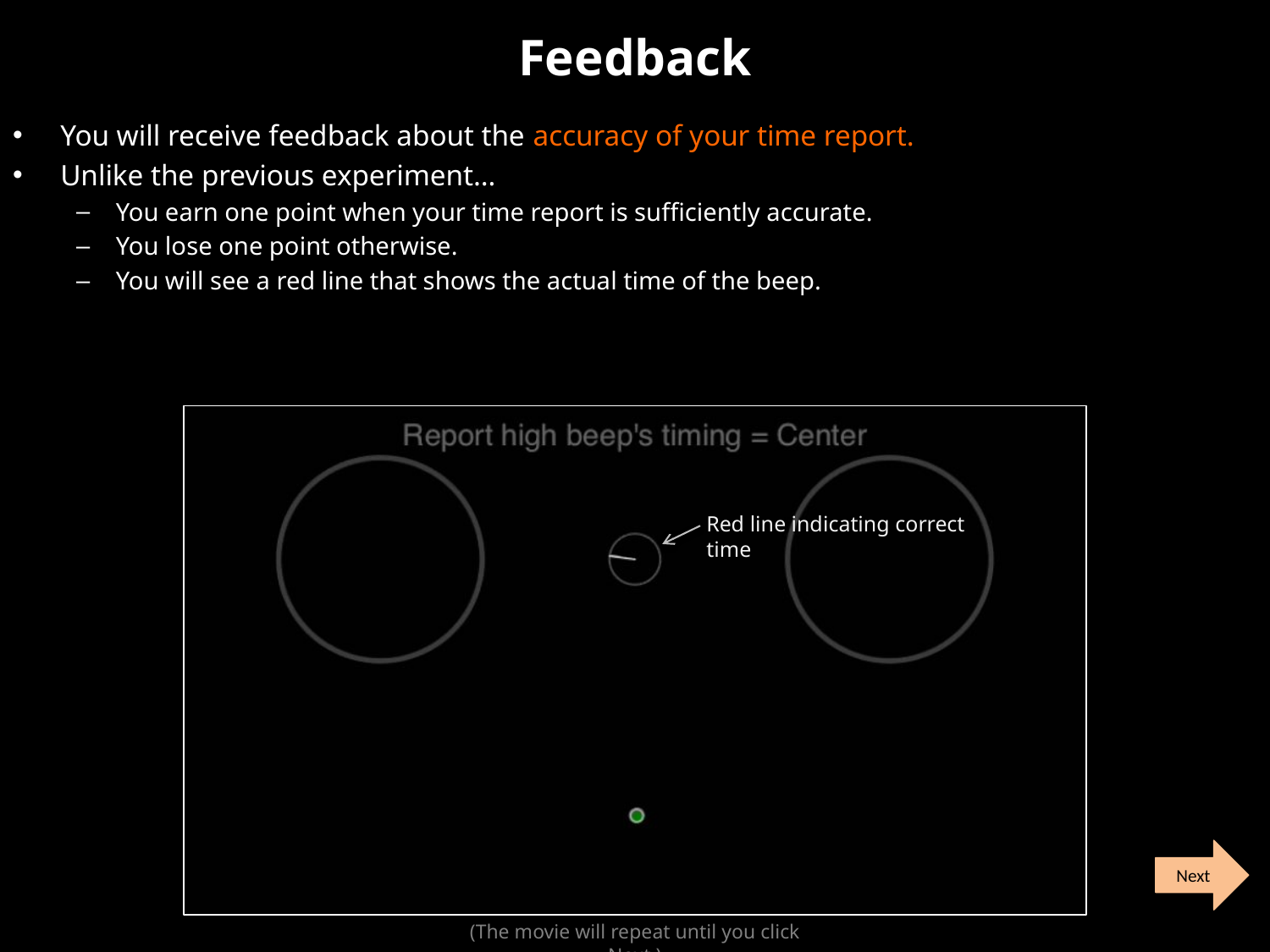

# Feedback
You will receive feedback about the accuracy of your time report.
Unlike the previous experiment…
You earn one point when your time report is sufficiently accurate.
You lose one point otherwise.
You will see a red line that shows the actual time of the beep.
Red line indicating correct time

## Slide 47
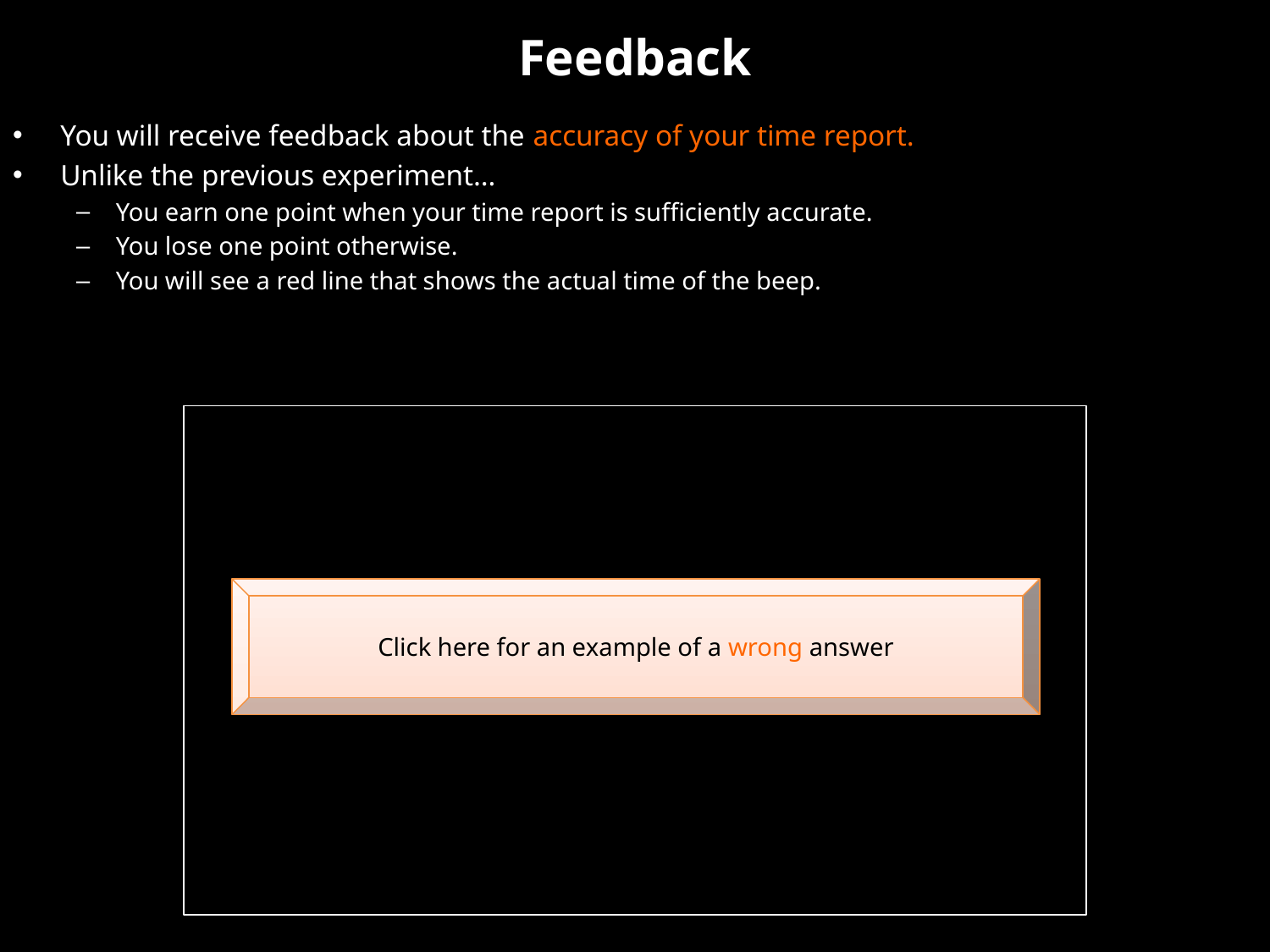

# Feedback
You will receive feedback about the accuracy of your time report.
Unlike the previous experiment…
You earn one point when your time report is sufficiently accurate.
You lose one point otherwise.
You will see a red line that shows the actual time of the beep.
Click here for an example of a wrong answer

## Slide 48
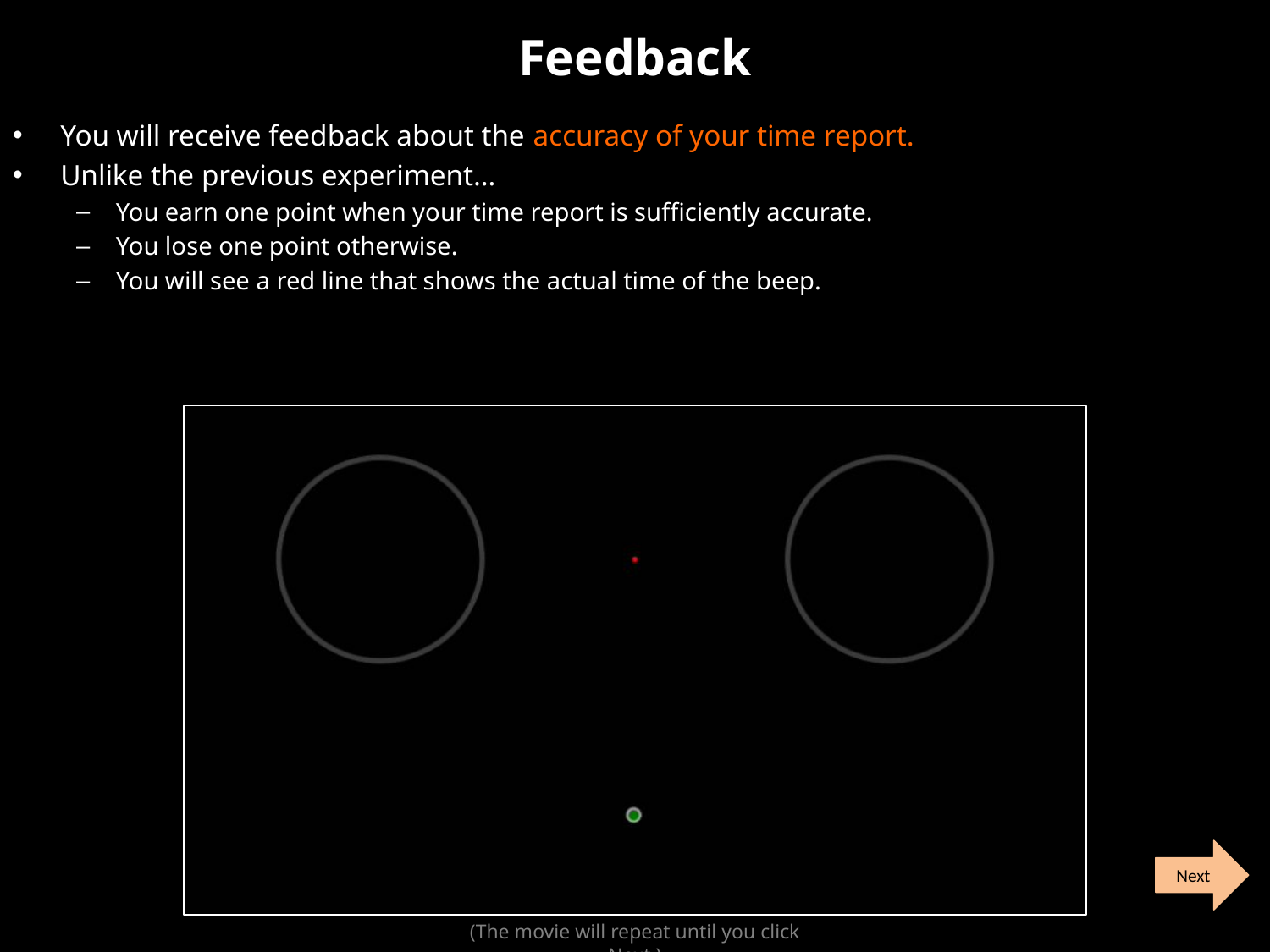

# Feedback
You will receive feedback about the accuracy of your time report.
Unlike the previous experiment…
You earn one point when your time report is sufficiently accurate.
You lose one point otherwise.
You will see a red line that shows the actual time of the beep.

## Slide 49
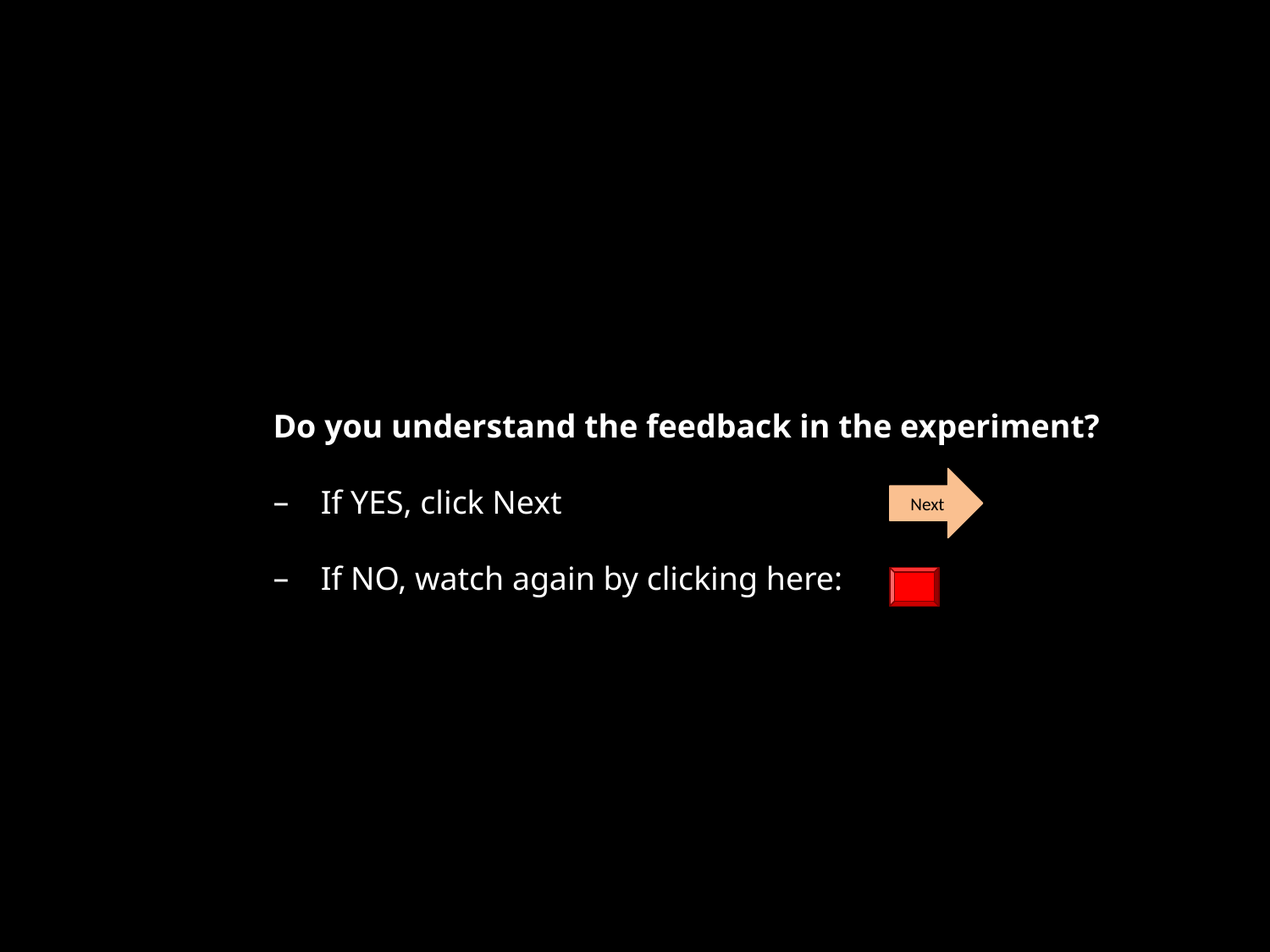

# Do you understand the feedback in the experiment?
Next
If YES, click Next
If NO, watch again by clicking here:

## Slide 50
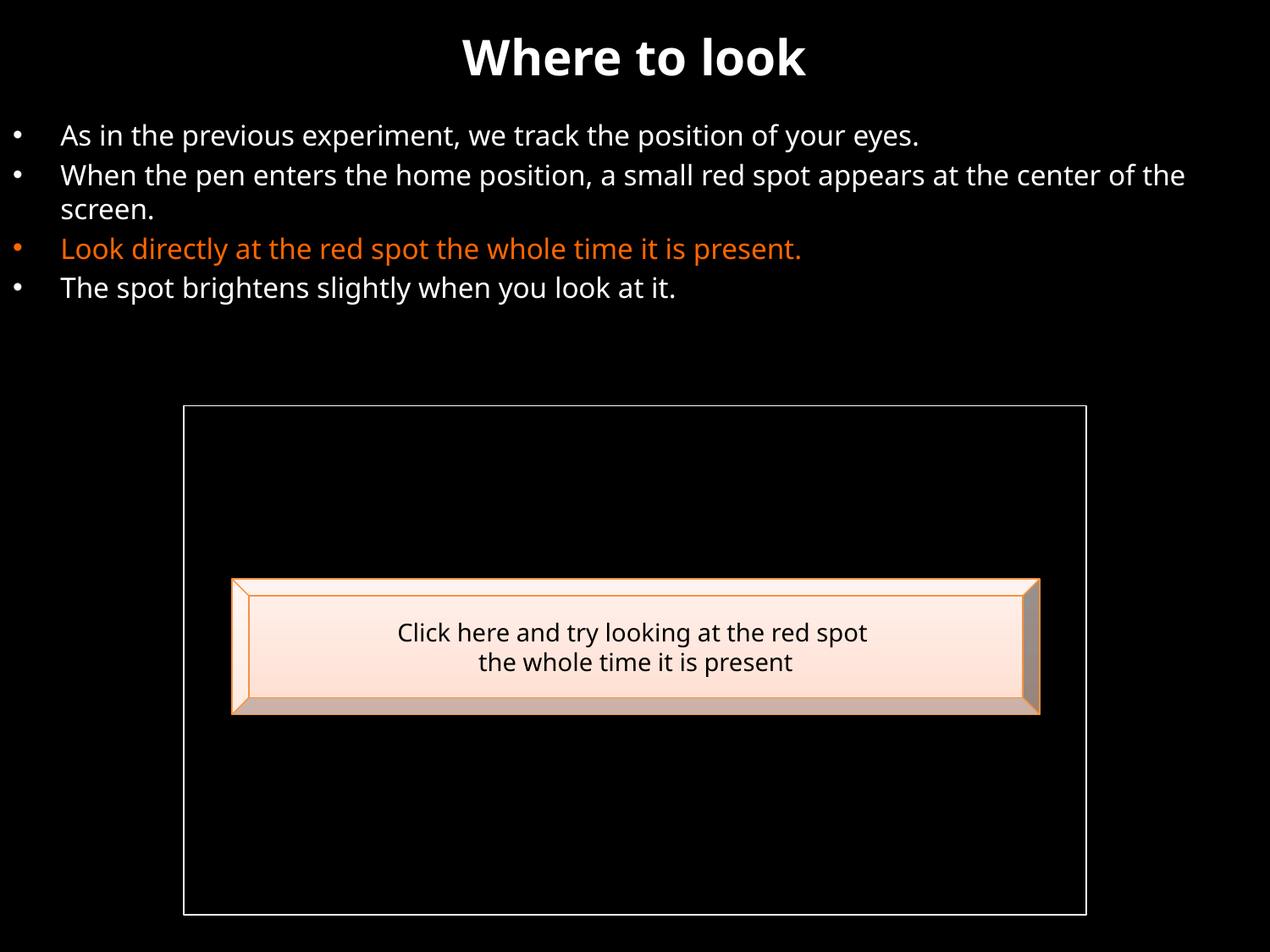

# Where to look
As in the previous experiment, we track the position of your eyes.
When the pen enters the home position, a small red spot appears at the center of the screen.
Look directly at the red spot the whole time it is present.
The spot brightens slightly when you look at it.
Click here and try looking at the red spot
the whole time it is present

## Slide 51
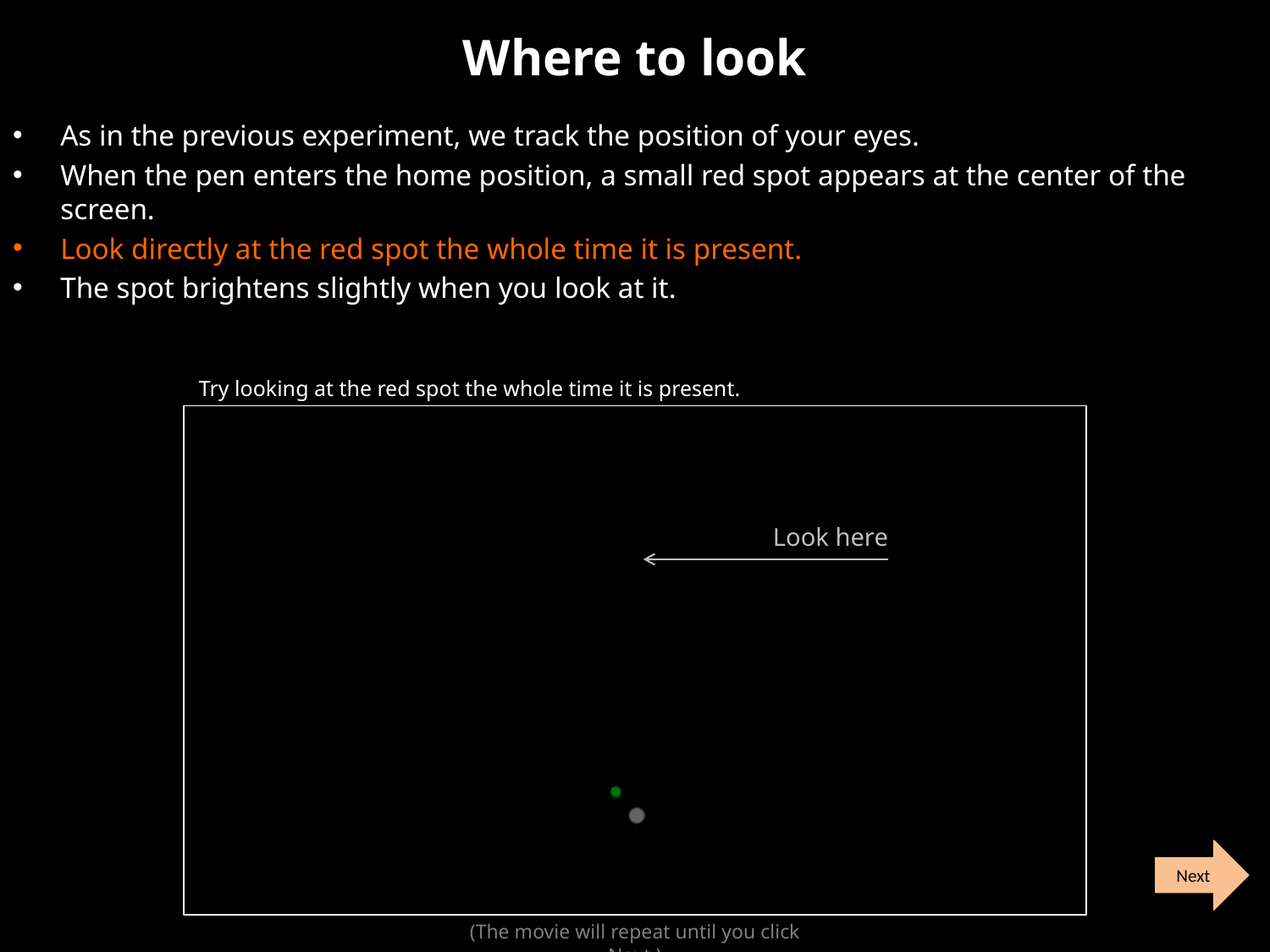

# Where to look
As in the previous experiment, we track the position of your eyes.
When the pen enters the home position, a small red spot appears at the center of the screen.
Look directly at the red spot the whole time it is present.
The spot brightens slightly when you look at it.
Try looking at the red spot the whole time it is present.
Look here

## Slide 52
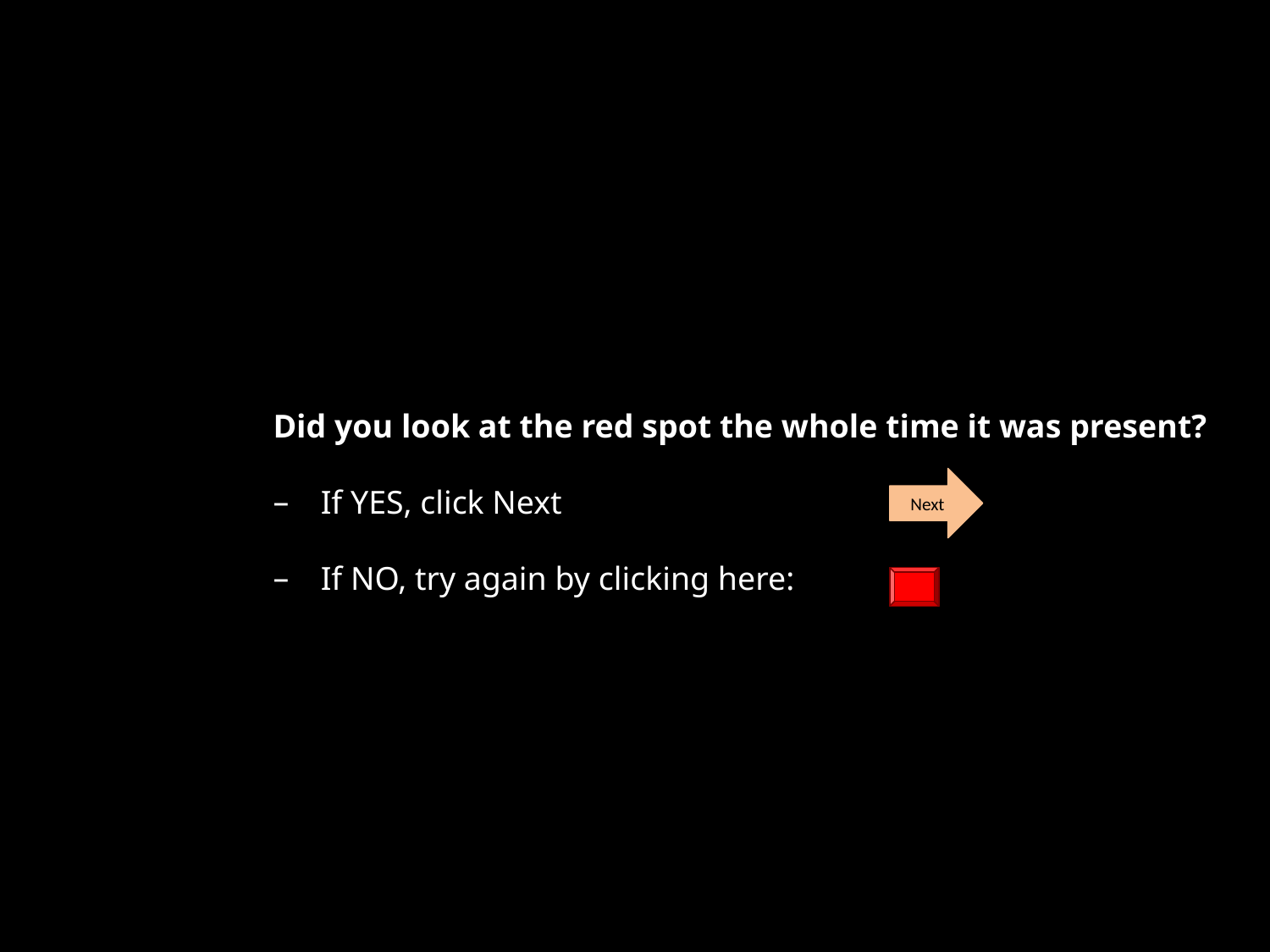

# Did you look at the red spot the whole time it was present?
Next
If YES, click Next
If NO, try again by clicking here:

## Slide 53
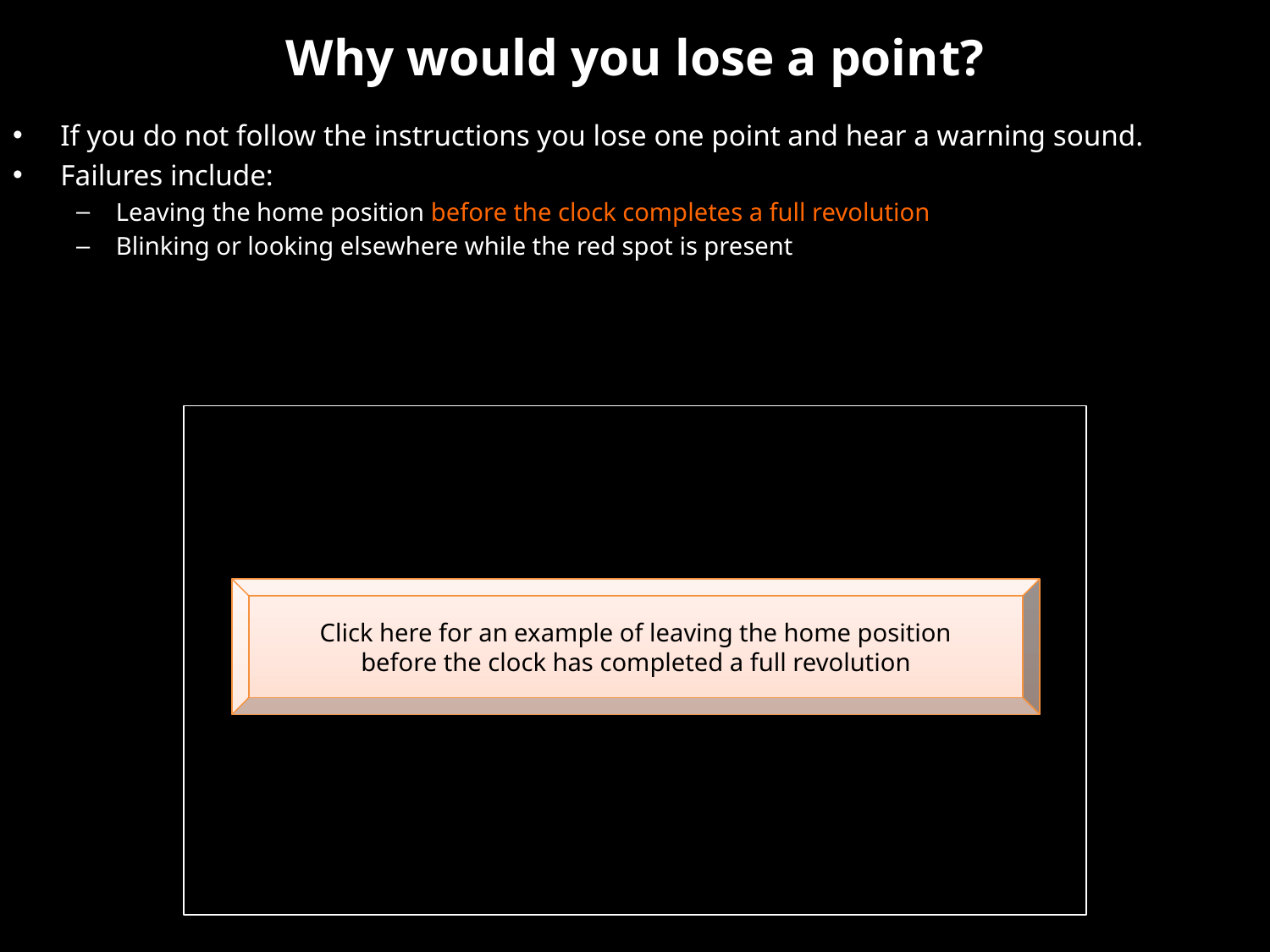

# Why would you lose a point?
If you do not follow the instructions you lose one point and hear a warning sound.
Failures include:
Leaving the home position before the clock completes a full revolution
Blinking or looking elsewhere while the red spot is present
Click here for an example of leaving the home position
before the clock has completed a full revolution

## Slide 54
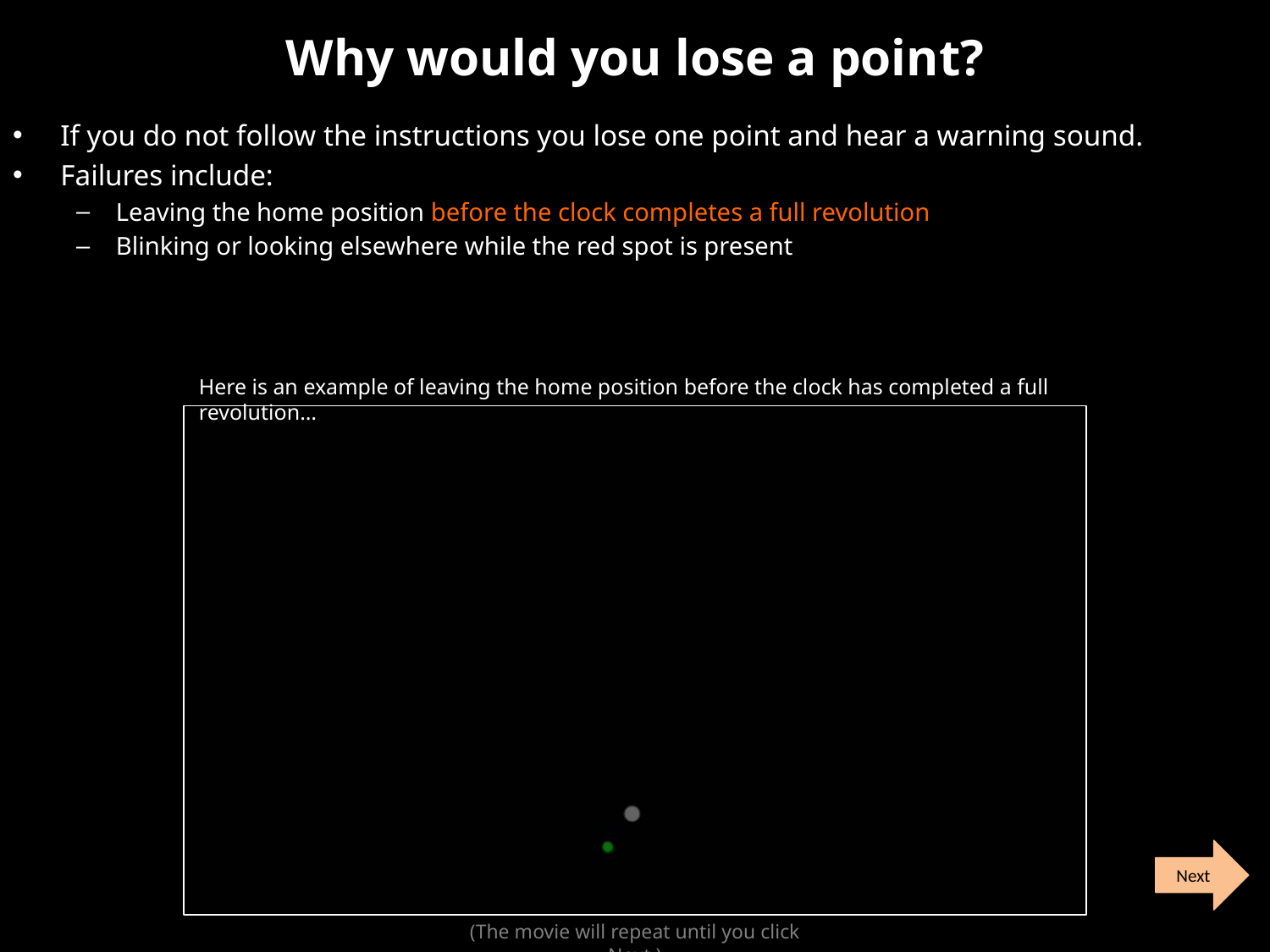

# Why would you lose a point?
If you do not follow the instructions you lose one point and hear a warning sound.
Failures include:
Leaving the home position before the clock completes a full revolution
Blinking or looking elsewhere while the red spot is present
Here is an example of leaving the home position before the clock has completed a full revolution…

## Slide 55
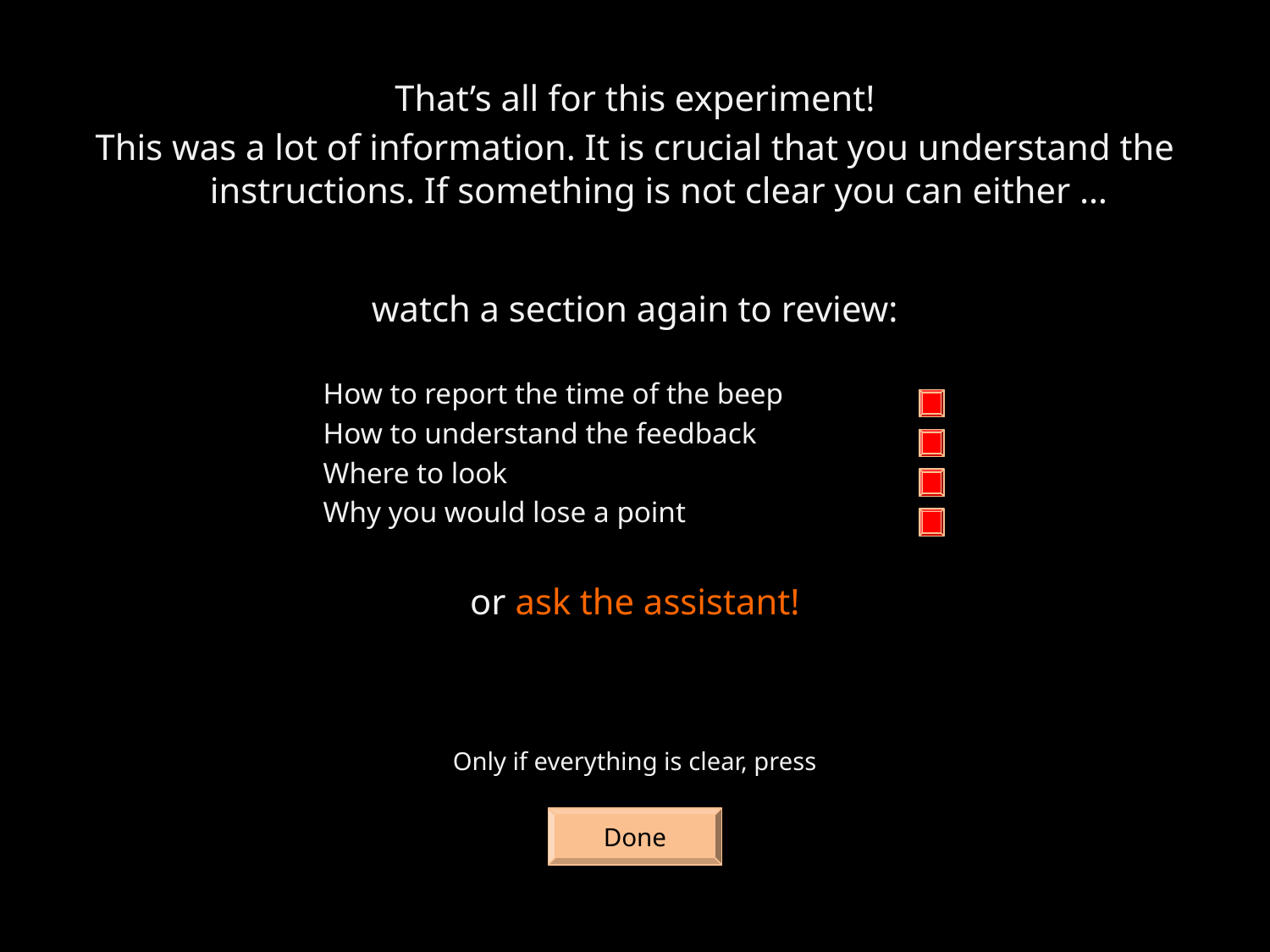

That’s all for this experiment!
This was a lot of information. It is crucial that you understand the instructions. If something is not clear you can either …
watch a section again to review:
How to report the time of the beep
How to understand the feedback
Where to look
Why you would lose a point
or ask the assistant!
Only if everything is clear, press
Done

## Slide 56
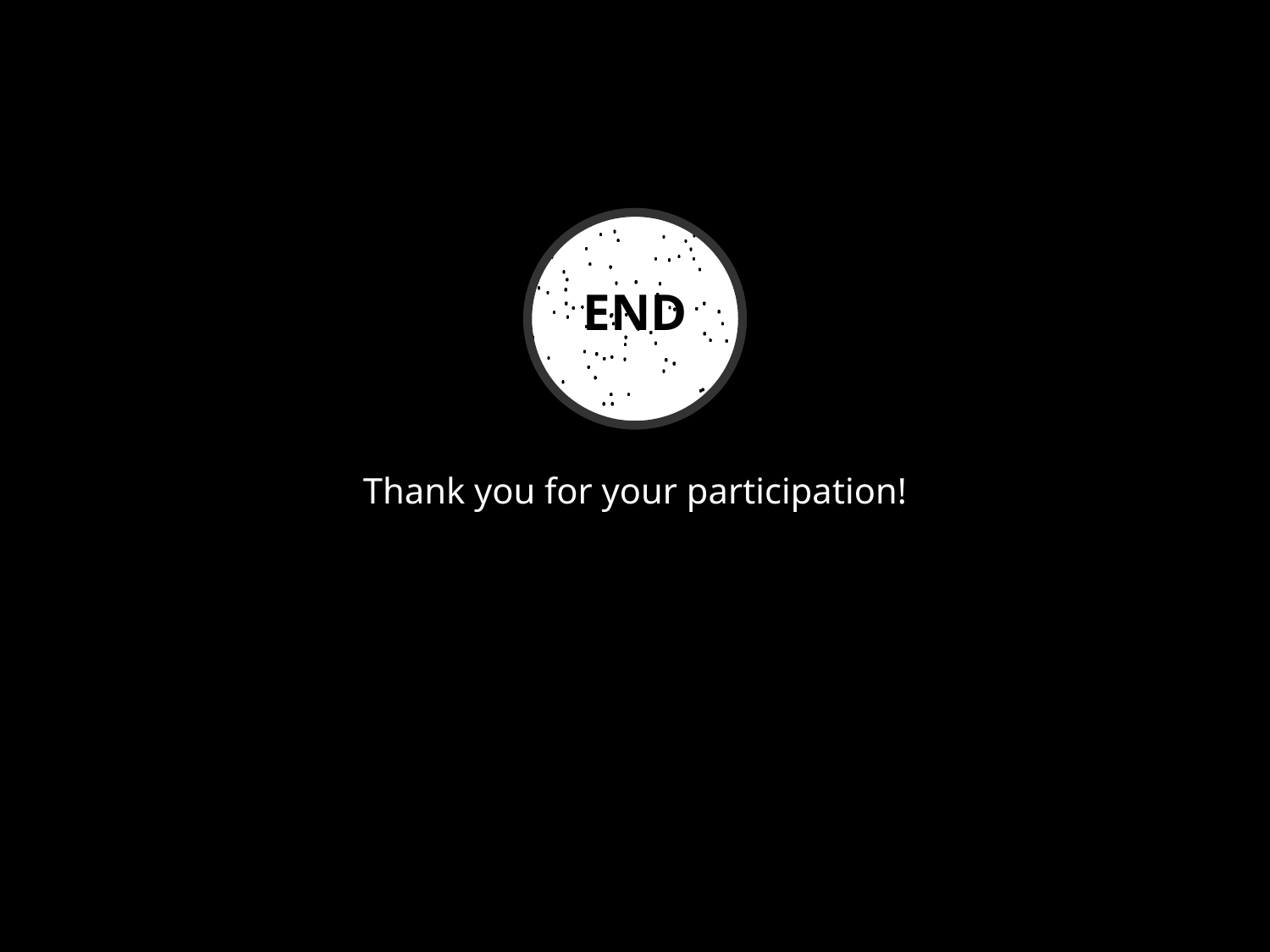

# END
Thank you for your participation!

## Slide 57
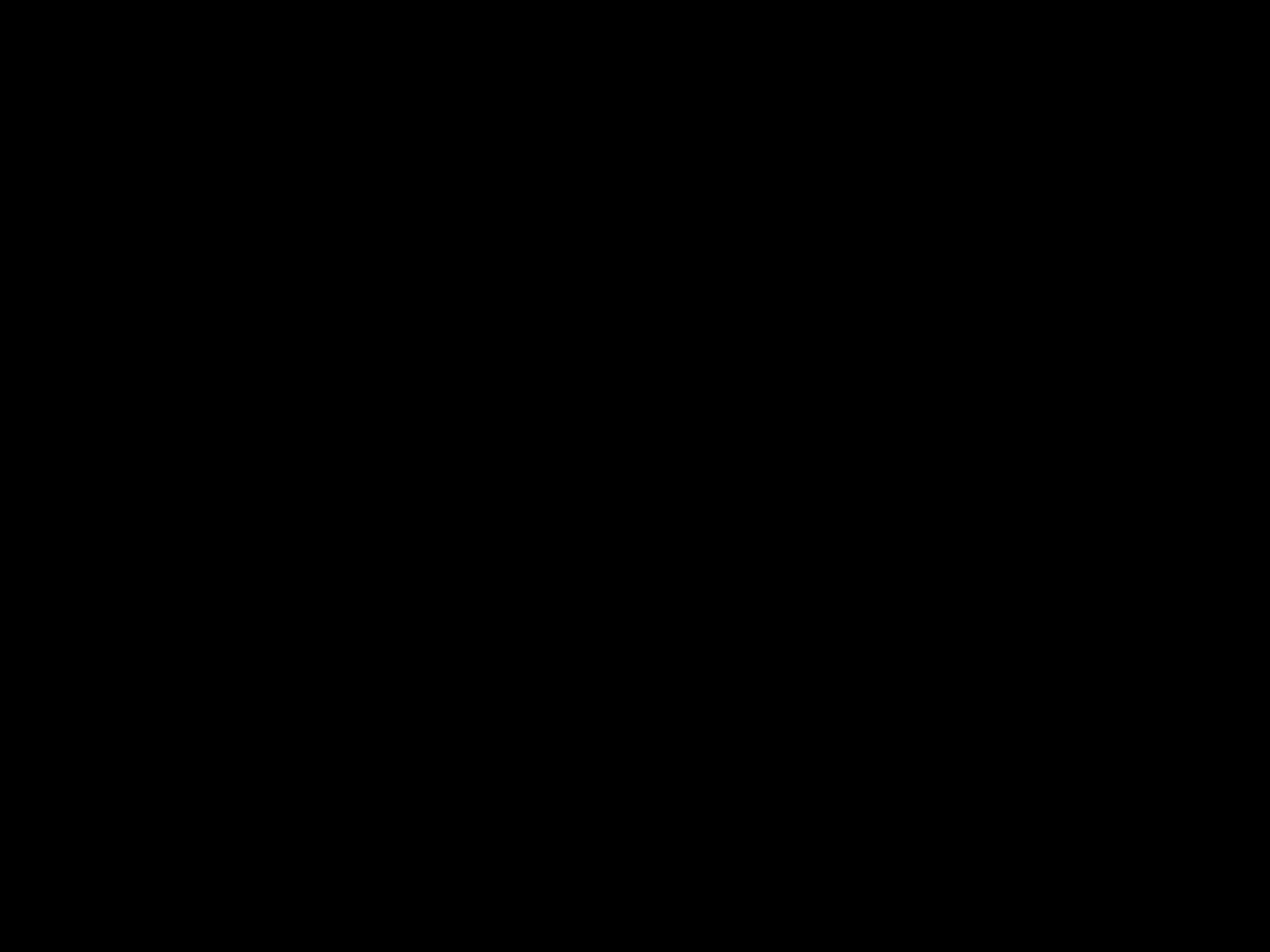

#

## Slide 58
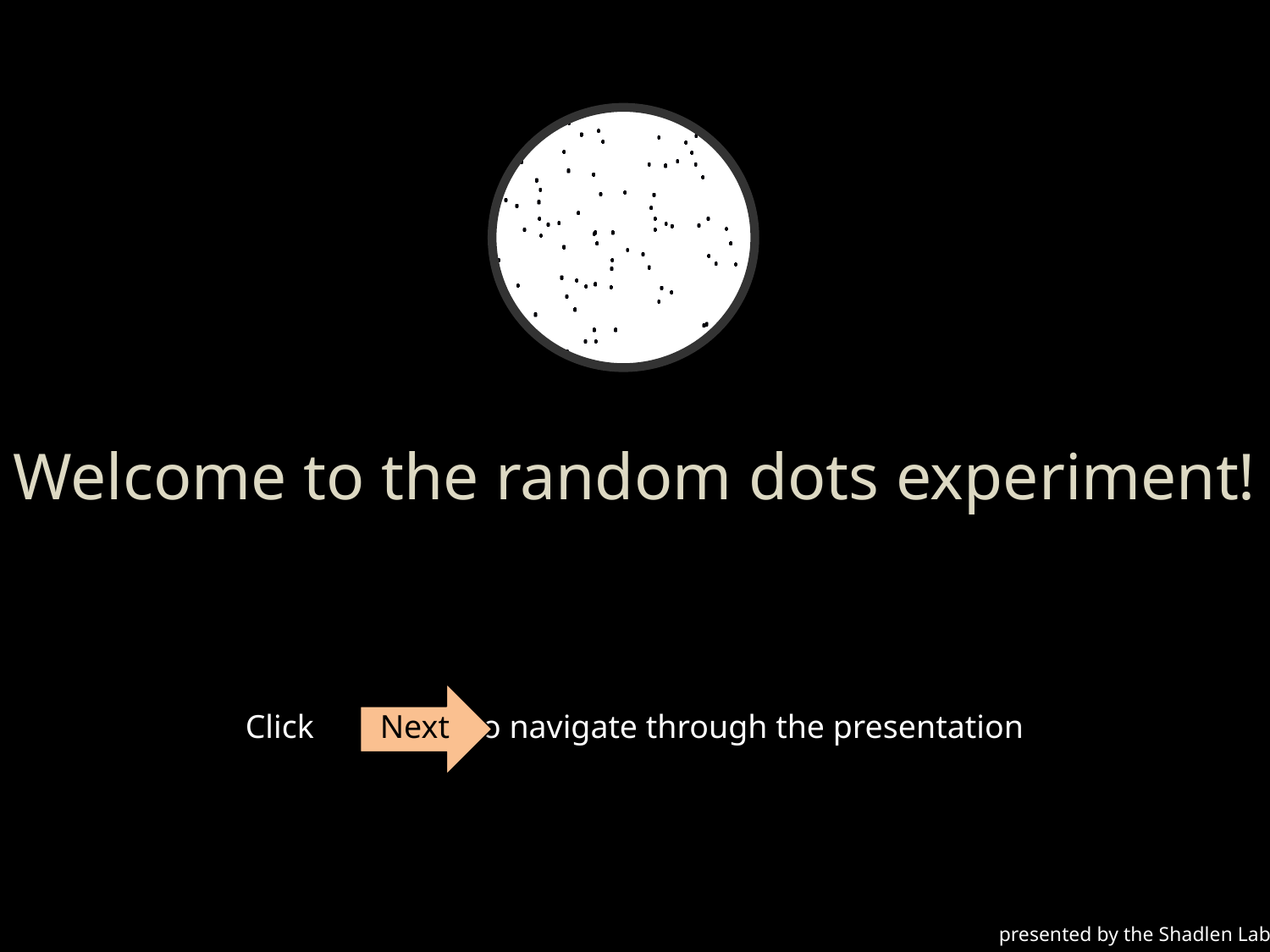

# Welcome to the random dots experiment!
Next
Click to navigate through the presentation
presented by the Shadlen Lab

## Slide 59
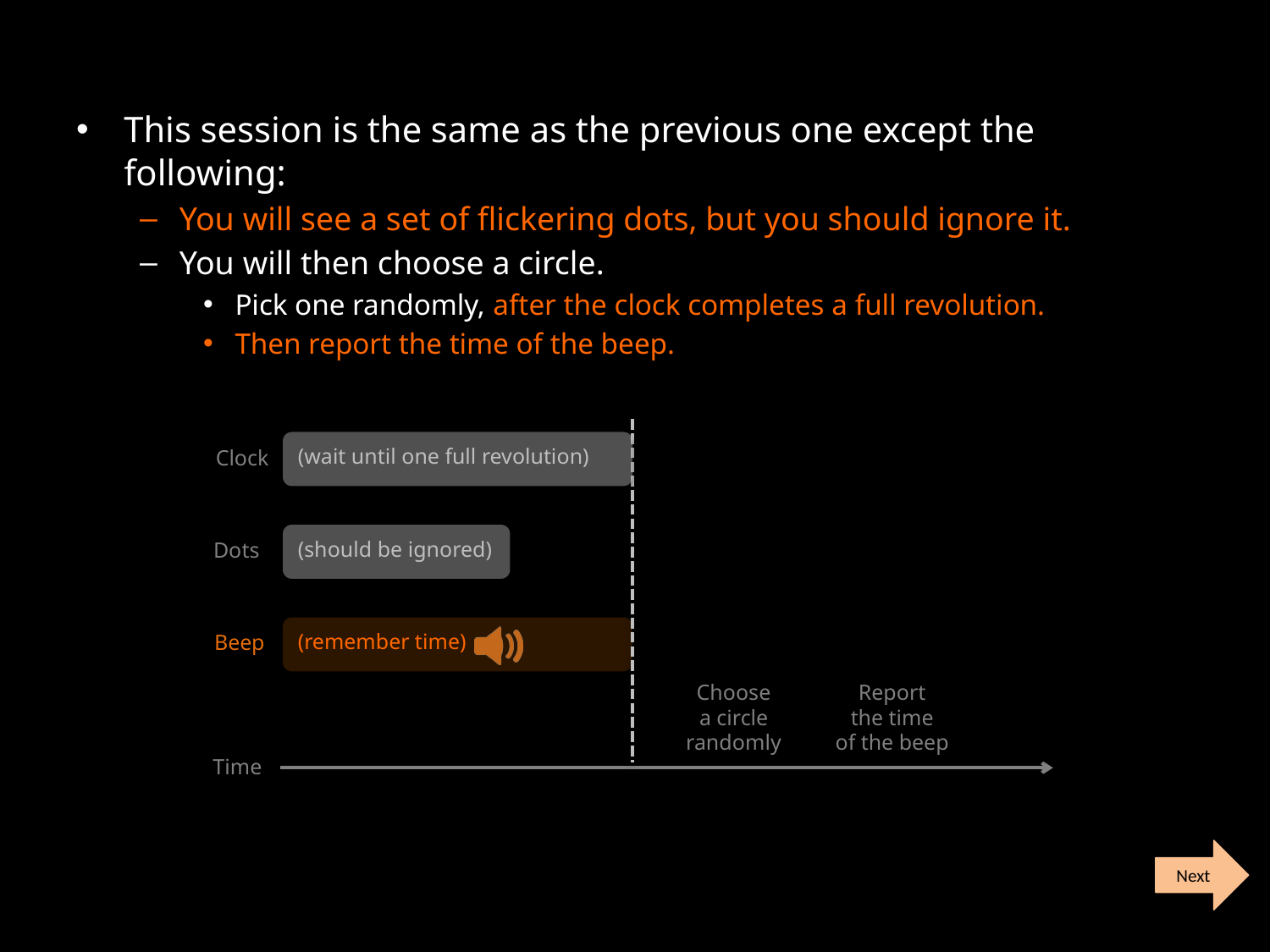

This session is the same as the previous one except the following:
You will see a set of flickering dots, but you should ignore it.
You will then choose a circle.
Pick one randomly, after the clock completes a full revolution.
Then report the time of the beep.
(wait until one full revolution)
Clock
(should be ignored)
Dots
(remember time)
Beep
Choose
a circle
randomly
Report
the time
of the beep
Time
Next

## Slide 60
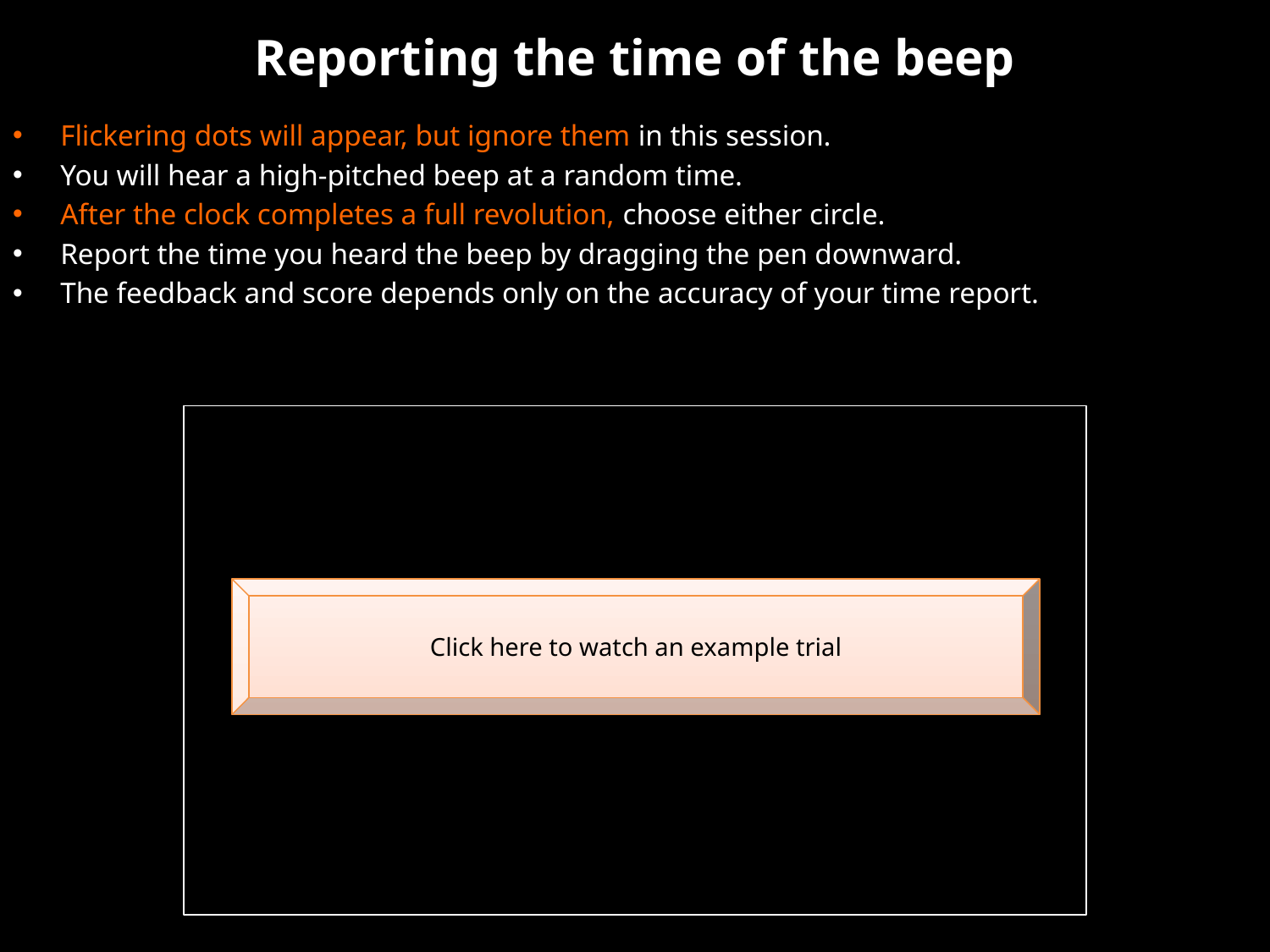

# Reporting the time of the beep
Flickering dots will appear, but ignore them in this session.
You will hear a high-pitched beep at a random time.
After the clock completes a full revolution, choose either circle.
Report the time you heard the beep by dragging the pen downward.
The feedback and score depends only on the accuracy of your time report.
Click here to watch an example trial

## Slide 61
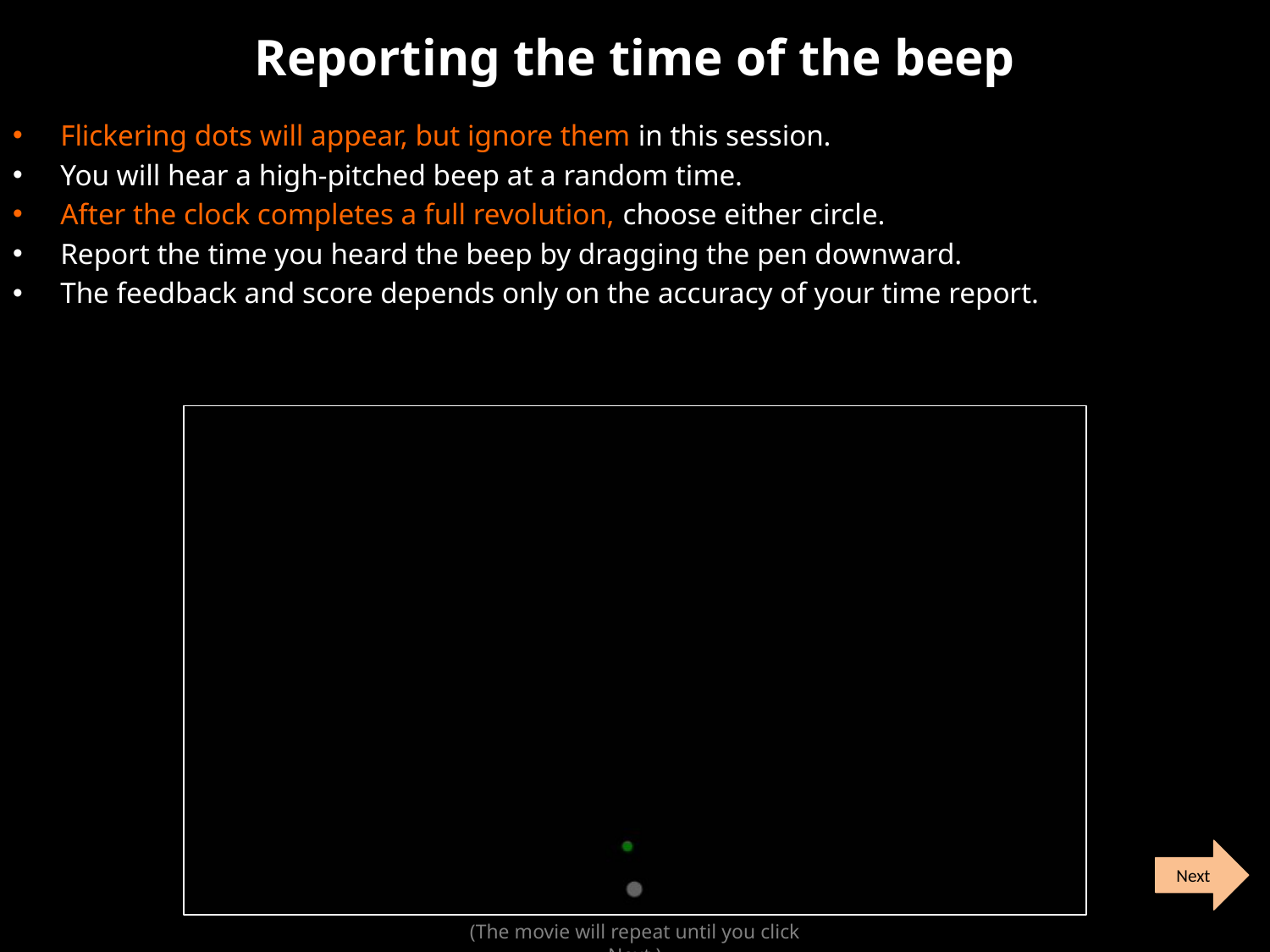

# Reporting the time of the beep
Flickering dots will appear, but ignore them in this session.
You will hear a high-pitched beep at a random time.
After the clock completes a full revolution, choose either circle.
Report the time you heard the beep by dragging the pen downward.
The feedback and score depends only on the accuracy of your time report.

## Slide 62
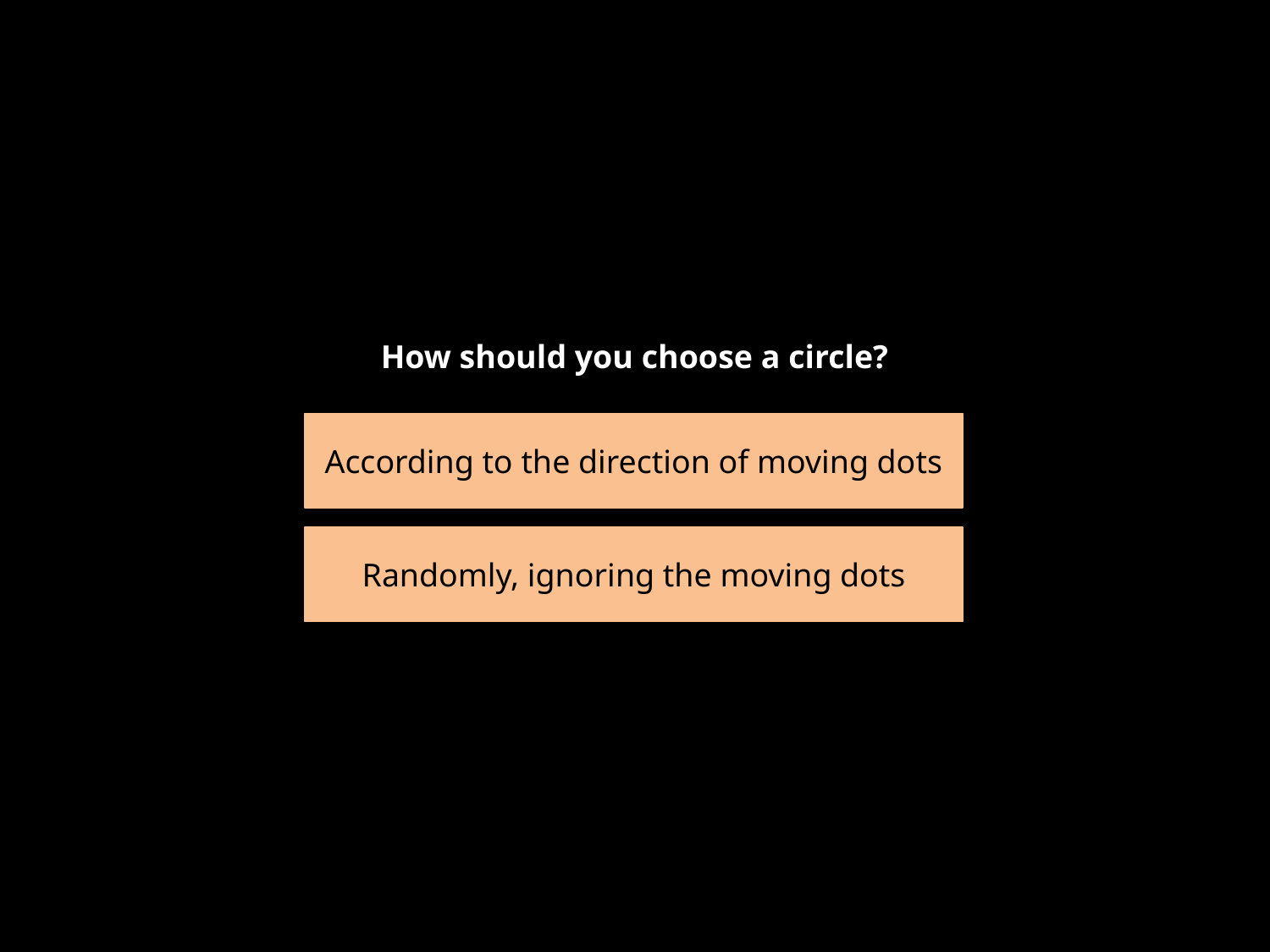

How should you choose a circle?
According to the direction of moving dots
Randomly, ignoring the moving dots

## Slide 63
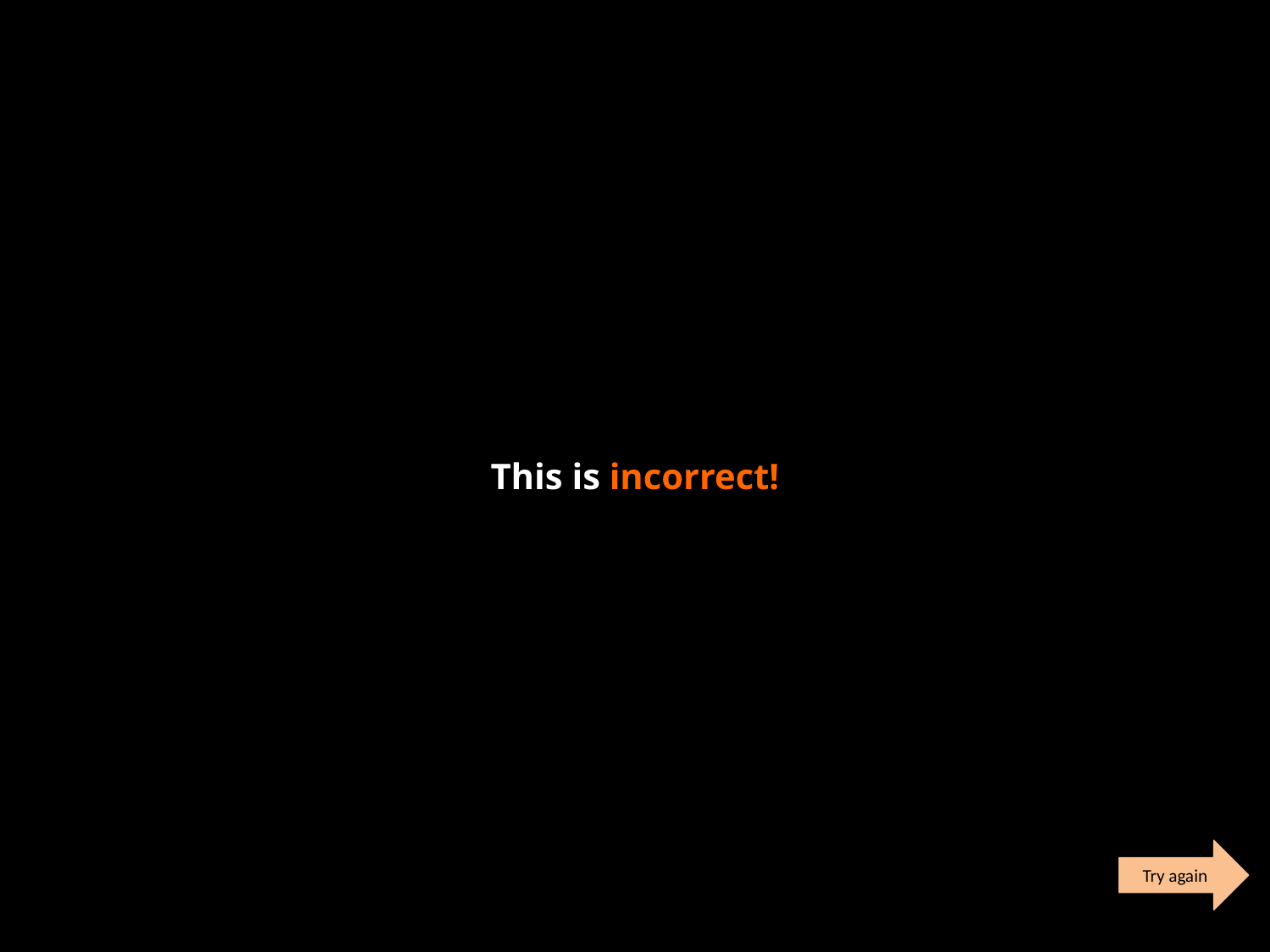

This is incorrect!
Try again

## Slide 64
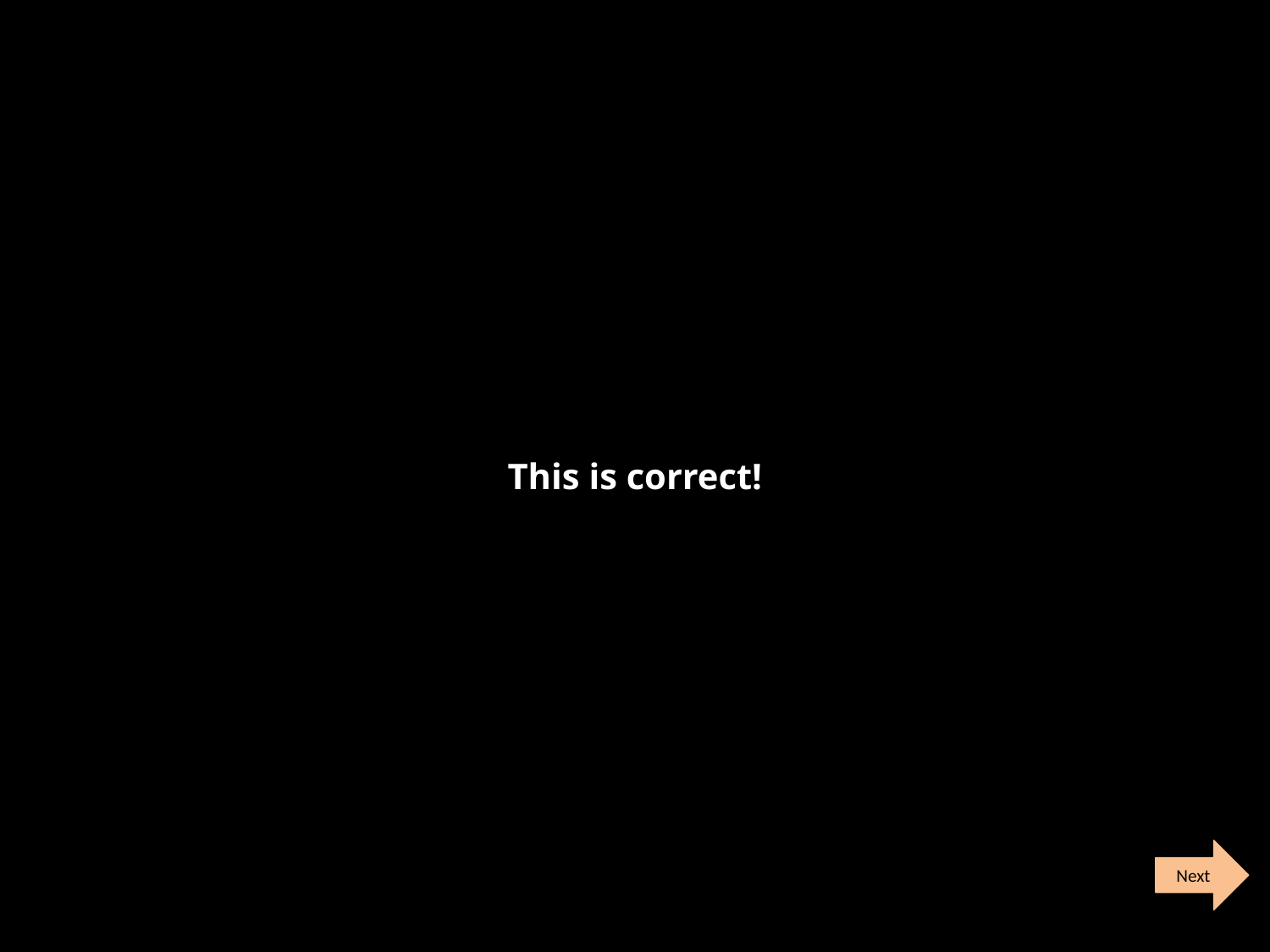

This is correct!
Next

## Slide 65
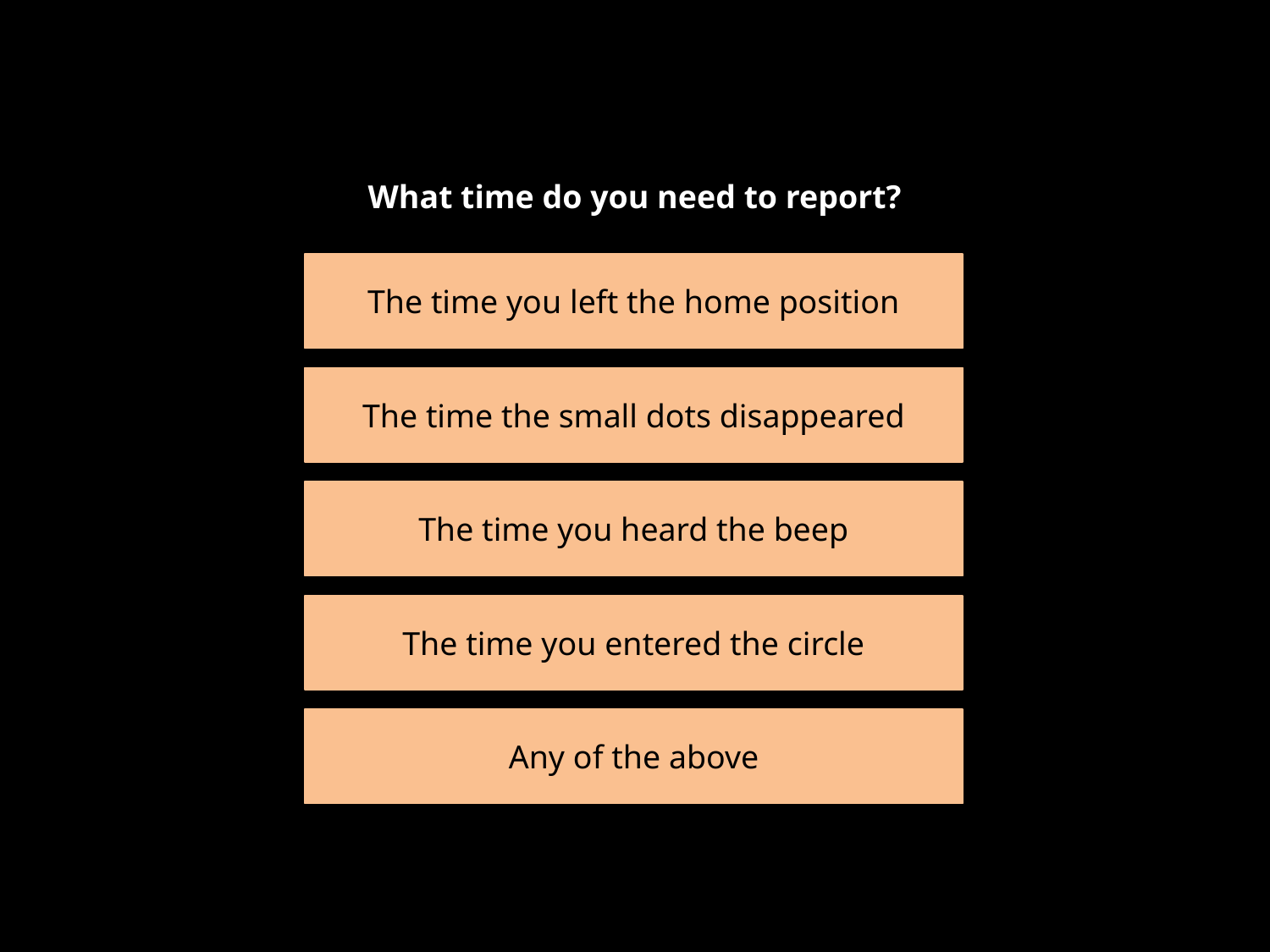

What time do you need to report?
The time you left the home position
The time the small dots disappeared
The time you heard the beep
The time you entered the circle
Any of the above

## Slide 66
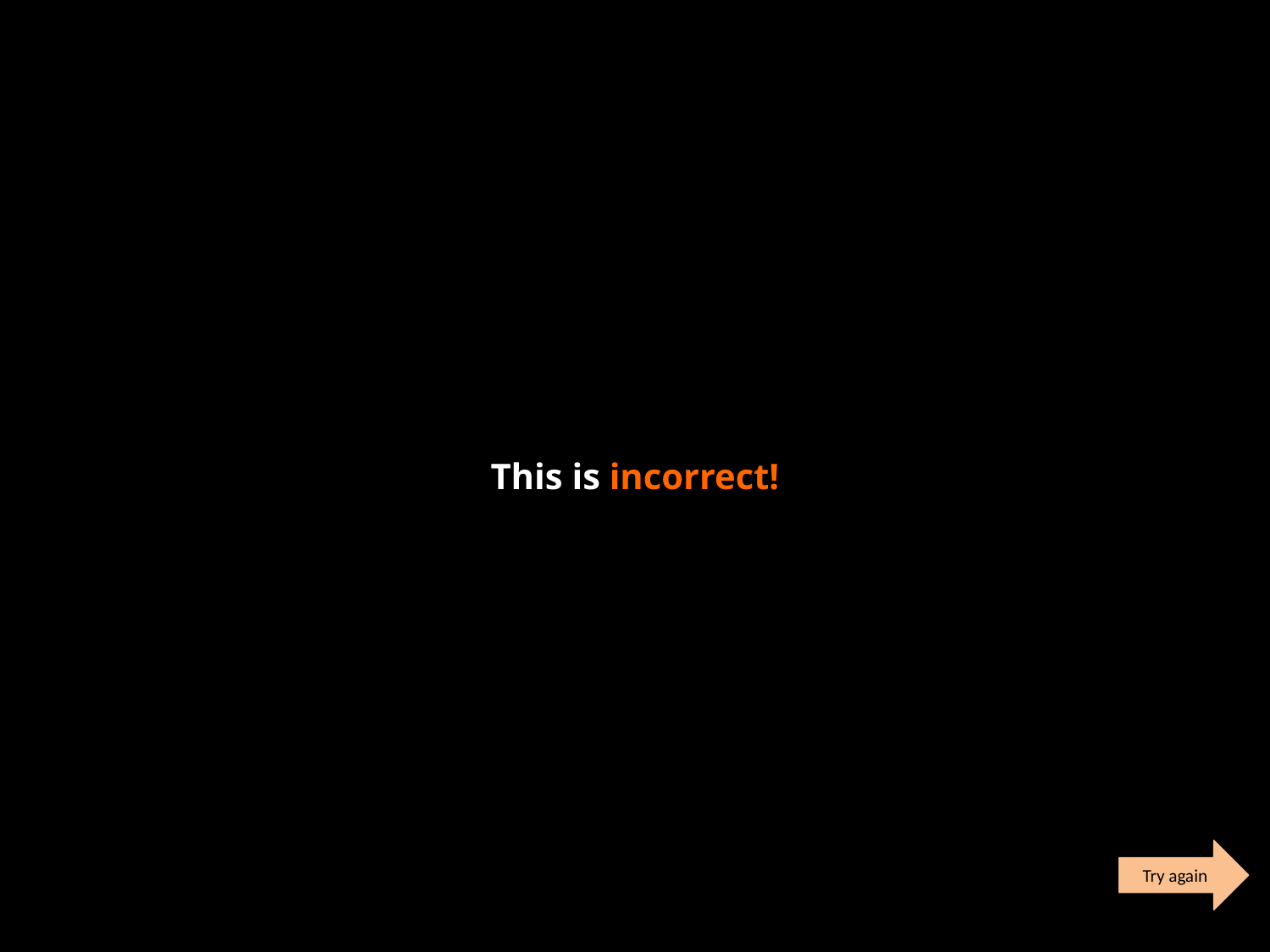

This is incorrect!
Try again

## Slide 67
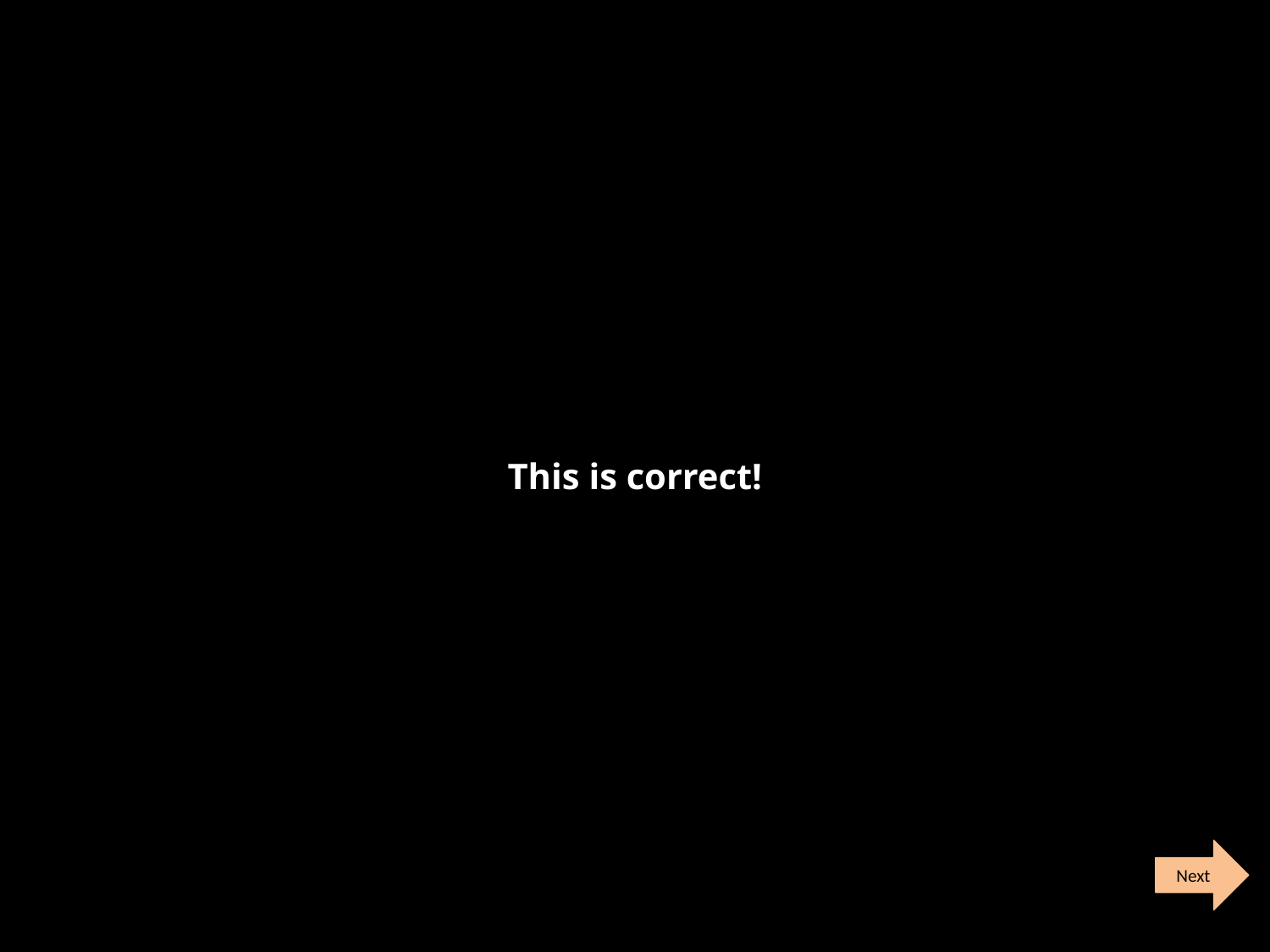

This is correct!
Next

## Slide 68
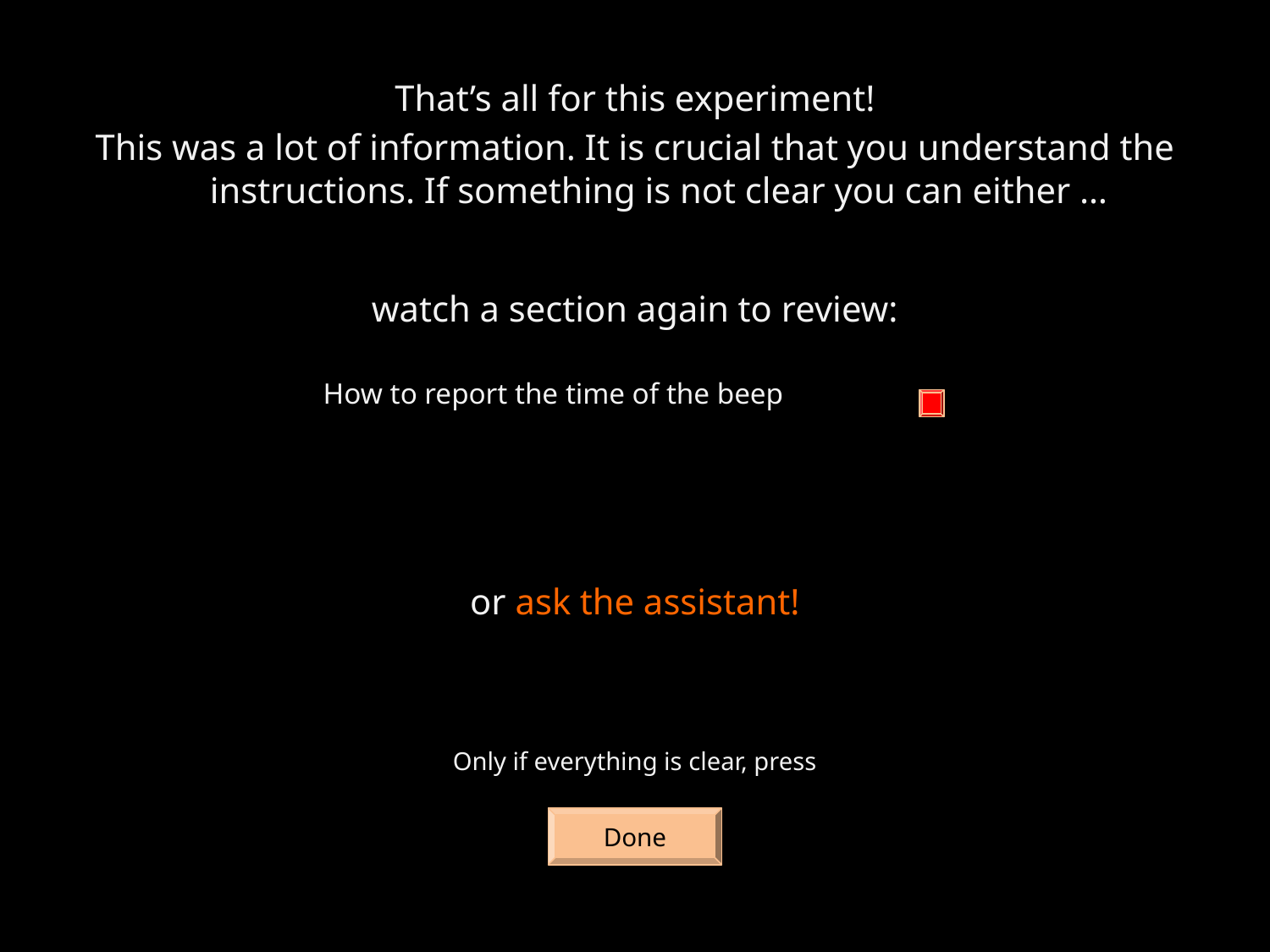

That’s all for this experiment!
This was a lot of information. It is crucial that you understand the instructions. If something is not clear you can either …
watch a section again to review:
How to report the time of the beep
or ask the assistant!
Only if everything is clear, press
Done

## Slide 69
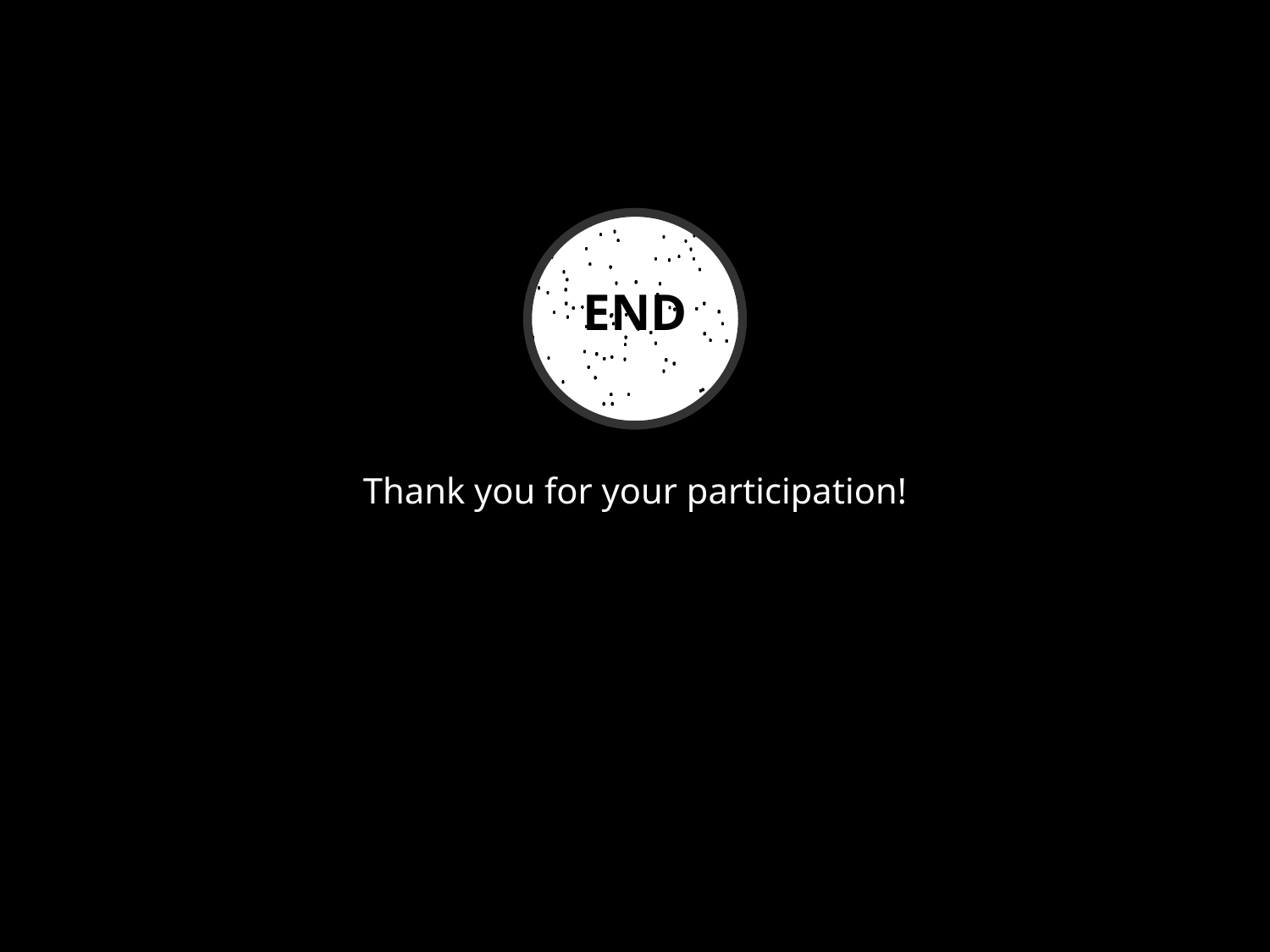

# END
Thank you for your participation!

## Slide 70
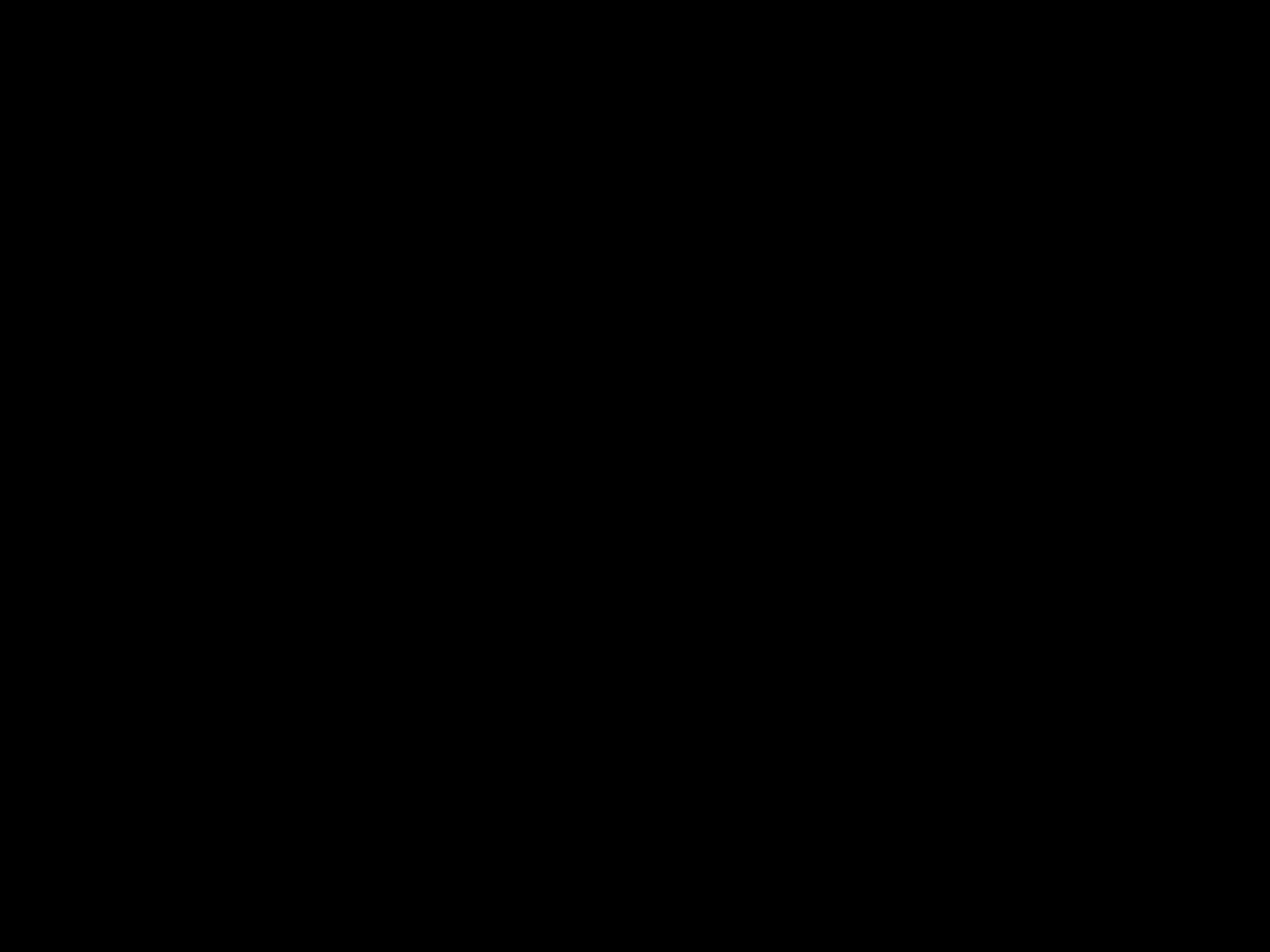

#

## Slide 71
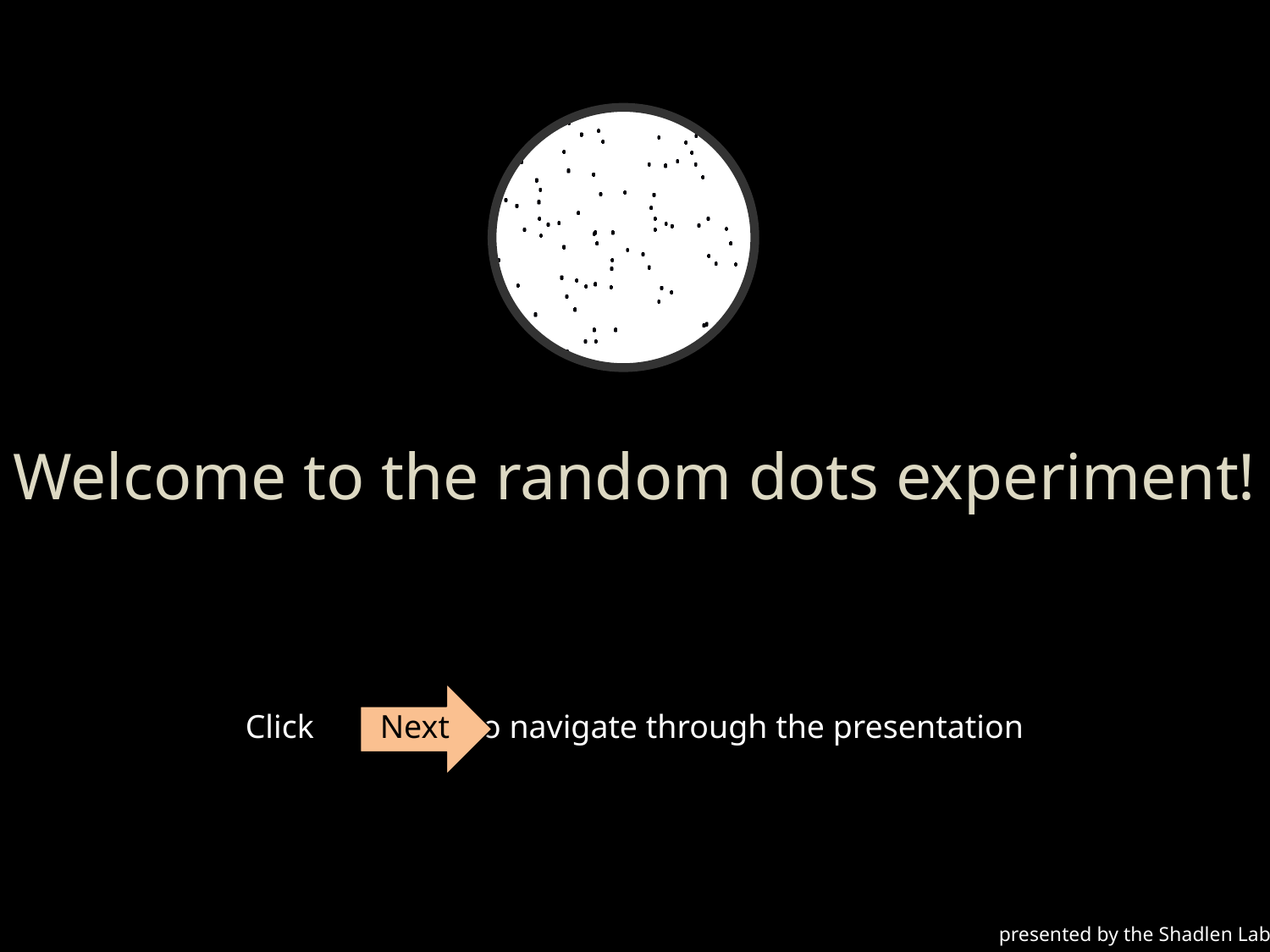

# Welcome to the random dots experiment!
Next
Click to navigate through the presentation
presented by the Shadlen Lab

## Slide 72
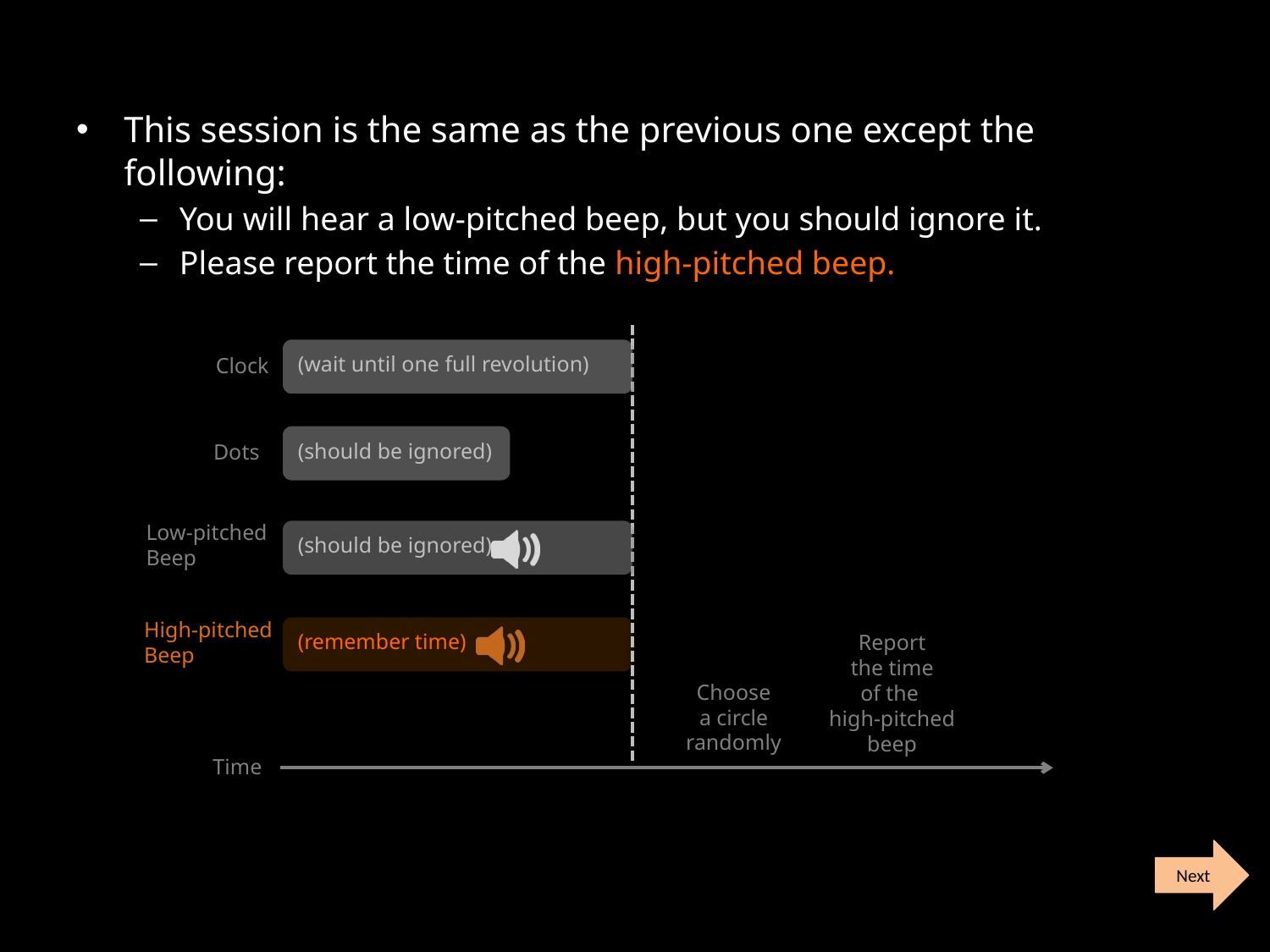

This session is the same as the previous one except the following:
You will hear a low-pitched beep, but you should ignore it.
Please report the time of the high-pitched beep.
(wait until one full revolution)
Clock
(should be ignored)
Dots
Low-pitched
Beep
(should be ignored)
High-pitched
Beep
(remember time)
Report
the time
of the
high-pitched
beep
Choose
a circle
randomly
Time
Next

## Slide 73
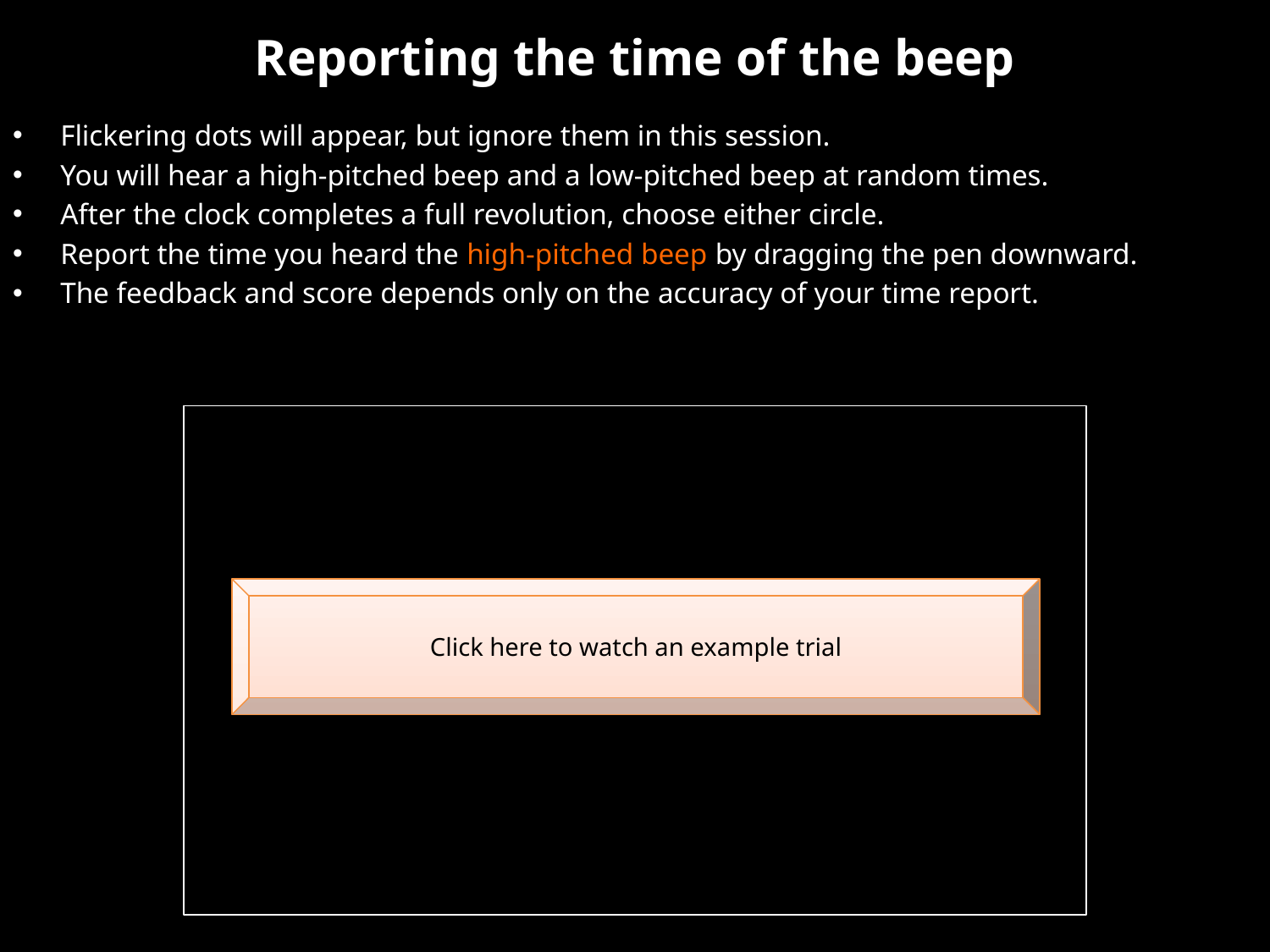

# Reporting the time of the beep
Flickering dots will appear, but ignore them in this session.
You will hear a high-pitched beep and a low-pitched beep at random times.
After the clock completes a full revolution, choose either circle.
Report the time you heard the high-pitched beep by dragging the pen downward.
The feedback and score depends only on the accuracy of your time report.
Click here to watch an example trial

## Slide 74
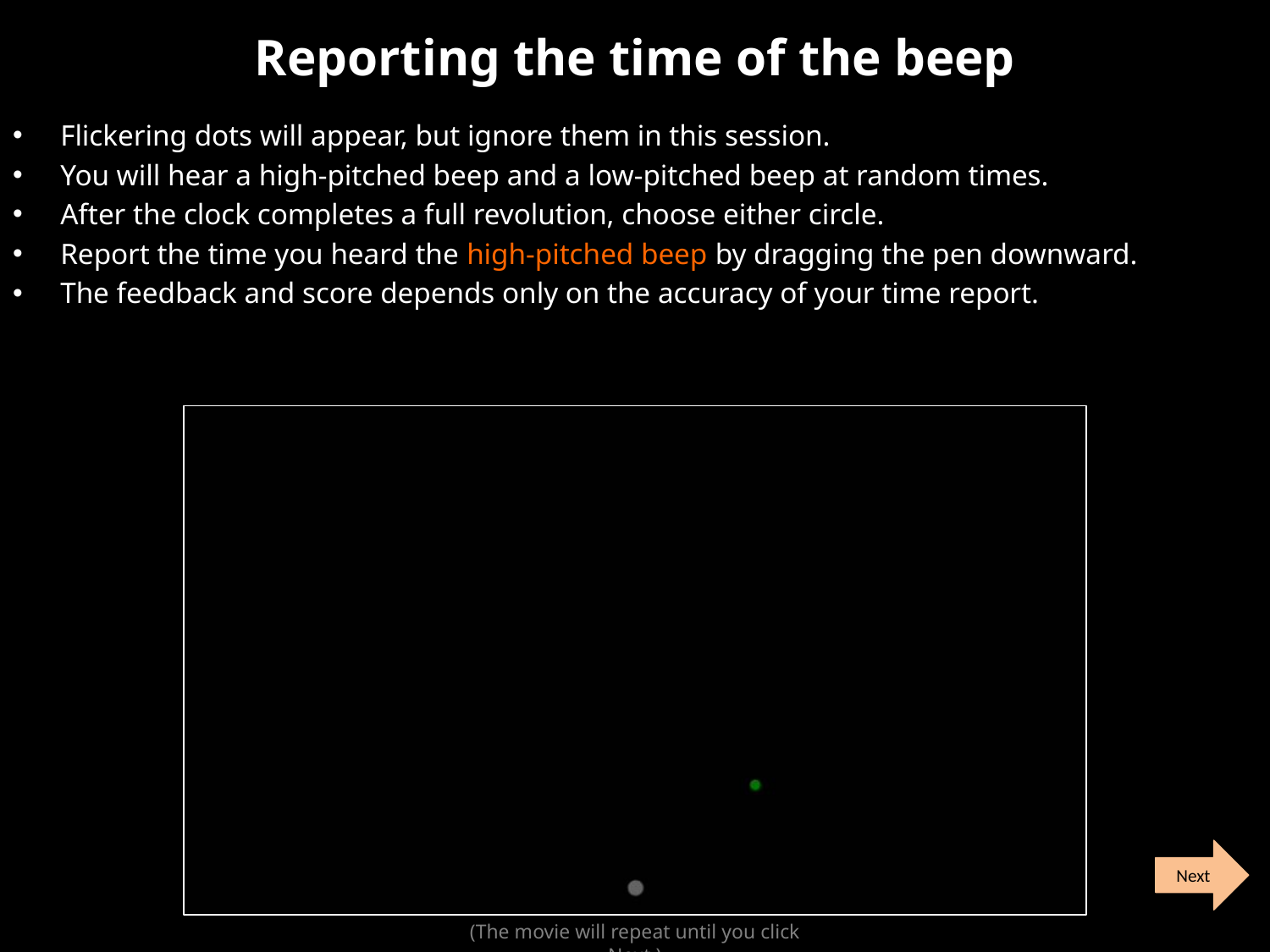

# Reporting the time of the beep
Flickering dots will appear, but ignore them in this session.
You will hear a high-pitched beep and a low-pitched beep at random times.
After the clock completes a full revolution, choose either circle.
Report the time you heard the high-pitched beep by dragging the pen downward.
The feedback and score depends only on the accuracy of your time report.

## Slide 75
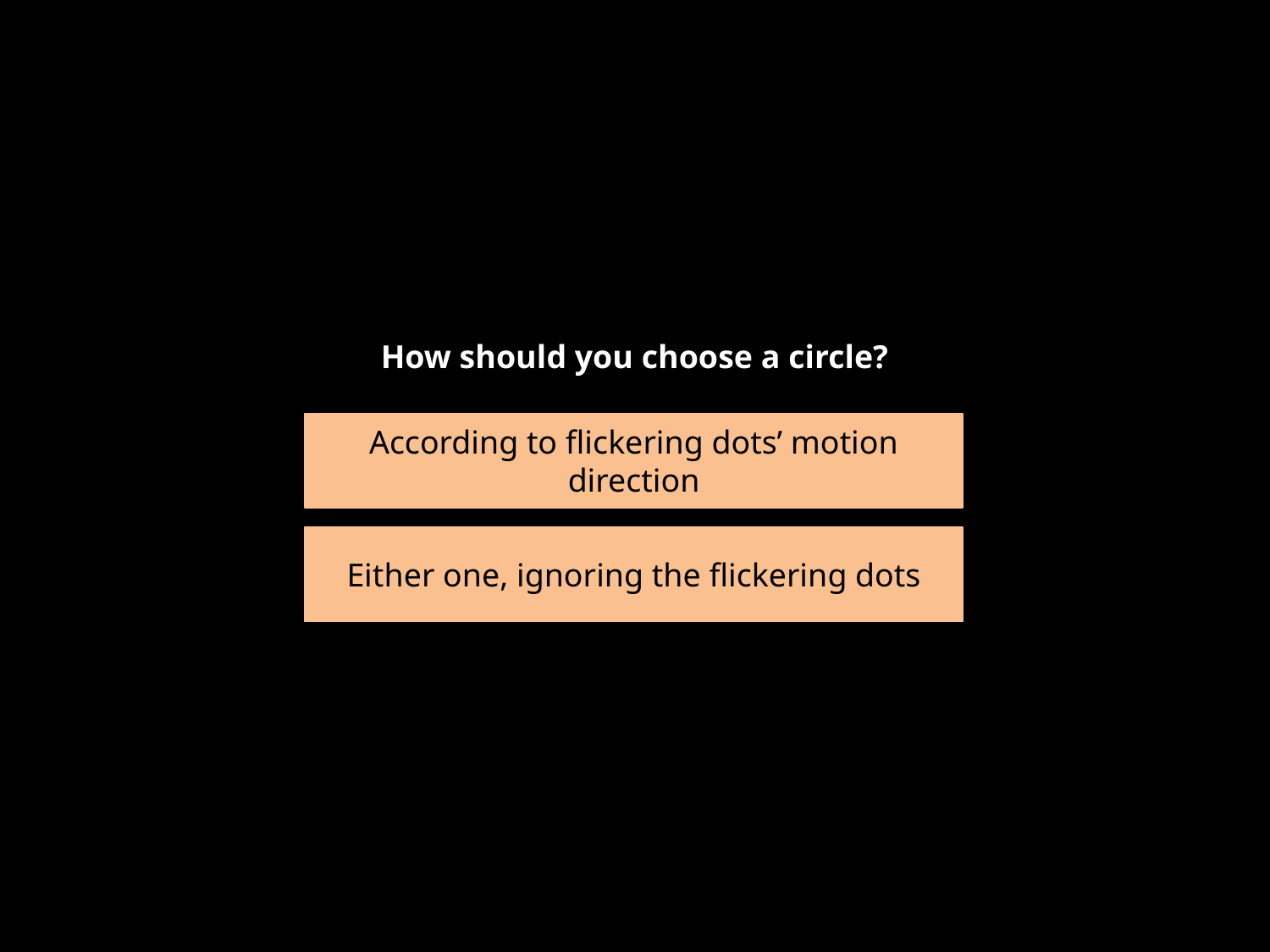

How should you choose a circle?
According to flickering dots’ motion direction
Either one, ignoring the flickering dots

## Slide 76
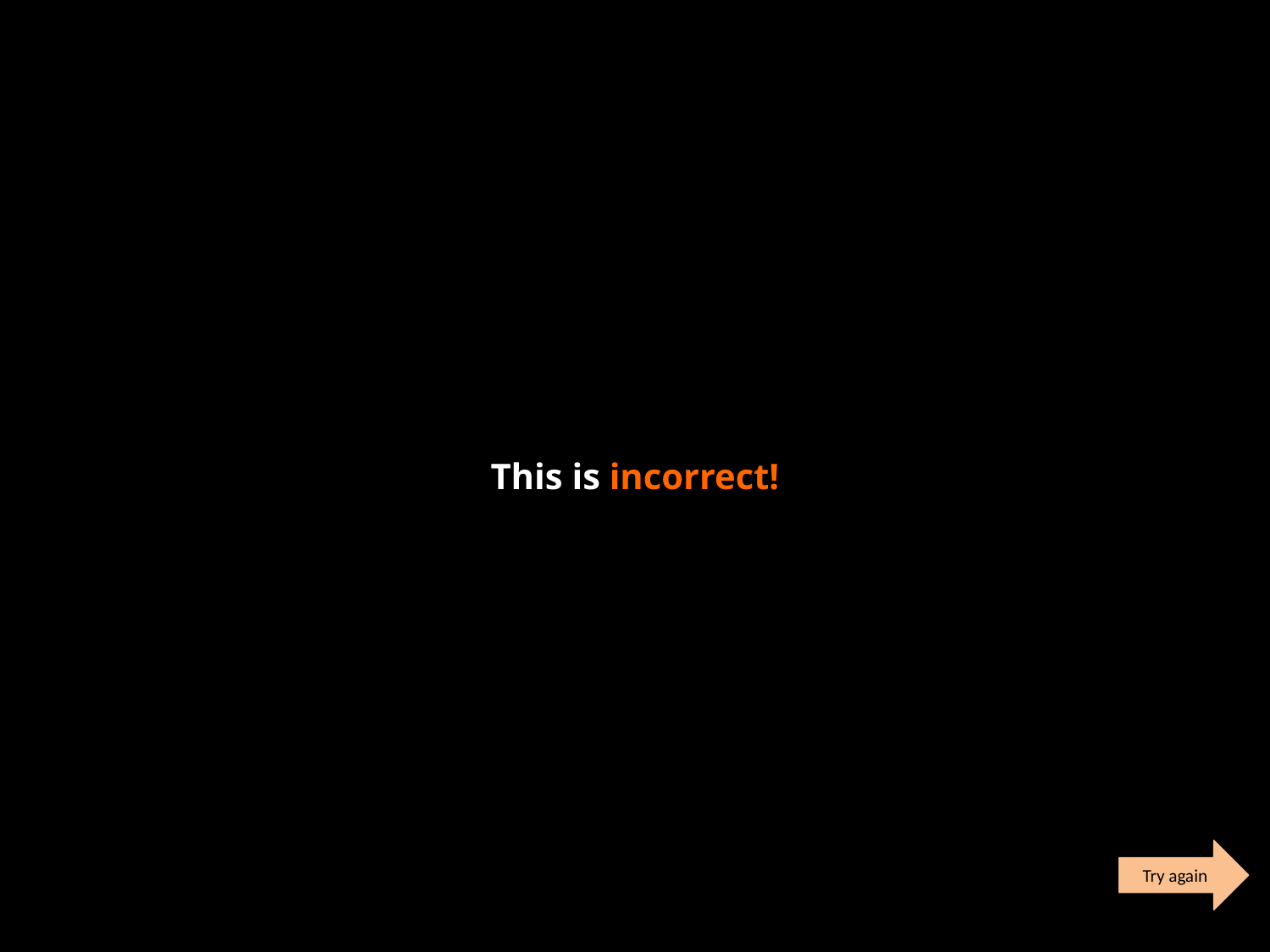

This is incorrect!
Try again

## Slide 77
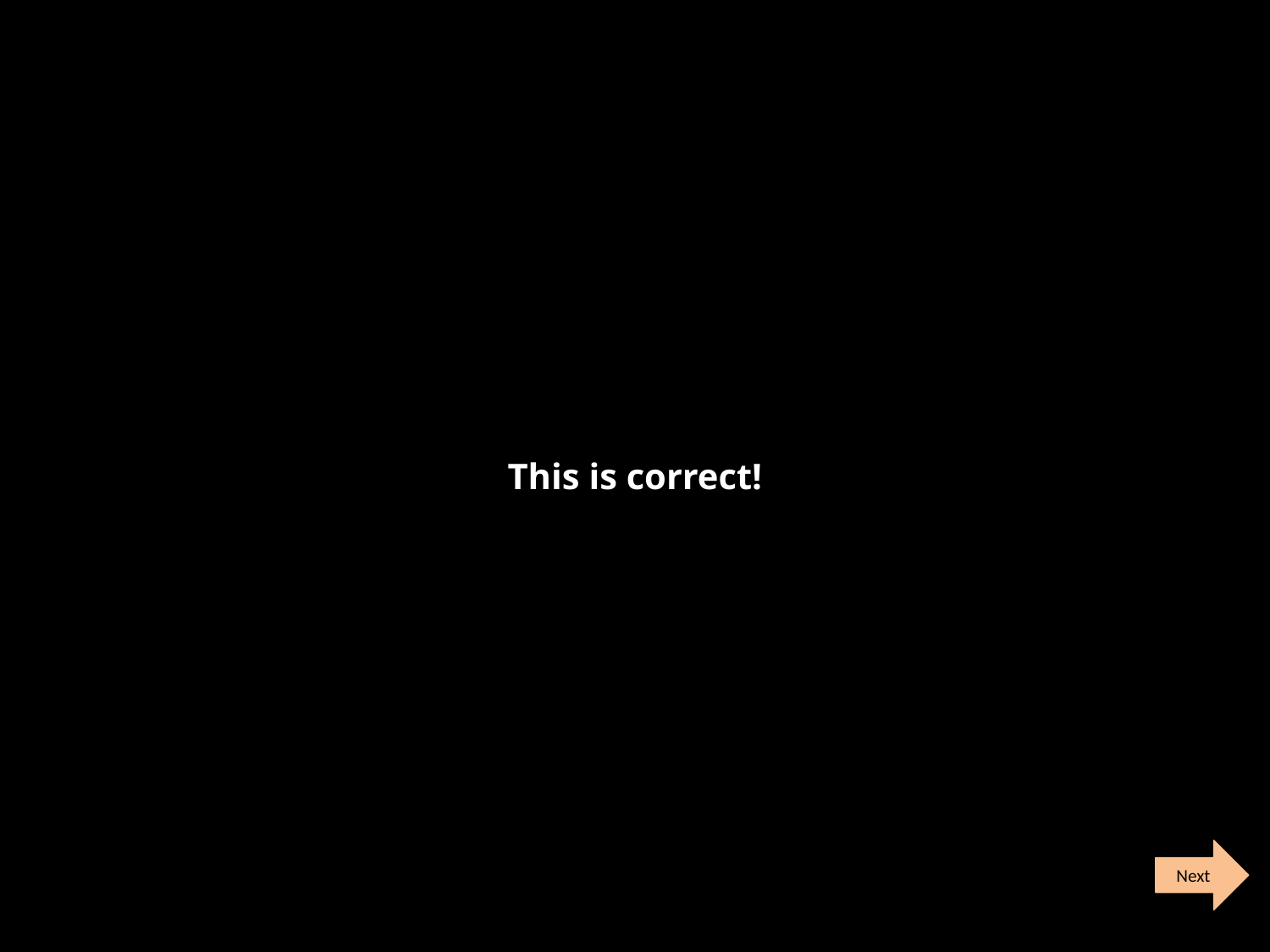

This is correct!
Next

## Slide 78
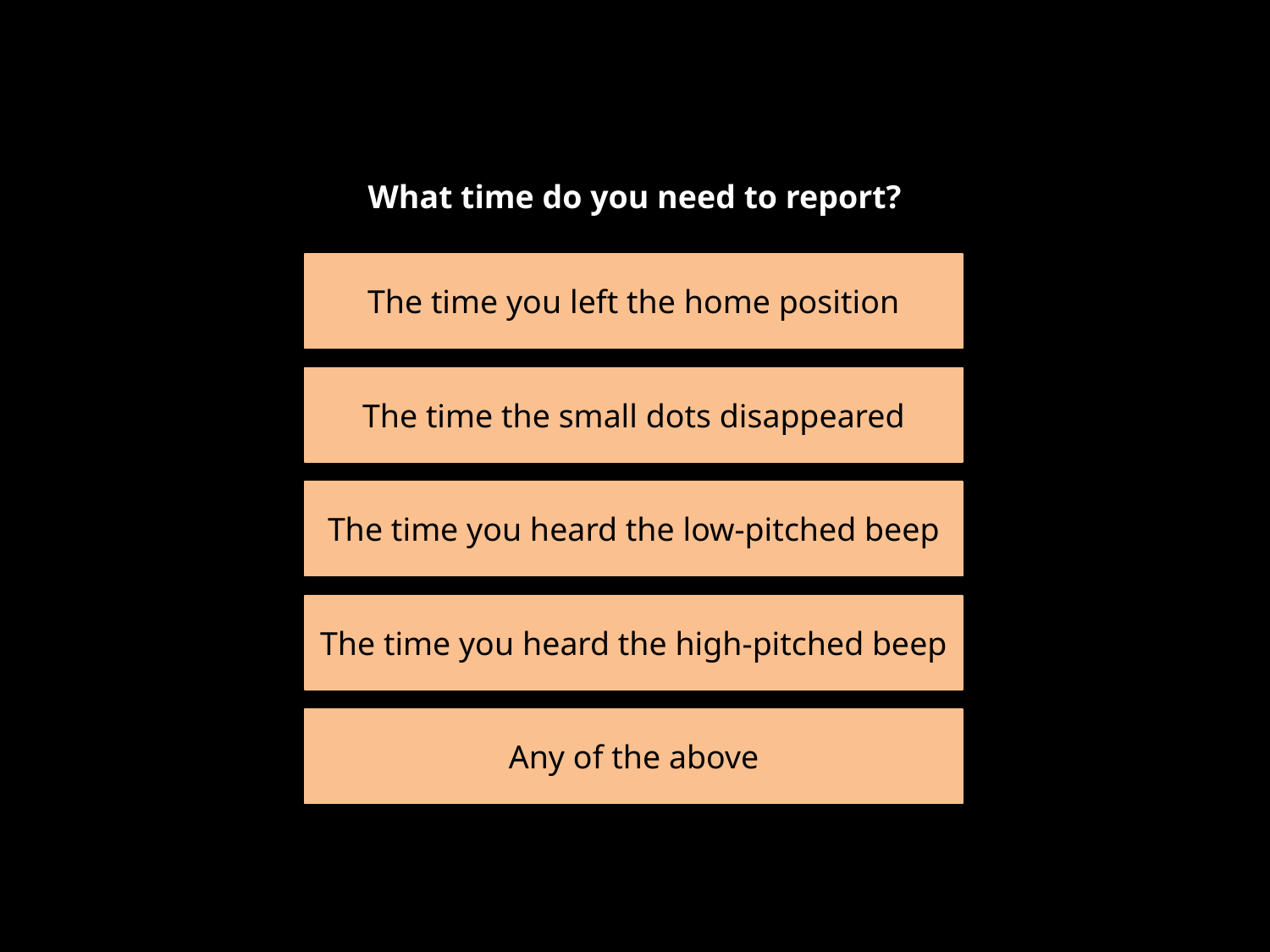

What time do you need to report?
The time you left the home position
The time the small dots disappeared
The time you heard the low-pitched beep
The time you heard the high-pitched beep
Any of the above

## Slide 79
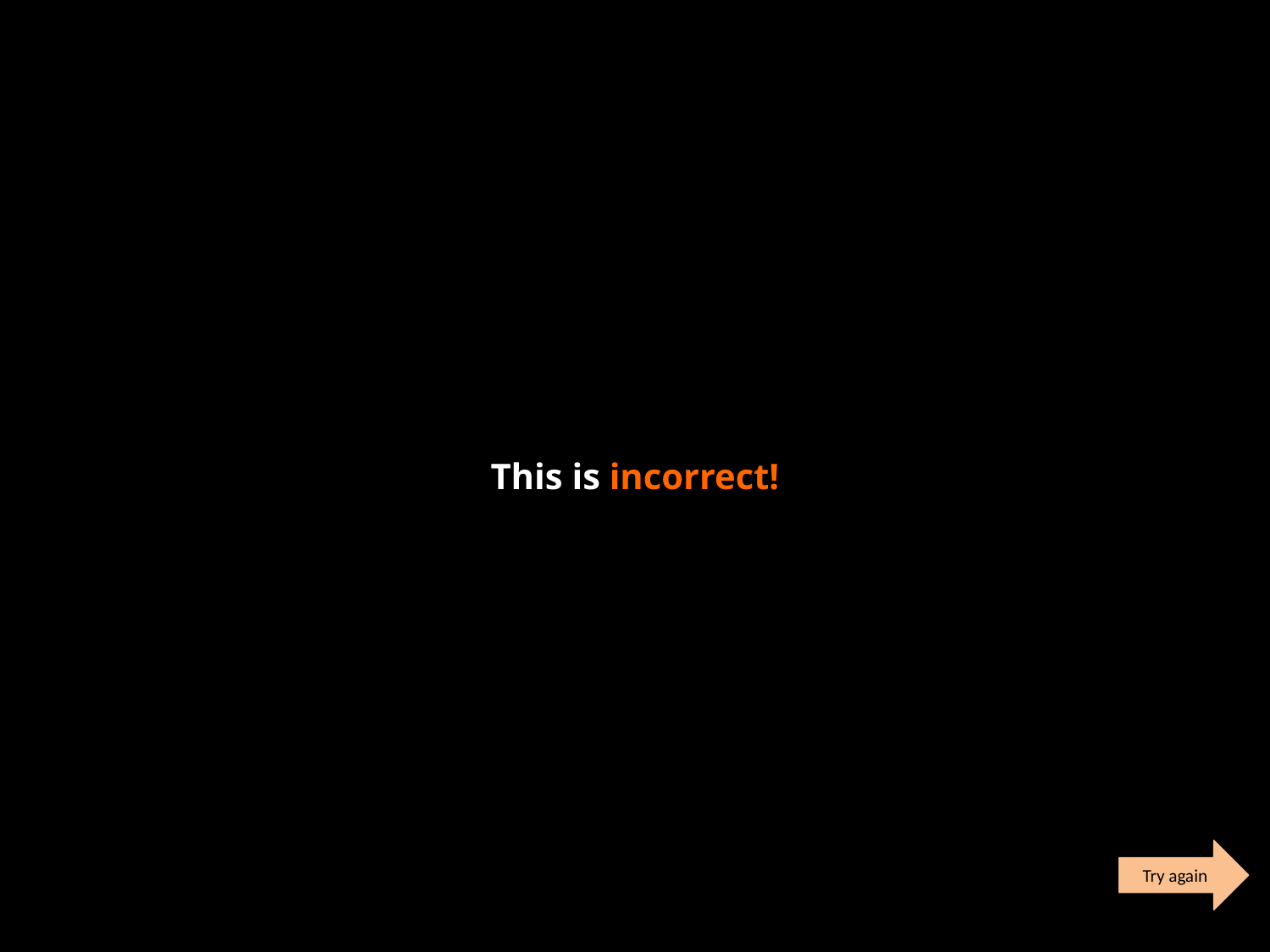

This is incorrect!
Try again

## Slide 80
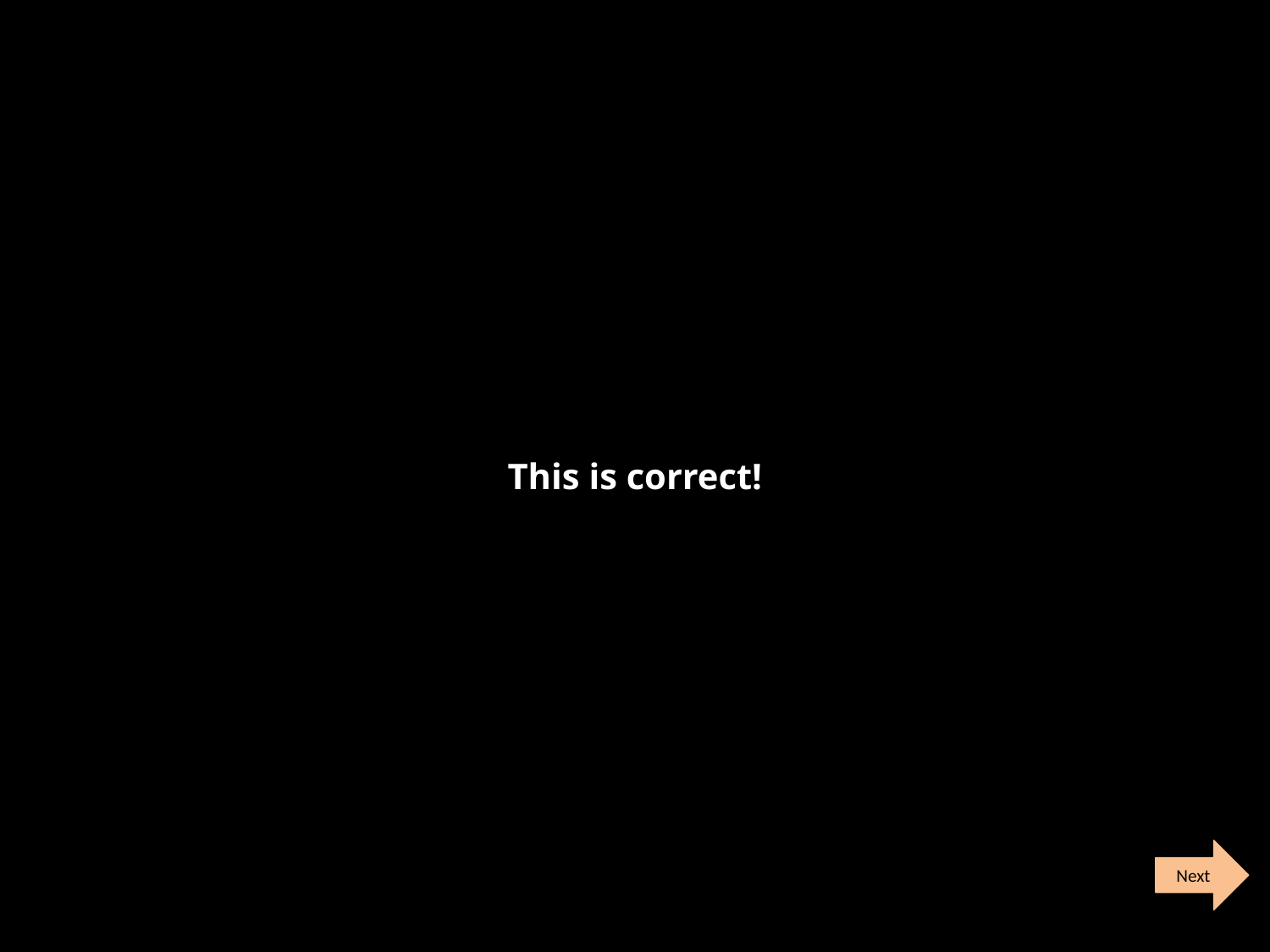

This is correct!
Next

## Slide 81
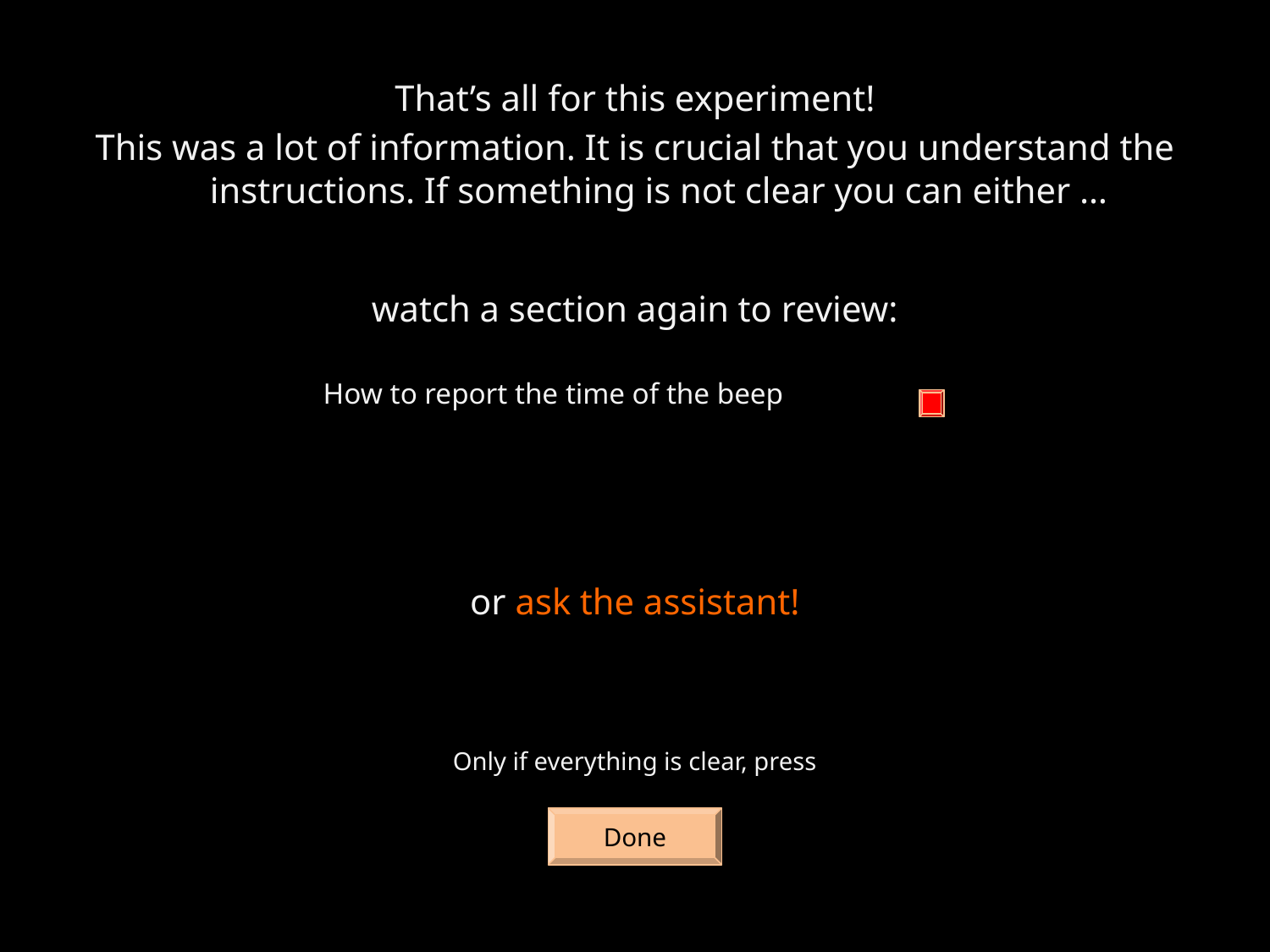

That’s all for this experiment!
This was a lot of information. It is crucial that you understand the instructions. If something is not clear you can either …
watch a section again to review:
How to report the time of the beep
or ask the assistant!
Only if everything is clear, press
Done

## Slide 82
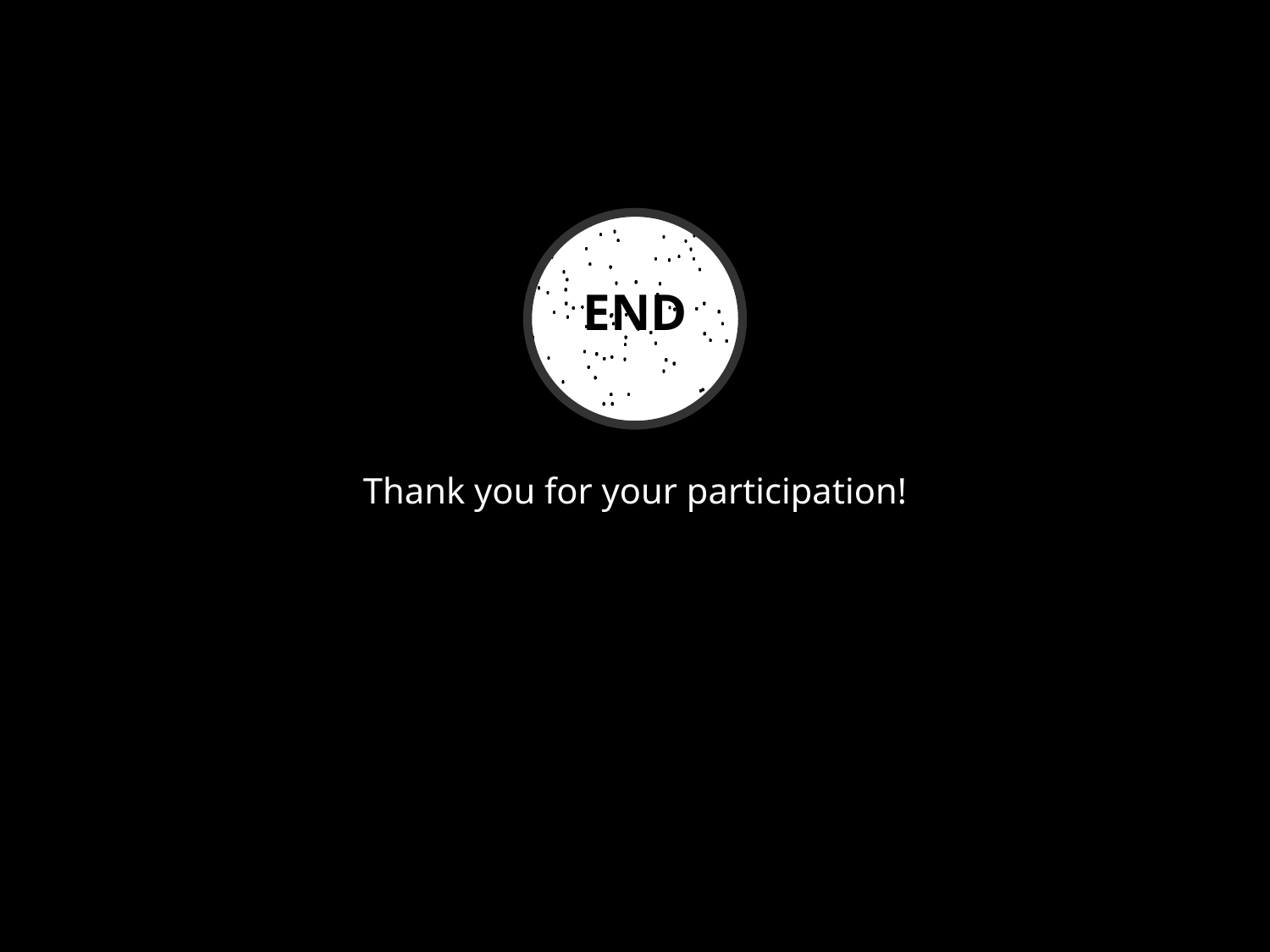

# END
Thank you for your participation!

## Slide 83
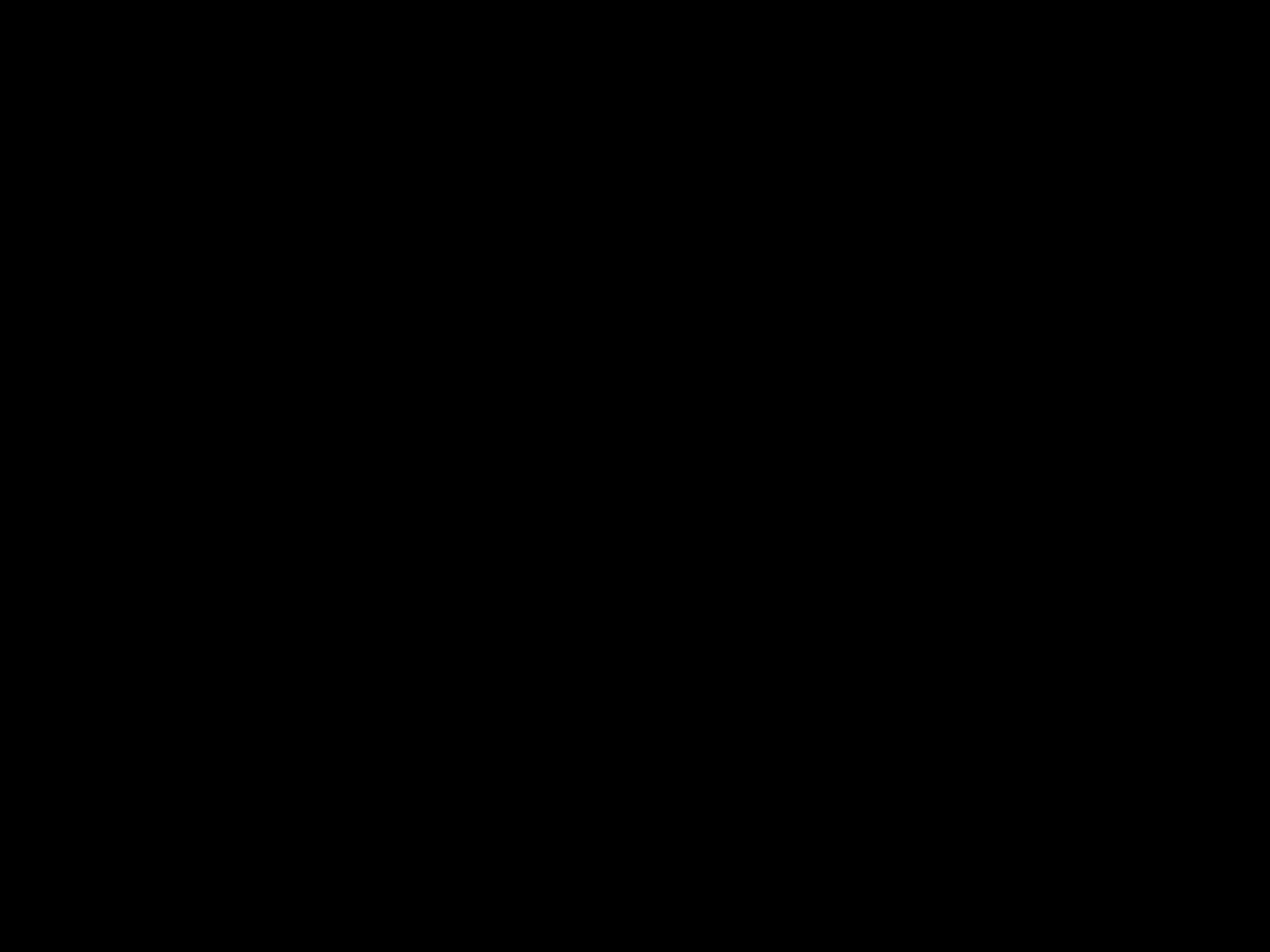

#

## Slide 84
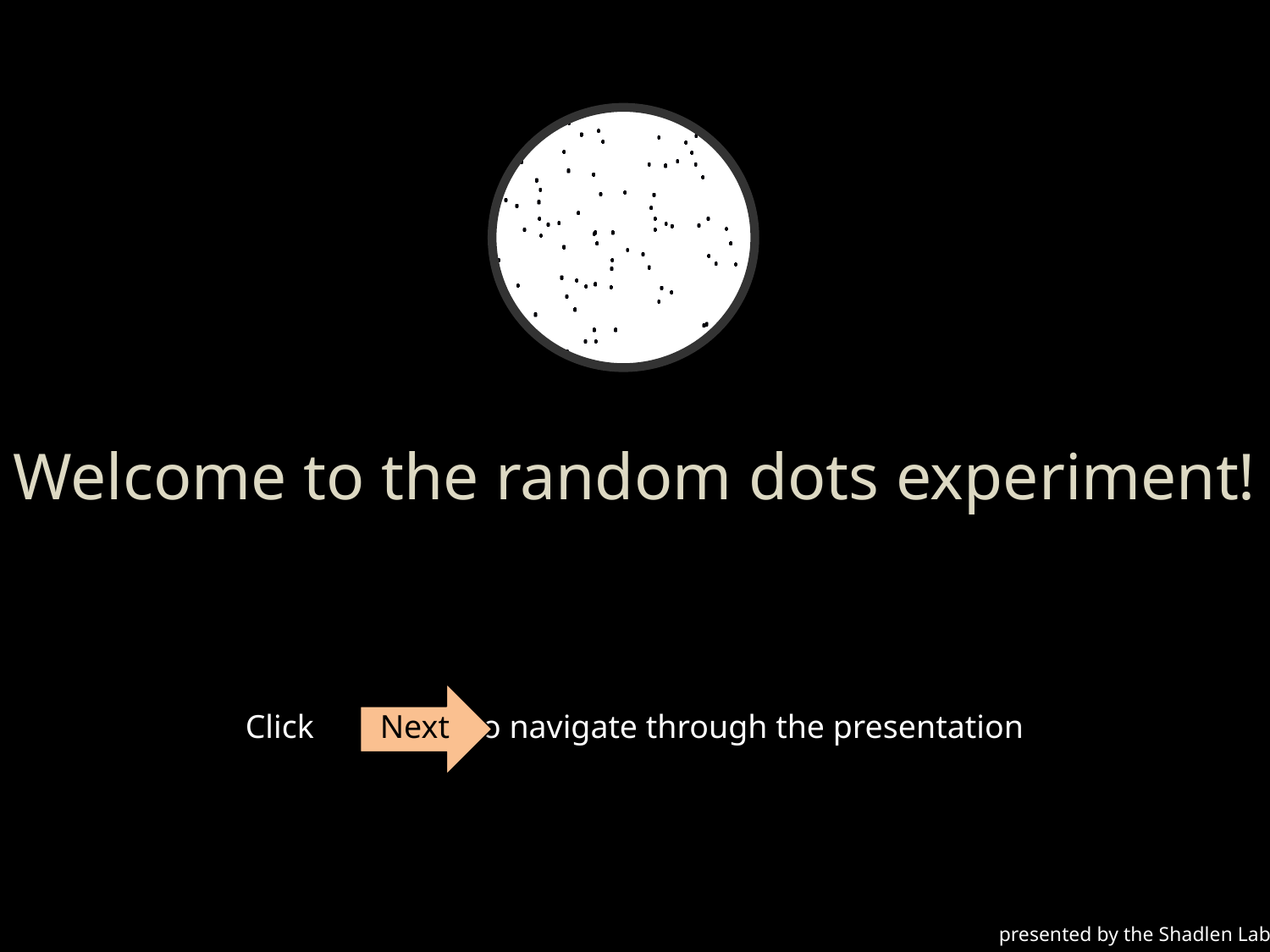

# Welcome to the random dots experiment!
Next
Click to navigate through the presentation
presented by the Shadlen Lab

## Slide 85
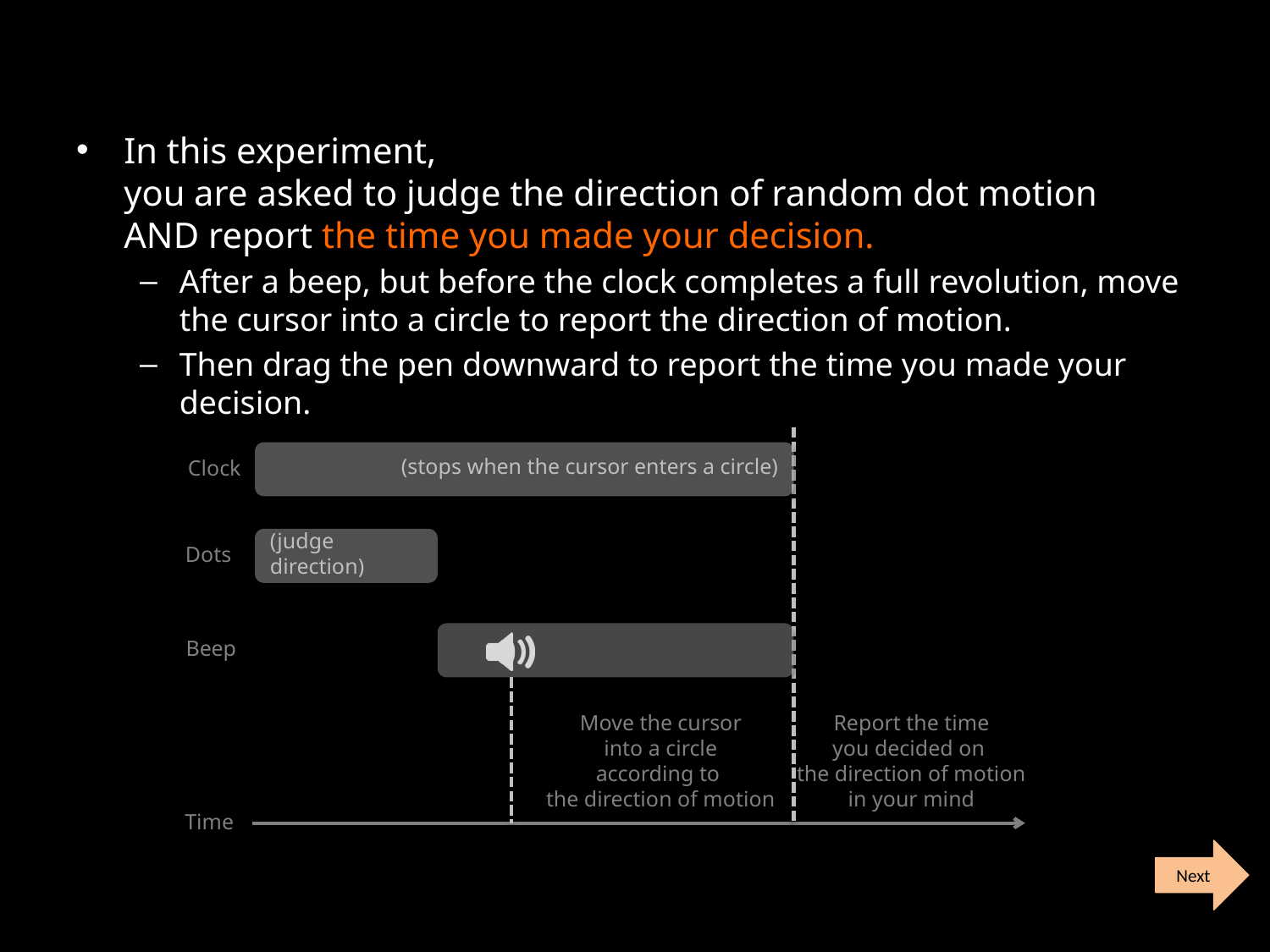

In this experiment,
you are asked to judge the direction of random dot motion
AND report the time you made your decision.
After a beep, but before the clock completes a full revolution, move the cursor into a circle to report the direction of motion.
Then drag the pen downward to report the time you made your decision.
(stops when the cursor enters a circle)
Clock
(judge direction)
Dots
Beep
Move the cursor
into a circle
according to
the direction of motion
Report the time
you decided on
the direction of motion
in your mind
Time
Next

## Slide 86
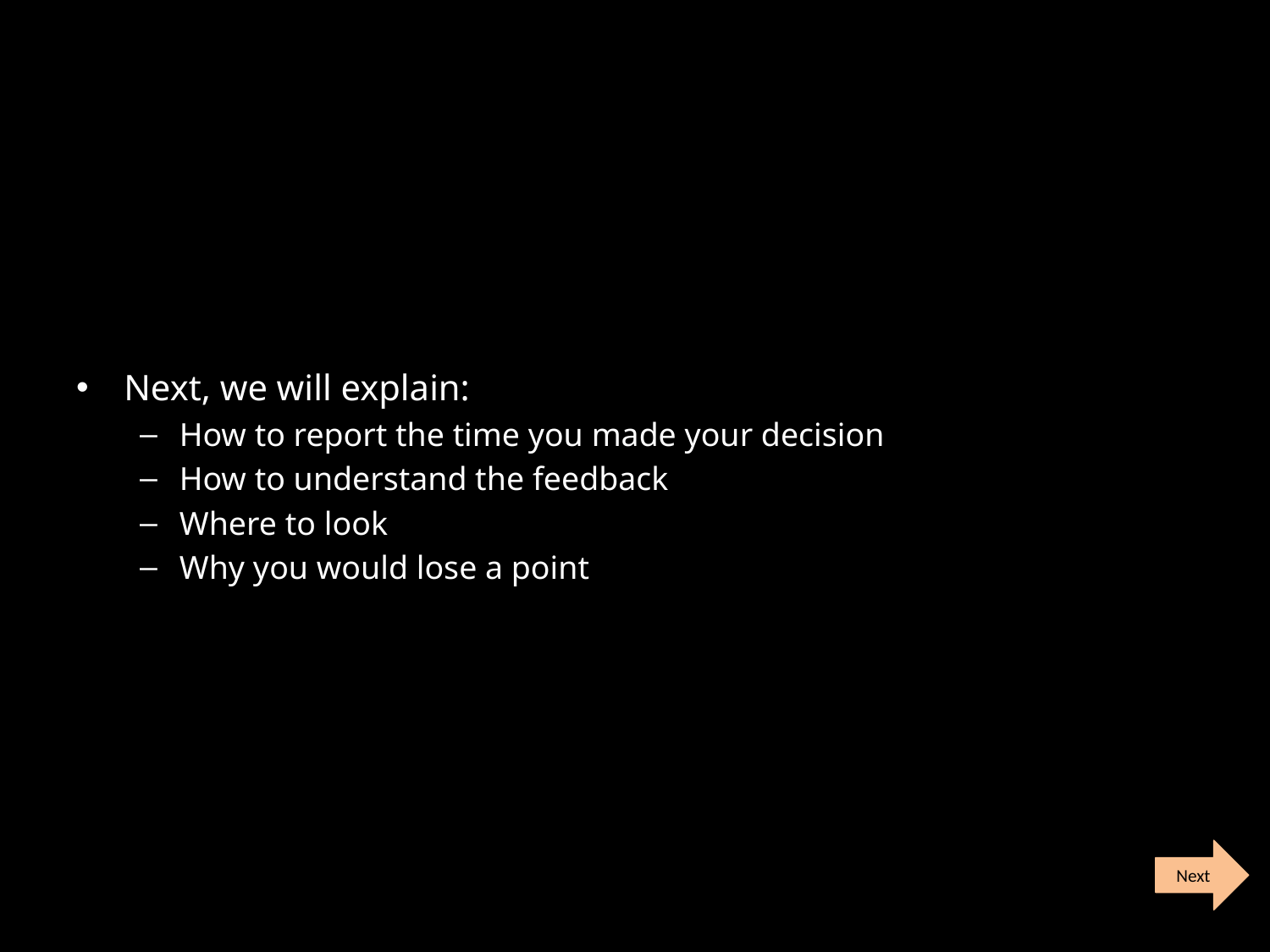

Next, we will explain:
How to report the time you made your decision
How to understand the feedback
Where to look
Why you would lose a point
Next

## Slide 87
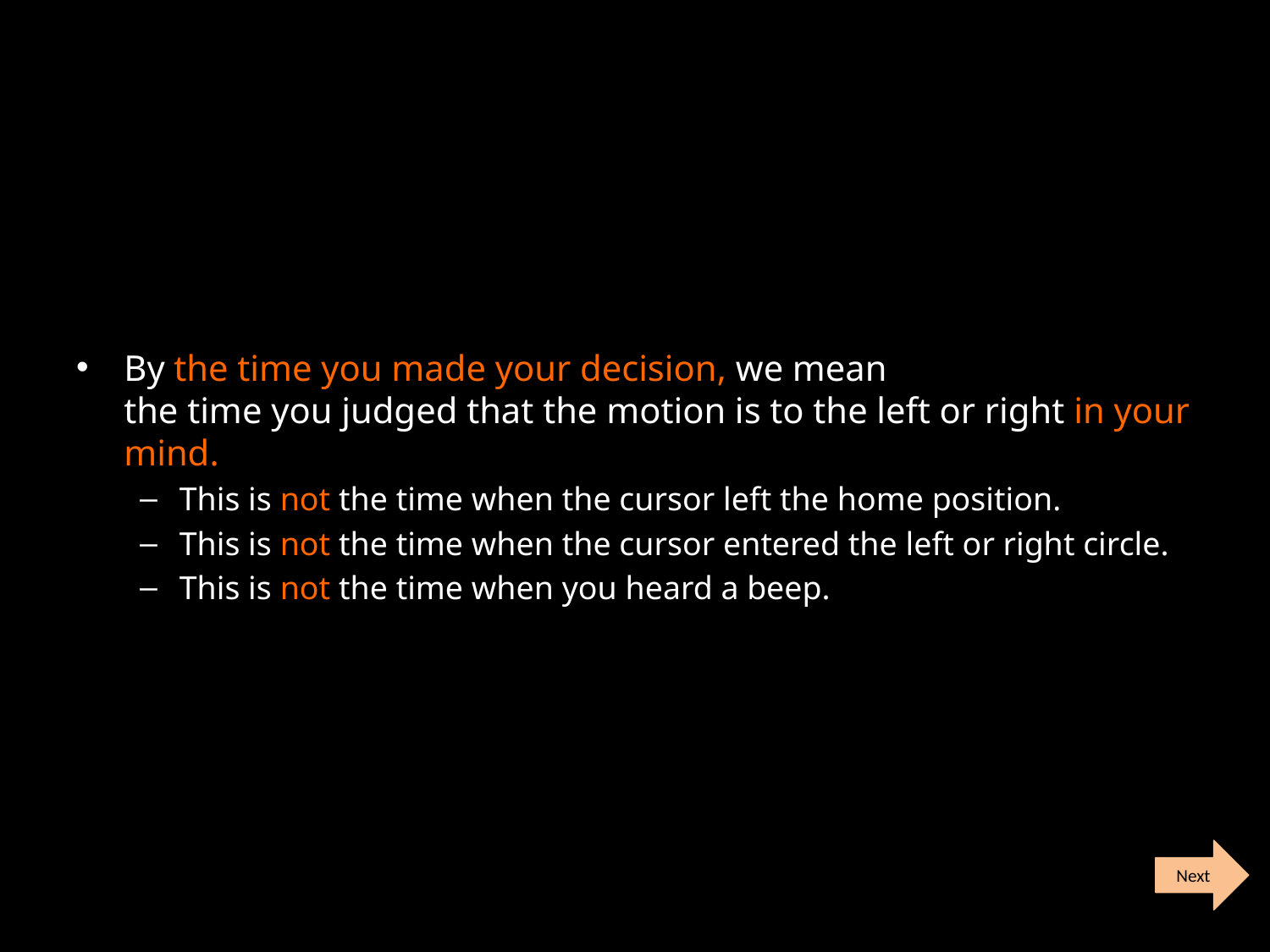

By the time you made your decision, we mean
the time you judged that the motion is to the left or right in your mind.
This is not the time when the cursor left the home position.
This is not the time when the cursor entered the left or right circle.
This is not the time when you heard a beep.
Next

## Slide 88
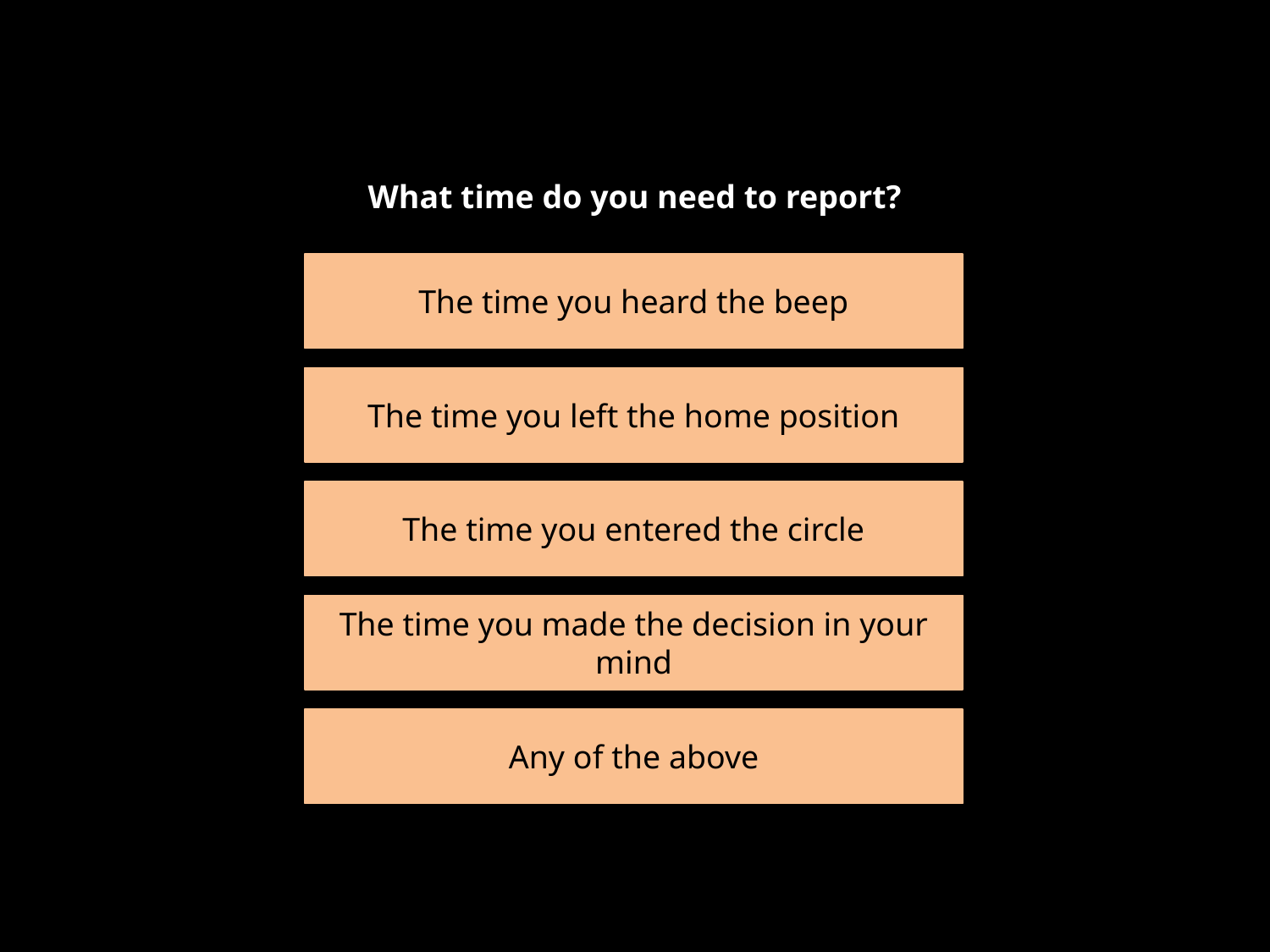

What time do you need to report?
The time you heard the beep
The time you left the home position
The time you entered the circle
The time you made the decision in your mind
Any of the above

## Slide 89
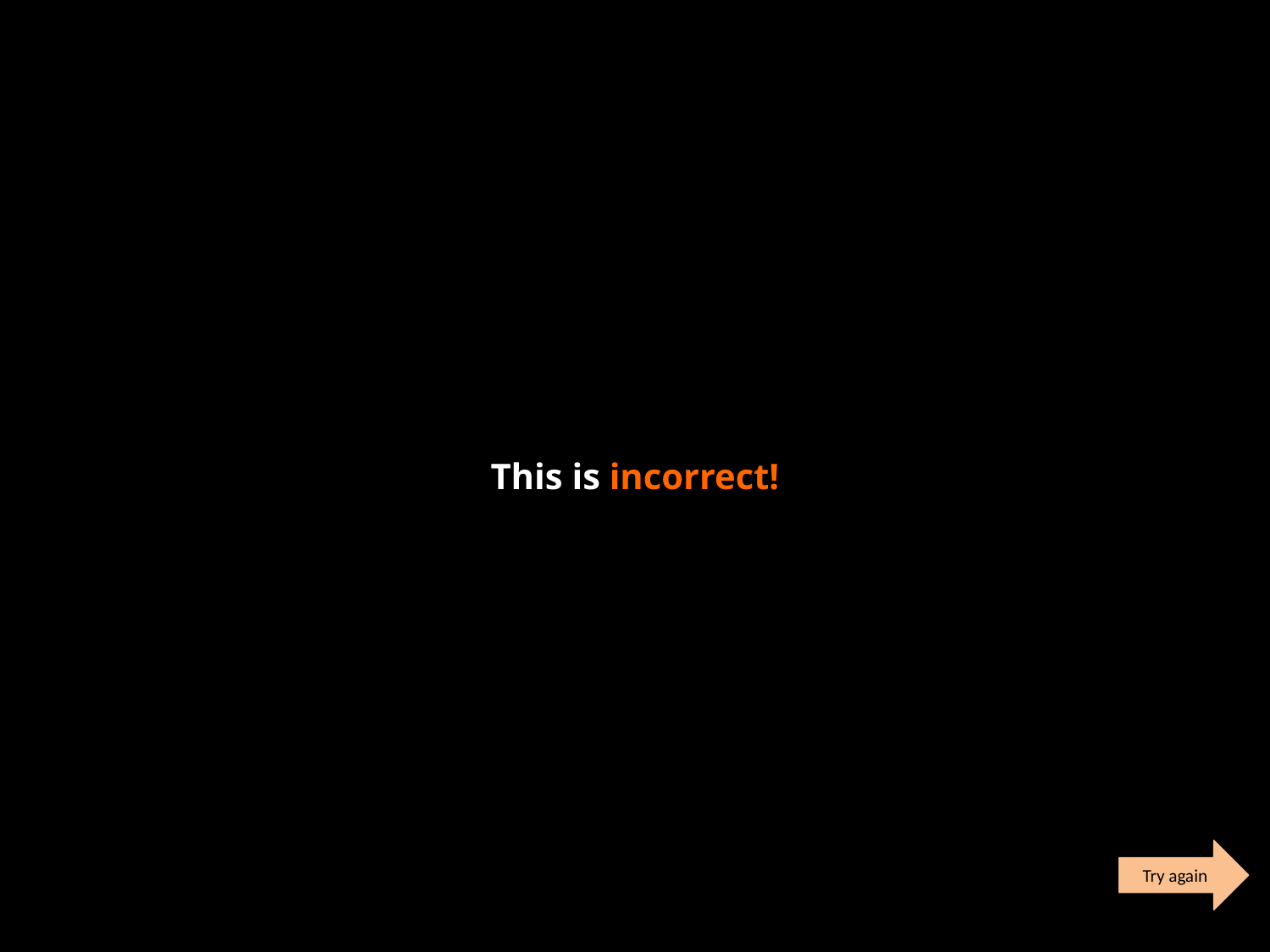

This is incorrect!
Try again

## Slide 90
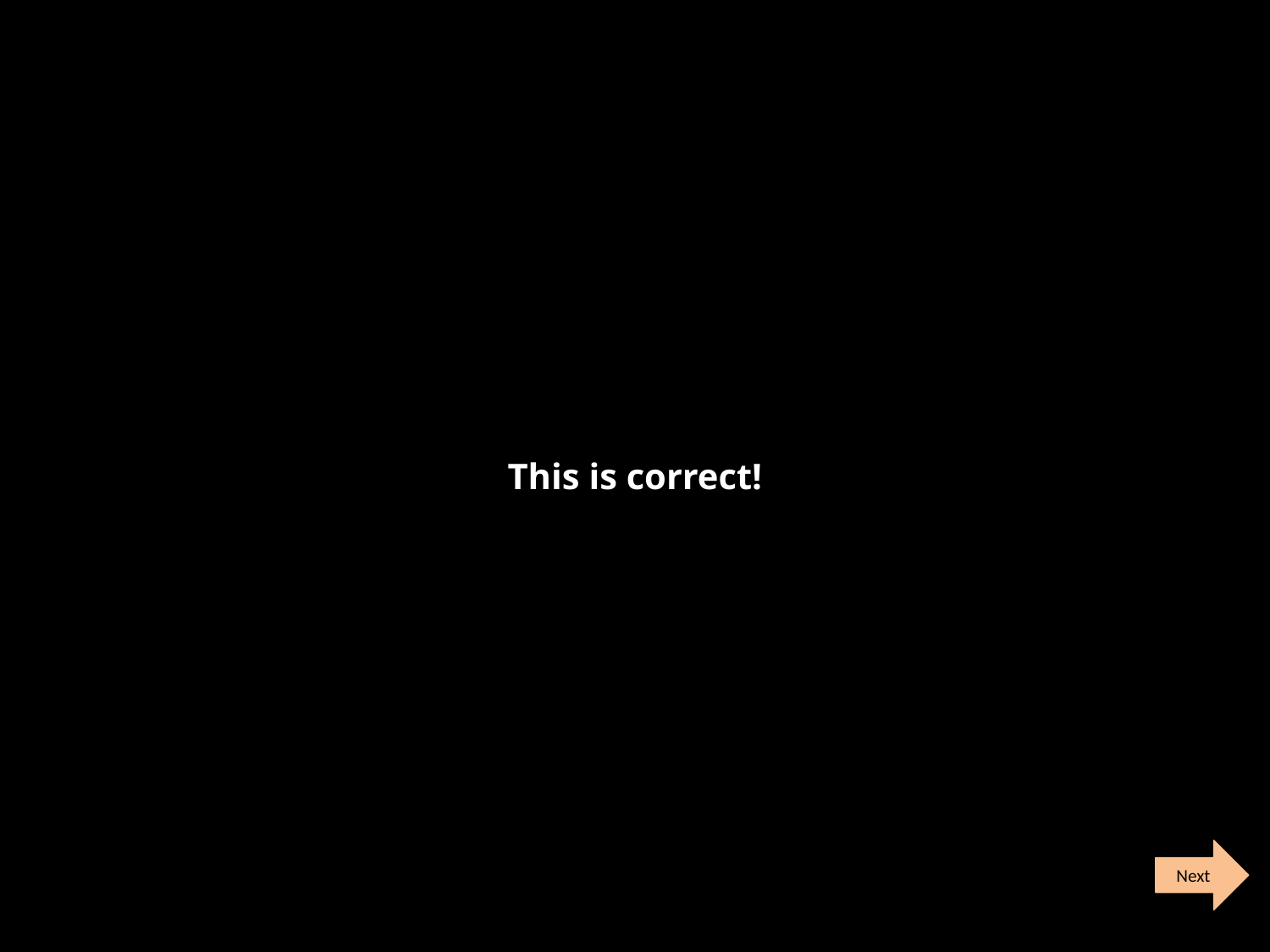

This is correct!
Next

## Slide 91
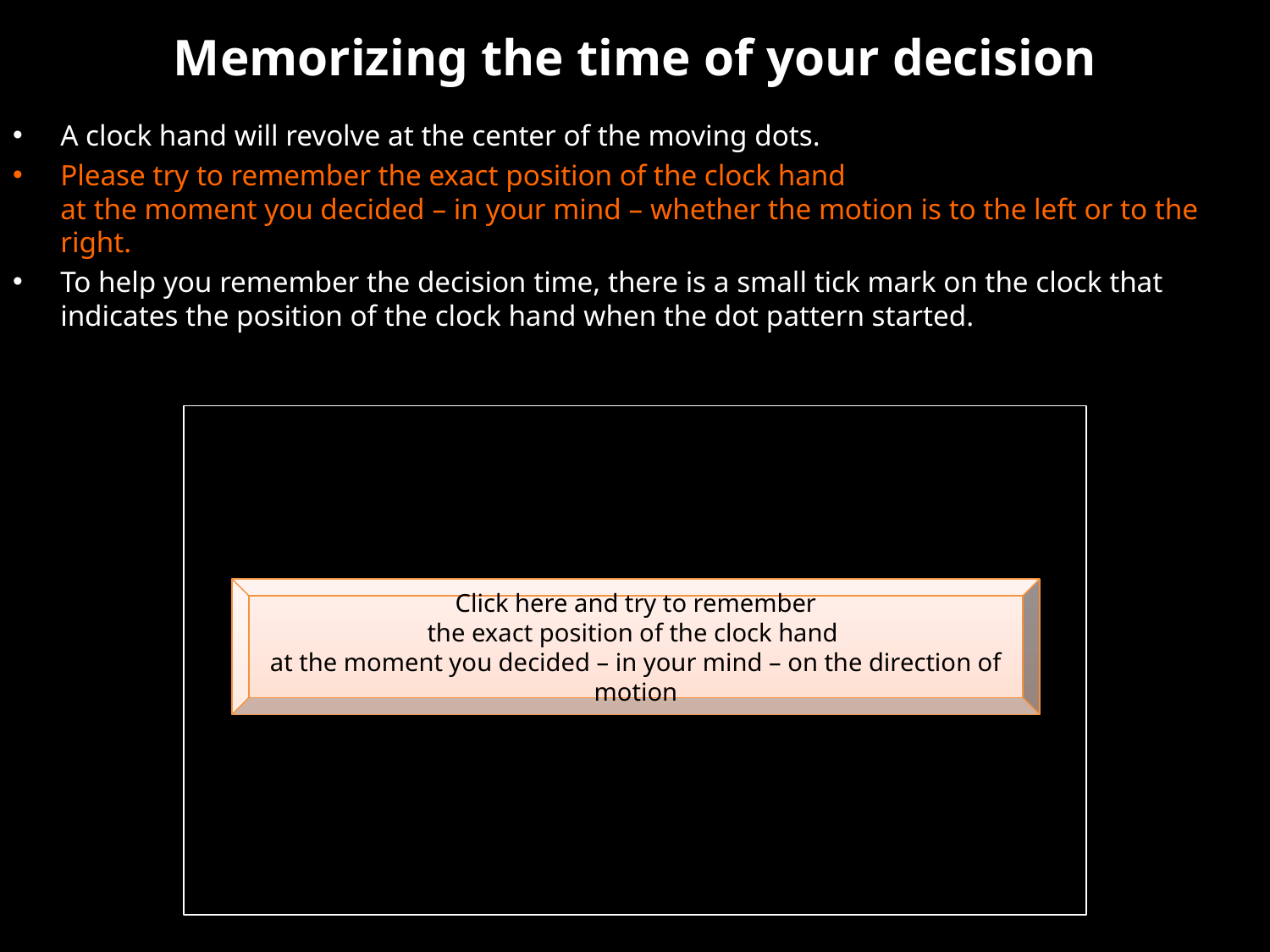

# Memorizing the time of your decision
A clock hand will revolve at the center of the moving dots.
Please try to remember the exact position of the clock hand
at the moment you decided – in your mind – whether the motion is to the left or to the right.
To help you remember the decision time, there is a small tick mark on the clock that indicates the position of the clock hand when the dot pattern started.
Click here and try to remember
the exact position of the clock hand
at the moment you decided – in your mind – on the direction of motion

## Slide 92
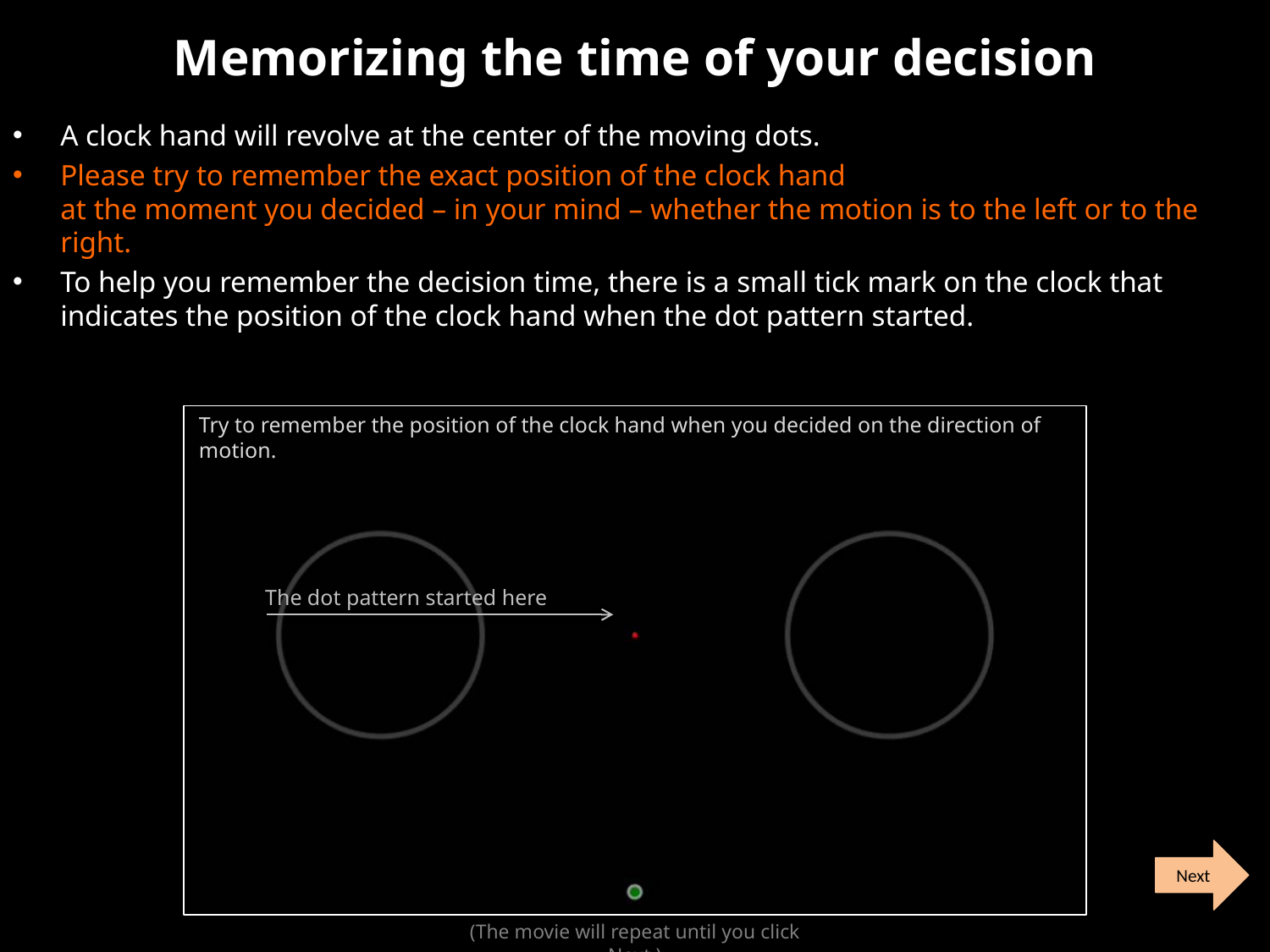

# Memorizing the time of your decision
A clock hand will revolve at the center of the moving dots.
Please try to remember the exact position of the clock hand
at the moment you decided – in your mind – whether the motion is to the left or to the right.
To help you remember the decision time, there is a small tick mark on the clock that indicates the position of the clock hand when the dot pattern started.
Try to remember the position of the clock hand when you decided on the direction of motion.
The dot pattern started here

## Slide 93
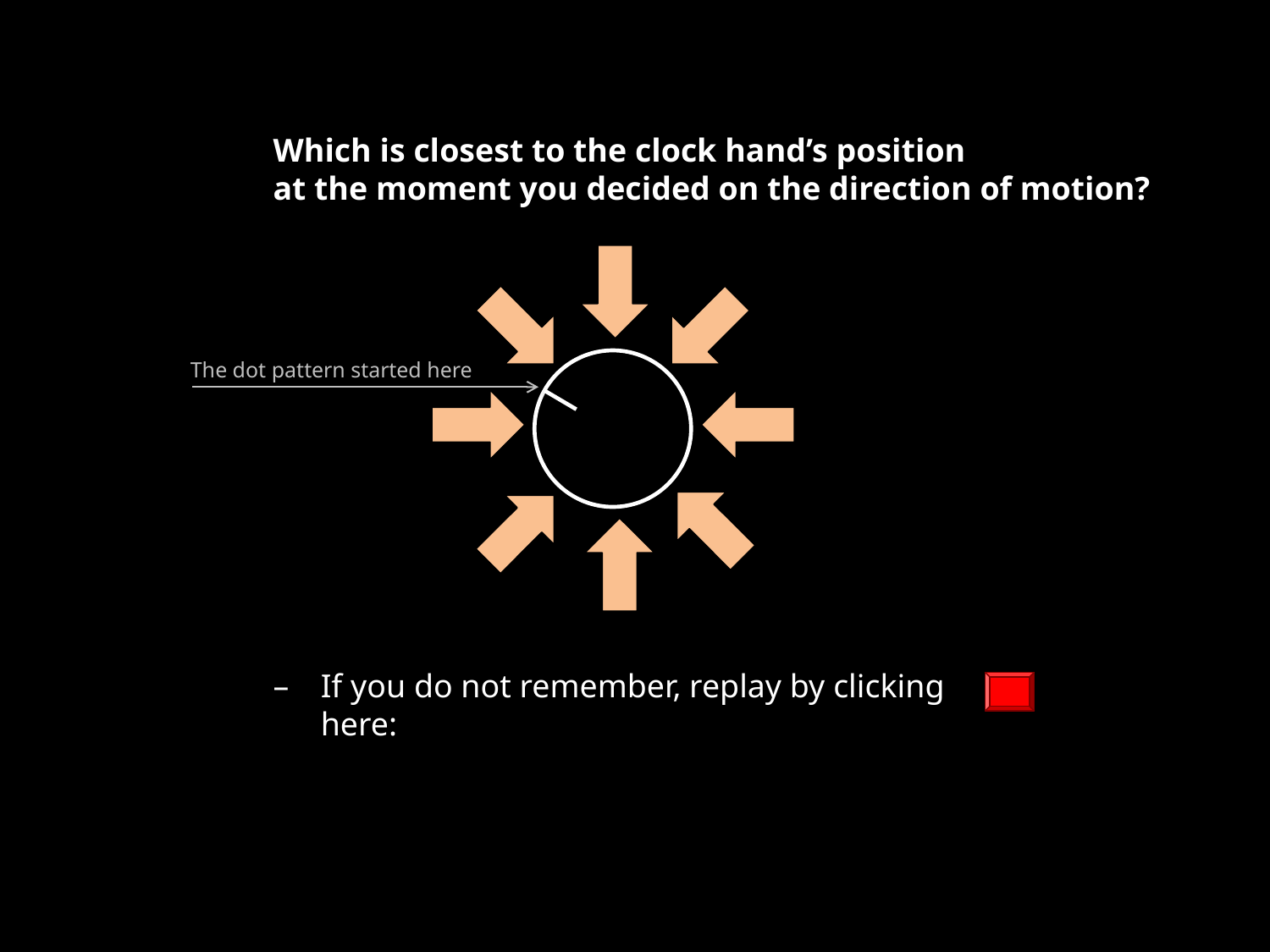

# Which is closest to the clock hand’s position at the moment you decided on the direction of motion?
The dot pattern started here
If you do not remember, replay by clicking here:

## Slide 94
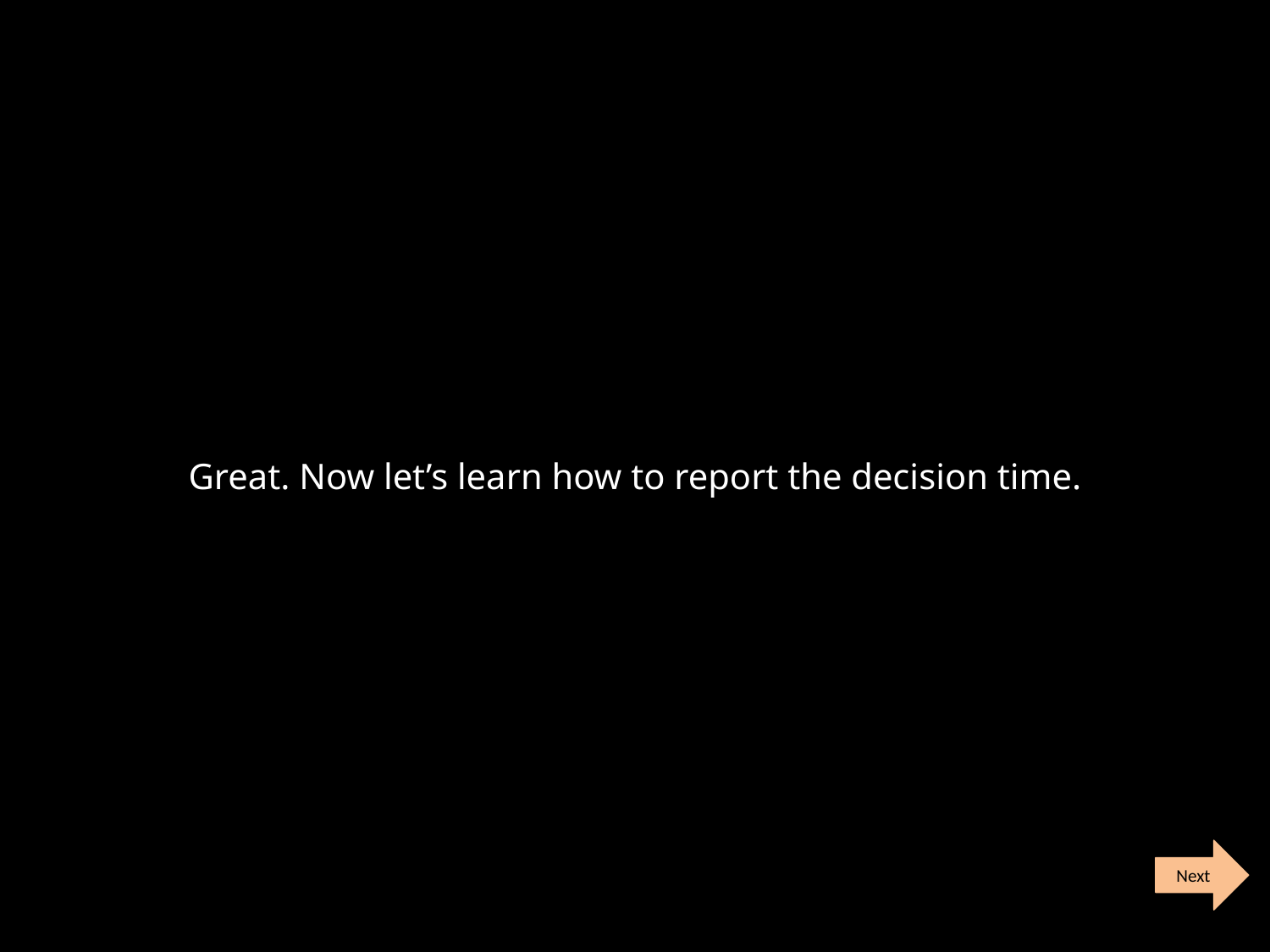

Great. Now let’s learn how to report the decision time.
Next

## Slide 95
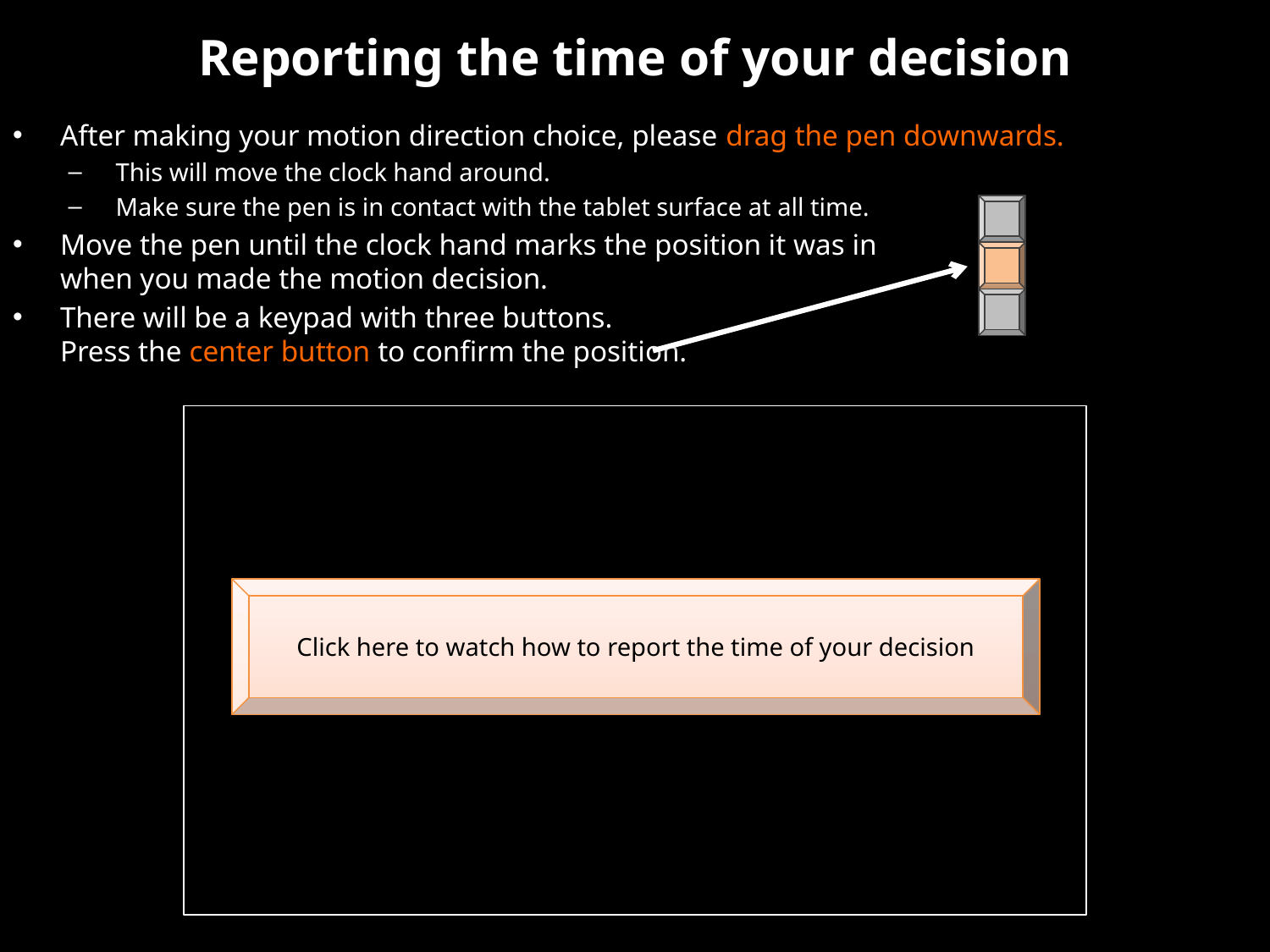

# Reporting the time of your decision
After making your motion direction choice, please drag the pen downwards.
This will move the clock hand around.
Make sure the pen is in contact with the tablet surface at all time.
Move the pen until the clock hand marks the position it was in
when you made the motion decision.
There will be a keypad with three buttons.
Press the center button to confirm the position.
Click here to watch how to report the time of your decision

## Slide 96
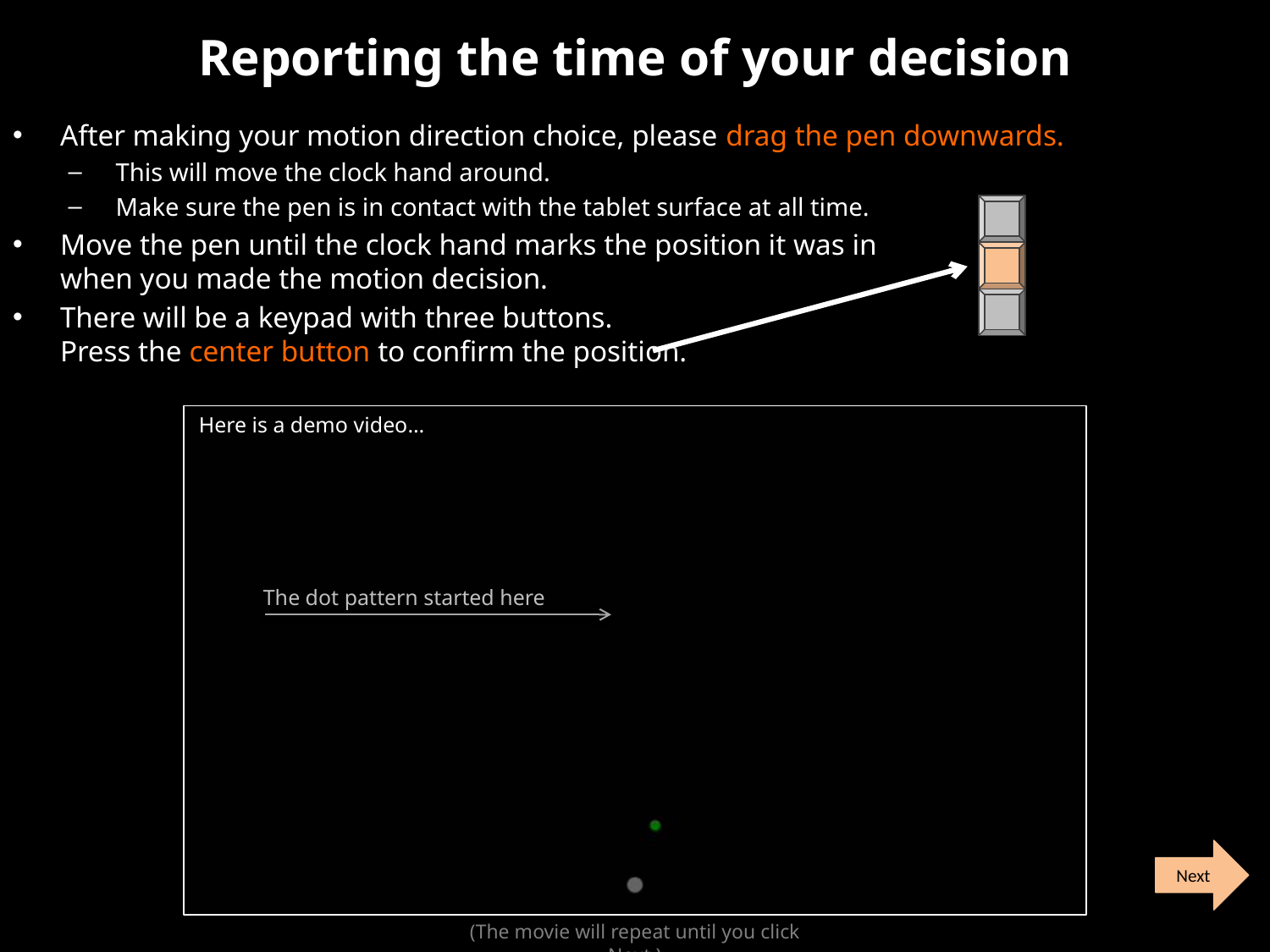

# Reporting the time of your decision
After making your motion direction choice, please drag the pen downwards.
This will move the clock hand around.
Make sure the pen is in contact with the tablet surface at all time.
Move the pen until the clock hand marks the position it was in
when you made the motion decision.
There will be a keypad with three buttons.
Press the center button to confirm the position.
Here is a demo video…
The dot pattern started here

## Slide 97
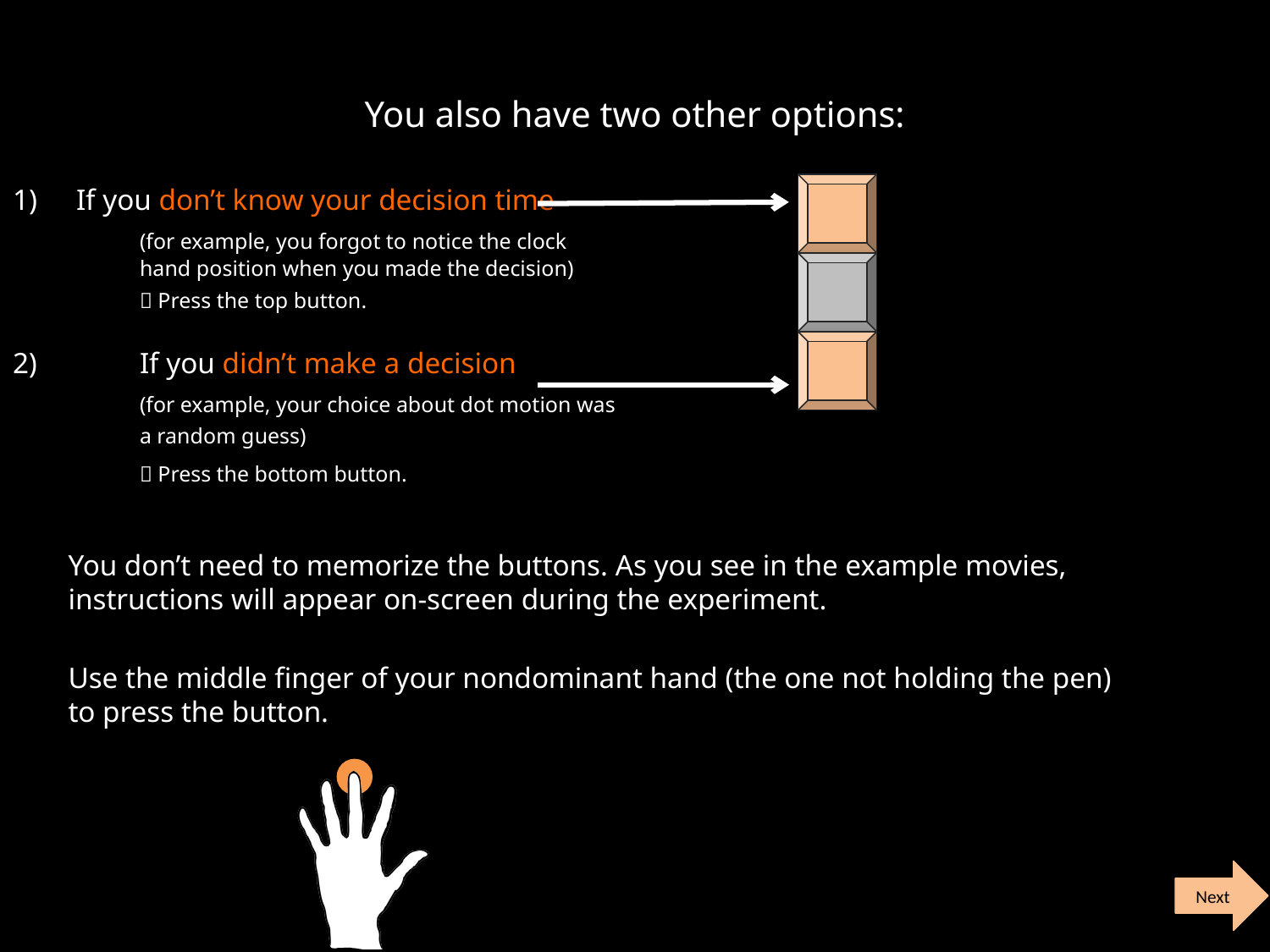

You also have two other options:
If you don’t know your decision time
	(for example, you forgot to notice the clock
	hand position when you made the decision)
	 Press the top button.
2) 	If you didn’t make a decision
	(for example, your choice about dot motion was
	a random guess)
	 Press the bottom button.
You don’t need to memorize the buttons. As you see in the example movies,
instructions will appear on-screen during the experiment.
Use the middle finger of your nondominant hand (the one not holding the pen)
to press the button.
Next

## Slide 98
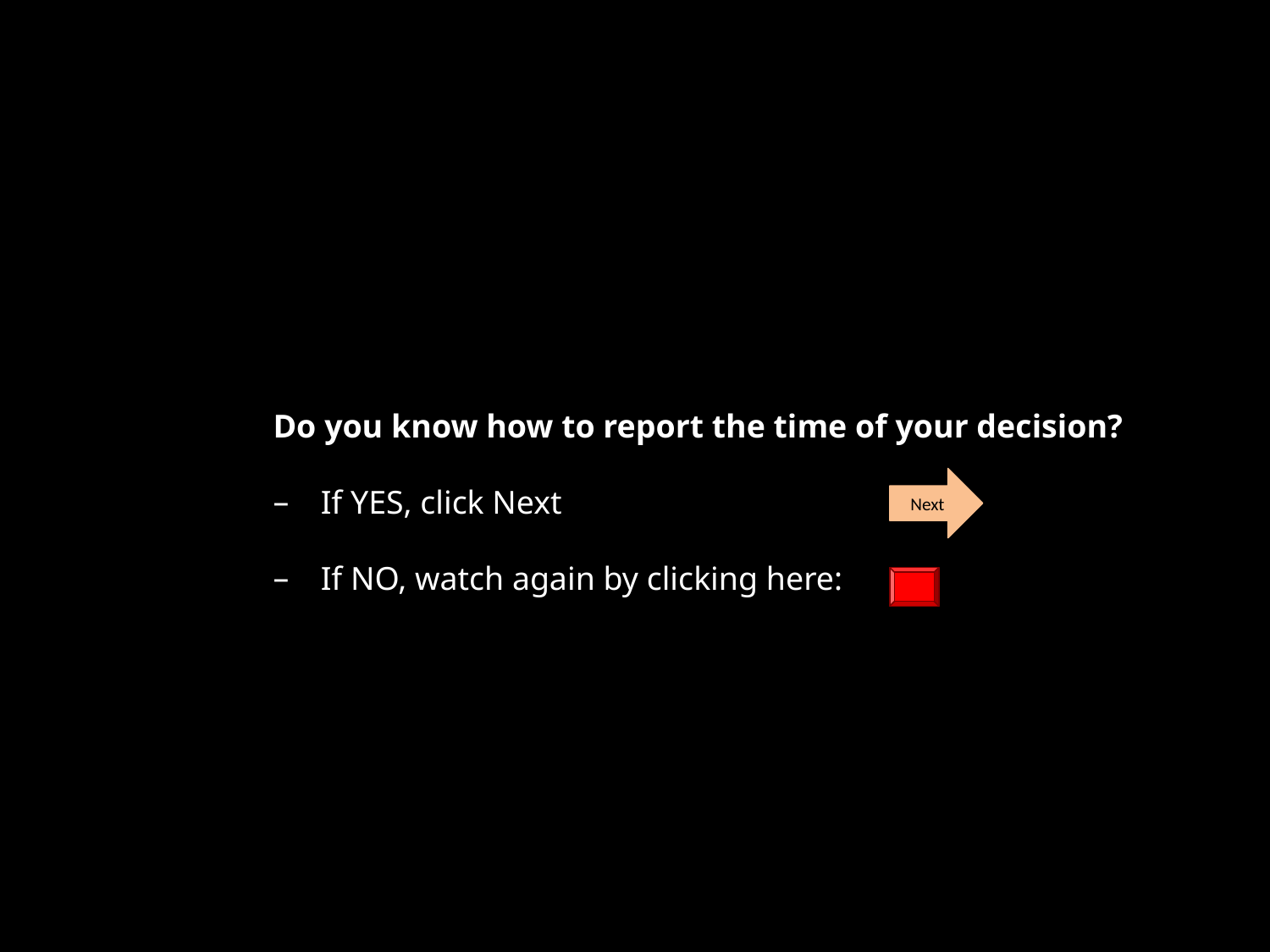

# Do you know how to report the time of your decision?
Next
If YES, click Next
If NO, watch again by clicking here:

## Slide 99
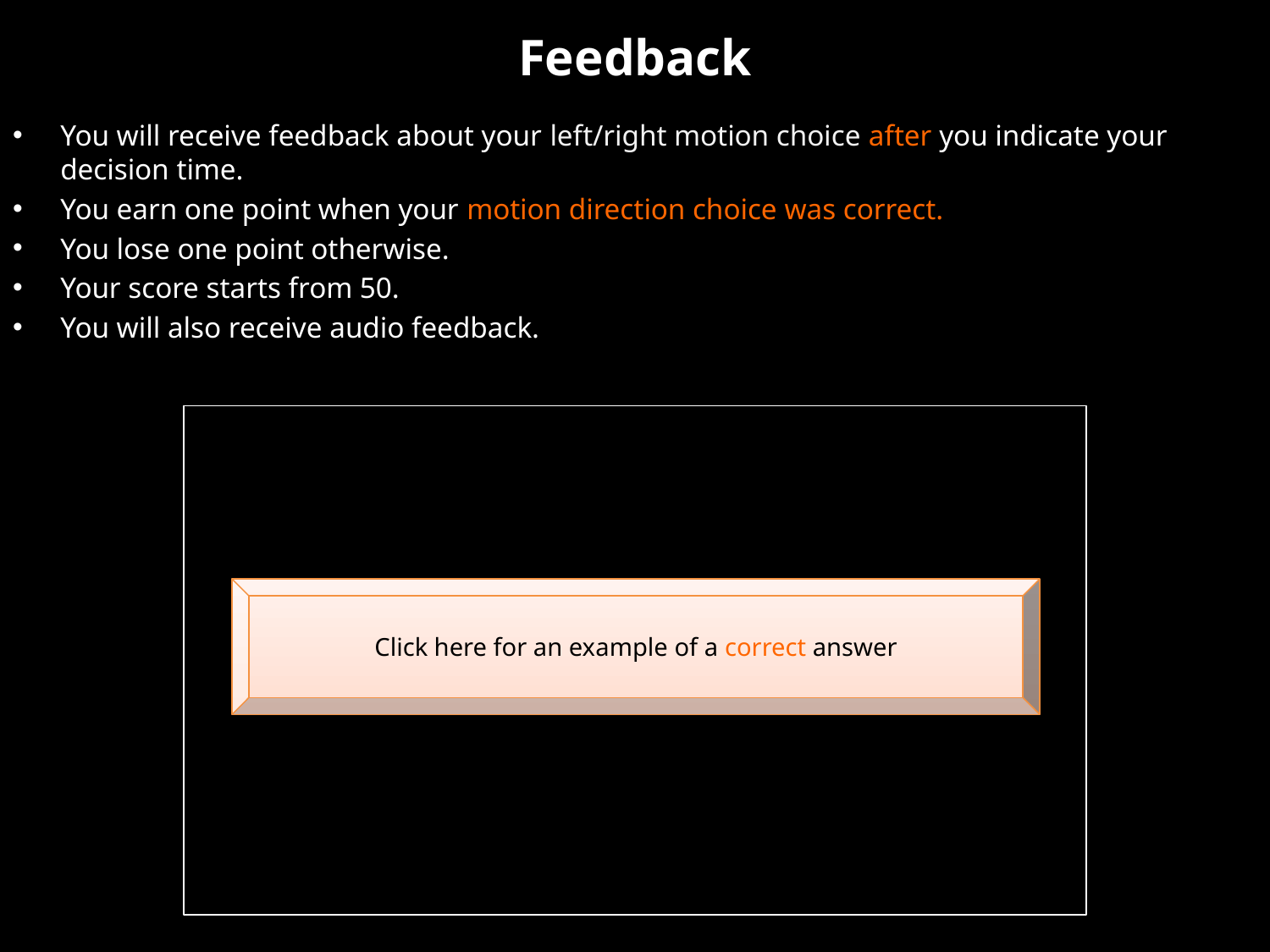

# Feedback
You will receive feedback about your left/right motion choice after you indicate your decision time.
You earn one point when your motion direction choice was correct.
You lose one point otherwise.
Your score starts from 50.
You will also receive audio feedback.
Click here for an example of a correct answer

## Slide 100
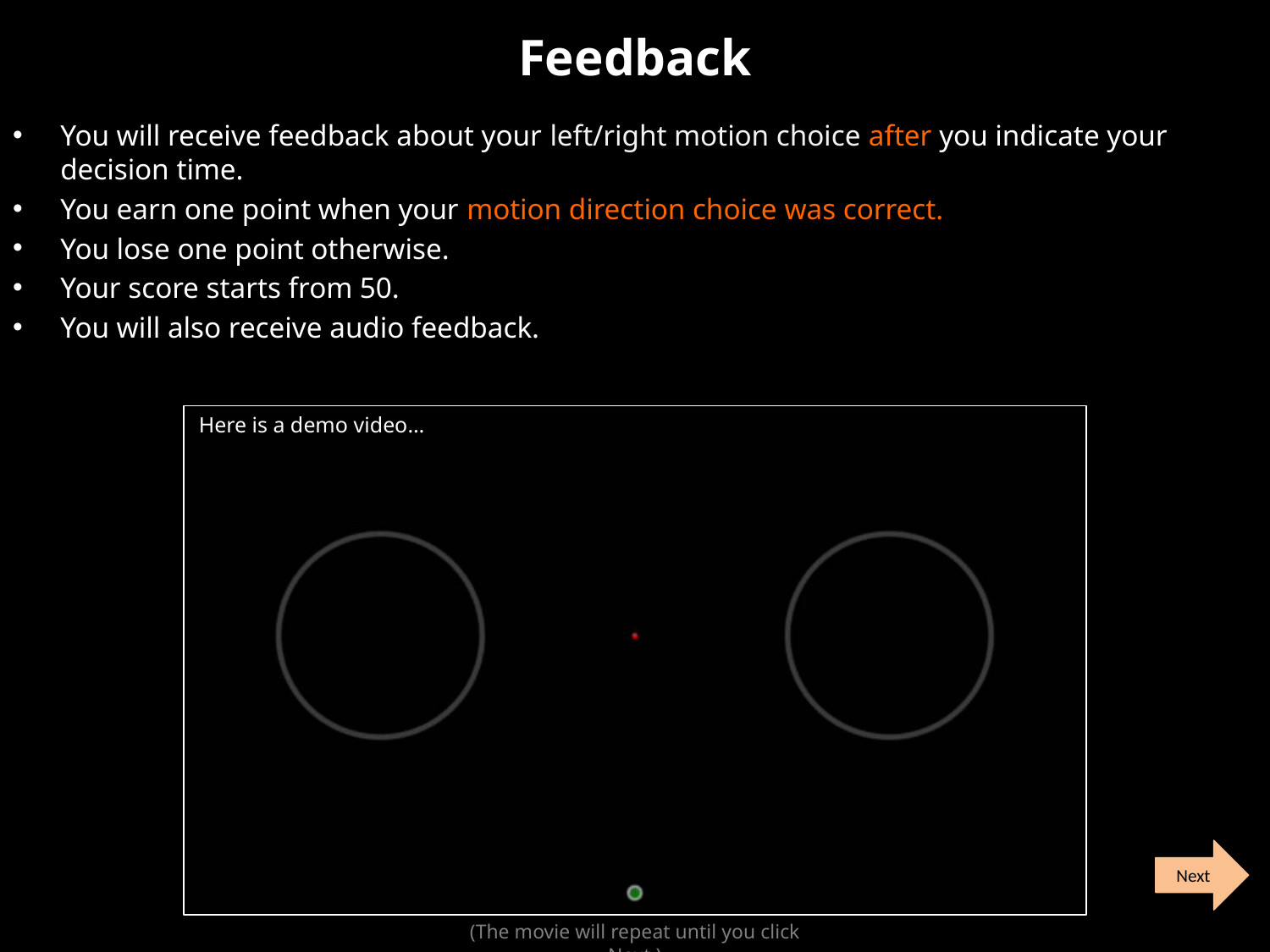

# Feedback
You will receive feedback about your left/right motion choice after you indicate your decision time.
You earn one point when your motion direction choice was correct.
You lose one point otherwise.
Your score starts from 50.
You will also receive audio feedback.
Here is a demo video…

## Slide 101
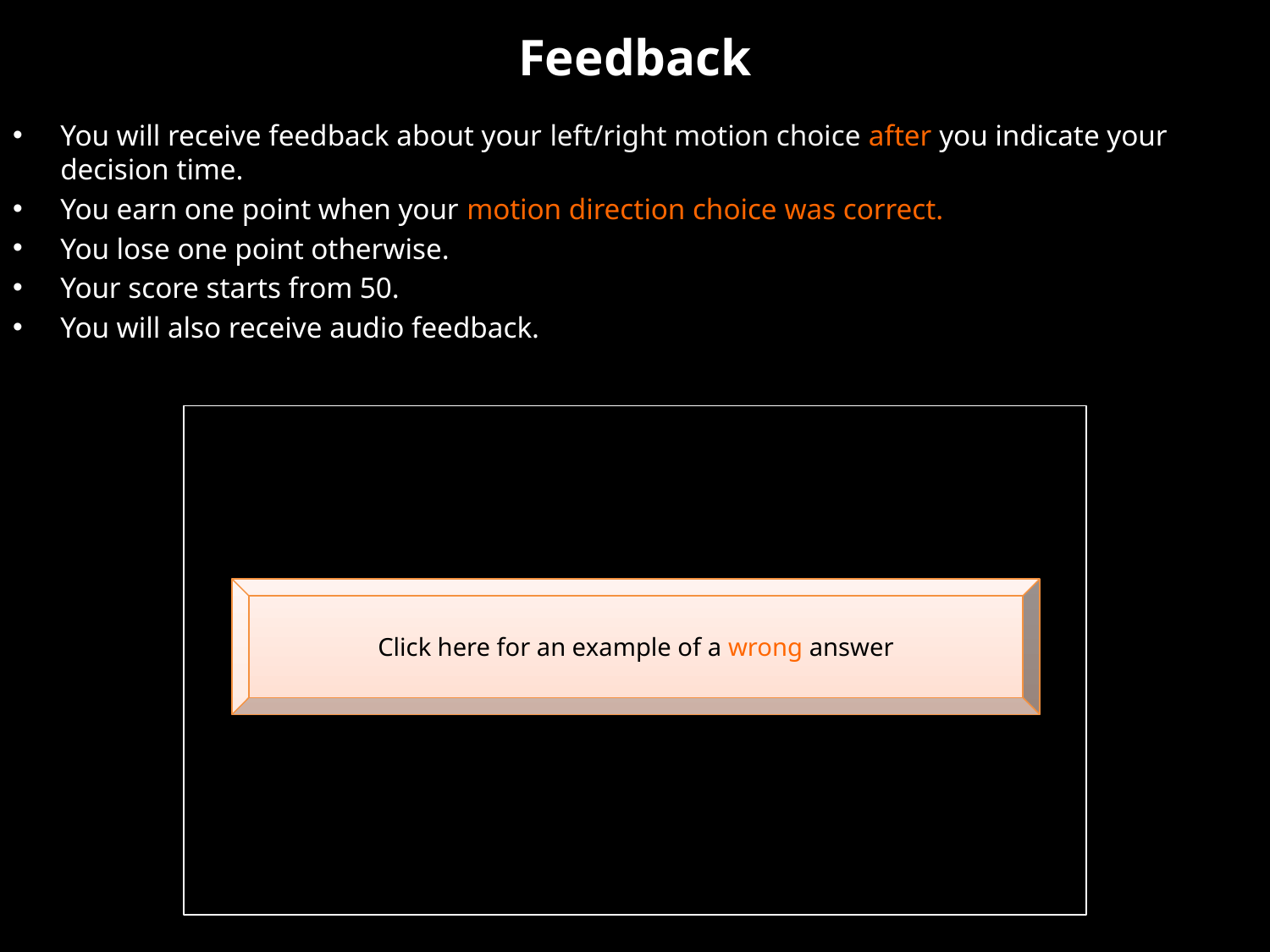

# Feedback
You will receive feedback about your left/right motion choice after you indicate your decision time.
You earn one point when your motion direction choice was correct.
You lose one point otherwise.
Your score starts from 50.
You will also receive audio feedback.
Click here for an example of a wrong answer

## Slide 102
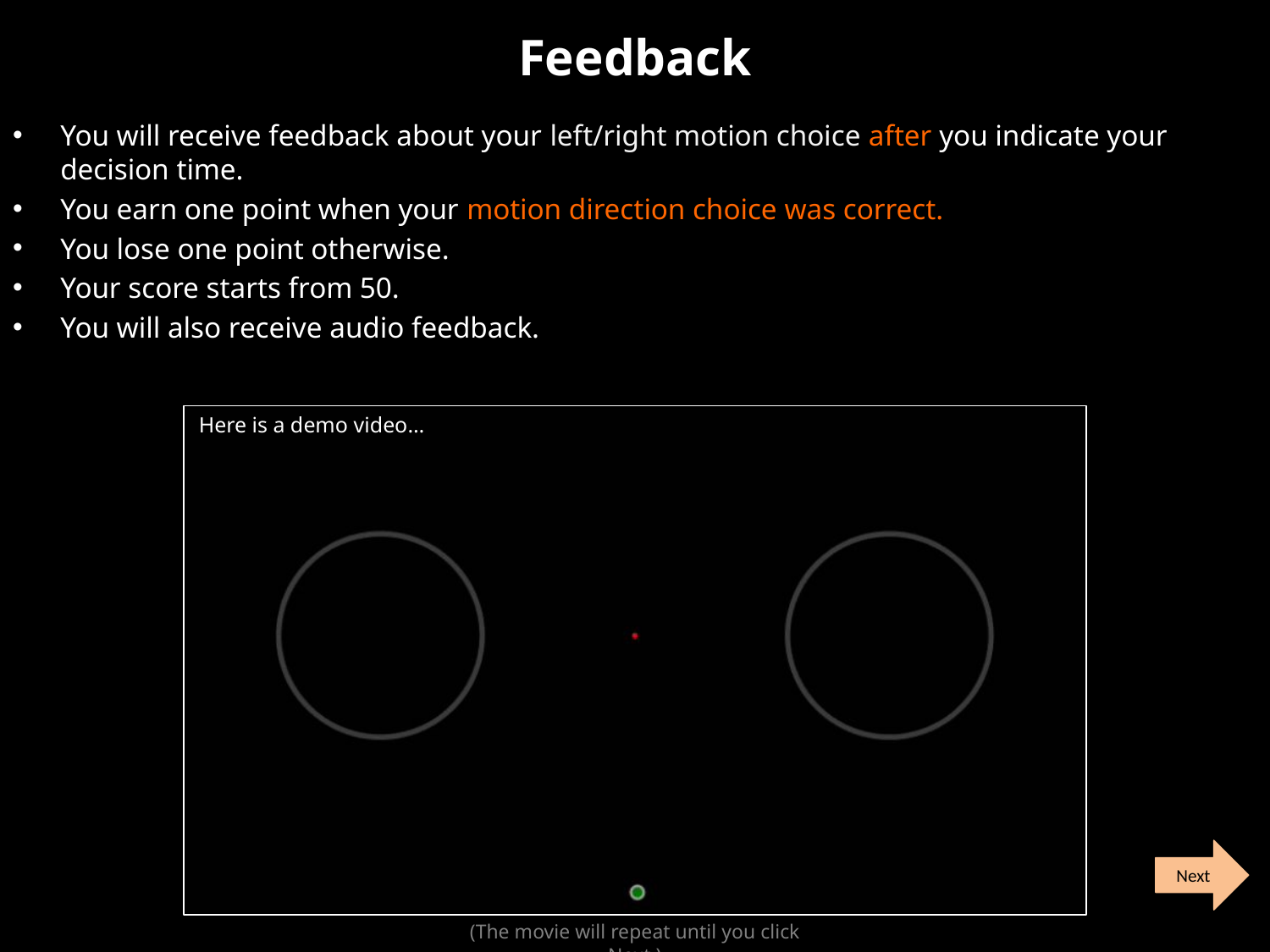

# Feedback
You will receive feedback about your left/right motion choice after you indicate your decision time.
You earn one point when your motion direction choice was correct.
You lose one point otherwise.
Your score starts from 50.
You will also receive audio feedback.
Here is a demo video…

## Slide 103
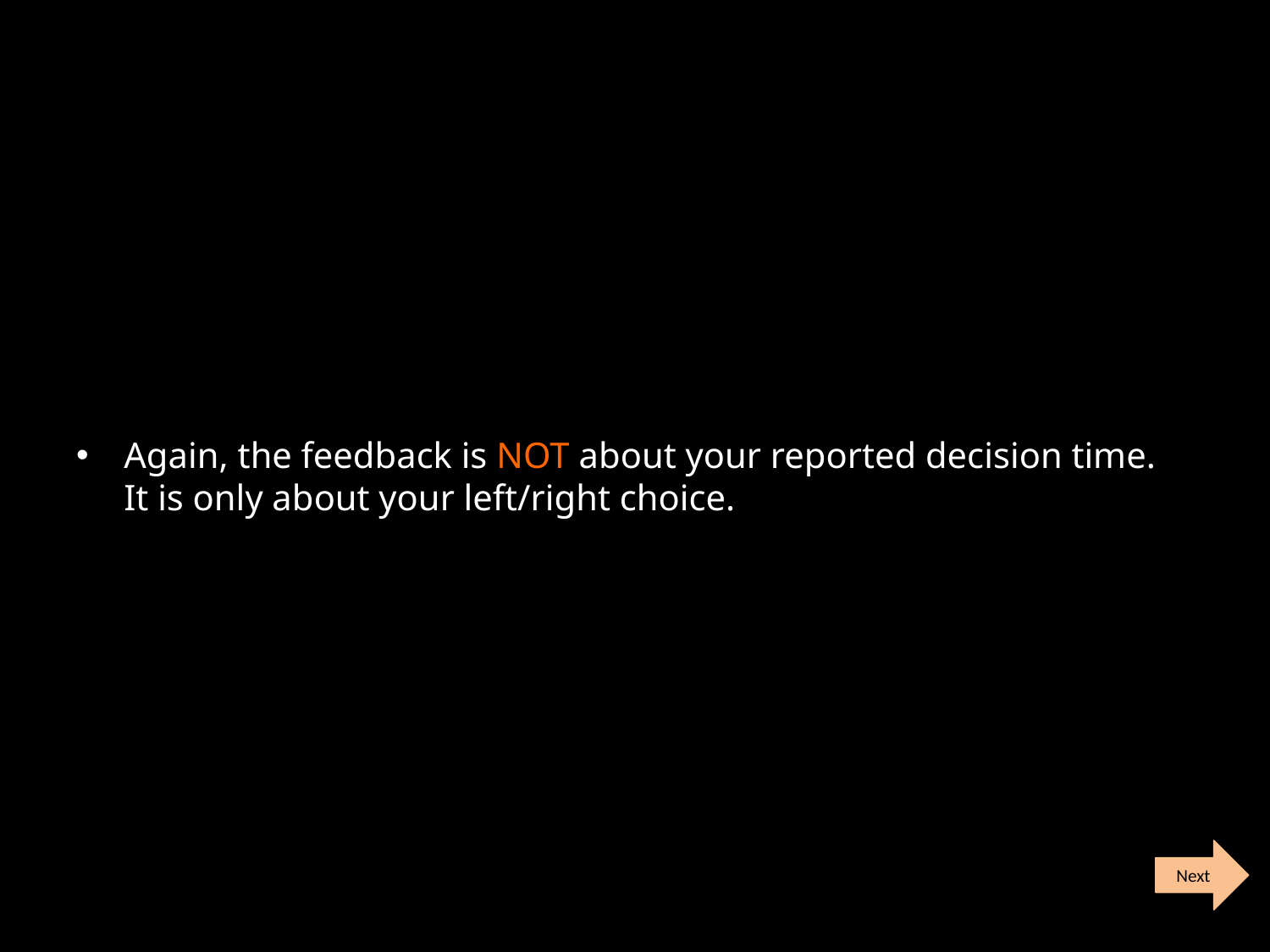

Again, the feedback is NOT about your reported decision time.
It is only about your left/right choice.
Next

## Slide 104
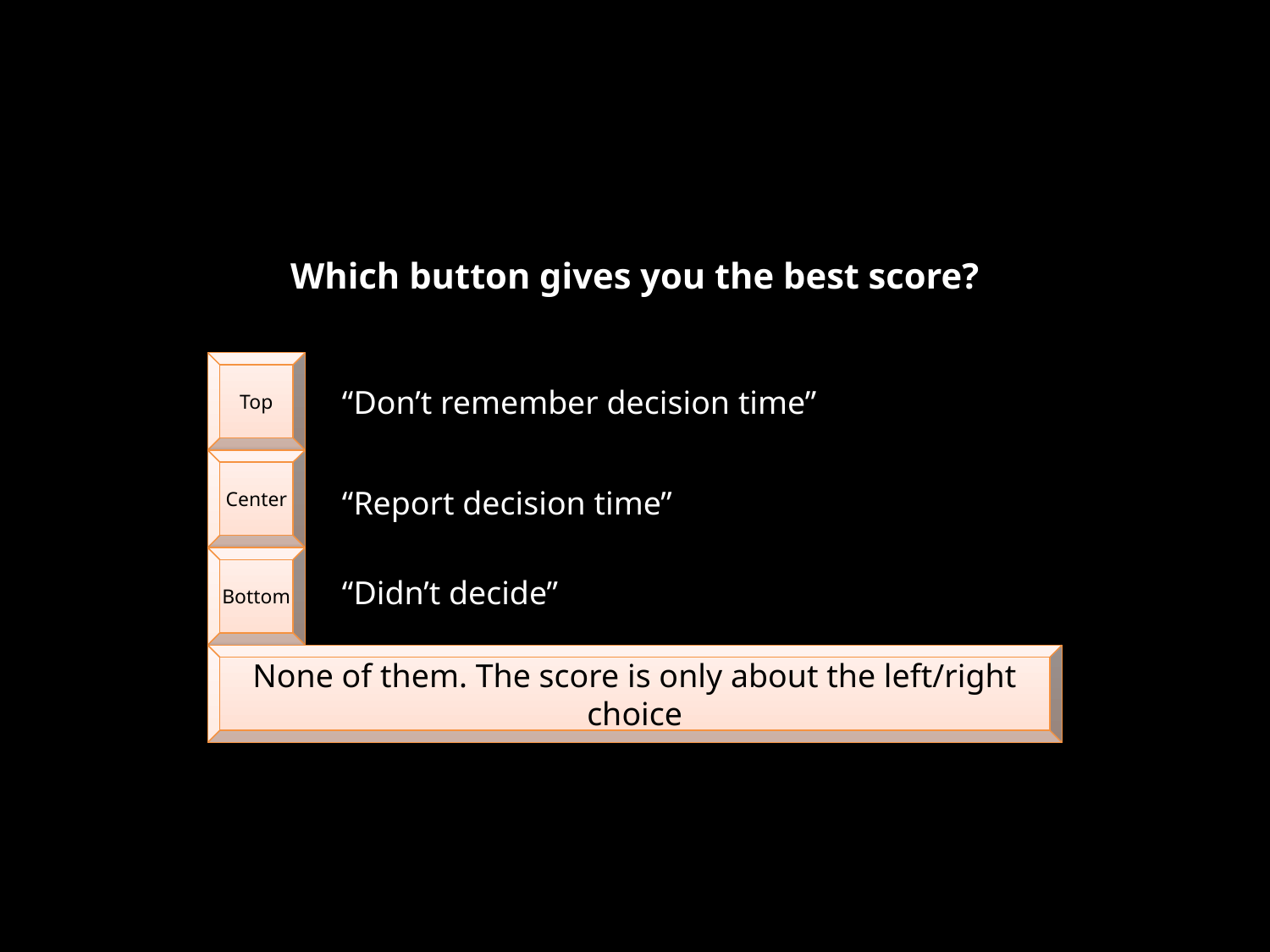

Which button gives you the best score?
Top
“Don’t remember decision time”
Center
“Report decision time”
Bottom
“Didn’t decide”
None of them. The score is only about the left/right choice

## Slide 105
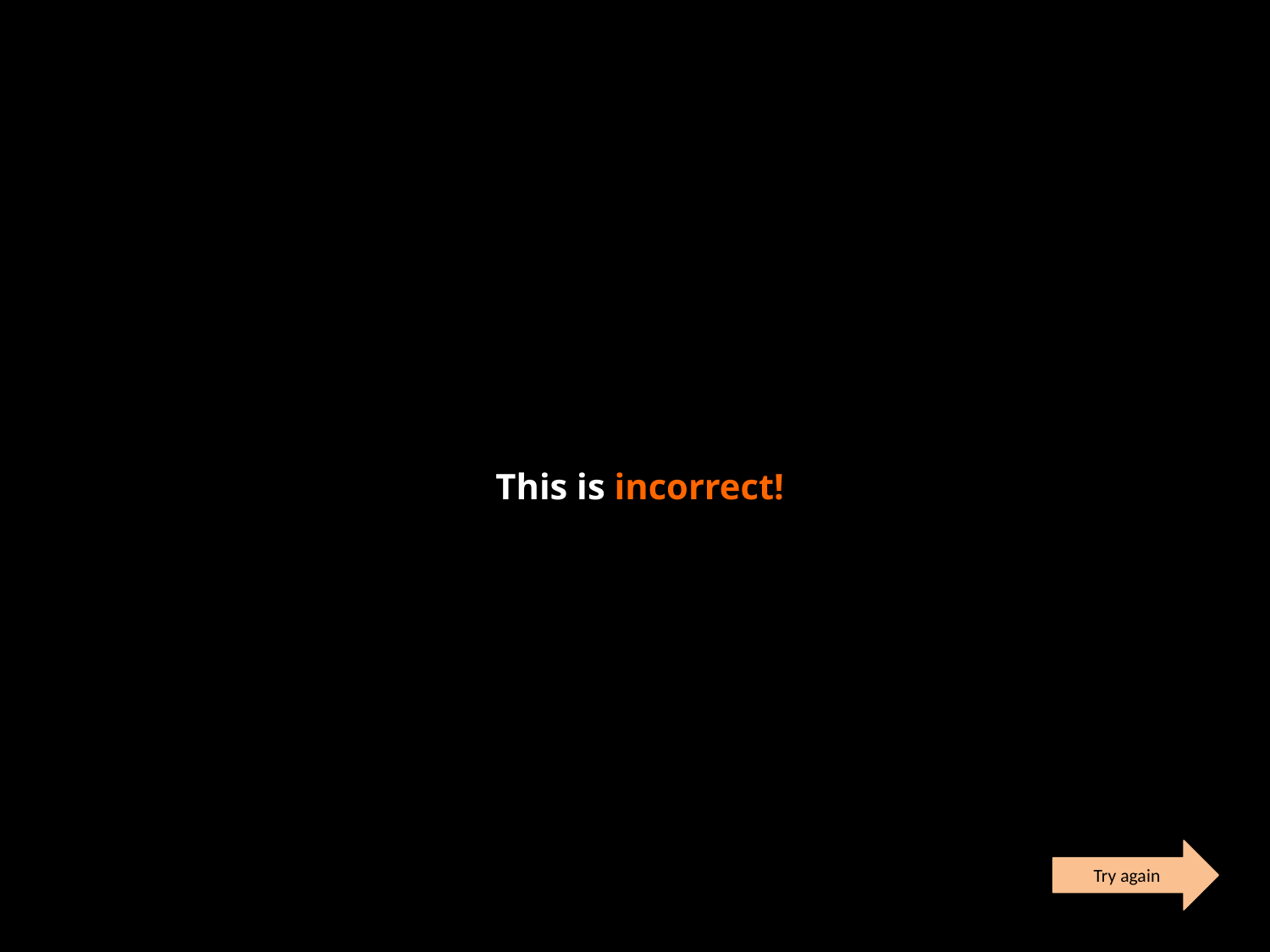

This is incorrect!
Try again

## Slide 106
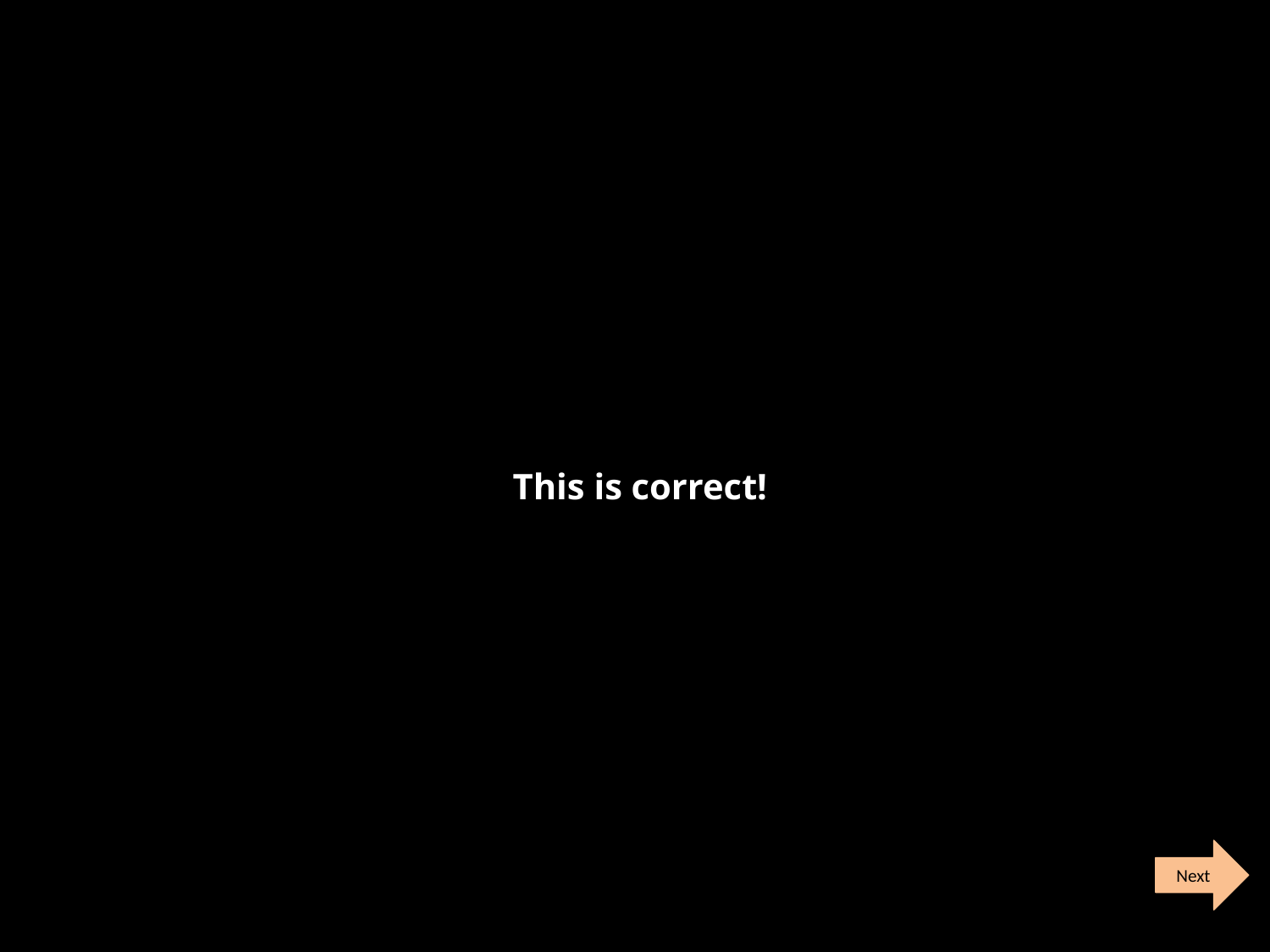

This is correct!
Next

## Slide 107
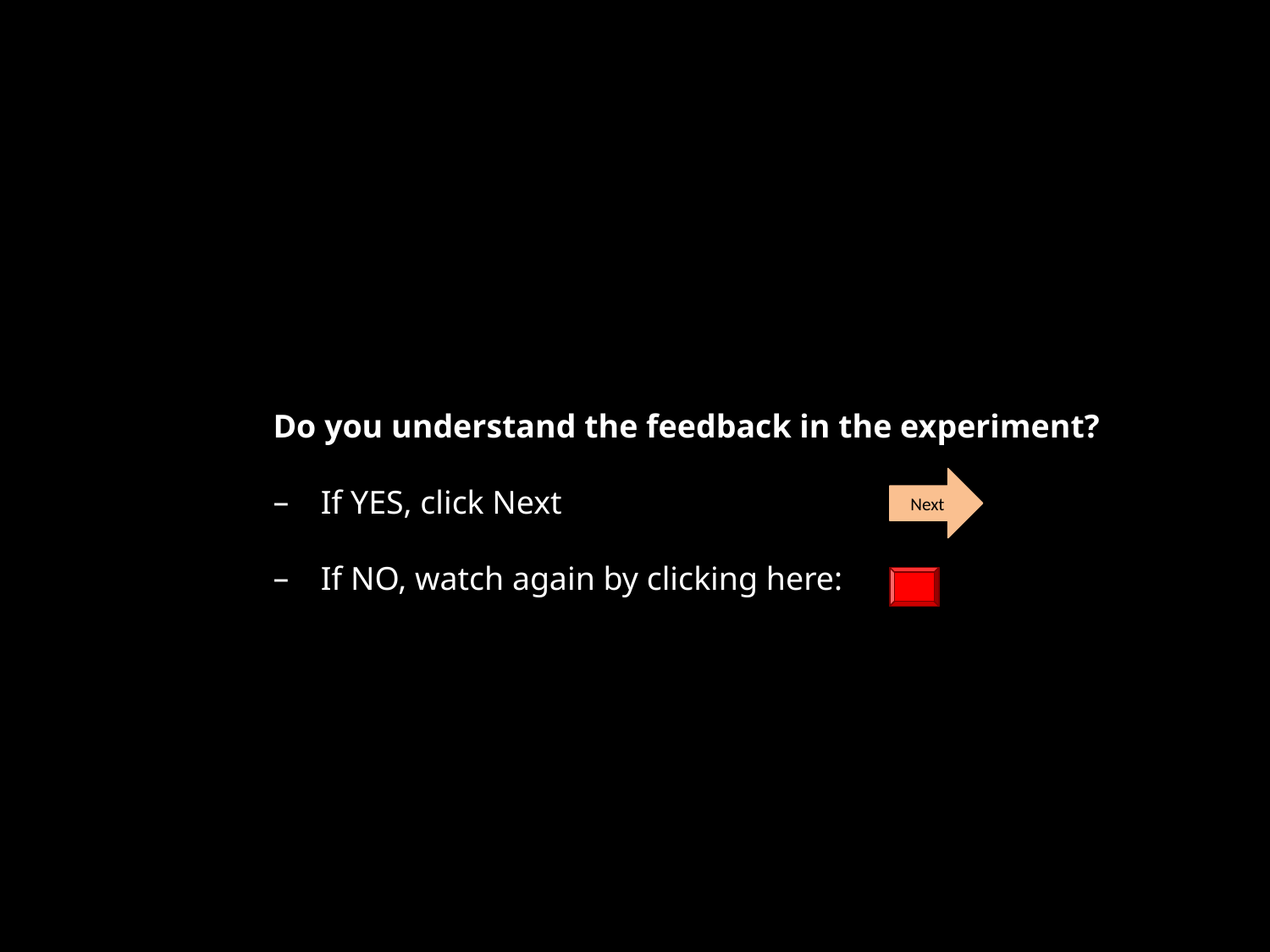

# Do you understand the feedback in the experiment?
Next
If YES, click Next
If NO, watch again by clicking here:

## Slide 108
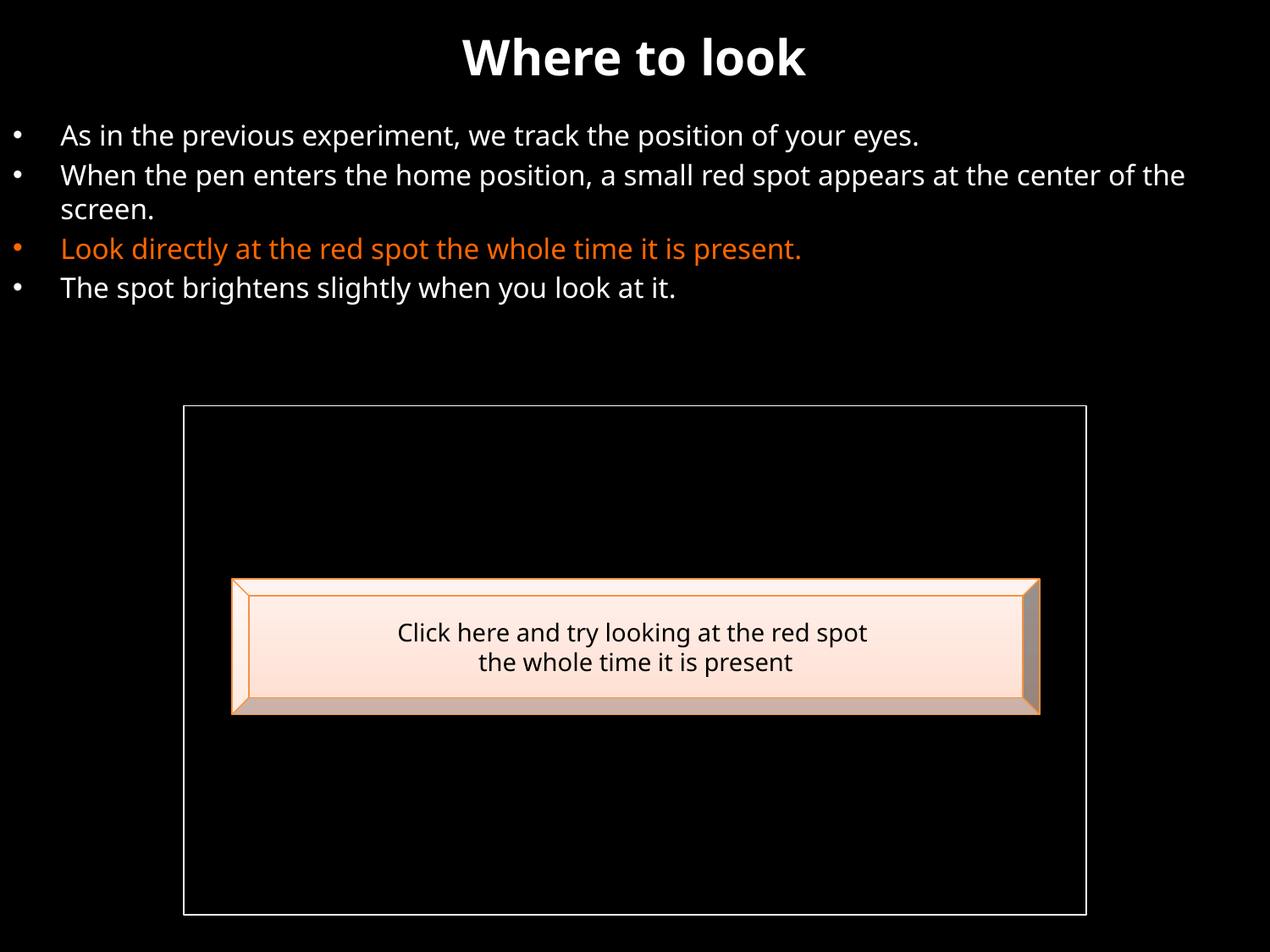

# Where to look
As in the previous experiment, we track the position of your eyes.
When the pen enters the home position, a small red spot appears at the center of the screen.
Look directly at the red spot the whole time it is present.
The spot brightens slightly when you look at it.
Click here and try looking at the red spot
the whole time it is present

## Slide 109
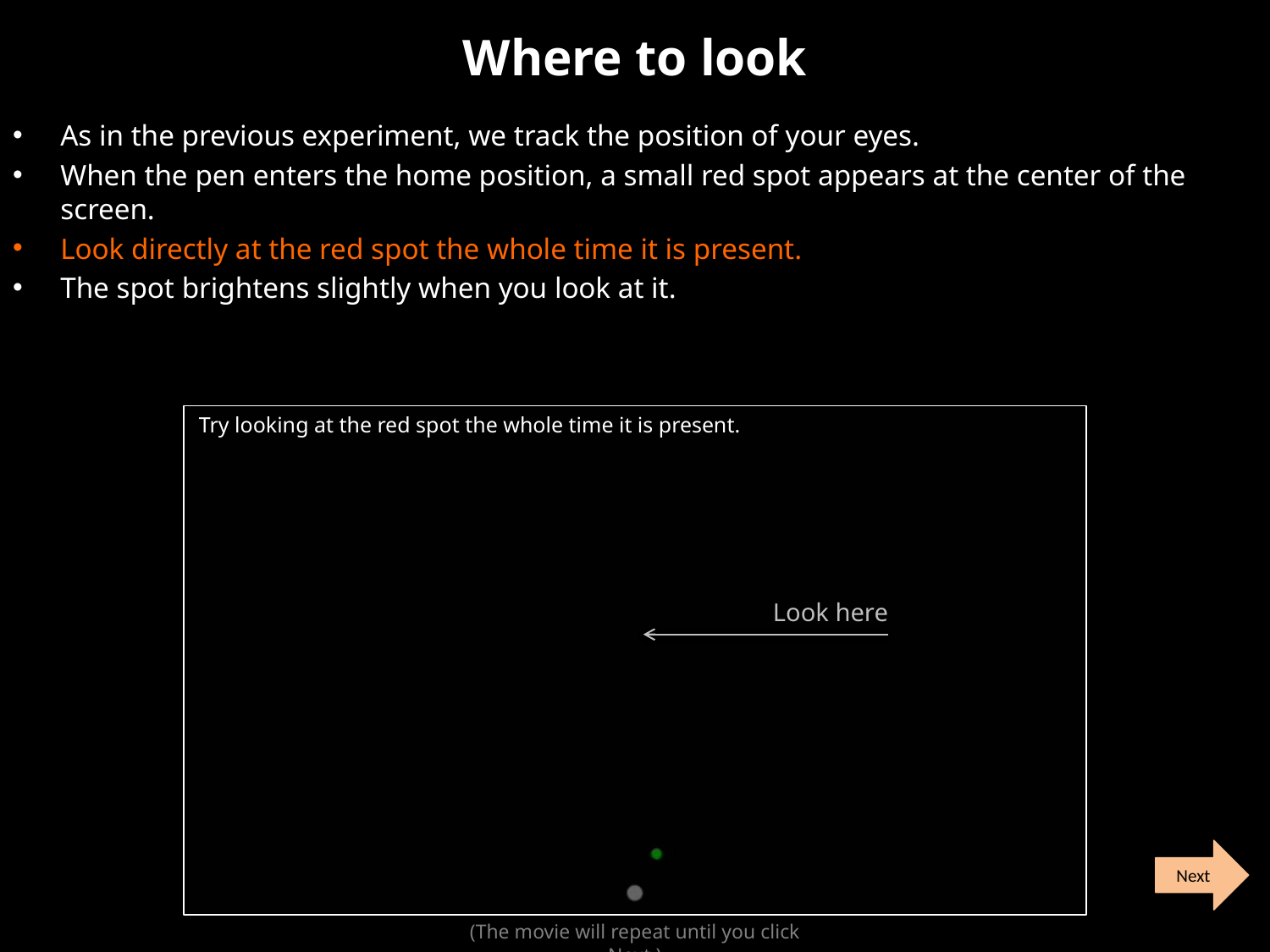

# Where to look
As in the previous experiment, we track the position of your eyes.
When the pen enters the home position, a small red spot appears at the center of the screen.
Look directly at the red spot the whole time it is present.
The spot brightens slightly when you look at it.
Try looking at the red spot the whole time it is present.
Look here

## Slide 110
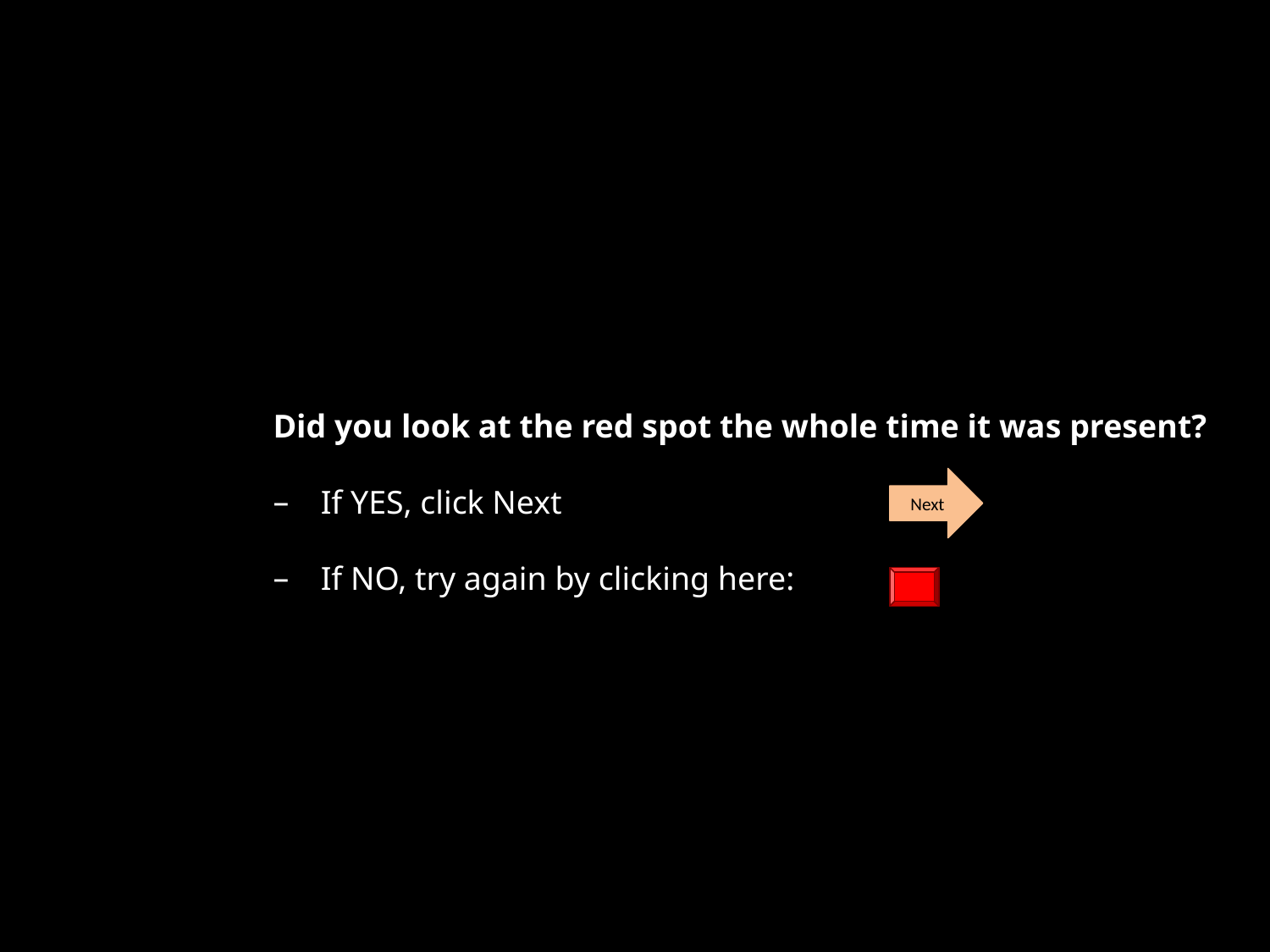

# Did you look at the red spot the whole time it was present?
Next
If YES, click Next
If NO, try again by clicking here:

## Slide 111
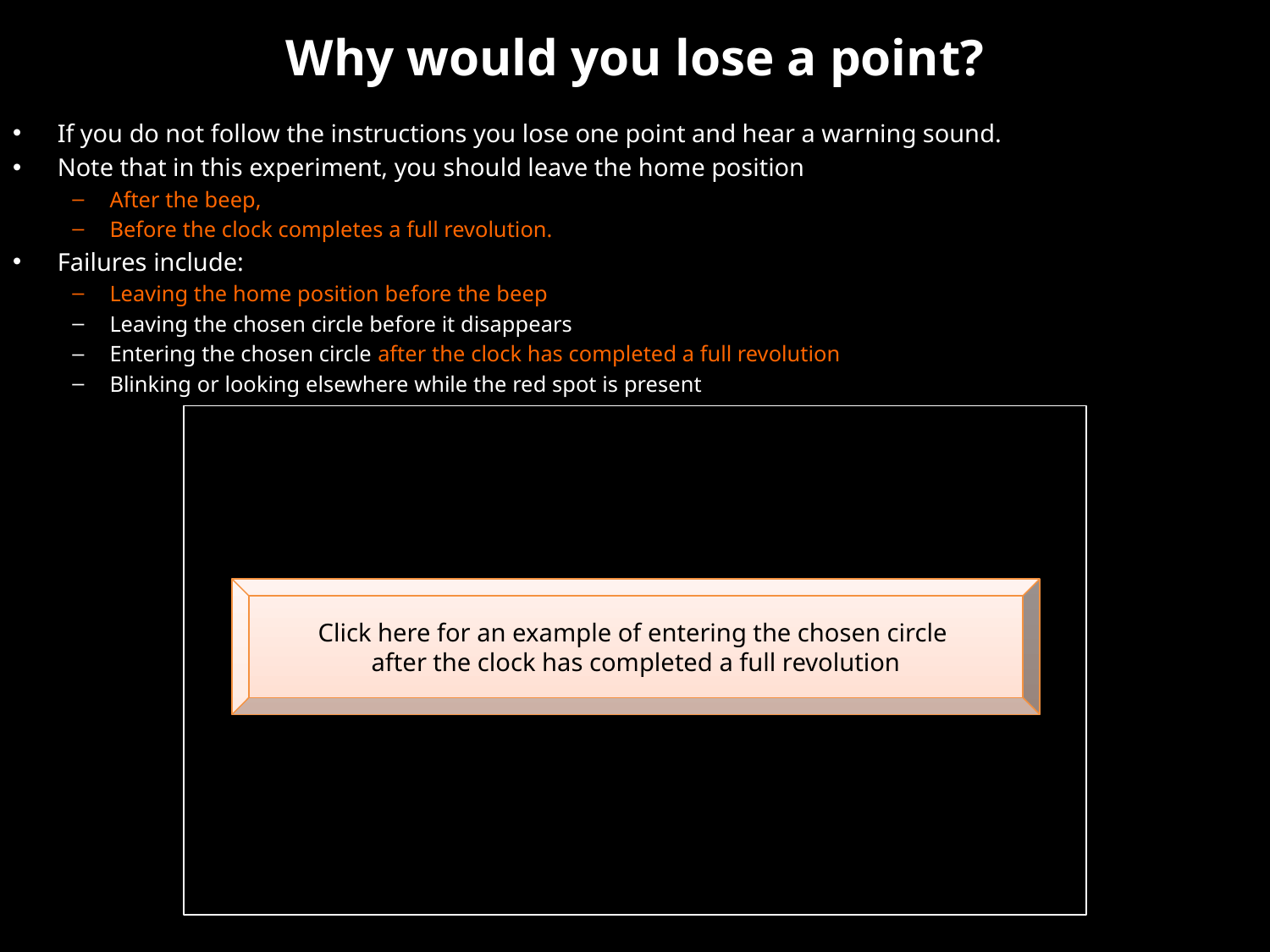

# Why would you lose a point?
If you do not follow the instructions you lose one point and hear a warning sound.
Note that in this experiment, you should leave the home position
After the beep,
Before the clock completes a full revolution.
Failures include:
Leaving the home position before the beep
Leaving the chosen circle before it disappears
Entering the chosen circle after the clock has completed a full revolution
Blinking or looking elsewhere while the red spot is present
Click here for an example of entering the chosen circle
after the clock has completed a full revolution

## Slide 112
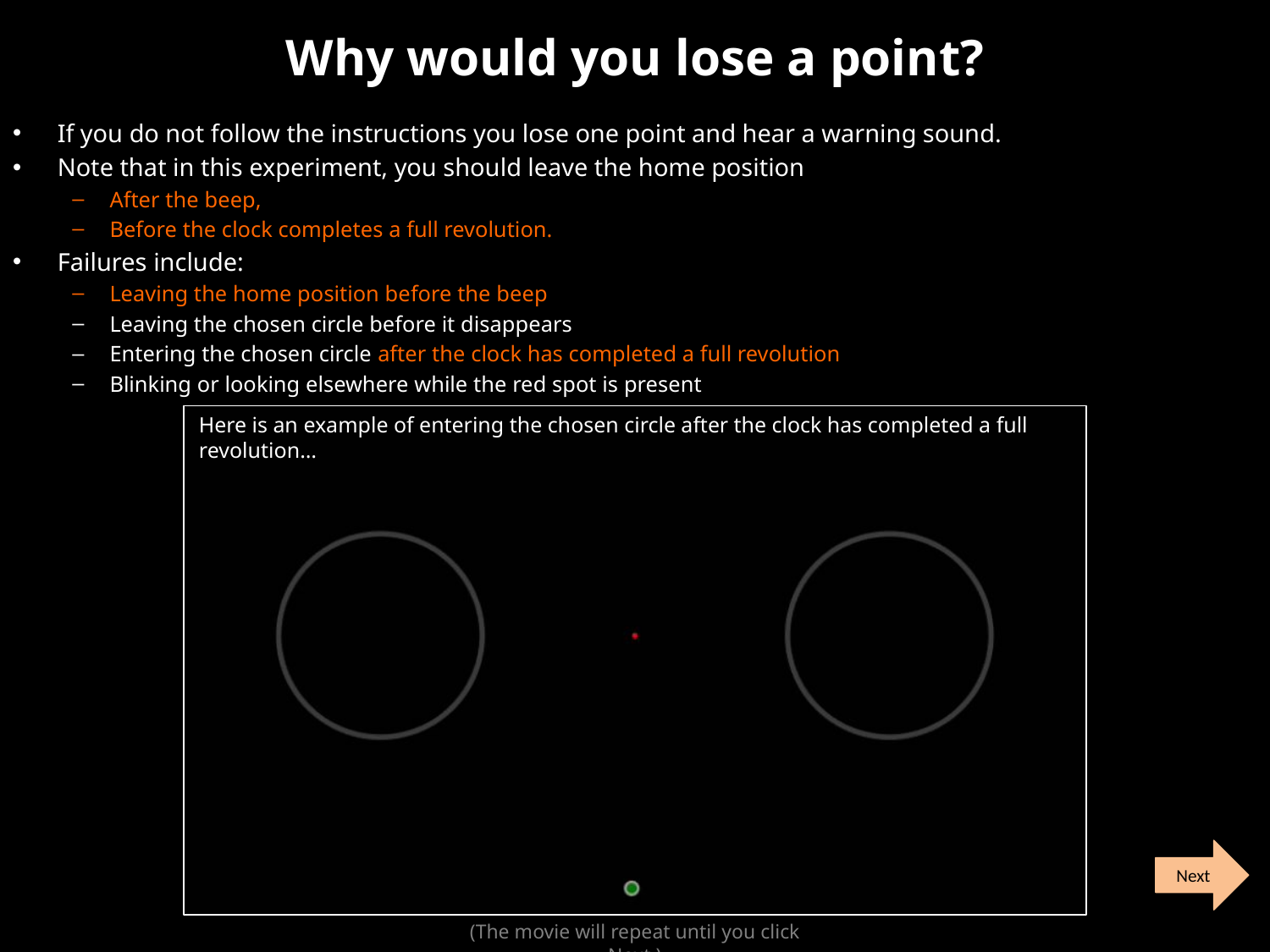

# Why would you lose a point?
If you do not follow the instructions you lose one point and hear a warning sound.
Note that in this experiment, you should leave the home position
After the beep,
Before the clock completes a full revolution.
Failures include:
Leaving the home position before the beep
Leaving the chosen circle before it disappears
Entering the chosen circle after the clock has completed a full revolution
Blinking or looking elsewhere while the red spot is present
Here is an example of entering the chosen circle after the clock has completed a full revolution…

## Slide 113
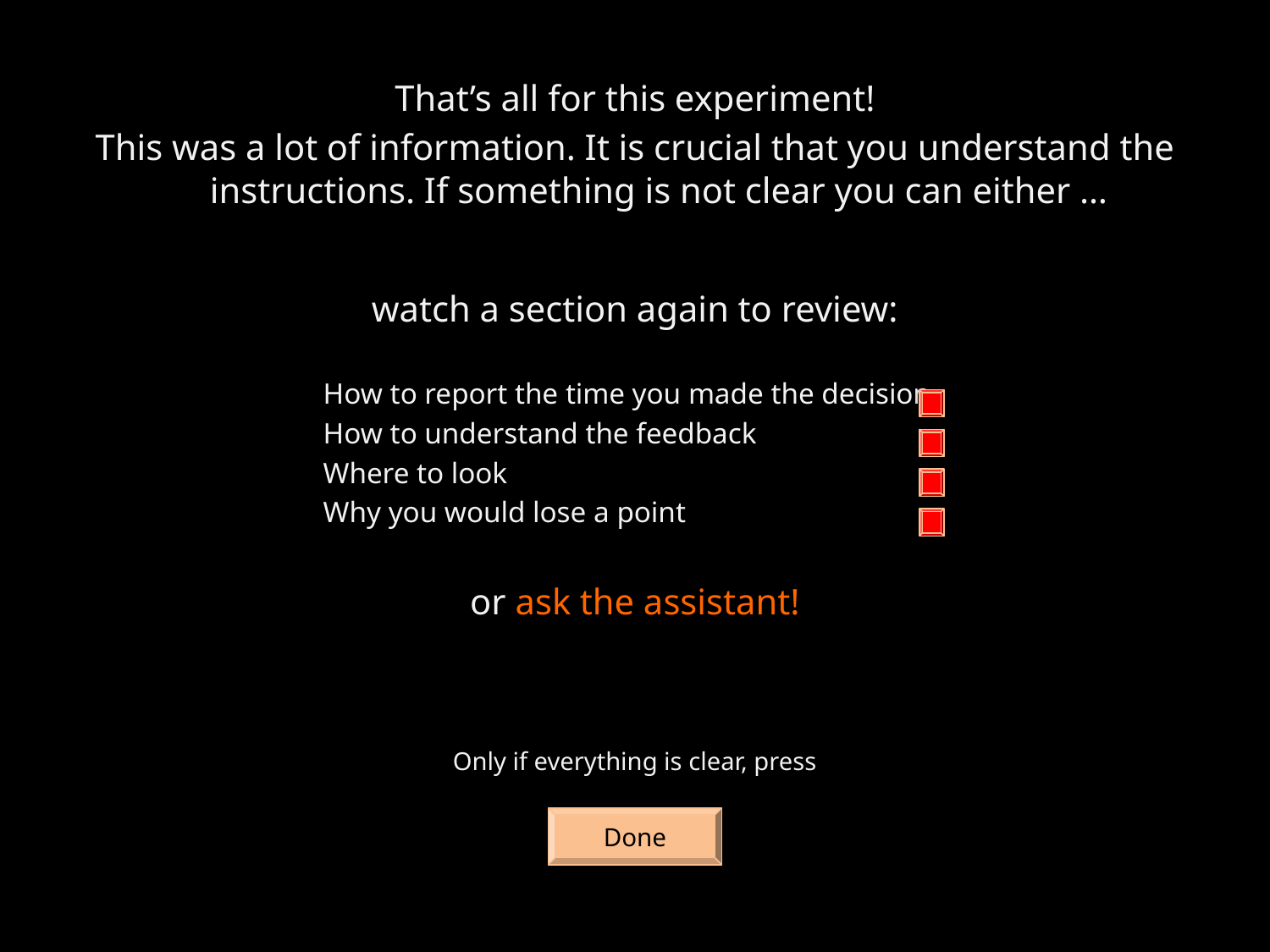

That’s all for this experiment!
This was a lot of information. It is crucial that you understand the instructions. If something is not clear you can either …
watch a section again to review:
How to report the time you made the decision
How to understand the feedback
Where to look
Why you would lose a point
or ask the assistant!
Only if everything is clear, press
Done

## Slide 114
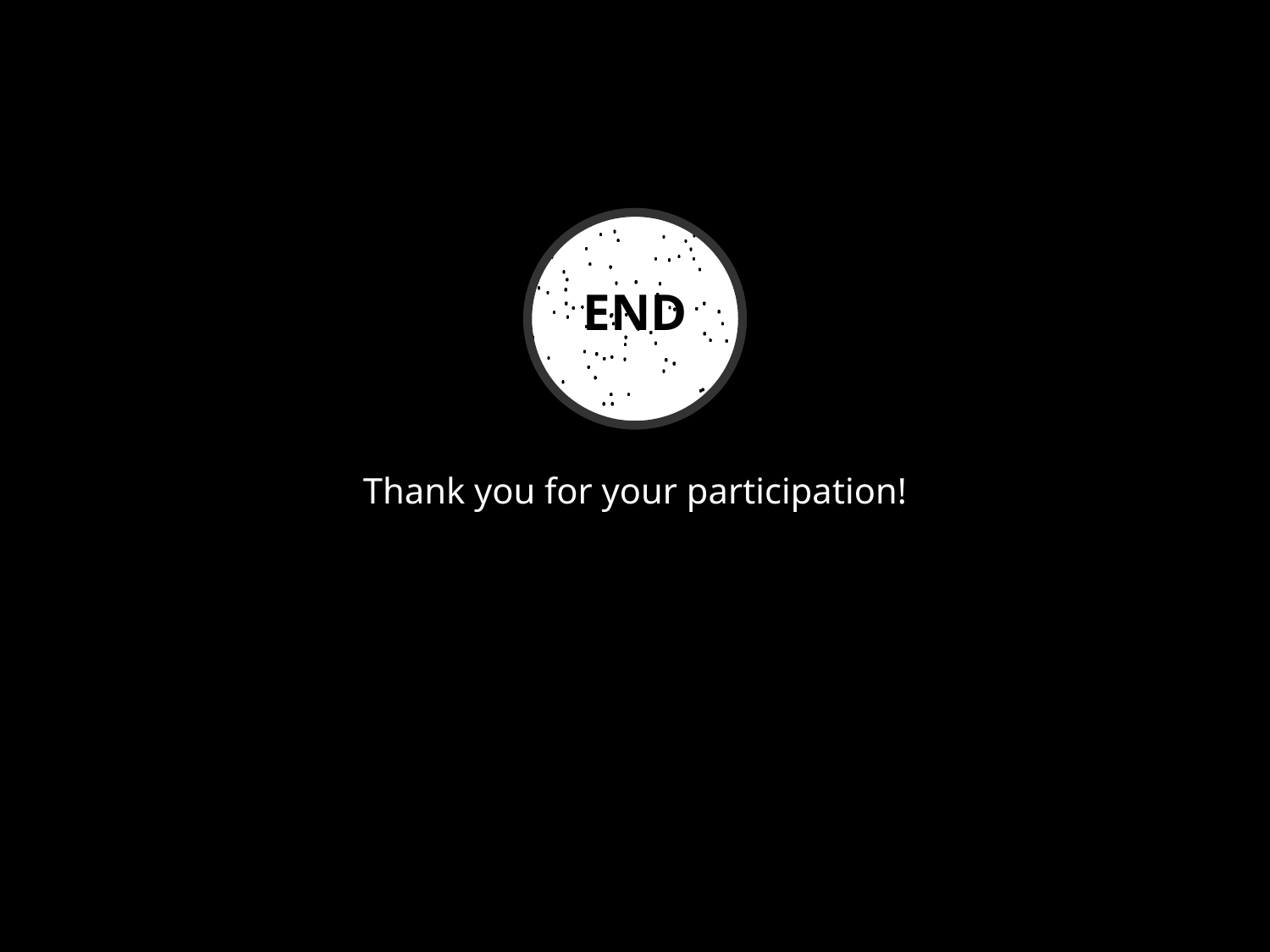

# END
Thank you for your participation!

## Slide 115
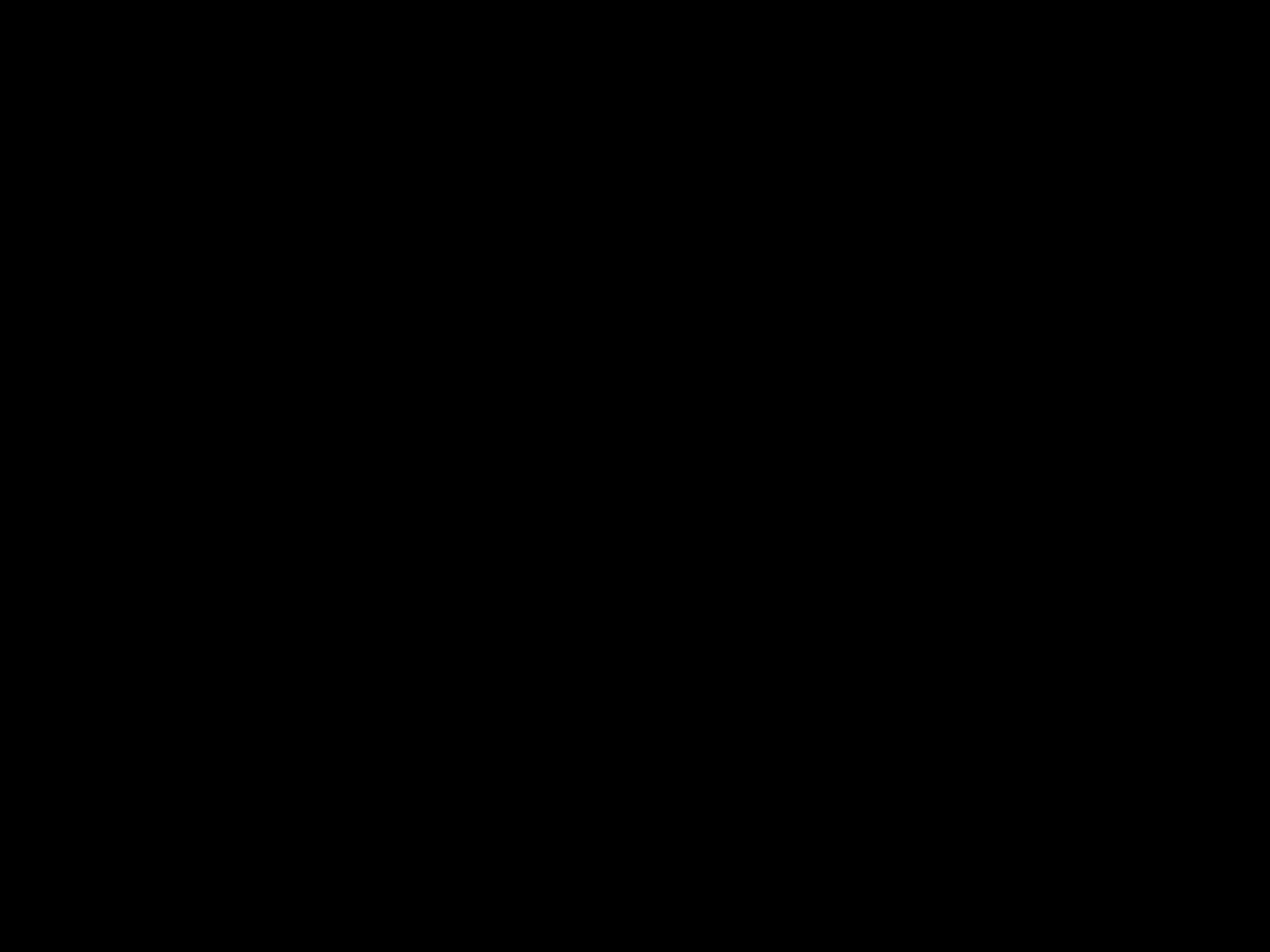

#

## Slide 116
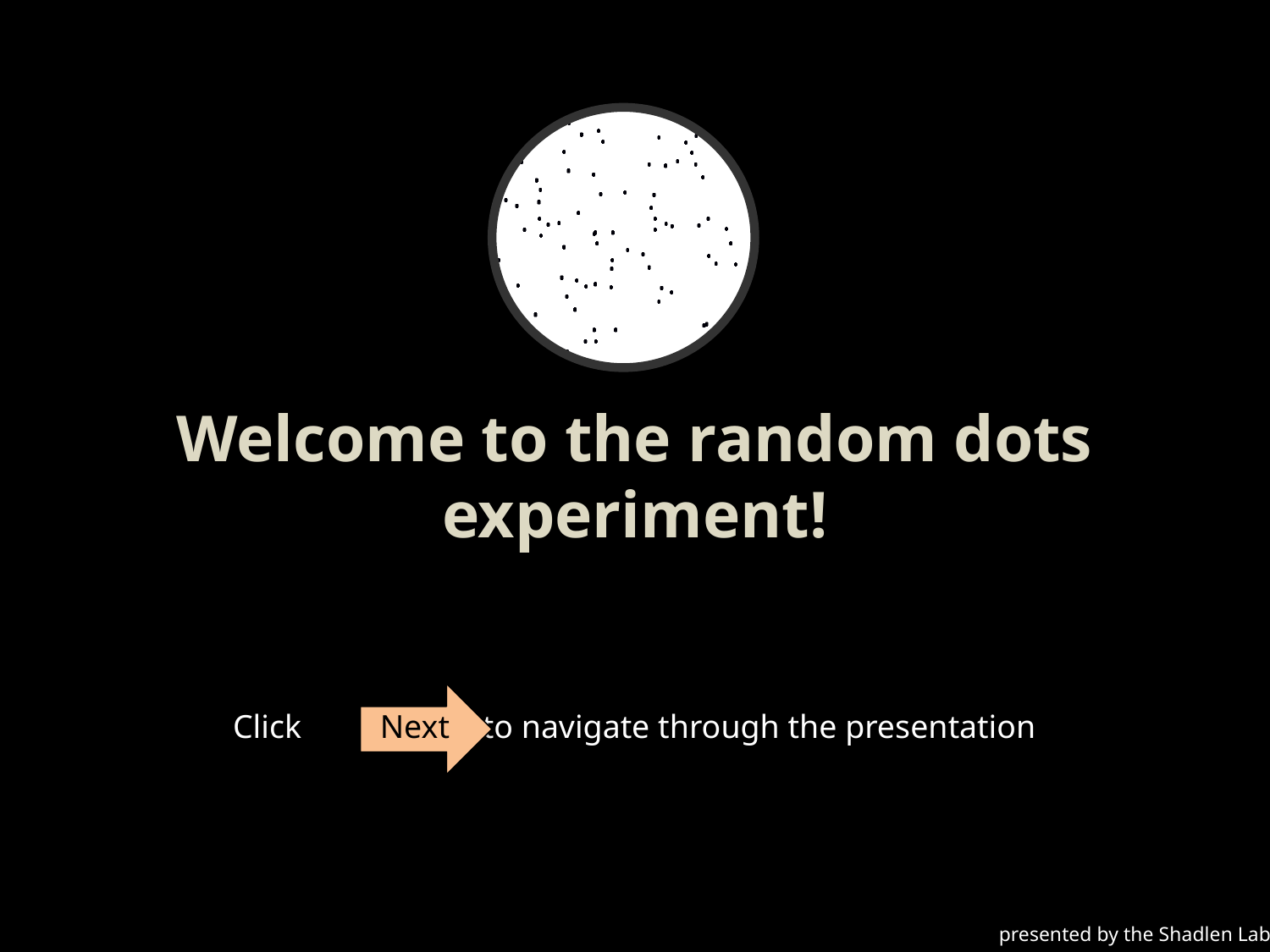

# Welcome to the random dots experiment!
Next
Click to navigate through the presentation
presented by the Shadlen Lab

## Slide 117
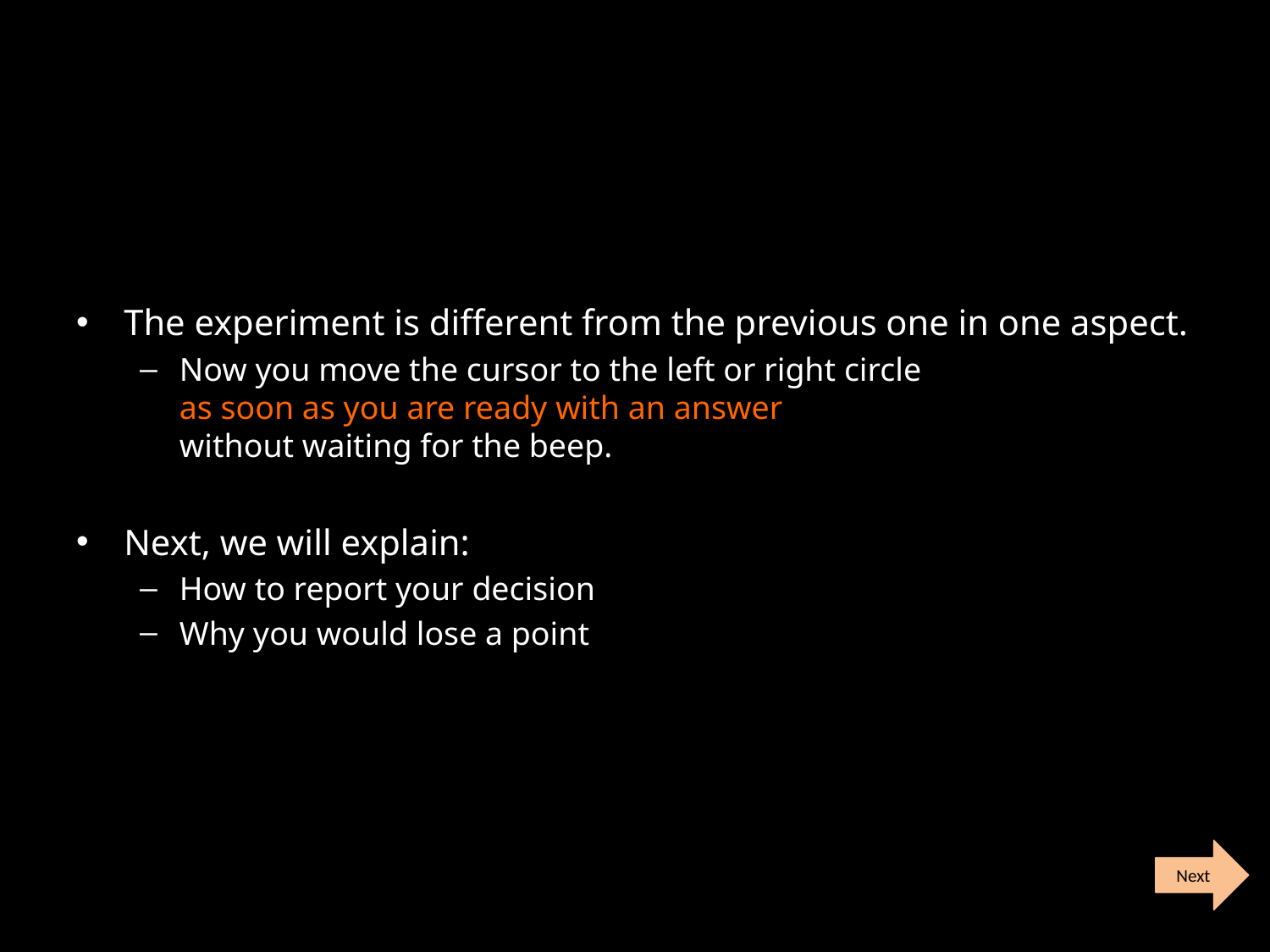

The experiment is different from the previous one in one aspect.
Now you move the cursor to the left or right circle
as soon as you are ready with an answer
without waiting for the beep.
Next, we will explain:
How to report your decision
Why you would lose a point
Next

## Slide 118
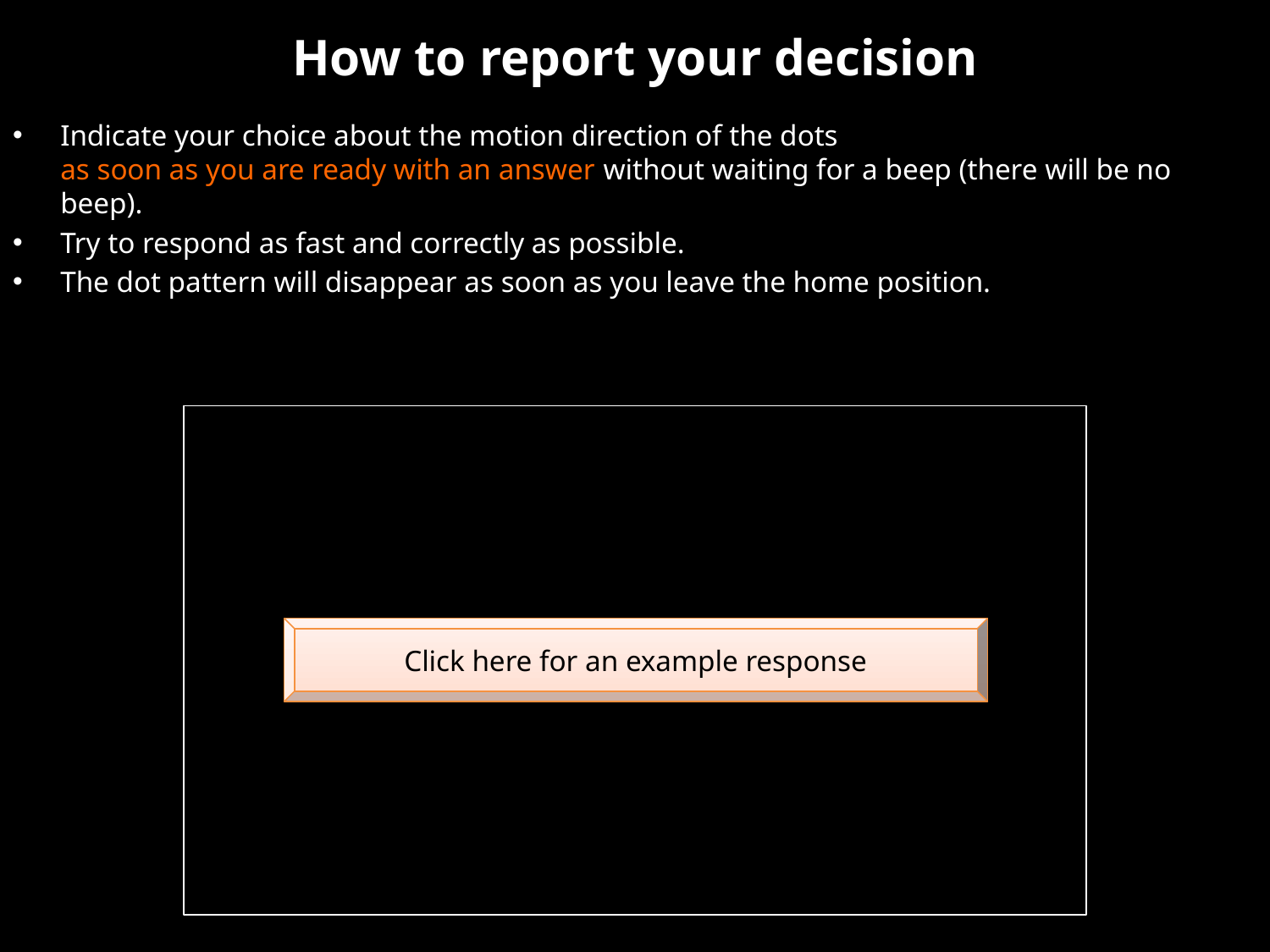

# How to report your decision
Indicate your choice about the motion direction of the dots
as soon as you are ready with an answer without waiting for a beep (there will be no beep).
Try to respond as fast and correctly as possible.
The dot pattern will disappear as soon as you leave the home position.
Click here for an example response

## Slide 119
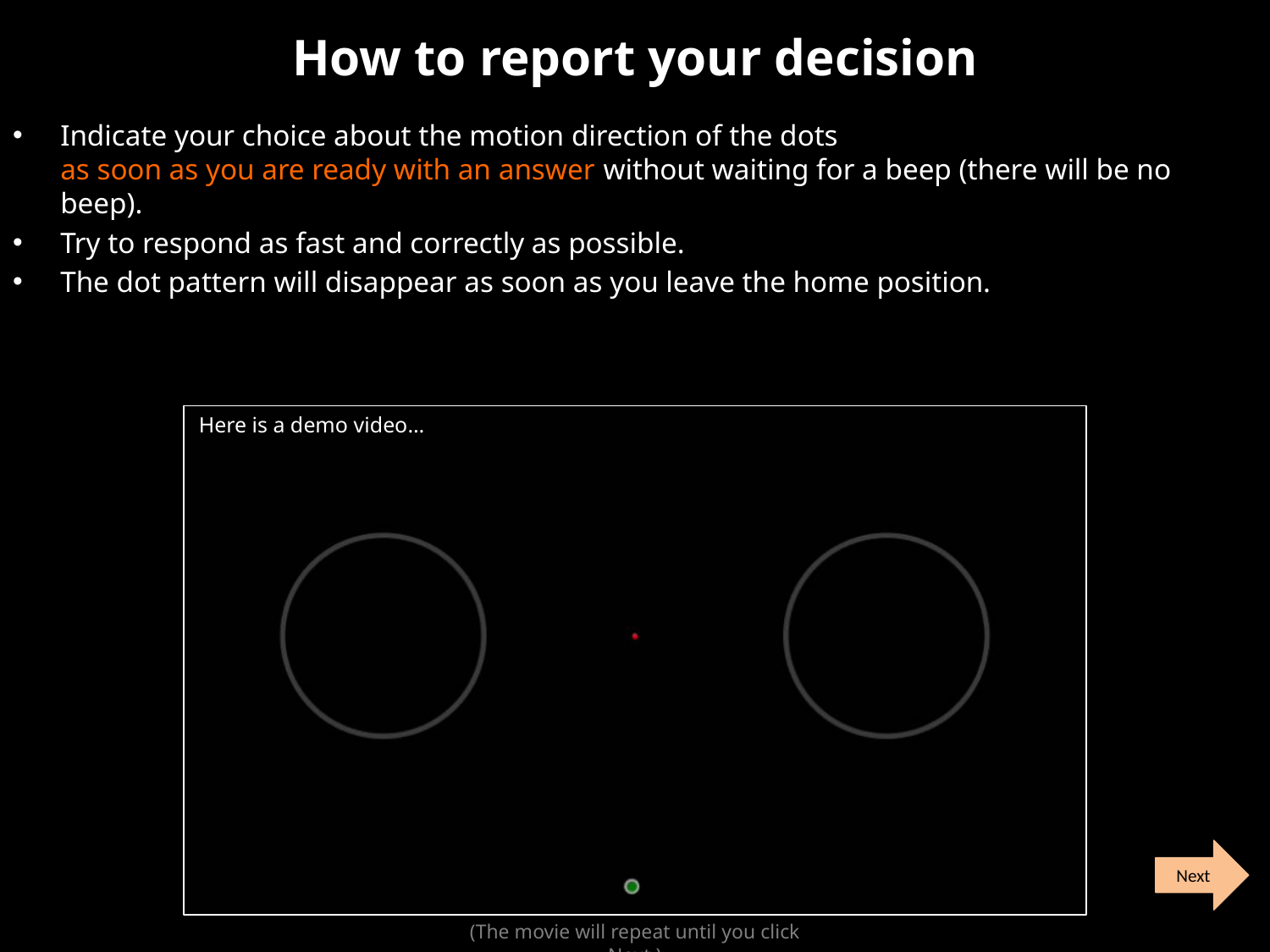

# How to report your decision
Indicate your choice about the motion direction of the dots
as soon as you are ready with an answer without waiting for a beep (there will be no beep).
Try to respond as fast and correctly as possible.
The dot pattern will disappear as soon as you leave the home position.
Here is a demo video…

## Slide 120
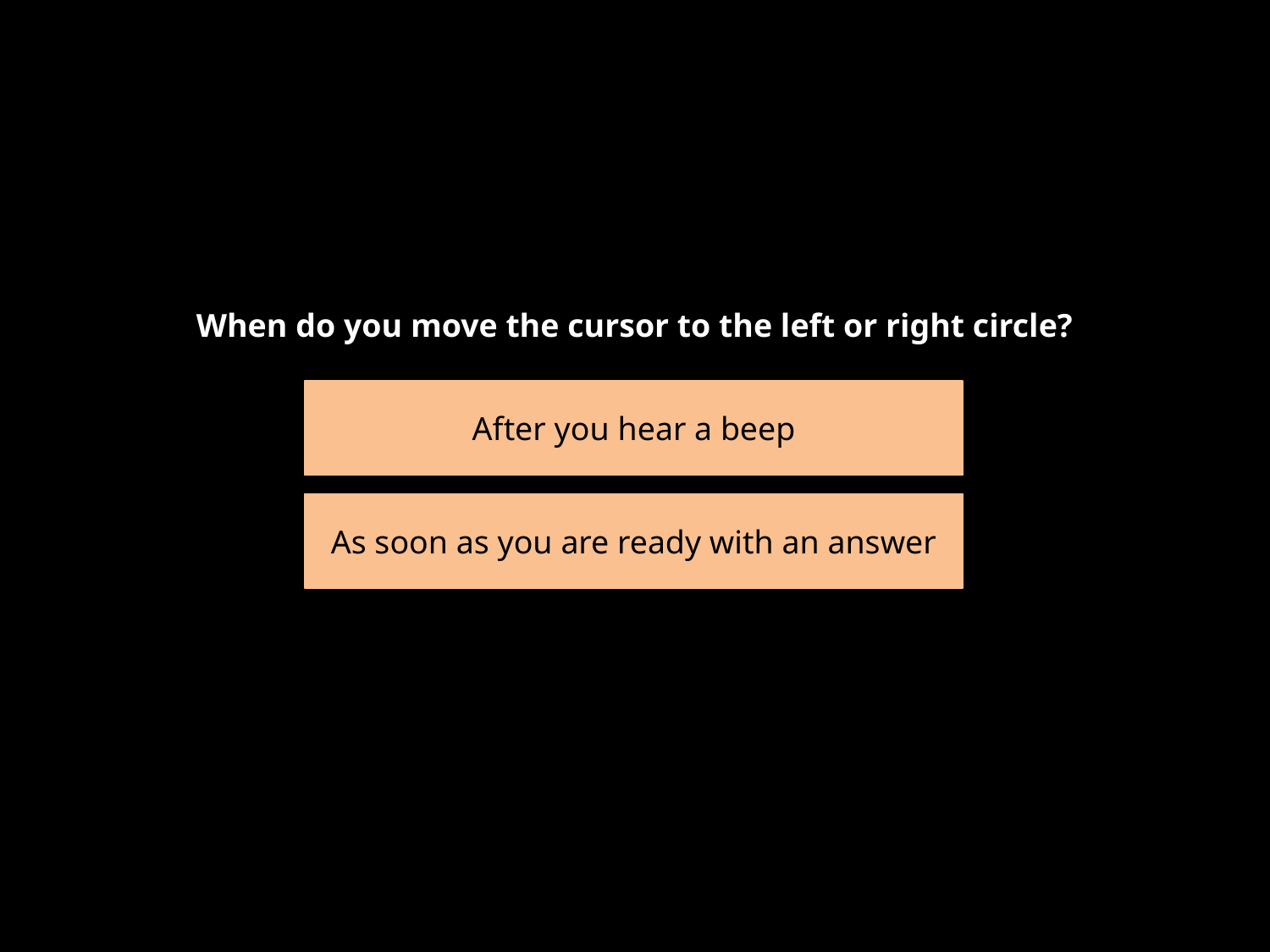

When do you move the cursor to the left or right circle?
After you hear a beep
As soon as you are ready with an answer

## Slide 121
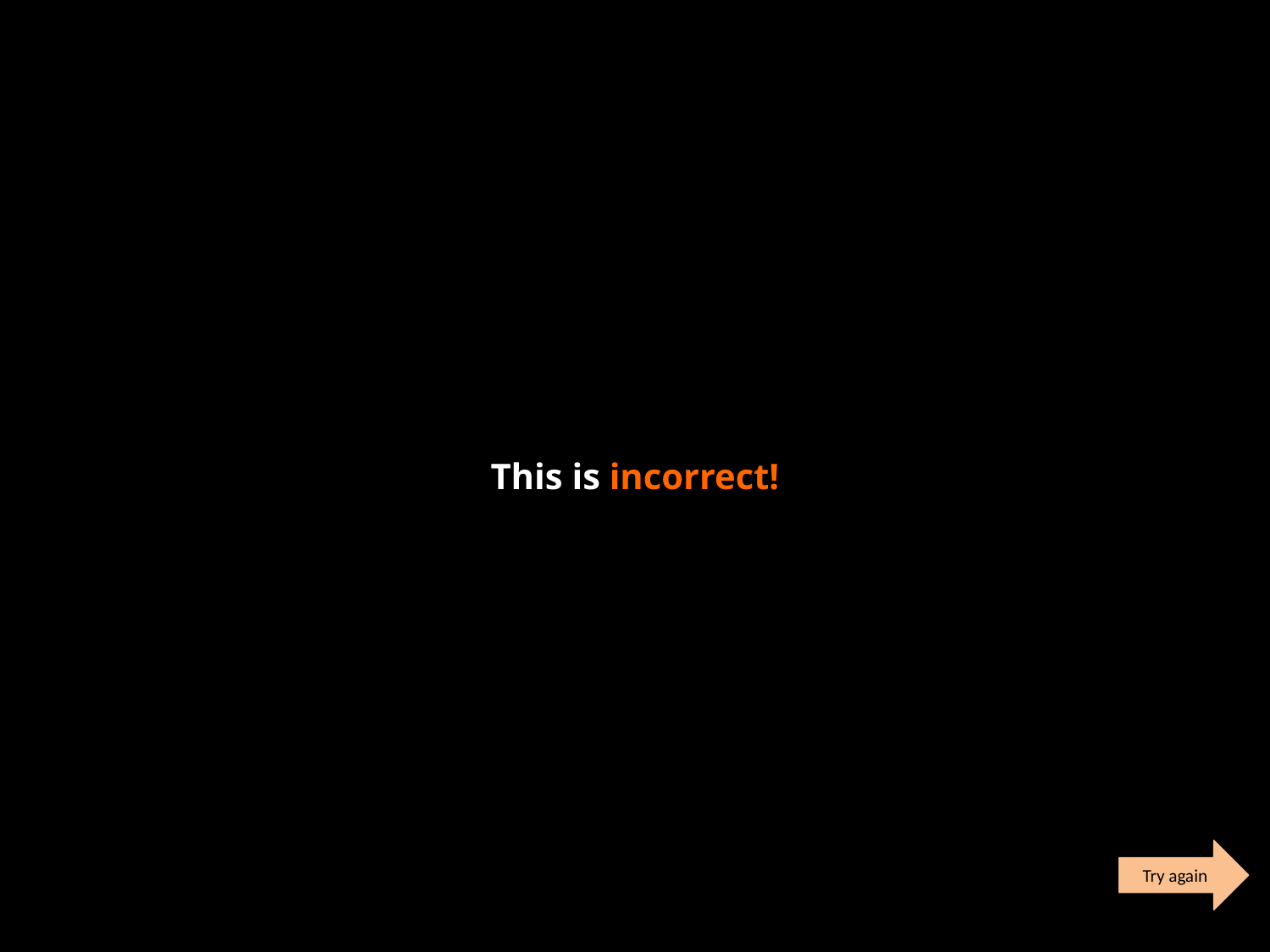

This is incorrect!
Try again

## Slide 122
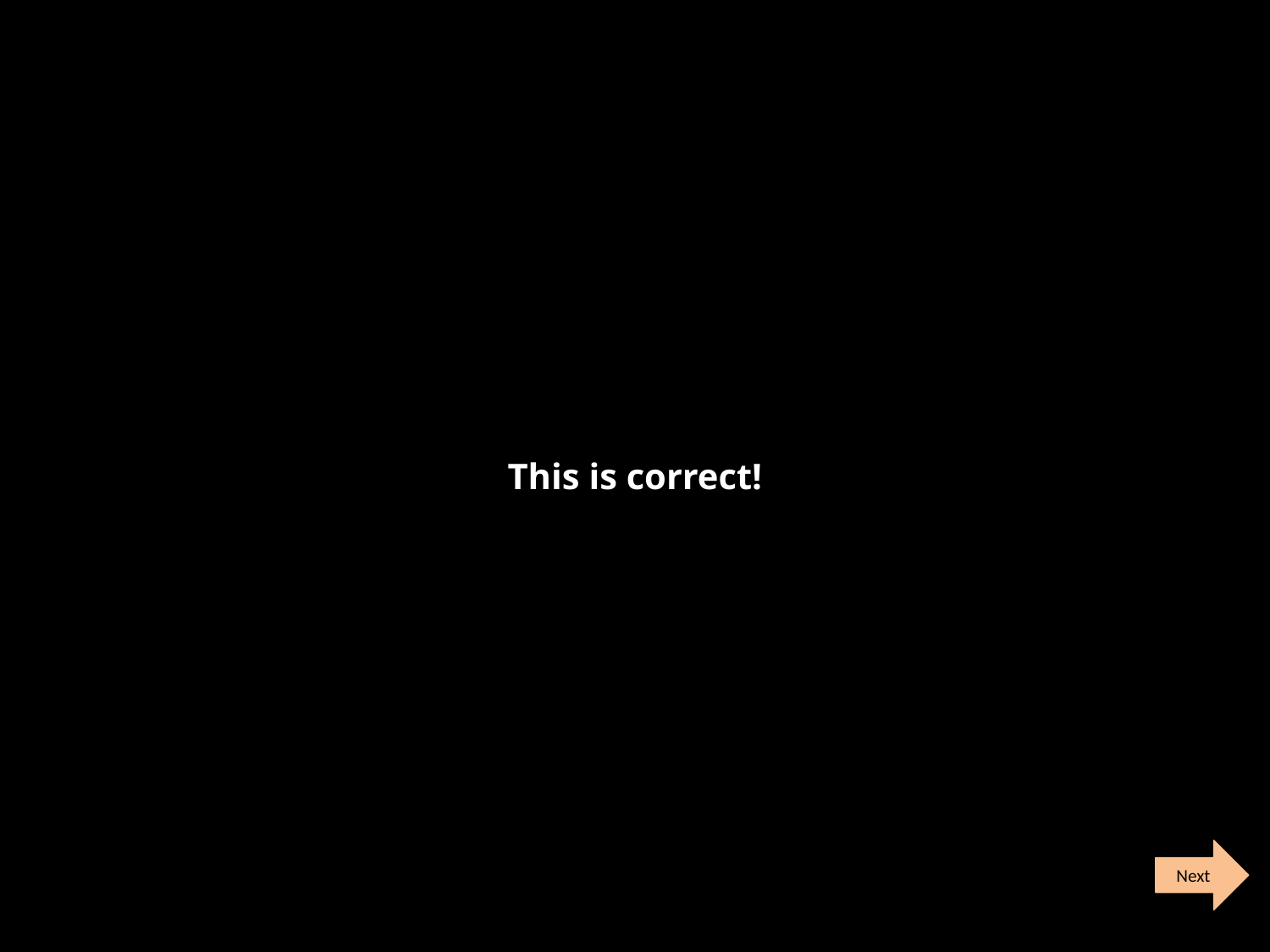

This is correct!
Next

## Slide 123
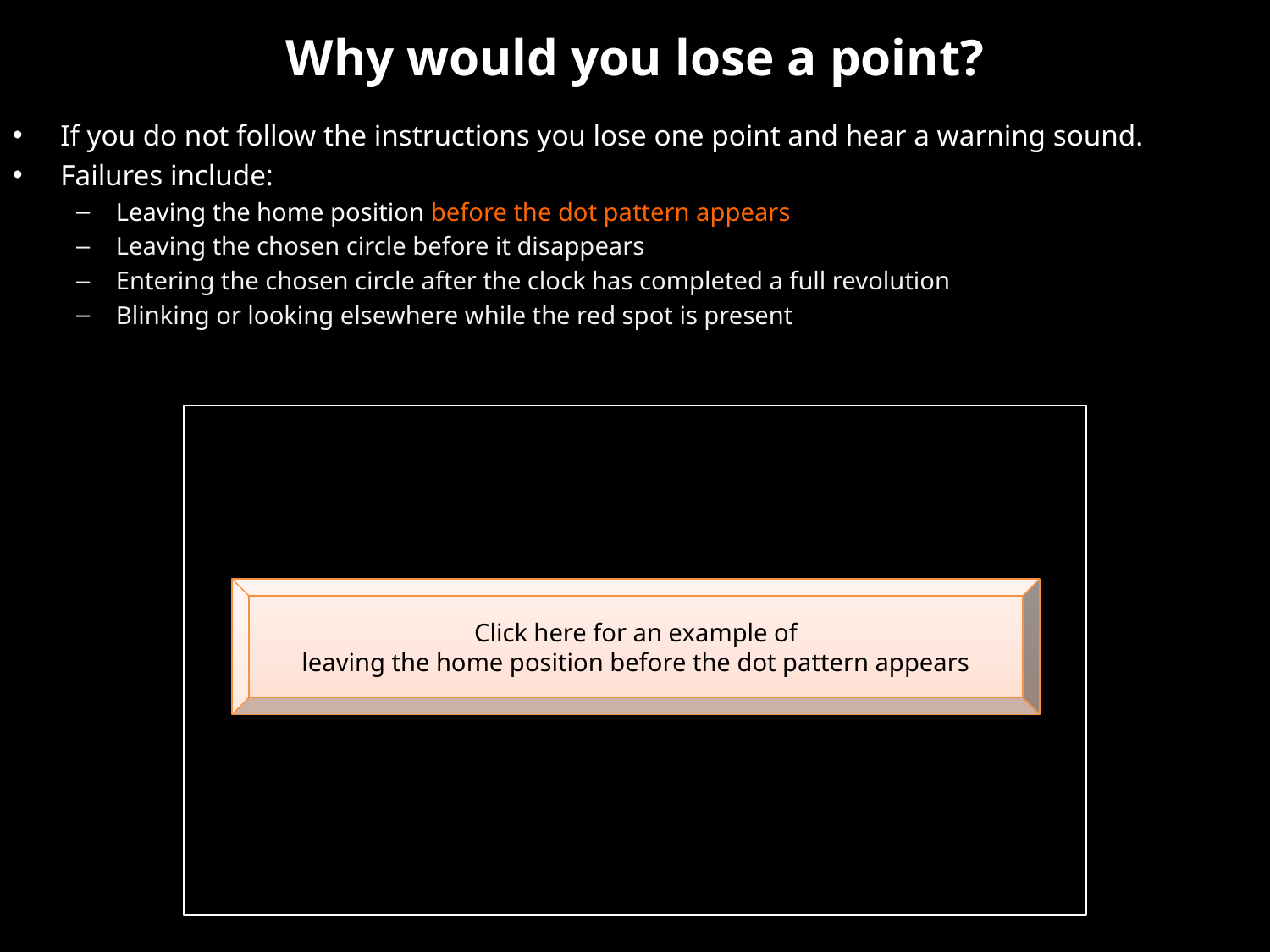

# Why would you lose a point?
If you do not follow the instructions you lose one point and hear a warning sound.
Failures include:
Leaving the home position before the dot pattern appears
Leaving the chosen circle before it disappears
Entering the chosen circle after the clock has completed a full revolution
Blinking or looking elsewhere while the red spot is present
Click here for an example of
leaving the home position before the dot pattern appears

## Slide 124
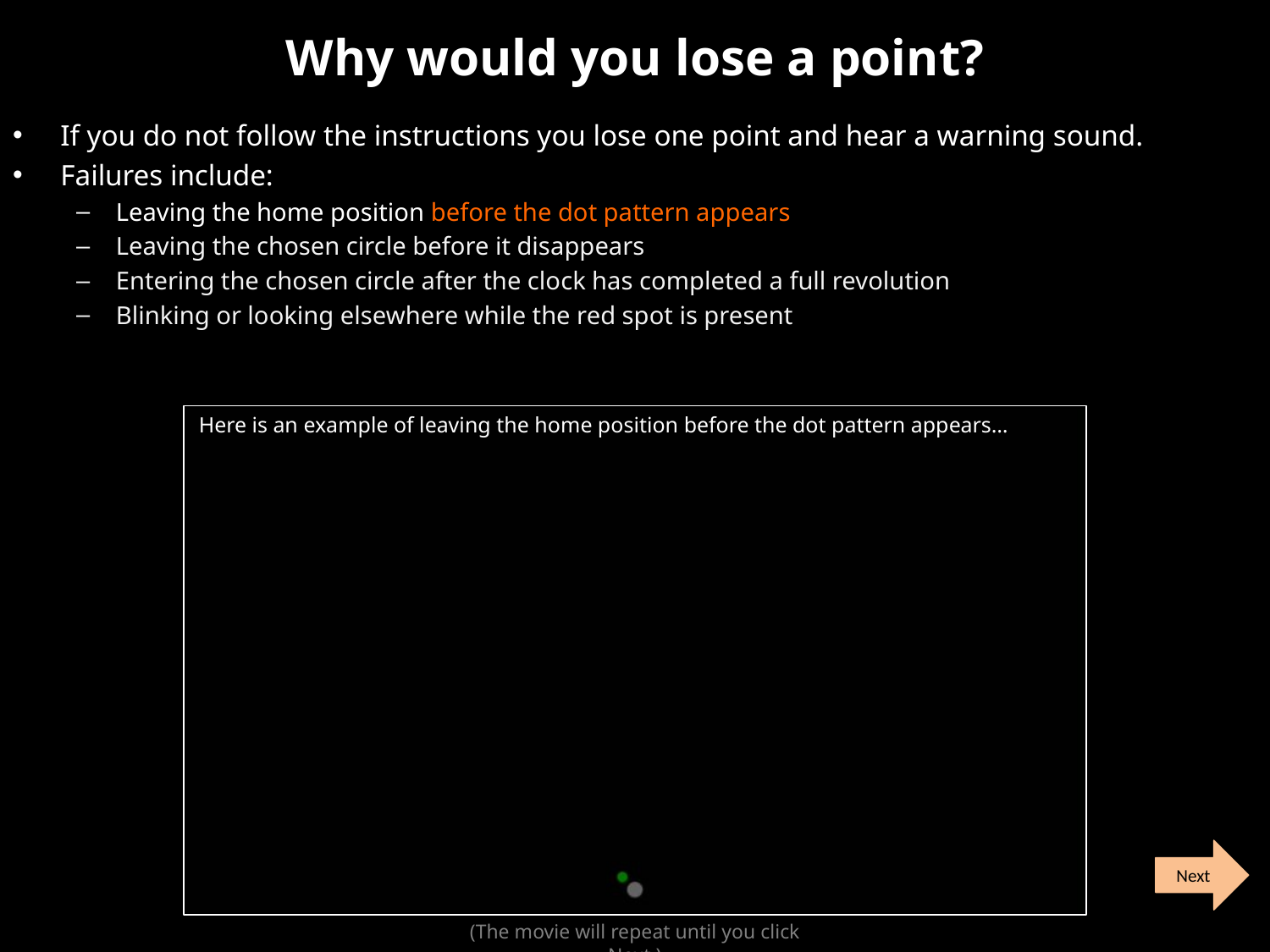

# Why would you lose a point?
If you do not follow the instructions you lose one point and hear a warning sound.
Failures include:
Leaving the home position before the dot pattern appears
Leaving the chosen circle before it disappears
Entering the chosen circle after the clock has completed a full revolution
Blinking or looking elsewhere while the red spot is present
Here is an example of leaving the home position before the dot pattern appears…

## Slide 125
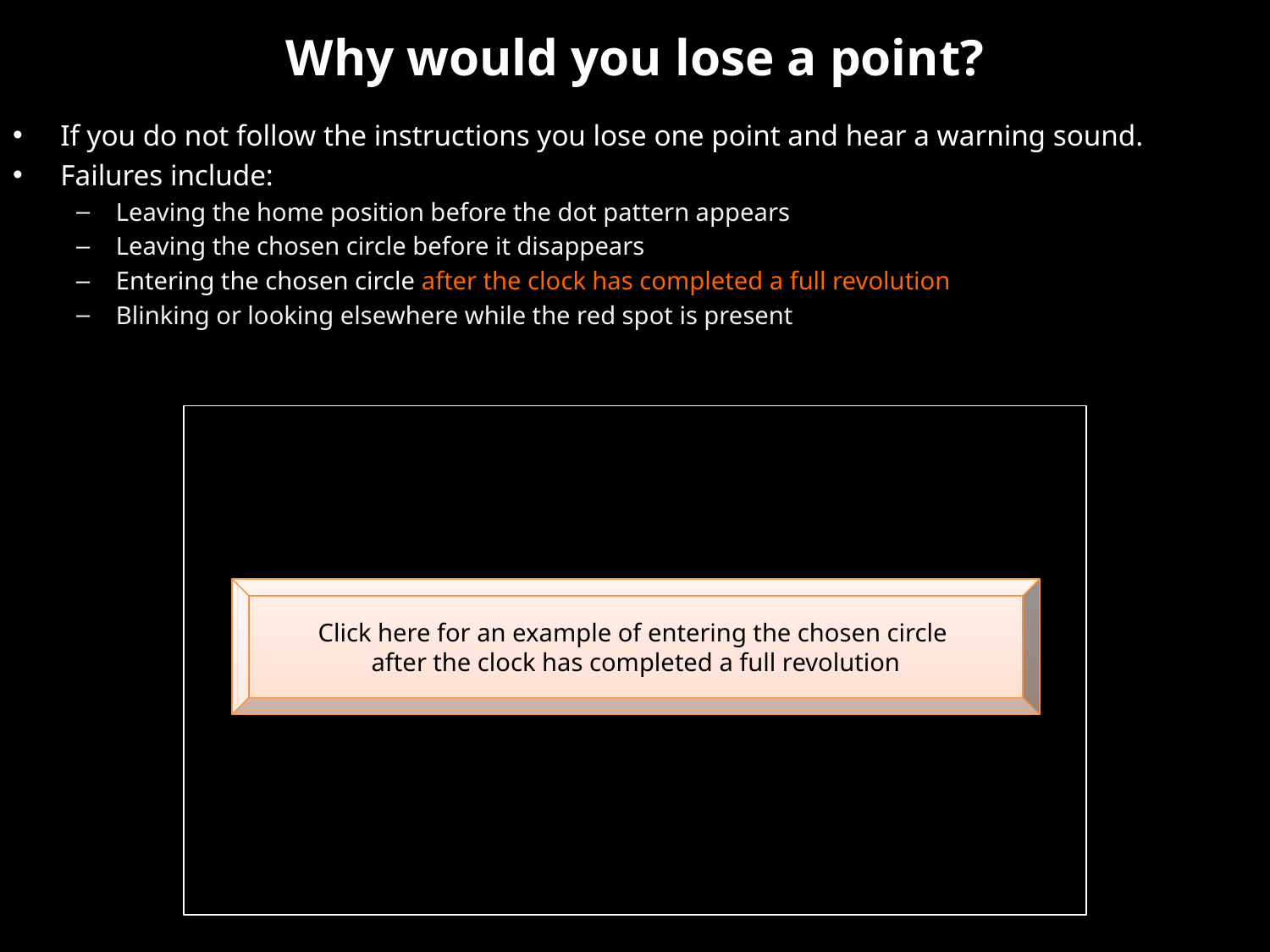

# Why would you lose a point?
If you do not follow the instructions you lose one point and hear a warning sound.
Failures include:
Leaving the home position before the dot pattern appears
Leaving the chosen circle before it disappears
Entering the chosen circle after the clock has completed a full revolution
Blinking or looking elsewhere while the red spot is present
Click here for an example of entering the chosen circle
after the clock has completed a full revolution

## Slide 126
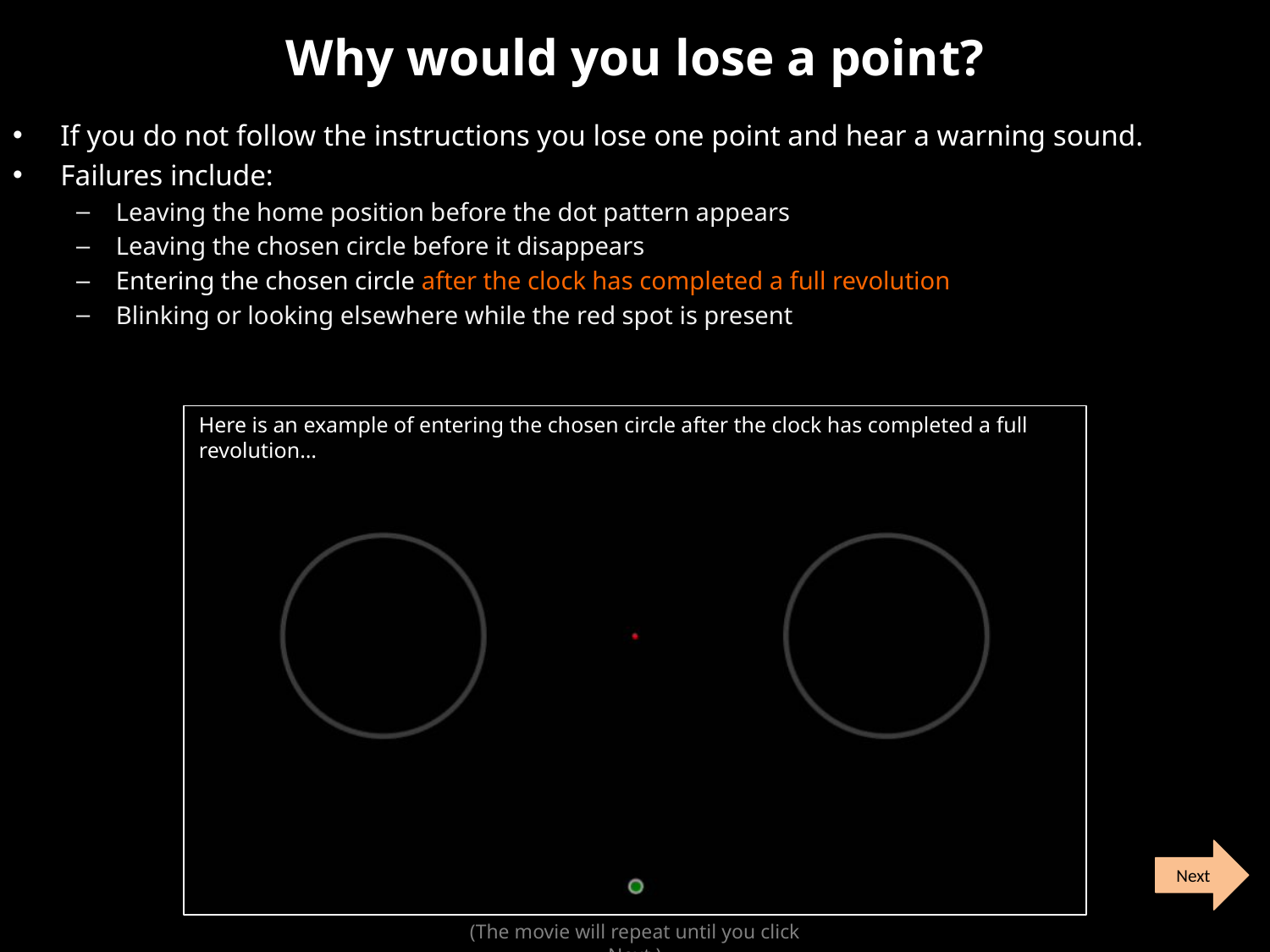

# Why would you lose a point?
If you do not follow the instructions you lose one point and hear a warning sound.
Failures include:
Leaving the home position before the dot pattern appears
Leaving the chosen circle before it disappears
Entering the chosen circle after the clock has completed a full revolution
Blinking or looking elsewhere while the red spot is present
Here is an example of entering the chosen circle after the clock has completed a full revolution…

## Slide 127
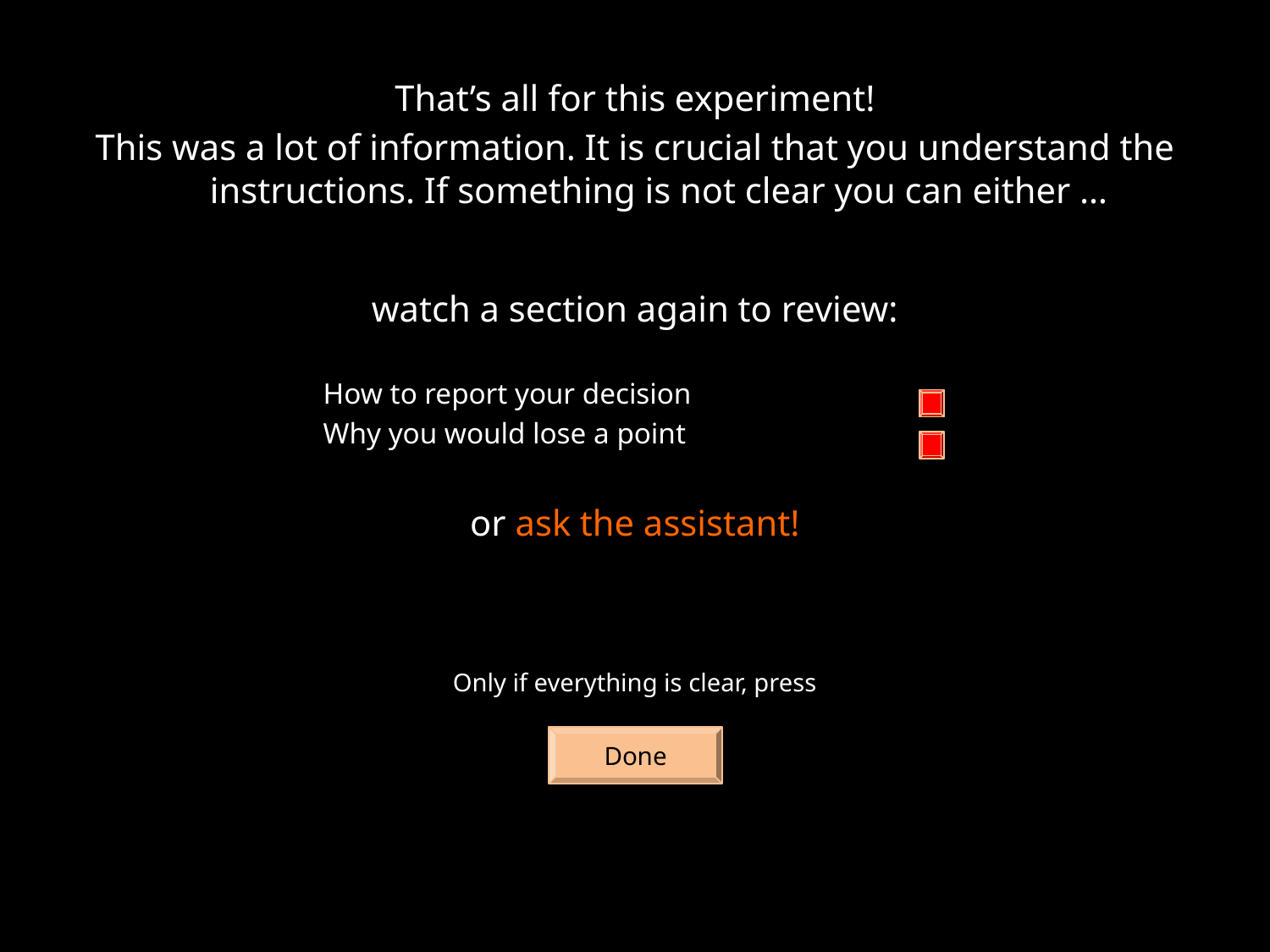

That’s all for this experiment!
This was a lot of information. It is crucial that you understand the instructions. If something is not clear you can either …
watch a section again to review:
How to report your decision
Why you would lose a point
or ask the assistant!
Only if everything is clear, press
Done

## Slide 128
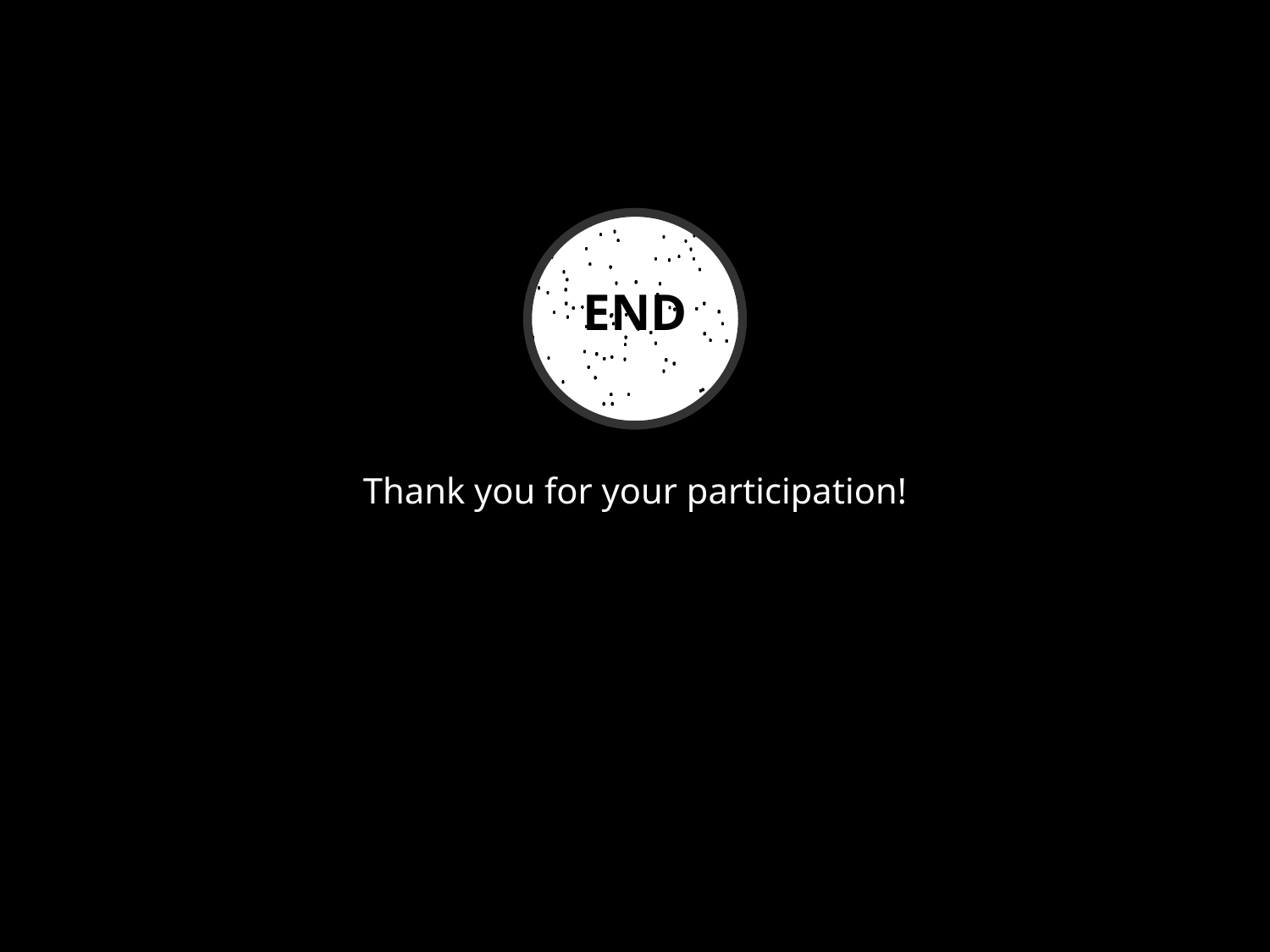

# END
Thank you for your participation!

## Slide 129
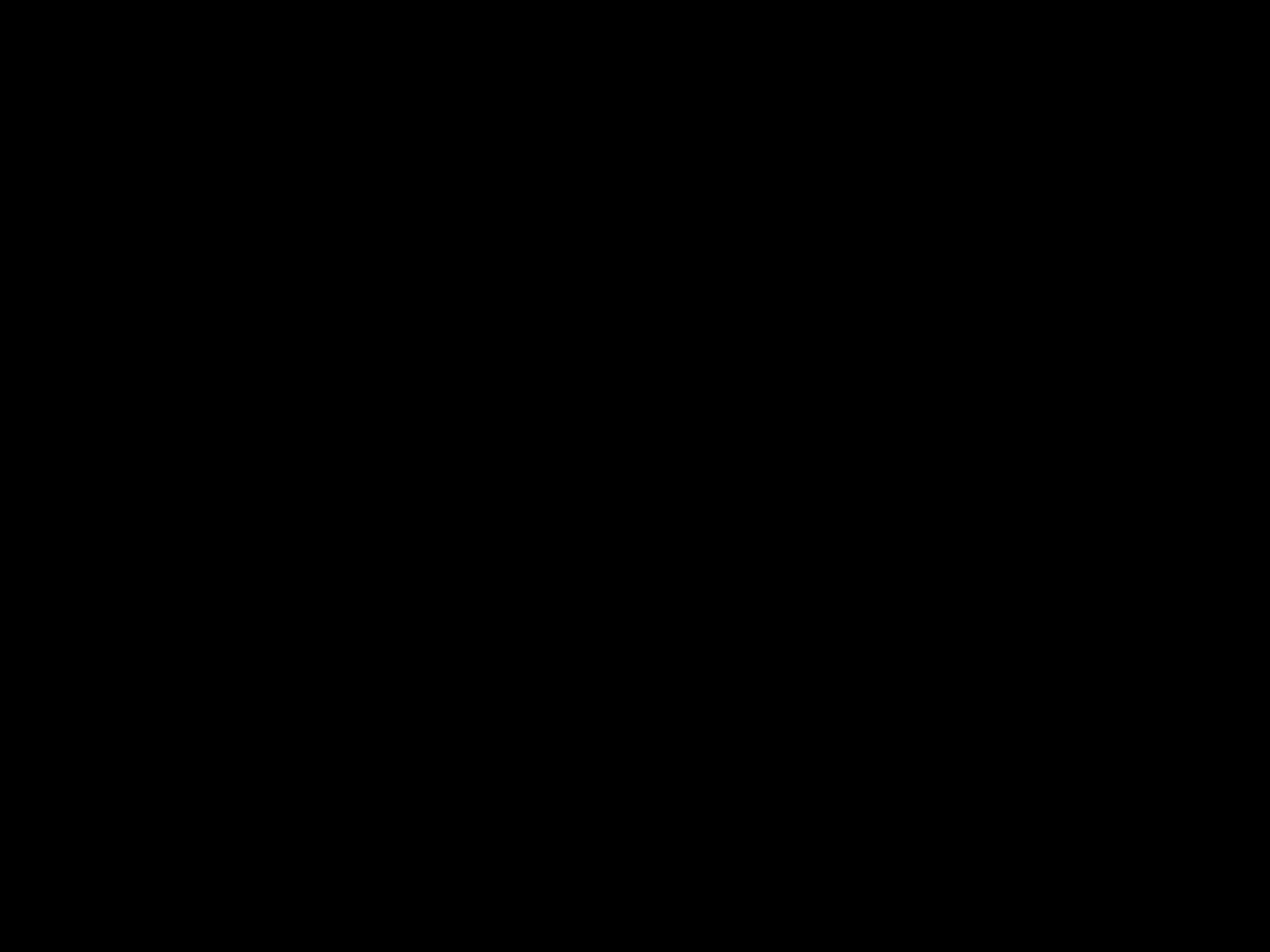

Supplement: Methods S2. tSD Instructions [file mmc3.pptx]
